# 1,6-Nucleophilic Di- and Trifluoromethylation of *para*-Quinone Methides with Me_3_SiCF_2_H/Me_3_SiCF_3_ Facilitated by CsF/18-Crown-6

**DOI:** 10.3390/molecules29122905

**Published:** 2024-06-19

**Authors:** Dingben Chen, Ling Huang, Mingyu Liang, Xiaojing Chen, Dongdong Cao, Pan Xiao, Chuanfa Ni, Jinbo Hu

**Affiliations:** 1 School of Pharmaceutical and Chemical Engineering, Taizhou University, Taizhou 318000, China; 2 Key Laboratory of Fluorine and Nitrogen Chemistry and Advanced Materials, Shanghai Institute of Organic Chemistry, University of Chinese Academy of Sciences, Chinese Academy of Sciences, 345 Lingling Road, Shanghai 200032, China; 3 School of Physical Science and Technology, ShanghaiTech University, 100 Haike Road, Shanghai 201210, China

**Keywords:** difluoromethylation, trifluoromethylation, Me_3_SiCF_2_H/Me_3_SiCF_3_, 1,6-nucleophilic addition, CsF/18-crown-6

## Abstract

The direct 1,6-nucleophilic difluoromethylation, trifluoromethylation, and difluoroalkylation of *para*-quinone methides (*p*-QMs) with Me_3_SiRf (Rf = CF_2_H, CF_3_, CF_2_CF_3_, CF_2_COOEt, and CF_2_SPh) under mild conditions are described. Although Me_3_SiCF_2_H shows lower reactivity than Me_3_SiCF_3_, it can react with *p*-QMs promoted by CsF/18-Crown-6 to give structurally diverse difluoromethyl products in good yields. The products can then be further converted into fluoroalkylated *para*-quinone methides and *α*-fluoroalkylated diarylmethanes.

## 1. Introduction

Organofluorine compounds have been widely applied in various fields, including pharmaceuticals, agrochemicals, materials, surfactants, and catalysis, thanks to the unique properties of fluorine [1,2,3,4,5,6,7,8,9]. The incorporation of fluorine atoms or fluorinated moieties is recognized for its ability to significantly enhance the metabolic stability, lipophilicity, and binding properties of bioactive organic molecules [10,11,12,13]. Among the various fluorinated moieties, the di- and trifluoromethyl groups have garnered considerable attention due to their utilization in numerous drugs and pesticides, such as efavirenz (HIV-RT inhibitor), mefloquine (antimalarial), eflornithine (ODC inhibitor), roflumilast (drug for COPD), fluxapyroxad (fungicide), and thiazopyr (herbicide) [3,14,15,16,17]. Consequently, developing new methods for the efficient introduction of di- and trifluoromethyl groups into organic molecules holds significant synthetic interest. 

Nucleophilic fluoroalkylation has proven to be a convenient method for preparing fluorinated compounds [18,19,20,21]. Among the various nucleophilic fluoroalkylating agents, Ruppert–Prakash reagent (Me_3_SiCF_3_) is the most popular trifluoromethylating agent, widely employed for direct nucleophilic trifluoromethylation of aldehyde, ketone, imine, ester, and amide substrates, etc. [22,23]. However, compared with Me_3_SiCF_3_, the silane reagent Me_3_SiCF_2_H exhibits lower reactivity due to the relatively weak electron-withdrawing ability of the CF_2_H group, which makes cleavage of the Si-CF_2_H bond more difficult than that of the Si-CF_3_ bond [24]. Therefore, the synthetic application of Me_3_SiCF_2_H in nucleophilic difluoromethylation has been largely retarded [25,26,27,28,29,30,31,32,33,34,35,36].

In 2011, our group first demonstrated the effectiveness of utilizing Me_3_SiCF_2_H in nucleophilic difluoromethylation activated by CsF or *t*BuOK under mild conditions [25]. This discovery made people realize that Me_3_SiCF_2_H could be used as an efficient difluoromethylation reagent. Subsequently, in 2016, our group conducted in-depth research on the 1,2-addition of Me_3_SiCF_2_H to enolizable ketones. We found that CsF/18-crown-6 acts as an initiation system to produce a pentavalent silicon reactive intermediate [(18-crown-6)Cs]^+^[(CH_3_)_3_Si(CF_2_H)_2_]^−^, which serves as a temporary reservoir for the difluoromethyl anion, playing a pivotal role in the success of the difluoromethylation in enolizable ketones [37]. In recent years, other strong basic initiators, such as *t*Bu-P_4_ and *t*-AmOK, among others, have been developed for various difluoromethylations with TMSCF_2_H [26,30,31]. However, identifying appropriate initiators to facilitate the difluoromethylation of base-sensitive substrates with Me_3_SiCF_2_H remains a formidable challenge.

**Scheme 1:**
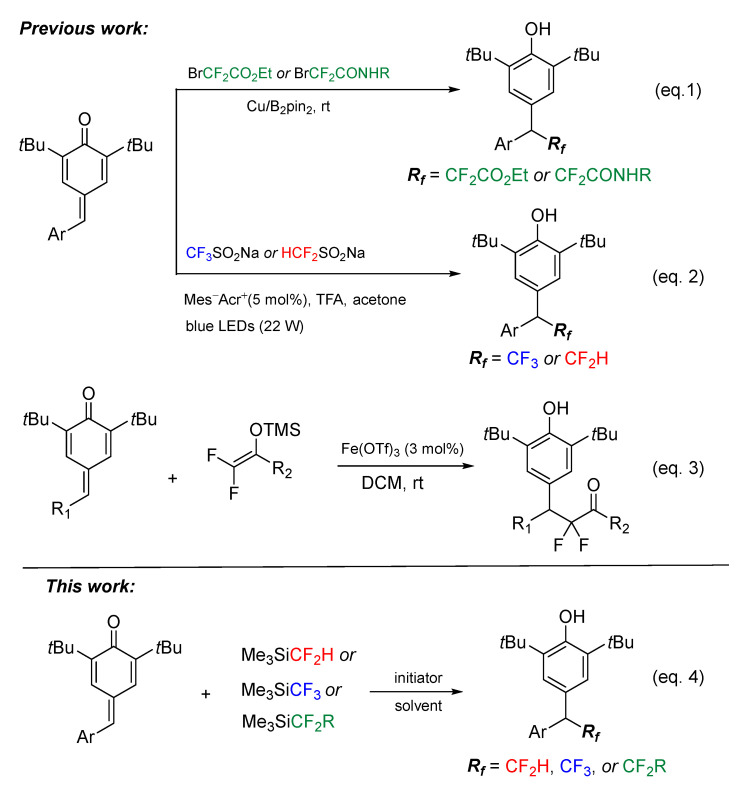
The reactions between *p*-QMs and different fluorine reagents.

Due to the hard nature of fluoroalkyl anions, the direct regioselective 1,4-nucleophilic fluoroalkylation of α,β-unsaturated carbonyl compounds is a challenging task [38,39,40,41], and 1,4-nucleophilic fluoroalkylations are often accompanied with a 1,2-addition reaction [39,40]. Moreover, 1,4-trifluoromethylation of Me_3_SiCF_3_ activated by AcONa or TBAF [38] is mainly limited to electron-deficient olefins containing two electron-withdrawing groups. However, weak basic initiators struggle to cleave the Si-CF_2_H bond of Me_3_SiCF_2_H, and using Me_3_SiCF_2_H to engage in 1,4-/1,6-nucleophilic addition of α,β-unsaturated carbonyl compounds is difficult and has not been reported previously. *para*-Quinone methides (*p*-QMs), often used as excellent receptors in Michael reactions, can be used as a potentially unique raw material for the synthesis of natural and bioactive diarylmethane compounds [42,43,44,45,46]. The radical reactions of *p*-QMs with fluoroalkylation reagents have been reported [47,48,49,50,51]; for instance, Song et al. reported the radical 1,6-hydrodifluoroacetylation of *p*-QMs with difluoroalkyl bromides and bis(pinacolato) diboron (B_2_pin_2_) via copper catalysis (Equation (1), Scheme 1) [47]. Liu et al. described the radical tri-/difluoromethylation of *p*-QMs using sodium tri-/difluoromethanesulfinate via organic photoredox catalysis (Equation (2)) [49]. In addition, Zhou et al. developed the Fe(III)-catalyzed 1,6-conjugate addition of *p*-QMs with fluorinated silyl enol ethers toward β,β-diaryl α-fluorinated ketones (Equation (3)) [52]. However, to the best of our knowledge, there are no reports on the 1,6-nucleophilic difluoromethylation of *p*-QMs with less reactive Me_3_SiCF_2_H. Herein, we report CsF/18-crown-6 facilitated 1,6-nucleophilic difluoromethylation of *p*-QMs under mild conditions, and the trifluoromethylation and difluoroalkylation of *p*-QMs are also presented.

## 2. Results

We initiated the study by optimizing the reaction conditions, including the choice of initiators, temperature, and solvents, using 4-benzylidene-2,6-di-*tert*-butylcyclohexa-2,5-dien-1-one (**1a**) as the model substrate and Me_3_SiCF_2_H as the difluoromethylation reagent (Table 1). We first performed the reaction under the previously reported conditions for the direct nucleophilic difluoromethylation of enolizable ketones, which involved using 0.2 equiv. of CsF/18-crown-6 (1:1) and THF as the solvent at room temperature [37]. However, we did not observe any product (entry 1). Then, by gradually increasing the temperature from −15 °C to room temperature and using DMF as the solvent, along with 0.2 equiv. of TBAF, CsF, or TMAF as the initiator, the product 2,6-di-*tert*-butyl-4-(2,2-difluoro-1-phenethyl) phenol **2a** was obtained in approximately 30% yield (entries 2, 3, and 5). When 0.2 equiv. of TBAF was used as the initiator, the yield of the product was significantly low, irrespective of the temperature (entries 4 and 7). Similarly, using KF as the initiator only yielded trace amounts of product (entry 6). In contrast, when 0.2 equiv. of CsF, 0.1 equiv. of 18-crown-6, and DMF were employed, the yield increased to 42% at −30 °C (entry 10). With 1.0 equiv of CsF/18-crown-6 (1:1), the yield of product reached 60% within a temperature range of −15 °C to room temperature (entry 12). Further, when 1.5 equiv. of CsF/18-crown-6 (1:1) was used, the yield increased up to 70% (entry 13). However, 2.0 equiv. of the initiator CsF/18-crown-6 (1:1) caused a decrease in the yield (60%, entry 14). A 1.5 equiv. amount of KF/18-crown-6 (1:1) was also not suitable for the reaction (12%, entry 15). Therefore, the optimum conditions for this experiment were 1.0 equiv. of **1a**, 2.0 equiv. of Me_3_SiCF_2_H, 1.5 equiv. of CsF/18-crown-6 (1:1), and running the reaction in DMF at temperatures ranging from −15 °C to room temperature overnight.

We next investigated the substrate scope of the direct nucleophilic difluoromethylation between Me_3_SiCF_2_H and 4-benzylidene-2,6-di-*tert*-butylcyclohexa-2,5-dien-1-one derivatives (Table 2). Using the above optimized conditions, as shown in Table 2, most of the substrates examined provided good yields. A series of *p*-QMs bearing electron-donating groups (R = 4-Me, 4-*t*Bu, and 4-OMe) (**2d** and **2i**–**2j**) produced the corresponding products in somewhat lower yields than *p*-QMs bearing electron-withdrawing groups (R = 4-F, 4-Cl, and 4-Br) (**2e**, **2f**, and **2k**). Among them, the 4-Cl substituted product 2,6-di-*tert*-butyl-4-[1-(4-chlorophenyl)-2,2-difluoroethyl]phenol (**2f**) was obtained in the highest yield of 86%. Among the *o*-, *m*-, and *p*-Me-substituted substrates examined in the reaction (**2b**–**2d**), the substrate with the *o*-Me substituent gave the corresponding product in the highest yield (70%). Additionally, when the benzene ring was replaced by naphthalene, *tert*-butyl, and pyridine moiety, the corresponding products were generated with yields of 61%, 23%, and 62%, respectively (**2l**–**2n**).

Furthermore, we extended our investigation to the nucleophilic trifluoromethylation of various *p*-QMs using Me_3_SiCF_3_ under similar reaction conditions (Table 3). A comparison of Table 2 with Table 3 indicates that there are some differences between the di- and trifluoromethylation of *p*-QMs; for instance, a series of *p*-QMs bearing Me groups (R = *o*-, *m*-, and *p*-Me) produced the corresponding trifluoromethylation products (Table 3, **3b**–**3d**) in higher yields than the difluoromethyl products (Table 2, **2b**–**2d**). As shown in Table 3, *p*-QMs bearing electron-donating groups (R = 4-*t*Bu and 4-OMe) (**3h**–**3i**) generated the corresponding trifluoromethyl products in lower yields than the *p*-QMs bearing other groups (H, Me, and Br) (**3a**–**3d** and **3f**). The other trifluoromethyl products **3j** and **3k** (R = naphthalene and pyridine moiety, respectively) were also obtained with yields of 78% and 30%, respectively.

**Scheme 2:**
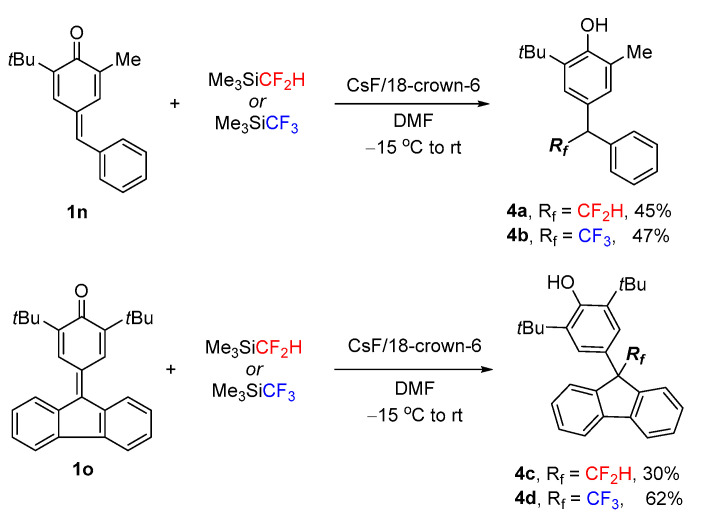
Direct nucleophilic di-/trifluoromethylation reactions of other *p*-QMs with Me_3_SiCF_2_H/Me_3_SiCF_3_.

The nucleophilic di-/trifluoromethylation reactions of other *p*-QMs with Me_3_SiRf (Rf = CF_2_H or CF_3_) were also investigated (Scheme 2). 4-Benzylidene-2-(*tert*-butyl)-6-methylcyclohexa-2,5-dien-1-one (**1n**) gave the corresponding di-/trifluoromethyl products **4a** and **4b** in 45–47% yields, which are significantly lower than those obtained with 4-benzylidene-2,6-di-*tert*-butylcyclohexa-2,5-dien-1-one (**1a**). 2,6-Di-*tert*-butyl-4-(9*H*-fluoren-9-ylidene) cyclohexa-2,5-dien-1-one (**1o**) could be engaged in reactions with Me_3_SiRf (Rf = CF_2_H or CF_3_) to form di- and trifluoromethyl products containing quaternary carbon centers. The yield of the trifluoromethyl product **4d** was much higher than that of the difluoromethyl product **4c**, indicating that Me_3_SiCF_3_ is more reactive than Me_3_SiCF_2_H.

In addition, as illustrated in Table 4, other fluoroalkyl silane reagents Me_3_SiRf (Rf = CF_2_CF_3_, CF_2_COOEt, and CF_2_SPh) could also react with *p*-QMs to generate the corresponding **5** products in 60–88% yields. It is noteworthy that the heterocycle-containing substrates are also compatible with the reaction conditions (**5d** and **5e**).

**Scheme 3:**
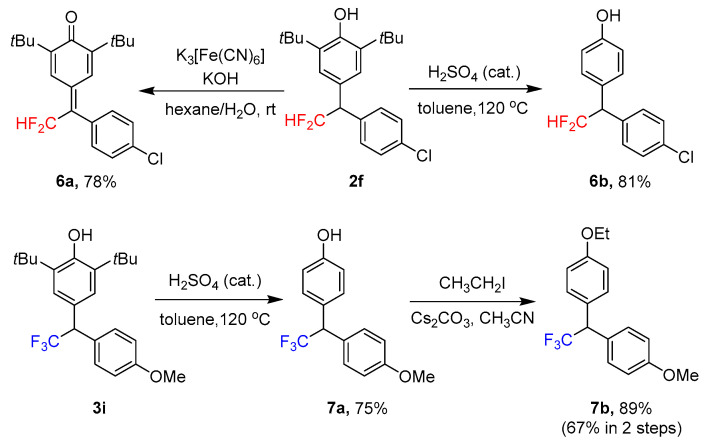
Synthetic applications of di- and trifluoromethylated *p*-quinone methides.

Finally, to showcase the practical utility of the fluoroalkylation products, we explored their further transformations (Scheme 3). Oxidation of **2f** with 4 equiv. of K_3_[Fe(CN)_3_] and KOH in a 1:1 mixture of hexane and H_2_O (*v*/*v*) at room temperature afforded difluoromethylated *p*-QM **6a** in 78% yield. De-*tert*-butylation of **2f** using a catalytic amount of H_2_SO_4_ at 120 °C provided **6b** in 81% yield. Notably, *α*-difluoromethylated diarylmethanes possess potent cytotoxic activity against HCT116 cells [53,54]. Moreover, we applied our protocol in the synthesis of a fluorinated analogue of the insecticide 1,1,1-trichloro-2,2-bis(*p*-chloro phenyl)ethane (DDT) [55]. Here, treatment of the trifluoromethylation product **3i** with H_2_SO_4_ followed by ethylation produced the DDT analogue **7b** in 67% overall yield.

## 3. Materials and Methods

### 3.1. General Information

All reactions were carried out in oven-dried glassware under nitrogen atmosphere. Commercially available reagents were used without further purification. *para*-Quinone methides were prepared according to the reported literature [56]. The solvent DMF was dried over CaH_2_ and distilled under reduced pressure. Column chromatography was performed with 300–400 mesh silica gel. All melting points are uncorrected. ^1^H, ^13^C, and ^19^F NMR spectra were recorded on a 400 MHz NMR spectrometer (Brucker, Karlsruhe, Germany). TLC was carried out with 0.2-millimeter-thick silica gel plates (GF254). Visualization was accomplished by UV light. Mass spectra were obtained on a mass spectrometer. High-resolution mass data were recorded on a high-resolution mass spectrometer in ESI positive ion mode (Q Exactive HF Orbitrap, Thermo Fisher Scientific, Waltham, MA, USA).

### 3.2. General Procedure

#### 3.2.1. Experimental Procedures for the Synthesis of **2**–**5**

Under nitrogen atmosphere, *para*-quinone methide 1 (0.4 mmol), CsF (91.14 mg, 0.6 mmol), and 18-crown-6 (158.6 mg, 0.6 mmol) were added into a Schlenk tube. The Schlenk tube was placed in a cold bath and stirred at −15 °C, and then DMF (2 mL) and TMSCF_2_H (100 mg, 107 μL, 0.80 mmol), TMSCF_3_ (114 mg, 118 μL, 0.80 mmol), or TMSCF_2_R (0.80 mmol) were added. The reaction mixture was gradually warmed to room temperature and stirred overnight. Subsequently, HCl aq. (1.0 M, 1.0 mL) was added at room temperature and the above mixture was stirred for another 15 min. Finally, the mixture was extracted with methyl *tert*-butyl ether (3 × 20 mL). The organic phase was washed with brine and then dried over anhydrous Na_2_SO_4_. After filtration and evaporation under vacuum, the residue was subjected to silica gel column chromatography using hexane/dichloromethane (4:1-1:1, *v*/*v*) as an eluent to give products **2**–**5**.

*2,6-Di-tert-butyl-4-(2,2-difluoro-1-phenylethyl)phenol* (**2a**) [49]: 97 mg, 70% yield. Yellow oil. Purification by column chromatography (hexane/dichloromethane = 4:1, *v*/*v*). **^1^H NMR** (400 MHz, CDCl_3_): *δ* 7.34–7.32 (m, 4H), 7.30–7.26 (m, 1H), 7.09 (s, 2H), 6.25 (td, *J* = 56.1, 4.4 Hz, 1H), 5.16 (s, 1H), 4.30 (td, *J* = 16.2, 4.3 Hz, 1H), 1.41 (s, 18H). **^13^C{^1^H} NMR** (101 MHz, CDCl_3_): *δ* 153.1, 137.6 (t, *J* = 3.4 Hz), 135.9, 129.1, 128.5, 127.6 (t, *J* = 3.8 Hz), 127.2, 125.6, 117.3 (t, *J* = 244.5 Hz), 55.1 (t, *J* = 20.4 Hz), 34.4, 30.2. **^19^F NMR** (376 MHz, CDCl_3_): *δ* −117.13 (ddd, *J* = 276.9, 56.1, 15.6 Hz, 1F), −118.28 (ddd, *J* = 276.9, 56.1, 17.0 Hz, 1F). **HRMS (ESI)** *m*/*z*: [M − H]^+^ calcd. for C_22_H_27_F_2_O, 345.2030; found, 345.2039.

*2,6-Di-tert-butyl-4-(2,2-difluoro-1-(o-tolyl)ethyl)phenol* (**2b**): 101 mg, 70% yield. Yellow solid. M.p.: 75–76 °C. Purification by column chromatography (hexane/dichloromethane = 4:1, *v*/*v*). **^1^H NMR** (400 MHz, CDCl_3_): *δ* 7.39 (d, *J* = 7.6 Hz, 1H), 7.24 (d, *J* = 3.8 Hz, 1H), 7.17 (d, *J* = 4.1 Hz, 2H), 7.04 (s, 2H), 6.30 (td, *J* = 56.2, 5.0 Hz, 1H), 5.14 (s, 1H), 4.53–4.45 (m, 1H), 2.29 (s, 3H), 1.38 (s, 18H). **^13^C{^1^H} NMR** (101 MHz, CDCl_3_): *δ* 152.9, 136.7, 136.4 (t, *J* = 3.2 Hz), 135.7, 130.8, 127.3, 127.0, 126.9 (td, *J* = 4.3, 2.8 Hz), 126.0, 125.8, 117.6 (t, *J* = 243.8 Hz), 50.7 (t, *J* = 20.8 Hz), 34.3, 30.2, 20.0. **^19^F NMR** (376 MHz, CDCl_3_): *δ* −116.62 (dd, *J* = 56.2, 14.4 Hz, 1F), −118.63 (ddd, *J* = 276.1, 56.1, 16.5 Hz, 1F). **HRMS (ESI)** *m*/*z*: [M − H]^+^ calcd. for C_23_H_29_F_2_O, 359.2186; found, 359.2187.

*2,6-Di-tert-butyl-4-(2,2-difluoro-1-(m-tolyl)ethyl)phenol* (**2c**): 66 mg, 46% yield. Yellow oil. Purification by column chromatography (hexane/dichloromethane = 4:1, *v*/*v*). **^1^H NMR** (400 MHz, CDCl_3_): *δ* 7.22 (d, *J* = 7.8 Hz, 1H), 7.14–7.12 (m, 2H), 7.12–7.08 (m, 3H), 6.24 (td, *J* = 56.2, 4.5 Hz, 1H), 5.17 (s, 1H), 4.25 (td, *J* = 16.1, 4.5 Hz, 1H), 2.34 (s, 3H), 1.41 (s, 18H). **^13^C{^1^H} NMR** (101 MHz, CDCl_3_): *δ* 153.0, 138.1, 137.5 (t, *J* = 3.4 Hz), 135.8, 129.9, 128.4, 128.0, 127.6 (t, *J* = 3.8 Hz), 125.8, 125.6, 117.3 (t, *J* = 244.5 Hz), 55.1 (t, *J* = 20.4 Hz), 34.3, 30.2, 21.5. **^19^F NMR** (376 MHz, CDCl_3_): *δ* (−116.75)–(−117.65) (m, 1F), (−117.66)–(−118.5) (m, 1F). **HRMS (ESI)** *m*/*z*: [M − H]^+^ calcd. for C_23_H_29_F_2_O, 359.2186; found, 359.2187.

*2,6-Di-tert-butyl-4-(2,2-difluoro-1-(p-tolyl)ethyl)phenol* (**2d**) [49]: 71 mg, 49 yield. Yellow solid. M.p.: 62–63 °C. Purification by column chromatography (hexane/dichloromethane = 4:1, *v*/*v*). **^1^H NMR** (400 MHz, CDCl_3_): *δ* 7.22 (d, *J* = 8.0 Hz, 2H), 7.15 (d, *J* = 8.0 Hz, 2H), 7.09 (s, 2H), 6.23 (td, *J* = 56.2, 4.4 Hz, 1H), 5.16 (s, 1H), 4.26 (td, *J* = 16.3, 4.3 Hz, 1H), 2.33 (s, 3H),1.41 (s, 18H). **^13^C{^1^H} NMR** (101 MHz, CDCl_3_): *δ* 153.0, 136.8, 135.8, 134.5 (t, *J* = 3.4 Hz), 129.2, 128.9, 127.8 (t, *J* = 3.7 Hz), 125.6, 117.3 (t, *J* = 244.4 Hz), 54.7 (t, *J* = 20.4 Hz), 34.3, 30.2, 21.0. **^19^F NMR** (376 MHz, CDCl_3_) *δ* −117.44 (dd, *J* = 56.2, 15.6 Hz, 1F), −118.01 (dd, *J* = 56.3, 17.0 Hz. 1F). **HRMS (ESI)** *m*/*z*: [M − H]^+^ calcd. for C_23_H_29_F_2_O, 359.2186; found, 359.2187.

*2,6-Di-tert-butyl-4-(2,2-difluoro-1-(4-fluorophenyl)ethyl)phenol* (**2e**): 114 mg, 78% yield. Yellow oil. Purification by column chromatography (hexane/dichloromethane = 4:1, *v*/*v*). **^1^H NMR** (400 MHz, CDCl_3_): *δ* 7.29 (dd, *J* = 7.8, 5.7 Hz, 2H), 7.06 (s, 2H), 7.03 (t, *J* = 8.6 Hz, 2H), 6.22 (td, *J* = 56.1, 4.1 Hz, 1H), 5.20 (d, *J* = 1.2 Hz, 1H), 4.30 (d, *J* = 6.4 Hz, 1H), 1.41 (s, 18H). **^13^C{^1^H} NMR** (101 MHz, CDCl_3_): *δ* 162.0 (d, *J* = 245.9 Hz), 153.1, 136.0, 133.2 (q, *J* = 3.2 Hz), 130.7 (d, *J* = 8.0 Hz), 127.3 (t, *J* = 3.6 Hz), 125.5, 117.0 (t, *J* = 244.7 Hz), 115.4 (d, *J* = 21.3 Hz), 54.2 (t, *J* = 20.5 Hz), 34.3, 30.2. **^19^F NMR** (376 MHz, CDCl_3_): *δ* (−115.45)–(−115.52) (m, 1F), −116.83 (ddd, *J* = 277.4, 56.0, 14.8 Hz, 1F), −119.06 (ddd, *J* = 277.5, 56.2, 18.1 Hz, 1F). **HRMS (ESI)** *m*/*z*: [M − H]^+^ calcd. for C_22_H_26_F_3_O, 363.1936; found, 363.1941.

*2,6-Di-tert-butyl-4-(1-(4-chlorophenyl)-2,2-difluoroethyl)phenol* (**2f**) [49]: 131 mg, 86% yield. Yellow solid. M.p.: 64–65 °C. Purification by column chromatography (hexane/dichloromethane = 4:1, *v*/*v*). **^1^H NMR** (400 MHz, CDCl_3_) *δ* 7.31 (d, *J* = 8.5 Hz,2H), 7.25 (d, *J* = 8.5 Hz, 2H), 7.05 (s, 2H), 6.22 (td, *J* = 56.0, 4.1 Hz, 1H), 5.20 (s, 1H), 4.28 (td, *J* = 18.3, 14.6, 4.0 Hz, 1H), 1.41 (s, 18H). **^13^C{^1^H} NMR** (101 MHz, CDCl_3_): *δ* 153.2, 136.0, 135.9 (t, *J* = 3.1 Hz), 133.2, 130.5, 128.6, 127.1 (t, *J =* 4.4 Hz), 125.5, 116.9 (t, *J* = 244.7 Hz), 54.3 (t, *J* = 20.5 Hz), 34.4, 30.2. **^19^F NMR** (376 MHz, CDCl_3_): *δ* −116.73 (ddd, *J* = 277.9, 55.9, 14.5 Hz, 1F), −119.09 (ddd, *J* = 278.0, 56.1, 18.2 Hz, 1F). **HRMS (ESI)** *m*/*z*: [M − H]^+^ calcd. for C_22_H_26_ClF_2_O, 379.1640; found, 379.1642.

*4-(1-(4-Bromophenyl)-2,2-difluoroethyl)-2,6-di-tert-butylphenol* (**2g**): 109 mg, 63% yield. Yellow solid. M.p.: 95–96 °C. Purification by column chromatography (hexane/dichloromethane = 4:1, *v*/*v*). **^1^H NMR** (400 MHz, CDCl_3_): *δ* 7.46 (d, *J* = 8.4 Hz, 2H), 7.20 (d, *J* = 8.3 Hz, 2H), 7.06 (s, 2H), 6.22 (tdd, *J =* 56.1, 4.0, 1.4 Hz, 1H), 5.20 (d, *J* = 1.6 Hz, 1H), 4.27 (t, *J* = 14.4 Hz, 1H), 1.41 (s, 18H). **^13^C{^1^H} NMR** (101 MHz, CDCl_3_): *δ* 153.2, 136.5 (t, *J* = 3.0 Hz), 136.0, 131.6, 130.8, 127.0 (t, *J* = 3.6 Hz), 125.5, 121.3, 116.9 (t, *J* = 244.8 Hz), 54.4 (t, *J* = 20.5 Hz), 34.4, 30.2. **^19^F NMR** (376 MHz, CDCl_3_): *δ* −116.66 (ddd, *J* = 278.1, 55.8, 14.4 Hz, 1F), −119.07 (ddd, *J* = 278.0, 56.1, 18.1 Hz, 1F). **HRMS (ESI)** *m*/*z*: [M − H]^+^ calcd. for C_22_H_26_BrF_2_O, 423.1135; found, 423.1139.

*4-(1-(3-Bromophenyl)-2,2-difluoroethyl)-2,6-di-tert-butylphenol* (**2h**): 115 mg, 68% yield. Yellow solid. M.p.: 86–87 °C. Purification by column chromatography (hexane/dichloromethane = 4:1, *v*/*v*). **^1^H NMR** (400 MHz, CDCl_3_): *δ* 7.47 (s, 1H), 7.42–7.40 (m, 1H), 7.25 (s, 1H), 7.21 (t, *J* = 7.8 Hz, 1H), 7.06 (s, 2H), 6.22 (td, *J* = 55.9, 4.1 Hz, 1H), 5.21 (s, 1H), 4.26 (td, *J* = 18.3, 14.6, 4.1 Hz, 1H), 1.42 (s, 18H). **^13^C{^1^H} NMR** (101 MHz, CDCl_3_): *δ* 153.3, 139.7 (t, *J* = 3.2 Hz), 136.1, 132.3, 130.4, 130.0, 127.7, 126.8 (t, *J* = 3.8 Hz), 125.5, 122.5, 116.8 (t, *J =* 244.9 Hz), 54.7 (t, *J* = 20.6 Hz), 34.4, 30.2. **^19^F NMR** (376 MHz, CDCl_3_): *δ* −116.78 (ddd, *J* = 278.2, 55.9, 14.6 Hz, 1F), −118.85 (ddd, *J* = 278.1, 56.1, 17.9 Hz, 1F). **HRMS (ESI)** *m*/*z*: [M − H]^+^ calcd. for C_22_H_26_BrF_2_O, 423.1135; found, 423.1139.

*2,6-Di-tert-butyl-4-(1-(4-tert-butylphenyl)-2,2-difluoroethyl)phenol* (**2i**): 97 mg, 60% yield. Yellow oil. Purification by column chromatography (hexane/dichloromethane = 4:1, *v*/*v*). **^1^H NMR** (400 MHz, CDCl_3_): *δ* 7.36 (d, *J* = 8.4 Hz, 2H), 7.26 (d, *J* = 8.3 Hz, 2H), 7.11 (s, 2H), 6.23 (td, *J* = 56.3, 4.4 Hz, 1H), 5.16 (s, 1H), 4.26 (td, *J* = 16.4, 4.3 Hz, 1H), 1.41 (s, 18H), 1.30 (s, 9H). **^13^C{^1^H} NMR** (101 MHz, CDCl_3_): *δ* 153.0, 150.0, 135.8, 134.5 (t, *J* = 3.3 Hz), 128.6, 127.7 (t, *J* = 3.7 Hz), 125.6, 125.4, 117.4 (t, *J* = 244.5 Hz), 54.7 (t, *J* = 20.4 Hz), 34.4, 34.3, 31.3, 30.2. **^19^F NMR** (376 MHz, CDCl_3_): *δ* −116.93 (ddd, *J* = 276.0, 56.2, 15.7 Hz, 1F), −118.29 (ddd, *J* = 276.0, 56.3, 17.0 Hz, 1F). **HRMS (ESI)** *m*/*z*: [M − H]^+^ calcd. for C_26_H_35_F_2_O, 401.2656; found, 401.2663.

*2,6-Di-tert-butyl-4-(2,2-difluoro-1-(4-methoxyphenyl)ethyl)phenol* (**2j**): 60 mg, 40% yield. Yellow solid. M.p.: 75–76 °C. Purification by column chromatography (hexane/dichloromethane = 4:1, *v*/*v*). **^1^H NMR** (400 MHz, CDCl_3_): *δ* 7.24 (d, *J* = 8.7 Hz, 2H), 7.09 (s, 2H), 6.88 (d, *J* = 8.7 Hz, 2H), 6.21 (td, *J* = 56.3, 4.3 Hz, 1H), 5.17 (s, 1H), 4.33–4.12 (m, 1H), 3.78 (s, 3H), 1.41 (s, 18H). **^13^C{^1^H} NMR** (101 MHz, CDCl_3_): *δ* 158.7, 153.0, 135.8, 130.1, 129.6 (t, *J* = 3.5 Hz), 127.9 (t, *J* = 3.7 Hz), 125.5, 117.3 (t, *J* = 244.4 Hz), 113.9, 55.2, 54.2 (t, *J* = 20.4 Hz), 34.3, 30.2. **^19^F NMR** (376 MHz, CDCl_3_): *δ* −116.96 (ddd, *J* = 276.2, 56.2, 15.3 Hz, 1F), −118.55 (ddd, *J* = 276.3, 56.3, 17.4 Hz, 1F). **HRMS (ESI)** *m*/*z*: [M − H]^+^ calcd. for C_23_H_29_F_2_O_2_, 375.2136; found, 375.2143.

*2,6-Di-tert-butyl-4-(1-(2,4-dichlorophenyl)-2,2-difluoroethyl)phenol* (**2k**): 111 mg, 67% yield. Yellow oil. Purification by column chromatography (hexane/dichloromethane = 4:1, *v*/*v*). **^1^H NMR** (400 MHz, CDCl_3_): *δ* 7.45–7.40 (m, 2H), 7.29–7.24 (m, 1H), 7.08 (s, 2H), 6.26 (td, *J* = 55.8, 4.1 Hz, 1H), 5.19 (s, 1H), 4.85 (td, *J* = 16.3, 4.0 Hz, 1H), 1.40 (s, 18H). **^13^C{^1^H} NMR** (101 MHz, CDCl_3_): *δ* 153.3, 136.0, 135.3, 134.3 (t, *J* = 3.2 Hz), 133.6, 130.5, 129.8, 127.2, 125.9 (t, *J* = 3.2 Hz), 125.7, 116.7 (t, *J* = 245.2 Hz), 50.2 (t, *J* = 21.1 Hz), 34.3, 30.2. **^19^F NMR** (376 MHz, CDCl_3_): *δ* (−117.38)–(−118.21) (m, 1F), (−118.27)–(−118.69) (m, 1F). **HRMS (ESI)** *m*/*z*: [M − H]^+^ calcd. for C_22_H_26_Cl_2_F_2_O, 413.1251; found, 413.1261.

*2,6-Di-tert-butyl-4-(2,2-difluoro-1-(naphthalen-2-yl)ethyl)phenol* (**2l**): 97 mg, 61% yield. Orange solid. M.p.: 104–105 °C. Purification by column chromatography (hexane/dichloromethane = 5:1, *v*/*v*). **^1^H NMR** (400 MHz, CDCl_3_): *δ* 8.06 (d, *J* = 8.1 Hz, 1H), 7.86 (d, *J* = 7.3 Hz, 1H), 7.80 (d, *J* = 8.1 Hz, 1H), 7.58 (d, *J* = 7.1 Hz, 1H), 7.51–7.46 (m, 3H), 7.15 (s, 2H), 6.43 (td, *J* = 56.0, 4.6 Hz, 1H), 5.13 (s, 1H), 1.37 (s, 18H). **^13^C{^1^H} NMR** (101 MHz, CDCl_3_): *δ* 153.1, 135.7, 134.1, 133.8 (t, *J* = 3.7 Hz), 131.8, 128.9, 127.9, 127.1 (t, *J* = 3.6 Hz), 126.3, 125.8, 125.6, 125.4, 125.2, 123.4, 117.6 (t, *J* = 244.3 Hz), 50.0 (t, *J* = 20.9 Hz), 34.3, 30.2. **^19^F NMR** (376 MHz, CDCl_3_): *δ* –115.55 (ddd, *J* = 275.9, 56.1, 13.6 Hz), –118.53 (ddd, *J* = 276.0, 56.0, 17.2 Hz). **HRMS (ESI)** *m*/*z*: [M − H]^+^ calcd. for C_26_H_29_F_2_O, 395.2186; found, 395.2192.

*2,6-Di-tert-butyl-4-(1,1,1,3,3-pentafluoropropan-2-yl)phenol* (**2m**): 30 mg, 23% yield. Yellow oil. Purification by column chromatography (hexane/dichloromethane = 3:1, *v*/*v*). **^1^H NMR** (400 MHz, CDCl_3_): *δ* 6.99 (s, 2H), 6.17 (td, *J* = 56.0, 3.8 Hz, 1H), 5.11 (s, 1H), 2.72 (ddd, *J* = 21.9, 15.1, 3.8 Hz, 1H), 1.43 (s, 18H), 0.98 (s, 9H). **^13^C{^1^H} NMR** (101 MHz, CDCl_3_): *δ* 152.7, 135.0, 126.8, 118.3 (t, *J* = 243.2 Hz), 58.9 (t, *J* = 17.7 Hz), 34.2, 33.6–32.7 (m), 30.4, 28.9. **^19^F NMR** (376 MHz, CDCl_3_): *δ* (−114.45)–(−115.41) (m, 1F), (−115.41)–(−116.36) (m, 1F). **HRMS (ESI)** *m*/*z*: [M − H]^+^ calcd. for C_20_H_31_F_2_O, 325.2343; found, 325.2348.

*2,6-Di-tert-butyl-4-(2,2-difluoro-1-(pyridin-2-yl)ethyl)phenol* (**2n**): 86 mg, 62% yield. Yellow oil. Purification by column chromatography (hexane/dichloromethane = 20:1, *v*/*v*). **^1^H NMR** (400 MHz, CDCl_3_) *δ* 8.65–8.50 (m, 1H), 7.60 (td, *J* = 7.7, 1.8 Hz, 1H), 7.24–7.12 (m, 4H), 6.59 (td, *J* = 56.3, 6.3 Hz, 1H), 5.17 (s, 1H), 4.37 (ddd, *J* = 14.0, 11.6, 6.3 Hz, 1H), 1.40 (s, 18H). **^13^C NMR** (101 MHz, CDCl_3_) *δ* 158.4 (d, *J* = 7.8 Hz), 153.4, 149.2, 136.7, 136.0, 126.9 (d, *J* = 7.0 Hz), 125.8, 124.1, 122.1, 117.9 (t, *J* = 243.3 Hz), 57.1 (t, *J* = 21.7 Hz), 34.3, 30.2. **^19^F NMR** (376 MHz, CDCl_3_) *δ* −117.08 (dd, *J* = 54.5, 11.9 Hz, 1F), −122.47 (dd, *J* = 56.8, 14.3 Hz, 1F). **HRMS (ESI)** *m*/*z*: [M + H]^+^ calcd. for C_21_H_27_F_2_NO, 348.2139; found, 348.2146.

*2,6-Di-tert-butyl-4-(2,2,2-trifluoro-1-phenyl-ethyl)-phenol* (**3a**) [49]: 96 mg, 66% yield. Yellow solid. M.p.: 64–65 °C. Purification by column chromatography (hexane/dichloromethane = 4:1, *v*/*v*). **^1^H NMR** (400 MHz, CDCl_3_): *δ* 7.40–7.29 (m, 5H), 7.15 (s, 2H), 5.19 (s, 1H), 4.56 (q, *J* = 10.2 Hz, 1H), 1.41 (s, 18H). **^13^C{^1^H} NMR** (101 MHz, CDCl_3_): *δ* 153.4, 136.0, 135.9, 129.0, 128.6, 127.9 (q, *J* = 280.5 Hz), 127.6, 125.9, 125.8, 55.5 (q, *J* = 27.3 Hz), 34.3, 30.2. **^19^F NMR** (376 MHz, CDCl_3_): *δ* −65.96 (d, *J* = 10.1 Hz, 3F). **HRMS (ESI)** *m*/*z*: [M − H]^+^ calcd. for C_22_H_26_F_3_O, 363.1936; found, 363.1931.

*2,6-Di-tert-butyl-4-(2,2,2-trifluoro-1-o-tolyl-ethyl)-phenol* (**3b**): 129 mg, 85% yield. Yellow solid. M.p.: 112–114 °C. Purification by column chromatography (hexane/dichloromethane = 4:1, *v*/*v*). **^1^H NMR** (400 MHz, CDCl_3_): *δ* 7.57 (d, *J* = 7.7 Hz, 1H), 7.29–7.13 (m, 3H), 7.11 (s, 2H), 5.17 (s, 1H), 4.79 (q, *J* = 10.2 Hz, 1H), 2.30 (s, 3H), 1.39 (s, 18H). **^13^C{^1^H} NMR** (101 MHz, CDCl_3_): *δ* 152.3, 135.5, 134.7, 133.5, 129.8, 126.5, 126.4, 126.4, 125.7 (q, *J* = 280.78 Hz), 125.2, 125.1, 49.8 (q, *J* = 27.1 Hz), 33.3, 29.2, 19.1. **^19^F NMR** (376 MHz, CDCl_3_): *δ* −65.24 (d, *J* = 10.2 Hz, 3F). **HRMS (ESI)** *m*/*z*: [M − H]^+^ calcd. for C_23_H_28_F_3_O, 377.2092; found, 377.2108.

*2,6-Di-tert-butyl-4-(2,2,2-trifluoro-1-m-tolyl-ethyl)-phenol* (**3c**): 92 mg, 61% yield. Yellow solid. M.p.: 68–70 °C. Purification by column chromatography (hexane/dichloromethane = 4:1, *v*/*v*). **^1^H NMR** (400 MHz, CDCl_3_): *δ* 7.23–7.19 (m, 3H), 7.16 (s, 2H), 7.10 (d, *J* = 6.4 Hz, 1H), 5.19 (s, 1H), 4.51 (q, *J* = 10.2 Hz, 1H), 2.34 (s, 3H), 1.41 (s, 18H). **^13^C{^1^H} NMR** (101 MHz, CDCl_3_): *δ* 152.4, 137.2, 134.8, 134.7, 128.9, 127.4, 127.3, 125.5 (q, *J* = 280.5 Hz), 124.9, 124.8, 124.7, 54.5 (q, *J* = 27.1 Hz), 33.3, 29.2, 20.4. **^19^F NMR** (376 MHz, CDCl_3_): *δ* −65.93 (d, *J* = 10.2 Hz, 3F). **HRMS (ESI)** *m*/*z*: [M − H]^+^ calcd. for C_23_H_28_F_3_O, 377.2092; found, 377.2101.

*2,6-Di-tert-butyl-4-(2,2,2-trifluoro-1-p-tolyl-ethyl)-phenol* (**3d**) [49]: 124 mg, 82% yield. Yellow solid. M.p.: 92–94 °C. Purification by column chromatography (hexane/dichloromethane = 4:1, *v*/*v*). **^1^H NMR** (400 MHz, CDCl_3_): *δ* 7.28–7.24 (m, 2H), 7.15–7.13 (m, 4H), 5.18 (s, 1H), 4.52 (q, *J* = 10.2 Hz, 1H), 2.33 (s, 3H), 1.41 (s, 18H). **^13^C{^1^H} NMR** (101 MHz, CDCl_3_): *δ* 152.3, 136.3, 134.8, 132.0, 128.3, 127.8, 125.5 (q, *J* = 280.4 Hz), 125.1, 124.7, 54.2 (q, *J* = 27.2 Hz), 33.3, 29.2, 20.0. **^19^F NMR** (376 MHz, CDCl_3_): *δ* −65.24 (d, *J* = 10.2 Hz, 3F). **HRMS (ESI)** *m*/*z*: [M − H]^+^ calcd. for C_23_H_28_F_3_O, 377.2092; found, 377.2098.

*2,6-Di-tert-butyl-4-[2,2,2-trifluoro-1-(4-fluoro-phenyl)-ethyl]-phenol* (**3e**) [49]: 87 mg, 57% yield. Yellow solid. M.p.: 84–85 °C. Purification by column chromatography (hexane/dichloromethane = 4:1, *v*/*v*). **^1^H NMR** (400 MHz, CDCl_3_): *δ* 7.35 (dd, *J* = 8.3, 5.4 Hz, 2H), 7.12 (s, 2H), 7.03 (t, *J* = 8.7 Hz, 2H), 5.22 (s, 1H), 4.56 (q, *J* = 10.0 Hz, 1H), 1.41 (s, 18H). **^13^C{^1^H} NMR** (101 MHz, CDCl_3_): *δ* 163.4, 161.0, 153.5, 136.0, 131.9, 130.7 (d, *J* = 8.1 Hz), 126.3 (q, *J* = 281.2, 280.7 Hz), 125.7, 115.5 (d, *J* = 21.5 Hz), 54.7 (q, *J* = 27.4 Hz), 30.2, 18.4. **^19^F NMR** (376 MHz, CDCl_3_): *δ* −66.24 (d, *J* = 10.3 Hz), −114.72 (ddd, *J* = 13.6, 8.6, 5.2 Hz, 3F). **HRMS (ESI)** *m*/*z*: [M − H]^+^ calcd. for C_22_H_25_F_4_O, 381.1842; found, 381.1851.

*4-[1-(4-Bromo-phenyl)-2,2,2-trifluoro-ethyl]-2,6-di-tert-butyl-phenol* (**3f**) [49]: 152 mg, 86% yield. Yellow solid. M.p.: 93–95 °C. Purification by column chromatography (hexane/dichloromethane = 4:1, *v*/*v*). **^1^H NMR** (400 MHz, CDCl_3_): *δ* 7.39 (d, *J* = 8.5 Hz, 2H), 7.18 (d, *J* = 8.4 Hz, 2H), 7.03 (s, 2H), 5.15 (s, 1H), 4.45 (q, *J* = 10.0 Hz, 1H), 1.33 (s, 18H). **^13^C{^1^H} NMR** (101 MHz, CDCl_3_): *δ* 152.5, 135.0, 134.0, 130.7, 129.7, 125.1 (q, *J* = 280.7 Hz), 124.6, 124.3, 120.8, 53.9 (q, *J* = 27.5 Hz), 33.3, 29.2. **^19^F NMR** (376 MHz, CDCl_3_): *δ* −66.08 (d, *J* = 10.2 Hz, 3F). **HRMS (ESI)** *m*/*z*: [M − H]^+^ calcd. for C_22_H_25_BrF_3_O, 441.1041; found, 441.1044.

*2,6-Di-tert-butyl-4-[1-(2,4-dichloro-phenyl)-2,2,2-trifluoro-ethyl]-phenol* (**3g**): 105 mg, 61% yield. Yellow solid. M.p.: 99–100 °C. Purification by column chromatography (hexane/dichloromethane = 4:1, *v*/*v*). **^1^H NMR** (400 MHz, CDCl_3_): *δ* 7.58 (d, *J* = 8.5 Hz, 1H), 7.42 (d, *J* = 2.1 Hz, 1H), 7.28 (dd, *J* = 8.5, 2.1 Hz, 1H), 7.12 (s, 2H), 5.23 (s, 1H), 5.17 (q, *J* = 10.0 Hz, 1H), 1.40 (s, 18H). **^13^C{^1^H} NMR** (101 MHz, CDCl_3_): *δ* 153.7, 136.0, 135.3, 134.1, 132.8, 129.9, 129.8, 127.4, 126.1 (q, *J* = 280.6 Hz), 125.9, 124.2, 50.5 (q, *J* = 28.2 Hz), 34.3, 30.2. **^19^F NMR** (376 MHz, CDCl_3_): *δ* −65.57 (d, *J* = 9.5 Hz, 3F). **HRMS (ESI)** *m*/*z*: [M − H]^+^ calcd. for C_22_H_24_Cl_2_F_3_O, 431.1156; found, 431.1151.

*2,6-Di-tert-butyl-4-[1-(4-tert-butyl-phenyl)-2,2,2-trifluoro-ethyl]-phenol* (**3h**) [50]: 76 mg, 45% yield. Yellow solid. M.p.: 83–85 °C. Purification by column chromatography (hexane/dichloromethane = 4:1, *v*/*v*). **^1^H NMR** (400 MHz, CDCl_3_): *δ* 7.37-7.31 (m, 4H), 7.17 (s, 2H), 5.18 (s, 1H), 4.52 (q, *J* = 10.3 Hz, 1H), 1.41 (s, 18H), 1.30 (s, 9H). **^13^C{^1^H} NMR** (101 MHz, CDCl_3_): *δ* 153.4, 150.5, 135.9, 133.0, 128.6, 128.0 (q, *J* = 280.6 Hz), 126.2, 125.8, 125.5, 55.2 (q, *J* = 27.1 Hz), 34.5, 34.4, 31.3, 30.3. **^19^F NMR** (376 MHz, CDCl_3_): *δ* −66.04 (d, *J* = 10.2 Hz, 3F). **HRMS (ESI)** *m*/*z*: [M − H]^+^ calcd. for C_26_H_34_F_3_O, 419.2562; found, 419.2552.

*2,6-Di-tert-butyl-4-(2,2,2-trifluoro-1-(4-methoxyphenyl)ethyl)phenol* (**3i**) [49]: 87 mg, 55% yield. Yellow oil. Purification by column chromatography (hexane/dichloromethane = 4:1, *v*/*v*). **^1^H NMR** (400 MHz, CDCl_3_): *δ* 7.30 (d, *J* = 8.4 Hz, 2H), 7.14 (s, 2H), 6.88 (d, *J* = 8.8 Hz, 2H), 5.19 (s, 1H), 4.52 (q, *J* = 10.2 Hz, 1H), 3.79 (s, 3H), 1.41 (s, 18H). **^13^C{^1^H} NMR** (101 MHz, CDCl_3_): *δ* 159.0, 153.3, 135.9, 130.2, 128.2, 128.0 (q, *J* = 280.5 Hz), 126.2, 125.6, 55.2, 54.7 (q, *J* = 27.2 Hz), 34.4, 30.2. **^19^F NMR** (376 MHz, CDCl_3_): *δ* −66.24 (d, *J* = 2.7 Hz), −66.27 (d, *J* = 2.7 Hz). **HRMS (ESI)** *m*/*z*: [M − H]^+^ calcd. for C_23_H_28_F_3_O_2_, 393.2041; found, 393.2044.

*2,6-Di-tert-butyl-4-(2,2,2-trifluoro-1-naphthalen-2-yl-ethyl)-phenol* (**3j**) [49]: 129 mg, 78% yield. Yellow solid. M.p.: 145–147 °C. Purification by column chromatography (hexane/dichloromethane = 5:1, *v*/*v*). **^1^H NMR** (400 MHz, CDCl_3_): *δ* 8.02 (d, *J* = 8.3 Hz, 1H), 7.90–7.74 (m, 3H), 7.57–7.43 (m, 3H), 7.21 (s, 2H), 5.44 (q, *J* = 9.9 Hz, 1H), 5.17 (s, 1H), 1.37 (s, 18H). **^13^C{^1^H} NMR** (101 MHz, CDCl_3_): *δ* 153.4, 135.8, 134.1, 131.7, 131.6, 129.1, 128.4, 126.9 (q, *J* = 286.4 Hz), 126.5, 126.0, 125.8, 125.7, 125.6, 125.5, 125.2, 123.1, 50.3 (q, *J* = 27.3 Hz), 34.3, 30.2. **^19^F NMR** (376 MHz, CDCl_3_): *δ* −64.83 (d, *J* = 9.7 Hz, 3F). **HRMS (ESI)** *m*/*z*: [M − H]^+^ calcd. for C_26_H_28_F_3_O, 413.2092; found, 413.2091.

*2,6-Di-tert-butyl-4-(2,2,2-trifluoro-1-(pyridin-2-yl)ethyl)phenol* (**3k**): 43 mg, 30% yield. Yellow oil. Purification by column chromatography (hexane/dichloromethane = 5:1, *v*/*v*). **^1^H NMR** (400 MHz, CDCl3) *δ* 8.62 (ddd, *J* = 4.9, 1.9, 0.9 Hz, 1H), 7.69 (td, *J* = 7.8, 1.9 Hz, 1H), 7.45 (d, *J* = 7.9 Hz, 1H), 7.26–7.19 (m, 3H), 5.23 (s, 1H), 4.79 (q, *J* = 9.9 Hz, 1H), 1.41 (s, 18H). **^13^C NMR** (101 MHz, CDCl_3_) *δ* 155.9 (d, *J* = 1.9 Hz), 153.7, 149.6, 136.8, 135.9, 126.2,126.0 (q, *J* = 280.2 Hz), 123.3, 122.6, 57.8 (q, *J* = 27.1 Hz), 34.4, 30.2. **^19^F NMR** (376 MHz, CDCl_3_) −66.14 (d, *J* = 8.7 Hz, 3F). **HRMS (ESI)** *m*/*z*: [M + H]+ calcd. for C_21_H_26_F_3_NO, 366.2045; found, 366.2049.

*2-Tert-butyl-4-(2,2-difluoro-1-phenylethyl)-6-methylphenol* (**4a**): 55 mg, 45% yield. Orange solid. M.p.: 104–105 °C. Purification by column chromatography (hexane/dichloromethane = 4:1, *v*/*v*). **^1^H NMR** (400 MHz, CDCl_3_): *δ* 7.33–7.11 (m, 5H), 6.99 (s, 1H), 6.85 (s, 1H), 6.17 (td, *J* = 56.1, 4.4 Hz, 1H), 4.67 (s, 1H), 4.21 (td, *J* = 16.1, 4.1 Hz, 1H), 2.12 (s, 3H), 1.30 (s, 9H). **^13^C{^1^H} NMR** (101 MHz, CDCl_3_): *δ* 152.0, 137.6 (t, *J* = 3.4 Hz), 135.8, 129.0, 128.9, 128.6, 128.3 (t, *J* = 3.7 Hz), 127.3, 126.0, 123.2, 117.2 (t, *J* = 244.3 Hz), 54.7 (t, *J* = 20.6 Hz), 34.6, 29.7, 16.1. **^19^F NMR** (376 MHz, CDCl_3_): *δ* (−116.71)–117.77 (m, 1F), (−117.78)–(−118.82) (m, 1F). **HRMS (ESI)** *m*/*z*: [M − H]^+^ calcd. for C_19_H_21_F_2_O, 303.1560; found, 303.1566.

*2-(Tert-butyl)-6-methyl-4-(2,2,2-trifluoro-1-phenylethyl)phenol* (**4b**) [50]: 61 mg, 47% yield. Yellow oil. Purification by column chromatography (hexane/dichloromethane = 4:1, *v*/*v*). **^1^H NMR** (400 MHz, CDCl_3_): *δ* 7.41–7.22 (m, 5H), 7.12 (s, 1H), 7.00 (s, 1H), 4.79 (s, 1H), 4.56 (q, *J* = 10.1 Hz, 1H), 2.21 (s, 3H), 1.38 (s, 9H). **^13^C{^1^H} NMR** (101 MHz, CDCl_3_): *δ* 152.3, 135.9, 135.8, 128.9, 128.9, 128.6 (q, *J* = 280.5 Hz), 127.8, 127.7, 126.5, 126.2, 123.2, 55.2 (q, *J* = 27.3 Hz), 34.6, 29.6, 16.1. **^19^F NMR** (376 MHz, CDCl_3_): *δ* −65.98 (d, *J* = 10.1 Hz, 3F). **HRMS (ESI)** *m*/*z*: [M − H]^+^ calcd. for C_19_H_20_F_3_O, 321.1466; found, 321.1474.

*2,6-Di-tert-butyl-4-(9-(difluoromethyl)-9H-fluoren-9-yl)phenol* (**4c**): 50 mg, 30% yield. Yellow oil. Purification by column chromatography (hexane/dichloromethane = 20:1, *v*/*v*). **^1^H NMR** (400 MHz, CDCl_3_) *δ* 7.77 (d, *J* = 7.6 Hz, 2H), 7.58 (d, *J* = 7.6 Hz, 2H), 7.43 (t, *J* = 7.6 Hz, 2H), 7.33 (t, *J* = 7.5 Hz, 2H), 7.22 (s, 2H), 6.08 (t, *J* = 56.0 Hz, 1H), 5.13 (s, 1H), 1.35 (s, 18H). **^13^C NMR** (101 MHz, CDCl_3_) *δ* 153.1, 144.91 (t, *J* = 3.5 Hz), 141.3, 135.6, 128.5, 127.6, 126.7, 124.5, 120.2, 117.8 (t, *J* = 233.1 Hz), 62.4 (t, *J* = 19.8 Hz), 34.5, 30.2. **^19^F NMR** (377 MHz, CDCl_3_) −119.21 (d, *J* = 56.2 Hz, 2F),. **HRMS (ESI)** *m*/*z*: [M − H]^+^ calcd. for C_28_H_30_F_2_O, 419.2186; found, 419.2191.

*2-(Tert-butyl)-6-methyl-4-(9-(trifluoromethyl)-9H-fluoren-9-yl)phenol* (**4d**): 109 mg, 62% yield. Yellow solid. M.p.: 156–158 °C. Purification by column chromatography (hexane/dichloromethane = 5:1, *v*/*v*). **^1^H NMR** (400 MHz, CDCl_3_): *δ* 7.66 (d, *J* = 7.4 Hz, 2H), 7.53 (d, *J* = 7.1 Hz, 2H), 7.33 (t, *J* = 7.4 Hz, 2H), 7.28–7.08 (m, 4H), 5.07 (s, 1H), 1.25 (s, 20H). **^13^C{^1^H} NMR** (101 MHz, CDCl_3_): *δ* 152.2, 142.9, 140.2, 134.5, 127.8, 126.6, 126.3, 125.3, 124.7 (q, *J* = 282.4 Hz), 123.3, 119.2, 62.5 (q, *J* = 26.5, 26.1 Hz), 33.4, 29.1. ^19^F NMR (376 MHz, CDCl_3_): *δ* −66.72 (s, 3F). **HRMS (ESI)** *m*/*z*: [M − H]^+^ calcd. for C_28_H_28_F_3_O, 437.2092; found, 437.2092.

*2,6-Di-tert-butyl-4-(2,2,3,3,3-pentafluoro-1-phenylpropyl)phenol* (**5a**): 146 mg, 88% yield. Yellow solid. M.p.: 70–72 °C. Purification by column chromatography (hexane/dichloromethane = 4:1, *v*/*v*). **^1^H NMR** (400 MHz, CDCl_3_) *δ* 7.46–7.45 (m, 2H), 7.39–7.26 (m, 3H), 7.21 (s, 2H), 5.19 (s, 1H), 4.45 (t, *J* = 18.0 Hz, 1H), 1.41 (s, 18H). **^13^C{^1^H} NMR** (101 MHz, CDCl3) *δ* 153.5, 135.9, 135.8, 129.3, 128.7, 127.8, 126.0, 125.6 (d, *J* = 3.3 Hz), 121.1–112.9 (m, CF_2_CF_3_), 53.3 (t, *J* = 20.7 Hz), 34.4, 30.2. **^19^F NMR** (376 MHz, CDCl_3_): *δ* −81.12 (s, 3F), −114.93 (qd, *J* = 270.8, 18.3 Hz, 2F). **HRMS (ESI)** *m*/*z*: [M − H]^+^ calcd. for C_27_H_35_F_5_O, 413.1904; found, 413.1913.

*Ethyl 3-(4-(tert-butyl)phenyl)-3-(3,5-di-tert-butyl-4-hydroxyphenyl)-2,2-difluoropropanoate* (**5b**) [47]: 112 mg, 67% yield. Brown oil. Purification by column chromatography (hexane/dichloromethane = 4:1, *v*/*v*). **^1^H NMR** (400 MHz, CDCl_3_): *δ* 7.43–7.41 (m, 2H), 7.34–7.25 (m, 3H), 7.18 (s, 2H), 5.17 (s, 1H), 4.64 (t, *J* = 18.5 Hz, 1H), 4.11 (qt, *J* = 7.1, 3.6 Hz, 2H), 1.40 (s, 18H), 1.02 (t, *J* = 7.1 Hz, 3H). **^13^C{^1^H} NMR** (101 MHz, CDCl_3_): *δ* 164.1 (t, *J* = 32.4 Hz), 153.4, 136.1 (d, *J* = 3.8 Hz), 135.8, 129.6, 128.5, 127.6, 126.3, 125.9 (d, *J* = 4.1 Hz), 116.1 (t, *J* = 255.7 Hz), 62.6, 55.5 (t, *J* = 21.8 Hz), 34.3, 30.3, 13.6. **^19^F NMR** (376 MHz, CDCl_3_): *δ* (−105.34)–(−107.42) (m, 2F). **HRMS (ESI)** *m*/*z*: [M − H]^+^ calcd. for C_29_H_40_F_2_O_3_, 417.2241; found, 417.2243.

*2,6-Di-tert-butyl-4-(2,2-difluoro-1-phenyl-2-(phenylthio)ethyl)phenol* (**5c**): 127 mg, 70% yield. Yellow solid. M.p.: 99–111 °C. Purification by column chromatography (hexane/dichloromethane = 5:1, *v*/*v*). **^1^H NMR** (400 MHz, CDCl_3_): *δ* 7.53–7.51 (m, 2H), 7.46–7.45 (m, 2H), 7.36–7.25 (m, 6H), 7.22 (s, 2H), 5.15 (s, 1H), 4.55 (t, *J* = 15.3 Hz, 1H), 1.41 (s, 18H). **^13^C{^1^H} NMR** (101 MHz, CDCl_3_): *δ* 153.2, 137.6 (d, *J* = 2.2 Hz), 136.2, 135.6, 130.2 (t, *J* = 284.6 Hz), 129.7, 129.5, 128.9, 128.4, 127.5, 127.4, 127.3, 126.4, 59.9 (t, *J* = 22.1 Hz), 34.4, 30.3. **^19^F NMR** (376 MHz, CDCl_3_): *δ* −72.36 (dd, *J* = 204.7, 15.0 Hz, 1F), −73.06 (dd, *J* = 204.8, 15.7 Hz, 1F). **HRMS (ESI)** *m*/*z*: [M − H]^+^ calcd. for C_28_H_31_OSF_2_, 452.2064; found, 453.2065.

*2,6-Di-tert-butyl-4-((2,2,3,3,3-pentafluoro-1-(furan-2-yl)propy)phenol* (**5d**): 118 mg, 73% yield. Yellow solid. M.p.: 112–114 °C. Purification by column chromatography (hexane/dichloromethane = 5:1, *v*/*v*). **^1^H NMR** (400 MHz, CDCl_3_): *δ* 7.39 (d, 1H), 7.24 (s, 2H), 6.39–6.33 (m, 2H), 5.24 (s, 1H), 4.61 (dd, *J* = 19.1, 14.8 Hz, 1H), 1.43 (s, 18H). **^13^C{^1^H} NMR** (101 MHz, CDCl_3_): *δ* 153.9, 148.4 (d, *J* = 6.1 Hz), 142.6, 135.9, 126.4, 122.8 (d, *J* = 2.8 Hz), 120.9–111.1 (m, CF_2_CF_3_), 110.5, 109.3, 47.2 (dd, *J* = 23.6, 20.9 Hz), 34.3, 30.2. **^19^F NMR** (376 MHz, CDCl_3_): *δ* −81.93 (s, 3F), −115.62 (dd, *J* = 269.2, 14.6 Hz, 1F), −117.41 (dd, *J* = 269.3, 19.3 Hz, 1F). **HRMS (ESI)** *m*/*z*: [M − H]^+^ calcd. for C_21_H_24_O_2_F_5_, 403.1696; found, 403.1699.

*Ethyl 3-(3,5-di-tert-butyl-4-hydroxyphenyl)-2,2-difluoro-3-(furan-2-yl)propanoate* (**5e**): 98 mg, 60% yield. Brown oil. Purification by column chromatography (hexane/dichloromethane = 5:1, *v*/*v*). **^1^H NMR** (400 MHz, CDCl_3_): *δ* 7.38 (dd, *J* = 1.8, 0.9 Hz, 1H), 7.19 (s, 2H), 6.37–6.33 (m, 2H), 5.22 (s, 1H), 4.75 (t, *J* = 16.8 Hz, 1H), 4.17 (qd, *J* = 7.2, 5.5 Hz, 2H), 1.42 (s, 18H), 1.15 (t, *J* = 7.1 Hz, 3H). **^13^C{^1^H} NMR** (101 MHz, CDCl_3_): *δ* 163.79 (t, *J* = 32.3 Hz), 153.81, 149.43 (d, *J* = 6.2 Hz), 142.4, 135.8, 126.6, 123.2 (d, *J* = 2.6 Hz), 114.9 (t, *J* = 256.5 Hz), 110.4, 109.1, 62.7, 49.7 (t, *J* = 23.3 Hz), 34.3, 30.2, 13.7. **^19^F NMR** (376 MHz, CDCl_3_): *δ* −108.07 (dd, *J* = 253.3, 15.9 Hz, 1F), −109.06 (dd, *J* = 253.5, 17.8 Hz, 1F). **HRMS (ESI)** *m*/*z*: [M − H]^+^ calcd. for C_23_H_29_F_2_O_4_, 407.2034; found, 407.2040.

#### 3.2.2. Experimental Procedures for the Synthesis of 2,6-Di-*tert*-butyl-4-(1-(4-chlorophenyl)-2,2-difluoroethylidene)cyclohexa-2,5-dien-1-one (**6a**)

K_3_[Fe(CN)_6_] (395 mg, 1.2 mmol) and KOH (71 mg, 1.26 mmol) in water (3 mL) were added in one portion to a solution of **2f** (114 mg, 0.3 mmol) in hexane (3 mL) under N_2_ in a 25-milliliter round-bottom flask equipped with a magnetic stir bar. The reaction mixture was stirred at room temperature for 5 h. The organic layer was separated and the aqueous layer was extracted with hexane. The combined organic layer was washed with brine and dried over Na_2_SO_4_. After filtration, the solution was concentrated by rotary evaporation. The residue was purified by silica gel flash column chromatography using petroleum ether to afford **6a** (88.5 mg, 78%).

*2,6-Di-tert-butyl-4-(1-(4-chlorophenyl)-2,2-difluoroethylidene)cyclohexa-2,5-dien-1-one* (**6a**): 118 mg, 78% yield. Yellow solid. M.p.: 125–127 °C. Purification by column chromatography using hexane. **^1^H NMR** (400 MHz, CDCl_3_): *δ* 7.48–7.42 (m, 2H), 7.41–7.37 (m, 1H), 7.25 (d, *J* = 9.0 Hz, 2H), 6.98 (t, *J* = 54.9 Hz, 1H), 6.81 (d, *J* = 2.5 Hz, 1H), 1.34 (s, 9H), 1.15 (s, 9H). **^13^C{^1^H} NMR** (101 MHz, CDCl_3_): *δ* 186.1, 150.5, 150.4, 139.9 (t, *J* = 20.6 Hz), 135.4, 133.5 (t, *J* = 7.5 Hz), 131.7, 131.7, 129.8, 128.6, 125.3, 111.7 (t, *J* = 237.9 Hz), 35.8, 35.5, 29.5, 29.3. **^19^F NMR** (376 MHz, CDCl_3_): *δ* −109.91 (d, *J* = 54.7 Hz, 2F). **HRMS (ESI)** *m*/*z*: [M − H]^+^ calcd. for C_22_H_24_ClF_2_O, 377.1484; found, 377.1482.

#### 3.2.3. Experimental Procedures for the Synthesis of 4-(1-(4-Chlorophenyl)-2,2-difluoroethyl) Phenol (**6b**)

A 10-milliliter sealed tube equipped with a magnetic stir bar was charged with **2f** (114 mg, 0.3 mmol) and dry toluene (3 mL). The solution was added with concentrated H_2_SO_4_ (1 drop) and heated at 120 °C (oil bath temperature) for 18 h with vigorous stirring. After cooling to room temperature, water (20 mL) was poured into the reaction mixture, and then the mixture was extracted with dichloromethane (3 × 20 mL). The combined organic layer was dried over Na_2_SO_4_, filtered, and evaporated under reduced pressure. The residue was purified by flash column chromatography on silica gel using petroleum ether-ethyl acetate (5:1-1:1, *v*/*v*) as an eluent to afford the product **6b** (65.0 mg, 81%).

*4-(1-(4-Chlorophenyl)-2,2-difluoroethyl)phenol* (**6b**): 87 mg, 81% yield. Yellow oil. Purification by column chromatography (hexane/dichloromethane = 5:1, *v*/*v*). **^1^H NMR** (400 MHz, CDCl_3_): *δ* 7.34–7.27 (m, 2H), 7.21 (d, *J* = 8.5 Hz, 2H), 7.17–7.07 (m, 2H), 6.87–6.71 (m, 2H), 6.22 (td, *J* = 55.8, 4.1 Hz, 1H), 5.14 (s, 1H), 4.32 (td, *J* = 16.1, 4.1 Hz, 1H). **^13^C{^1^H} NMR** (101 MHz, CDCl_3_): *δ* 155.1, 135.7 (t, *J* = 3.3 Hz), 133.4, 130.4, 130.2, 128.8, 128.7, 116.6 (t, *J* = 244.7 Hz), 115.7, 53.5 (t, *J* = 20.8 Hz). **^19^F NMR** (376 MHz, CDCl_3_): *δ* −117.75 (ddd, *J* = 279.3, 55.8, 15.6 Hz, 1F), −118.89 (ddd, *J* = 280.0, 56.8, 17.0 Hz, 1F). **HRMS (ESI)** *m*/*z*: [M − H]^+^ calcd. for C_14_H_10_ClF_2_O, 267.0388; found, 267.0390.

#### 3.2.4. General Experimental Procedure for the Synthesis of 1-Ethoxy-4-(2,2,2-trifluoro-1-(4-methoxyphenyl)ethyl)benzene (**7b**)

A 30-milliliter sealed tube equipped with a magnetic stir bar was charged with **3i** (236 mg, 0.6 mmol) and dry toluene (5 mL). The solution was added with concentrated H_2_SO_4_ (2 drops) and heated at 120 °C (oil bath temperature) for 18 h with vigorous stirring. After cooling to room temperature, water (20 mL) was poured into the reaction mixture, and then the mixture was extracted with dichloromethane (3 × 20 mL). The combined organic layer was dried over Na_2_SO_4_, filtered, and evaporated under reduced pressure. The residue was purified by flash column chromatography on silica gel using petroleum ether-ethyl acetate (5:1–1:1, *v*/*v*) as an eluent to afford the intermediate **7a** (127.0 mg, 75%).

A 25-milliliter round-bottom flask was charged with a magnetic stir bar, the intermediate **7a** (84.5 mg, 0.3 mmol), Cs_2_CO_3_ (71 mg, 0.6 mmol), CH_3_CN (10 mL), and iodoethane (93.5 mg, 0.6 mmol). The reaction mixture was stirred for about 24 h at 90 °C (oil bath temperature) and then cooled to room temperature and filtered. The solvent was evaporated under vacuum. The residue was subjected to silica gel column chromatography using petroleum ether–ethyl acetate (10:1, *v*/*v*) as an eluent to give the product **7b** (82.8 mg, 89% yield).

*4-(2,2,2-Trifluoro-1-(4-methoxyphenyl)ethyl)phenol* (**7a**): 85 mg, 75% yield. Yellow oil. Purification by column chromatography (hexane/dichloromethane = 5:1, *v*/*v*). **^1^H NMR** (400 MHz, CDCl_3_): *δ* 7.26 (d, *J* = 8.7 Hz, 2H), 7.21 (d, *J* = 8.5 Hz, 2H), 6.87 (d, *J* = 8.7 Hz, 2H), 6.82–6.73 (m, 2H), 5.21– 5.17 (m, 1H), 4.56 (q, *J* = 10.0 Hz, 1H), 3.79 (s, 1H). **^13^C{^1^H} NMR** (101 MHz, CDCl_3_): *δ* 159.1, 155.2, 130.4, 130.1, 128.0, 127.8, 126.4 (q, *J* = 280.3 Hz), 115.5, 114.1, 55.3, 53.9 (q, *J* = 27.6 Hz). **^19^F NMR** (376 MHz, CDCl_3_): *δ* −66.38 (s, 3F). **HRMS (ESI)** *m*/*z*: [M − H]^+^ calcd. for C_15_H_12_F_3_O_2_, 281.0789; found, 281.0793.

*1-Ethoxy-4-(2,2,2-trifluoro-1-(4-methoxyphenyl)ethyl)benzene* (**7b**): 110 mg, 89% yield. Yellow oil. Purification by column chromatography (hexane/dichloromethane = 10:1, *v*/*v*). **^1^H NMR** (400 MHz, CDCl_3_): *δ* 7.28–7.24 (m, 4H), 7.04–6.44 (m, 4H), 4.57 (q, *J* = 9.8 Hz, 1H), 4.00 (q, *J* = 7.0 Hz, 2H), 3.78 (s, 3H), 1.39 (t, *J* = 7.0 Hz, 3H). **^13^C{^1^H} NMR** (101 MHz, CDCl_3_): *δ* 159.1, 158.5, 130.1, 130.1, 129.2 (q, *J* = 280.4 Hz), 127.9 (d, *J* = 1.4 Hz), 127.6 (d, *J* = 1.3 Hz), 114.6, 114.1, 63.4, 55.2, 54.0 (q, *J* = 27.5 Hz), 14.8. **^19^F NMR** (376 MHz, CDCl_3_): *δ* −66.38 (s). **MS** (**EI**, *m*/*z*, %): 213 (46.74), 241 (100.00), 310 (M^+^, 36.84).

## 4. Conclusions

In summary, we have developed a direct method for the 1,6-nucleophilic difluoromethylation, trifluoromethylation, and difluoroalkylation of *p*-QMs using Me_3_SiRf (Rf = CF_2_H, CF_3_, CF_2_CF_3_, CF_2_COOEt, and CF_2_SPh) as a reagent, promoted by CsF/18-crown-6, within a temperature range of −15 °C to room temperature. The nucleophilic reaction is suitable for *p*-QMs with various substituents, giving the corresponding products in satisfactory to good yields. The synthetic utility of the approach has been exemplified by the formation of fluoroalkylated *p*-quinone methide (via oxidation) and *α*-fluoroalkyl diarylmethane (via de-*tert*-butylation).

## Figures and Tables

**Table 1:** Optimization of reaction conditions between *p*-QMs **1a** and Me_3_SiCF_2_H ^a^.

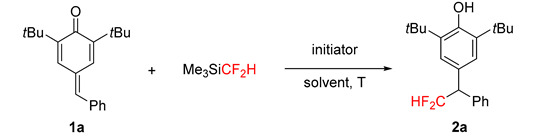				
Entry	Initiator (equiv)	T (°C)	Solvent	Yield (%) ^b^
1	CsF (0.2)/18-crown-6 (0.2)	rt	THF	0
2	TBAF (0.2)	−15 to rt	DMF	30
3	CsF (0.2)	−15 to rt	DMF	33
4	TBAF (0.2)	−30	DMF	17
5	TMAF (0.2)	−15 to rt	DMF	30
6	KF (0.2)	−30	DMF	trace
7	TBAF (0.2)	rt	DMF	20
8	CsF(1.0)	−30	DMF	36
9	CsF (1.0)/18-crown-6 (0.2)	−30	DMF	51
10	CsF (0.2)/18-crown-6 (0.1)	−30	DMF	42
11	CsF (0.2)/18-crown-6 (0.1)	−15 to rt	DMF	52
12	CsF (1.0)/18-crown-6 (1.0)	−15 to rt	DMF	60
13	CsF (1.5)/18-crown-6 (1.5)	−15 to rt	DMF	70
14	CsF (2.0)/18-crown-6 (2.0)	−15 to rt	DMF	60
15	KF (1.5)/18-crown-6 (1.5)	−15 to rt	DMF	12

^a^ In all entries, Me_3_SiCF_2_H (0.4 mmol, 2 equiv) and **1a** (0.2 mmol, 1.0 equiv) were used. ^b^ Yields were determined by ^19^F NMR analysis using PhCF_3_ as an internal standard.

**Table 2:** Direct nucleophilic difluoromethylation of *p*-QMs with Me_3_SiCF_2_H ^a,b^.

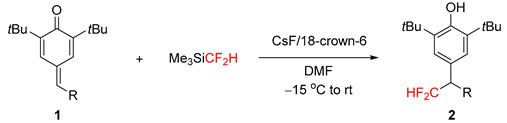				
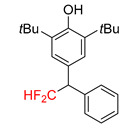	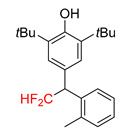	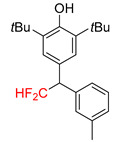	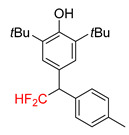	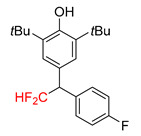
**2a**, 70%	**2b**, 70%	**2c**, 46%	**2d**, 49%	**2e**, 78%
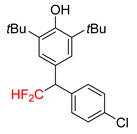	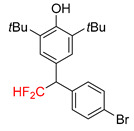	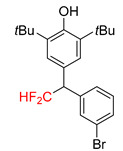	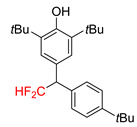	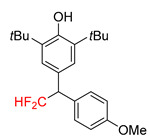
**2f**, 86%	**2g**, 63%	**2h**, 68%	**2i**, 60%	**2j**, 40%
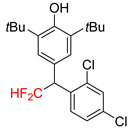	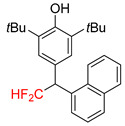	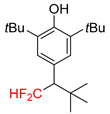	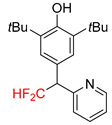	
**2k**, 67%	**2l**, 61%	**2m**, 23%	**2n**, 62%	

^a^ Me_3_SiCF_2_H (0.8 mmol, 2 equiv) and **1** (0.4 mmol, 1.0 equiv) were used. ^b^ Isolated yields were given.

**Table 3:** Direct nucleophilic trifluoromethylation of *p*-QMs with Me_3_SiCF_3_ ^a,b^.

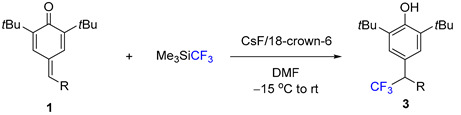				
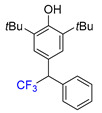	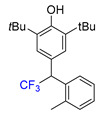	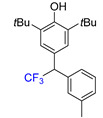	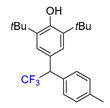	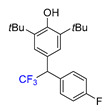
**3a**, 66%	**3b**, 85%	**3c**, 61%	**3d**, 82%	**3e**, 57%
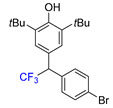	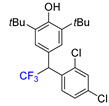	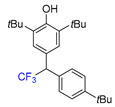	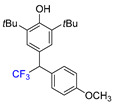	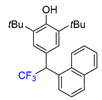
**3f**, 86%	**3g**, 61%	**3h**, 45%	**3i**, 55%	**3j**, 78%
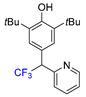				
**3k**, 30%				

^a^ Me_3_SiCF_3_ (0.8 mmol, 2.0 equiv) and **1** (0.4 mmol, 1.0 equiv) were used. ^b^ Isolated yields were given.

**Table 4:** Direct nucleophilic fluoroalkylation of *p*-QMs with other fluoroalkyltrimethylsilane reagents ^a,b^.

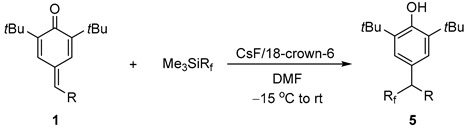				
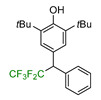	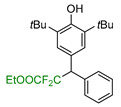	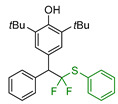	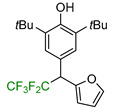	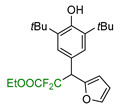
**5a**, 88%	**5b**, 67%	**5c**, 70%	**5d**, 73%	**5e**, 60%

^a^ Me_3_SiRf (0.8 mmol, 2.0 equiv) and **1** (0.4 mmol, 1.0 equiv) were used. ^b^ Isolated yields are given.

## Data Availability

Data are contained within the article and Supplementary Materials.

## Supplementary Materials

The following supporting information can be downloaded at: https://www.mdpi.com/article/10.3390/molecules29122905/s1. The NMR spectra of all products are included in.
