# Supplementary material for: 1,6-Nucleophilic Di- and Trifluoromethylation of para-Quinone Methides with Me3SiCF2H/Me3SiCF3 Facilitated by CsF/18-Crown-6
Source: Molecules. 2024 Jun 19;29(12):2905. doi: 10.3390/molecules29122905 (PMC11206660; doi:10.3390/molecules29122905)

# *Supporting Information*

*for*

# 1,6-Nucleophilic Di- and Trifluoromethylation of *para*-Quinone Methides with $Me_3SiCF_2H/Me_3SiCF_3$ Facilitated by CsF/18-Crown-6

**Dingben Chen [1,2], Ling Huang [1], Mingyu Liang [1], Xiaojing Chen [1], Dongdong Cao [1], Pan Xiao [2,3], Chuanfa Ni [2] and Jinbo Hu [2,3,*]**

[1] School of Pharmaceutical and Chemical Engineering, Taizhou University, Taizhou 318000, China
[2] Key Laboratory of Fluorine and Nitrogen Chemistry and Advanced Materials, Shanghai Institute of Organic Chemistry, University of Chinese Academy of Sciences, Chinese Academy of Sciences, 345 Lingling Road, Shanghai 200032, China
[3] School of Physical Science and Technology, ShanghaiTech University, 100 Haike Road, Shanghai 201210, China
[*] Correspondence: jinbohu@sioc.ac.cn

## Table of contents

I. NMR spectra of products S2

# I. NMR spectra of products

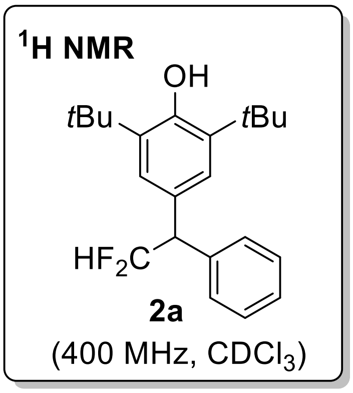


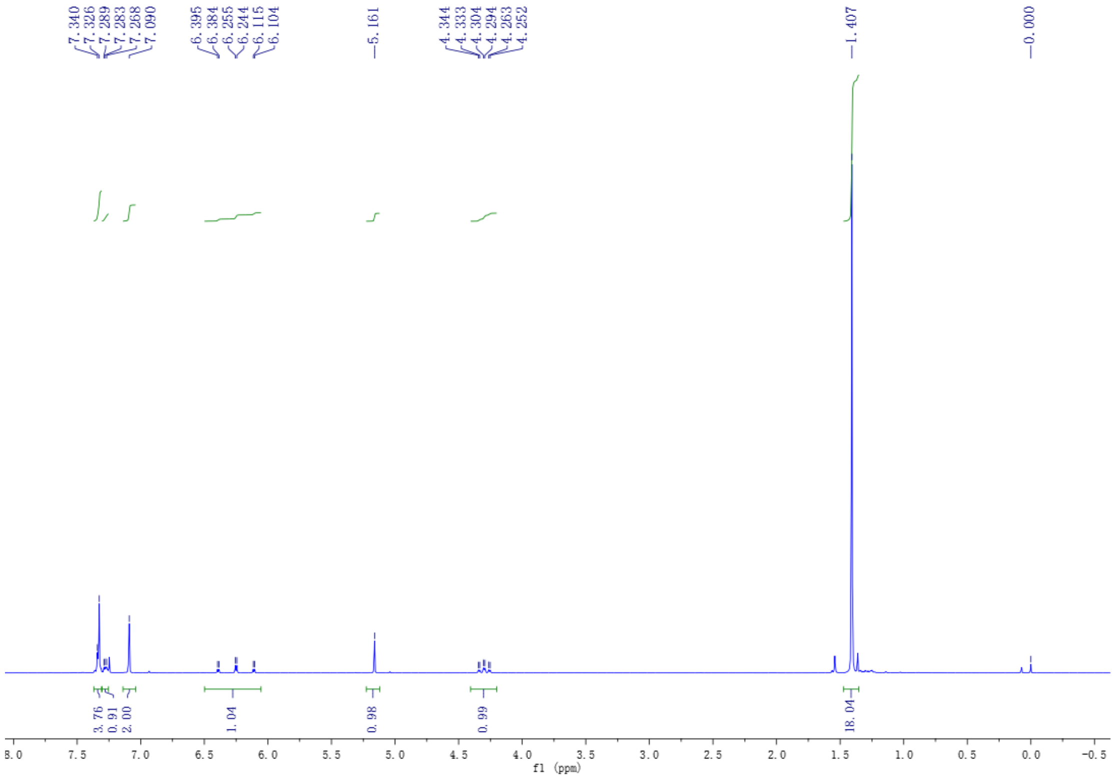

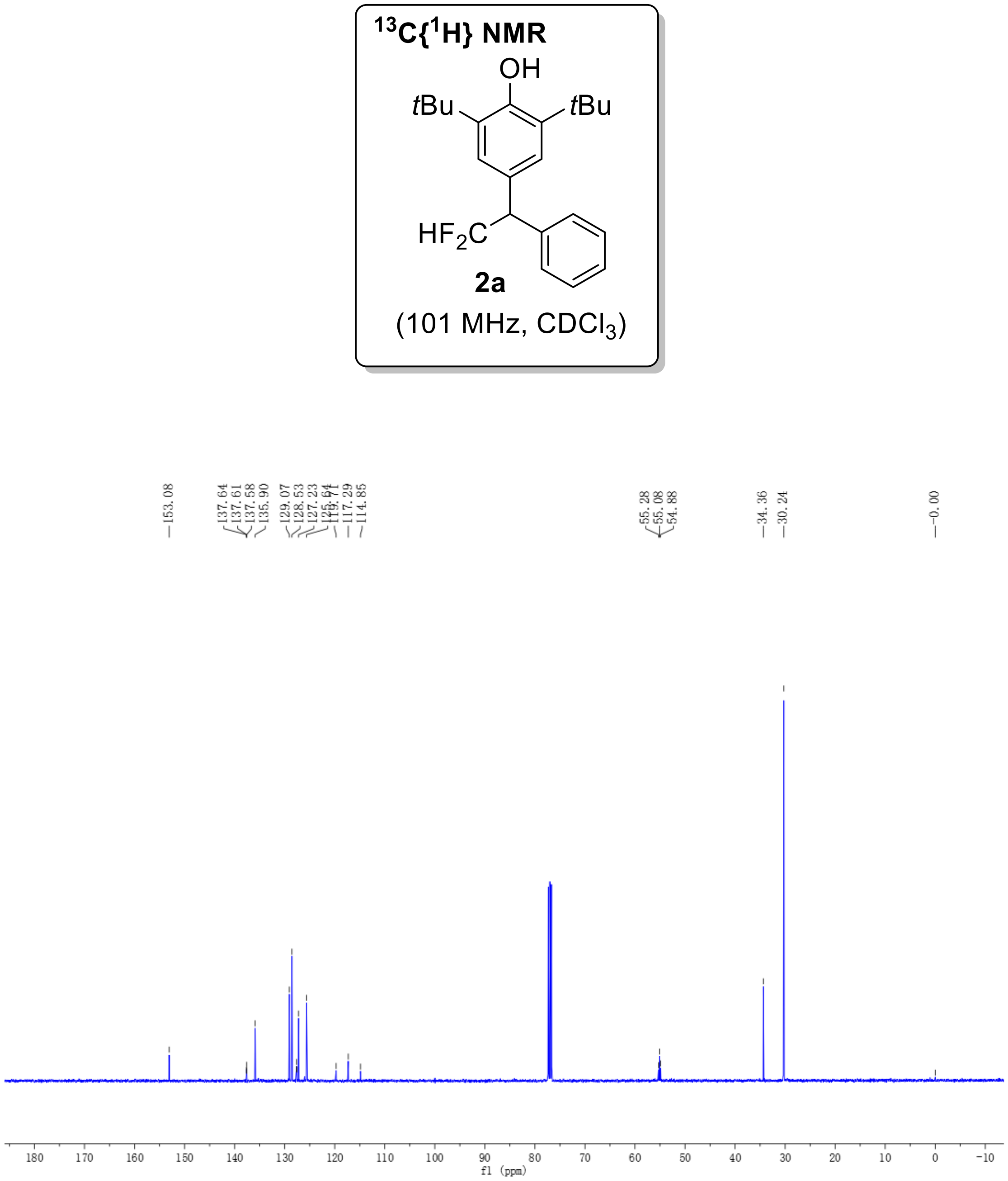

$^{13}C\{^{1}H\}$ NMR
OH
tBu
tBu
$HF_2C$
2a
(101 MHz, $CDCl_3$)
153.08
137.64
137.61
137.58
135.90
129.07
128.53
127.23
125.64
119.71
117.29
114.85
55.28
55.08
54.88
34.36
30.24
0.00
180
170
160
150
140
130
120
110
100
90
80
70
60
50
40
30
20
10
0
-10
f1 (ppm)

**$^{19}$F NMR**

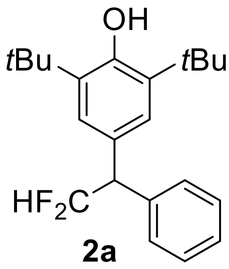


**2a**

(377 MHz, $CDCl_3$)

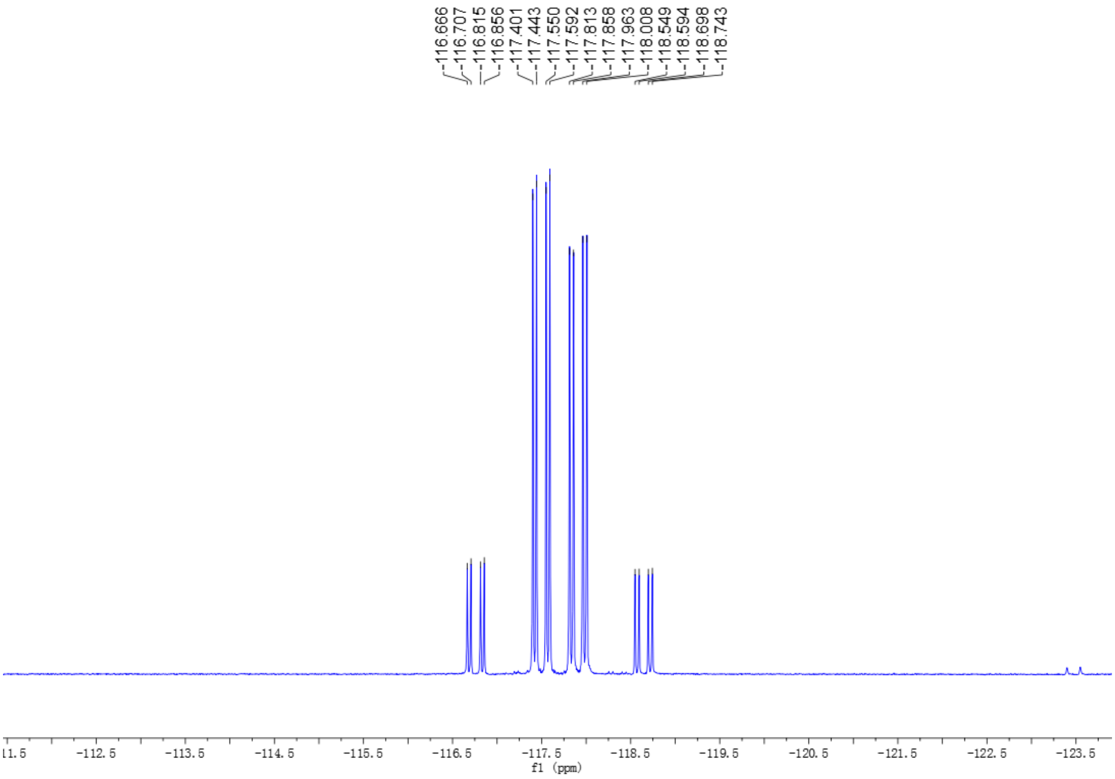

**¹H NMR**

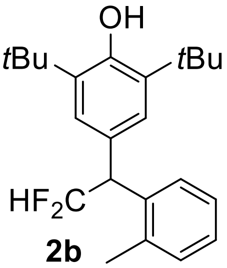


**2b**

(400 MHz, $CDCl_3$)

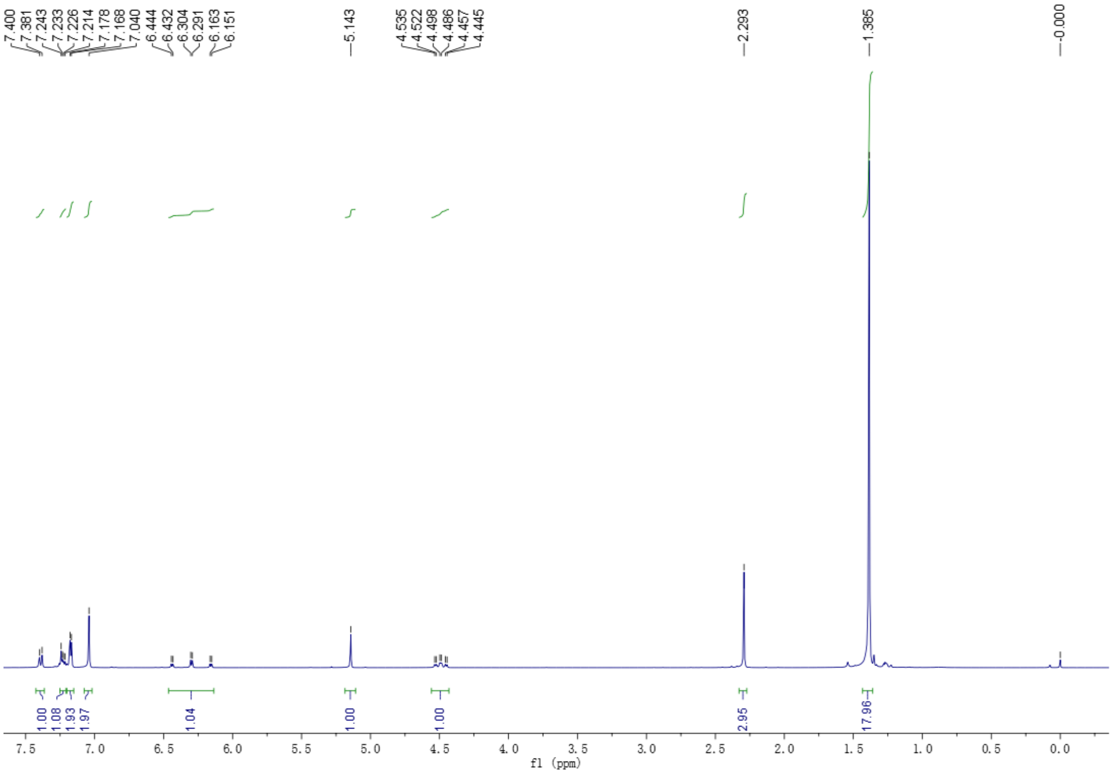

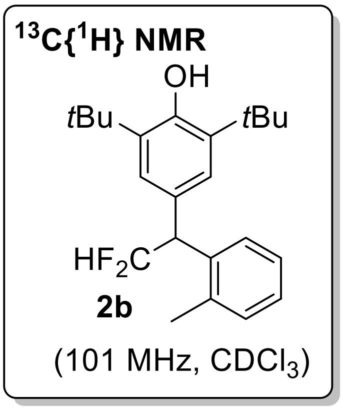

$^{13}C\{^1H\}$ NMR
OH
tBu
tBu
$HF_2C$
2b
(101 MHz, $CDCl_3$)

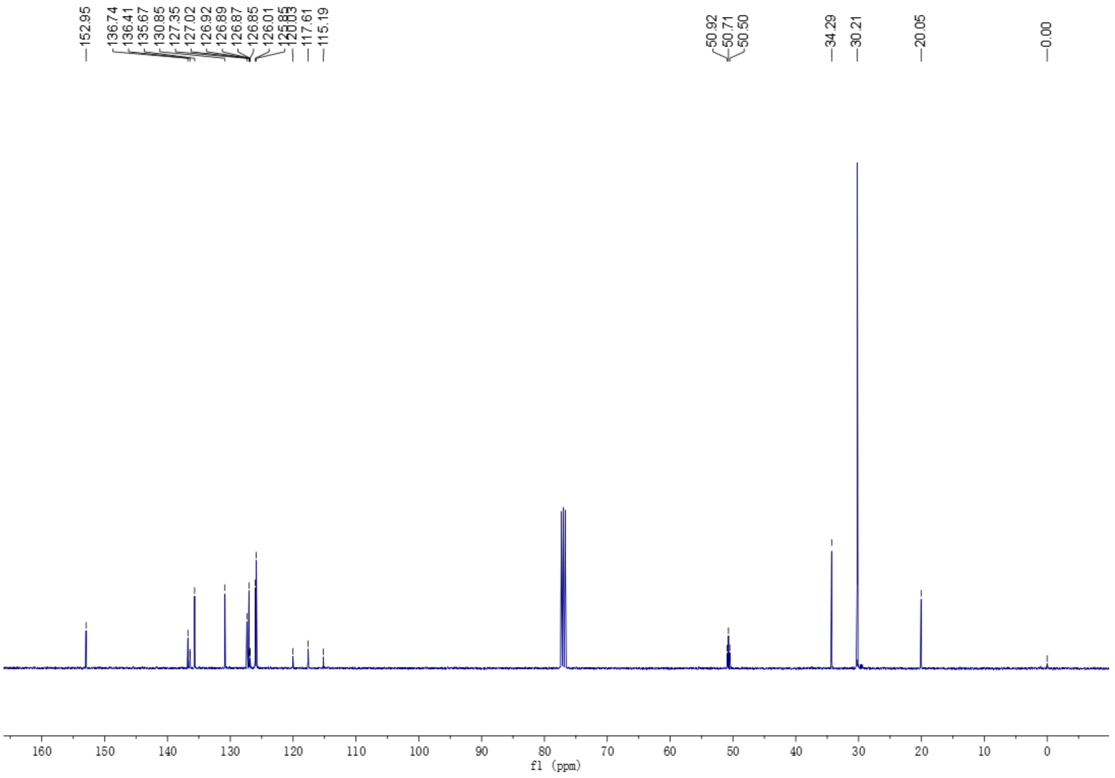


**$^{19}$F NMR**

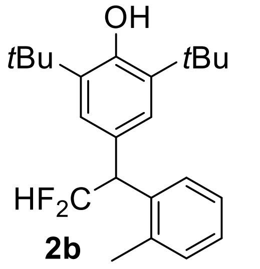


**2b**

(377 MHz, $CDCl_3$)

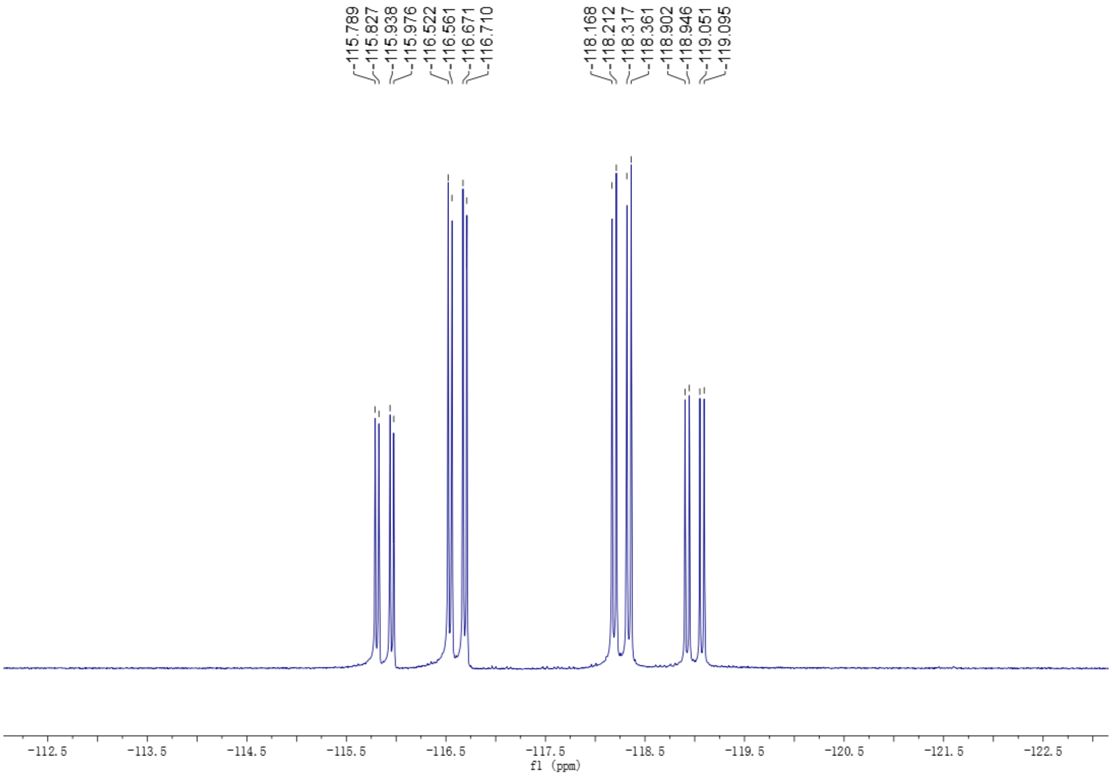
-115.789
-115.827
-115.938
-115.976
-116.522
-116.561
-116.671
-116.710
-118.168
-118.212
-118.317
-118.361
-118.902
-118.946
-119.051
-119.095
-112.5
-113.5
-114.5
-115.5
-116.5
-117.5
-118.5
-119.5
-120.5
-121.5
-122.5
f1 (ppm)

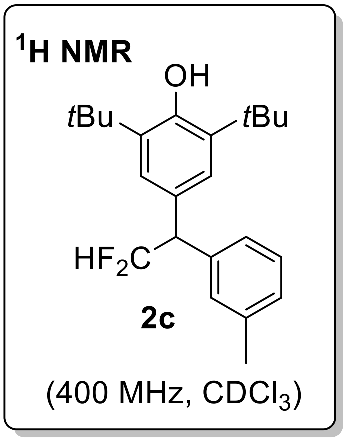
1H NMR
OH
tBu
tBu
HF2C
2c
(400 MHz, CDCl3)

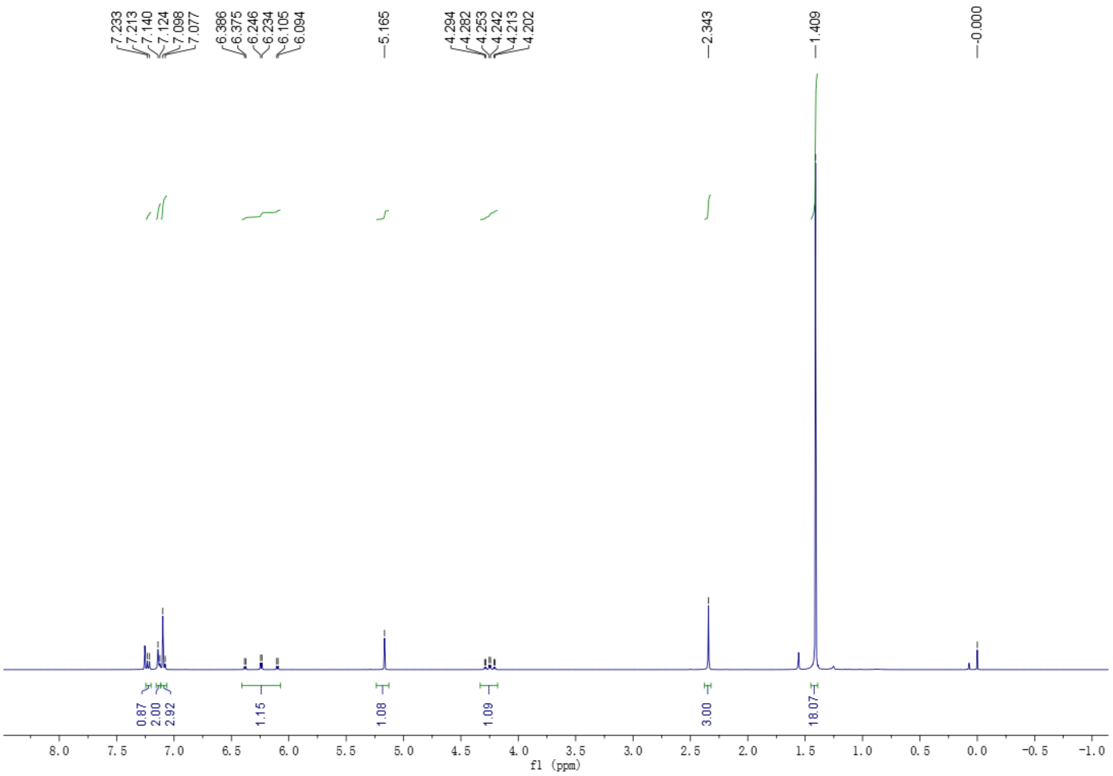
7.233
7.213
7.140
7.124
7.098
7.077
6.386
6.375
6.246
6.234
6.105
6.094
5.165
4.294
4.282
4.253
4.242
4.213
4.202
2.343
1.409
0.000
0.87
2.00
2.92
1.15
1.08
1.09
3.00
18.07
8.0
7.5
7.0
6.5
6.0
5.5
5.0
4.5
4.0
3.5
3.0
2.5
2.0
1.5
1.0
0.5
0.0
-0.5
-1.0
f1 (ppm)

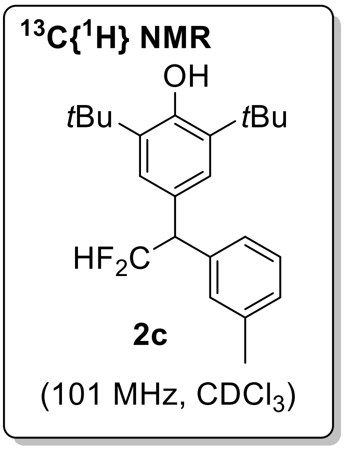
13C{1H} NMR
OH
tBu
tBu
HF2C
2c
(101 MHz, CDCl3)

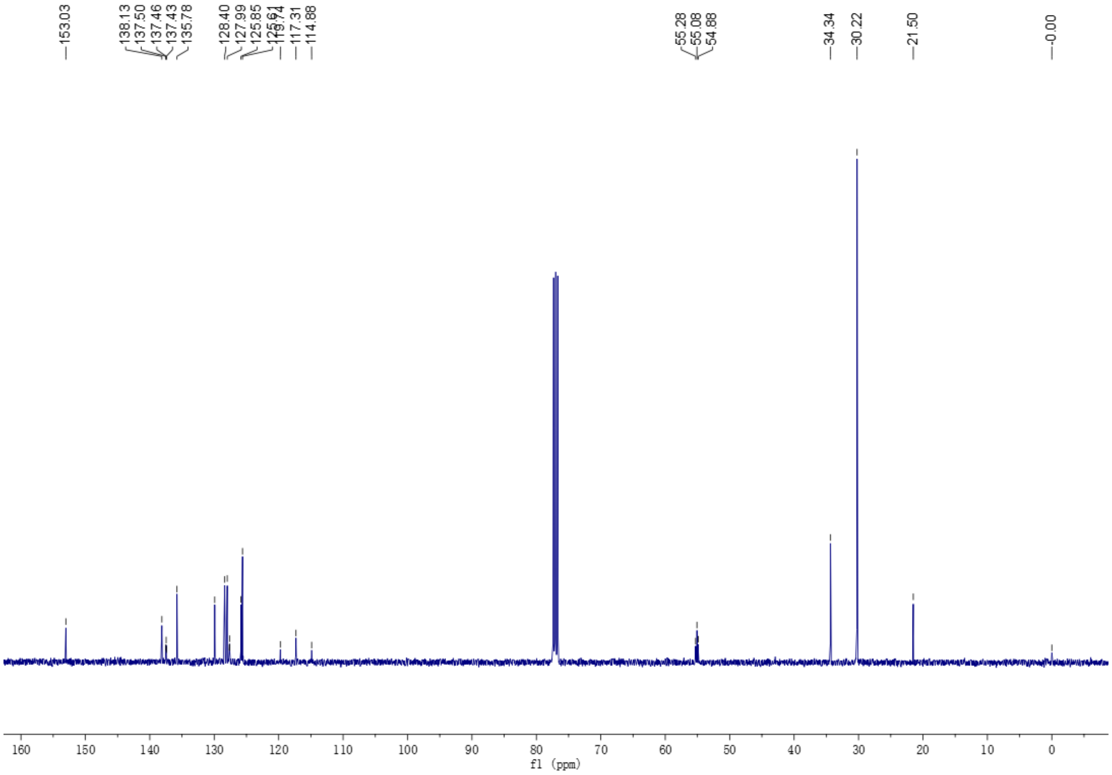
153.03
138.13
137.50
137.46
137.43
135.78
128.40
127.99
125.85
125.61
119.74
117.31
114.88
55.28
55.08
54.88
34.34
30.22
21.50
-0.00
f1 (ppm)

**$^{19}$F NMR**

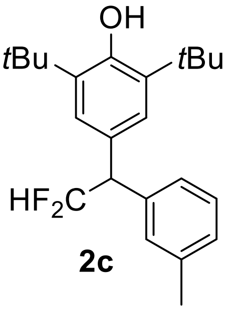


**2c**

(377 MHz, $CDCl_3$)

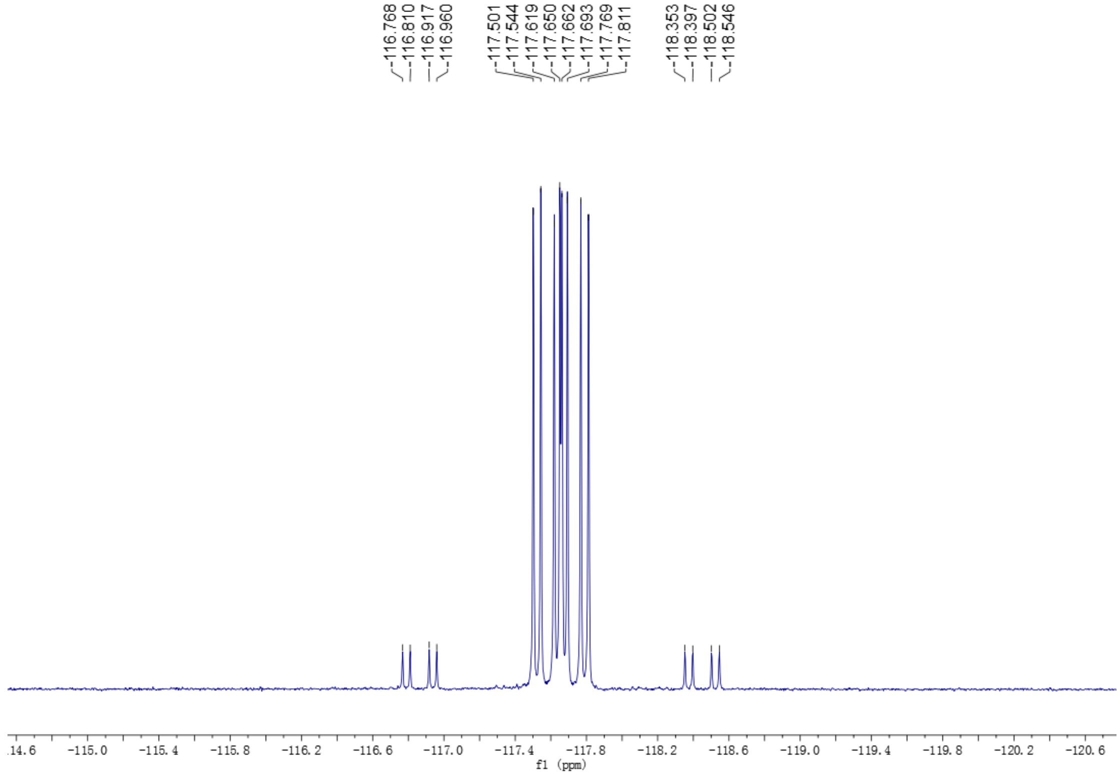

**$^1$H NMR**

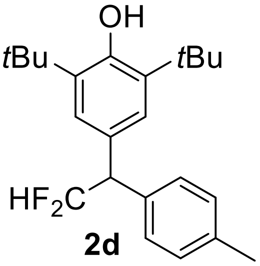


**2d**

(400 MHz, $CDCl_3$)

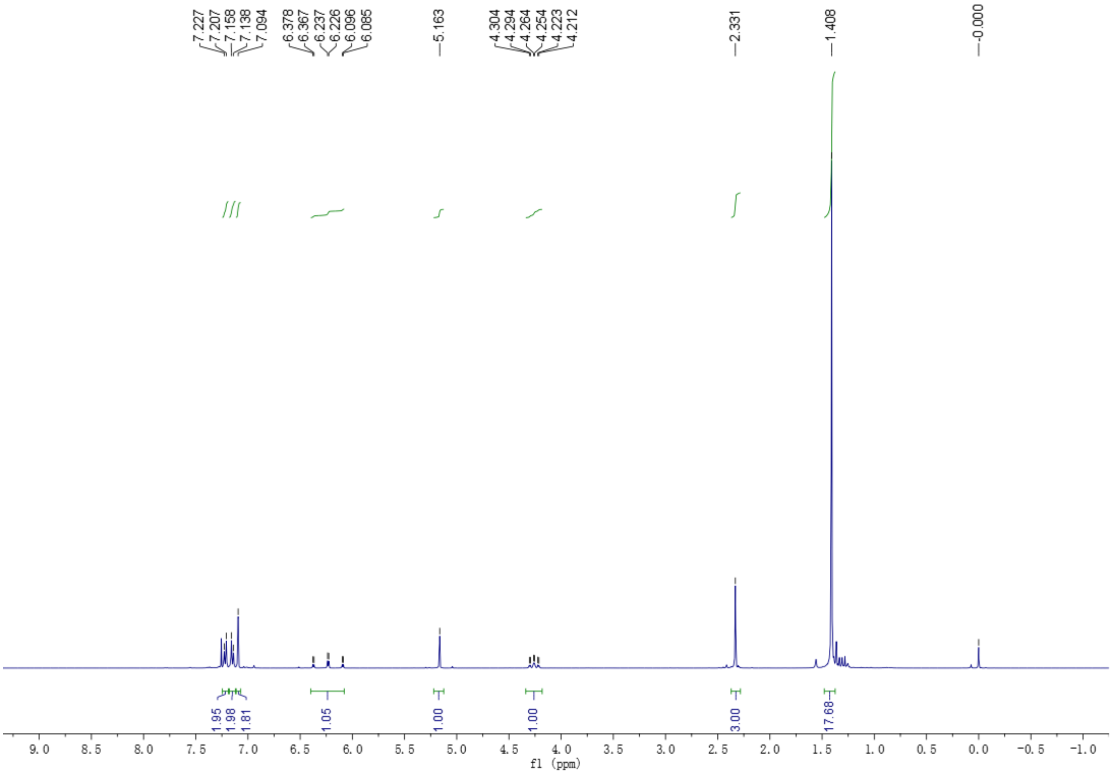

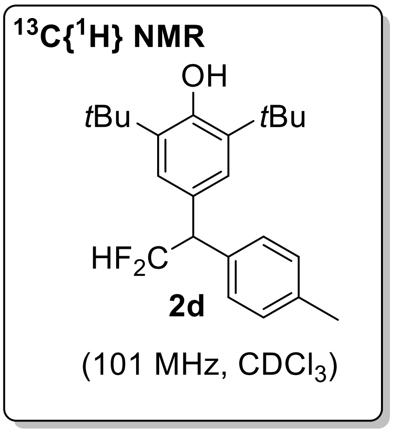

$^{13}C\{^1H\}$ NMR
OH
tBu
tBu
$HF_2C$
2d
(101 MHz, $CDCl_3$)


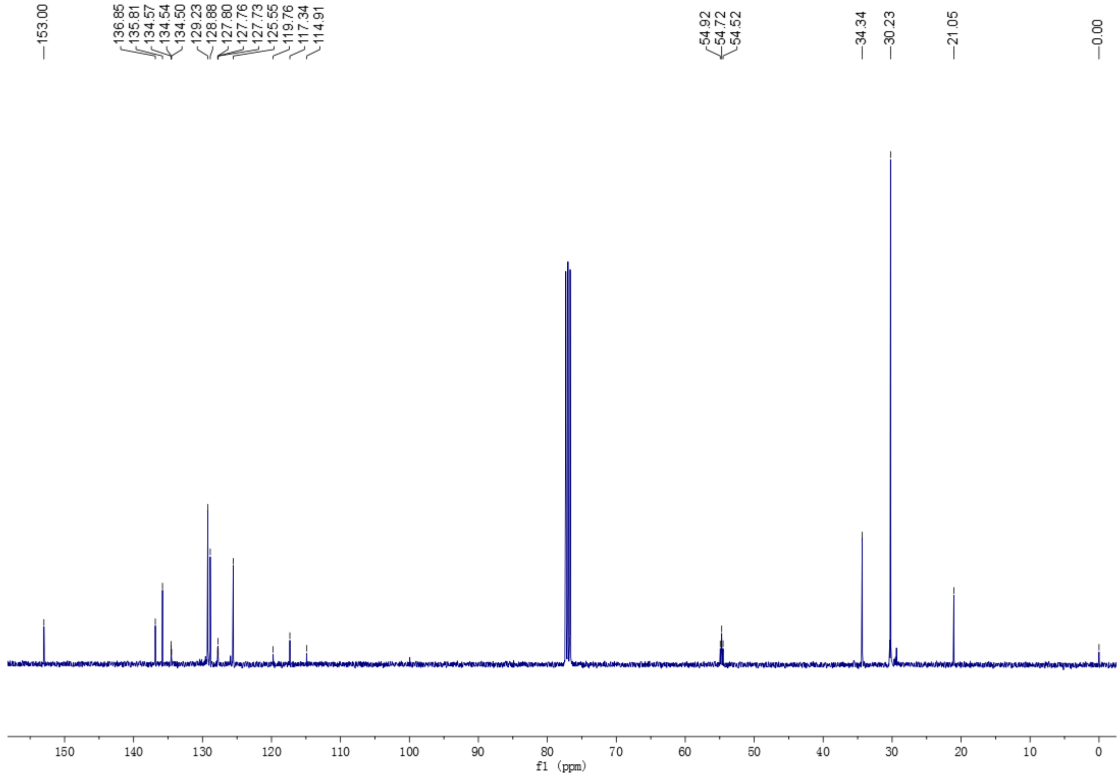

153.00
136.85
135.81
134.57
134.54
134.50
129.23
128.88
127.80
127.76
127.73
125.55
119.76
117.34
114.91
54.92
54.72
54.52
34.34
30.23
21.05
0.00
f1 (ppm)

**$^{19}F$ NMR**

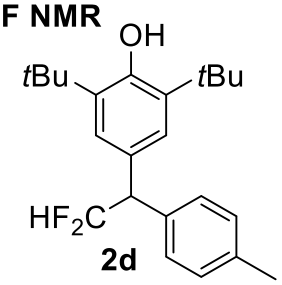


**2d**

(377 MHz, $CDCl_3$)

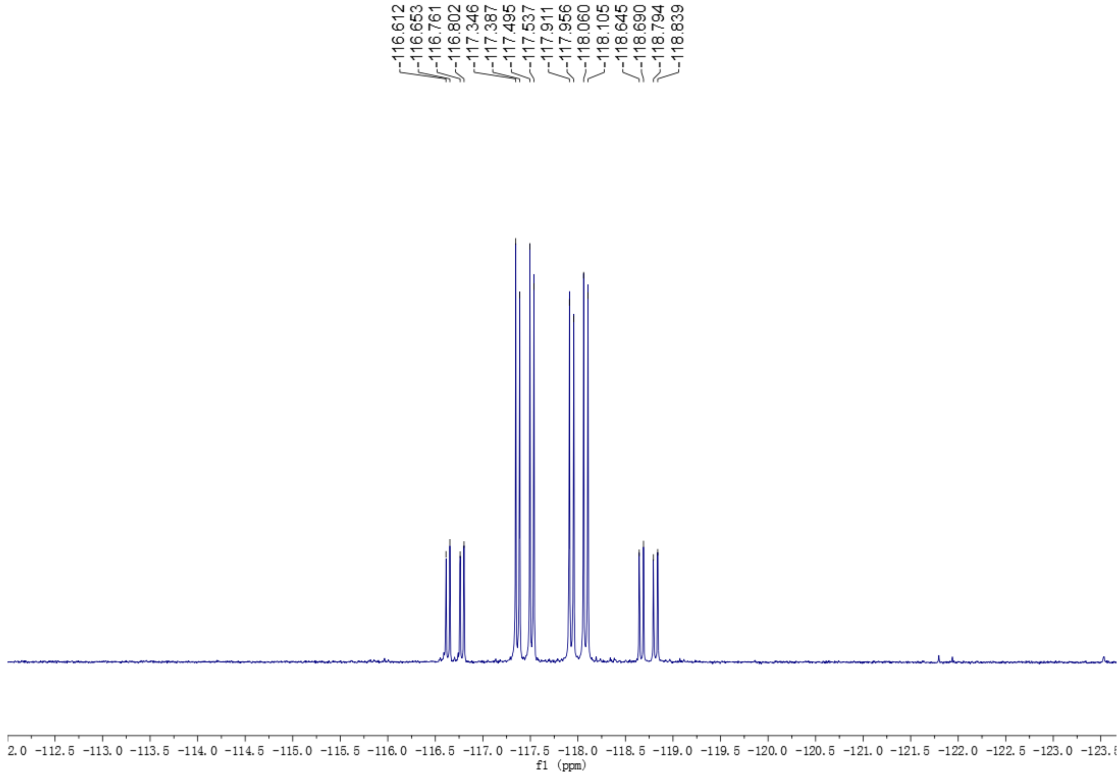

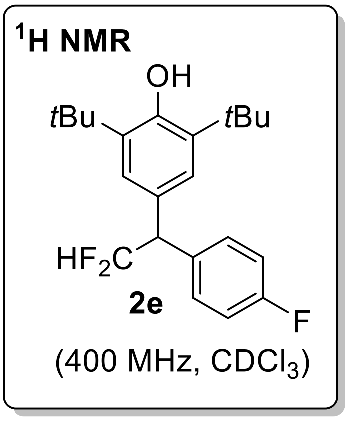

1H NMR
OH
tBu
tBu
HF2C
2e
F
(400 MHz, CDCl3)


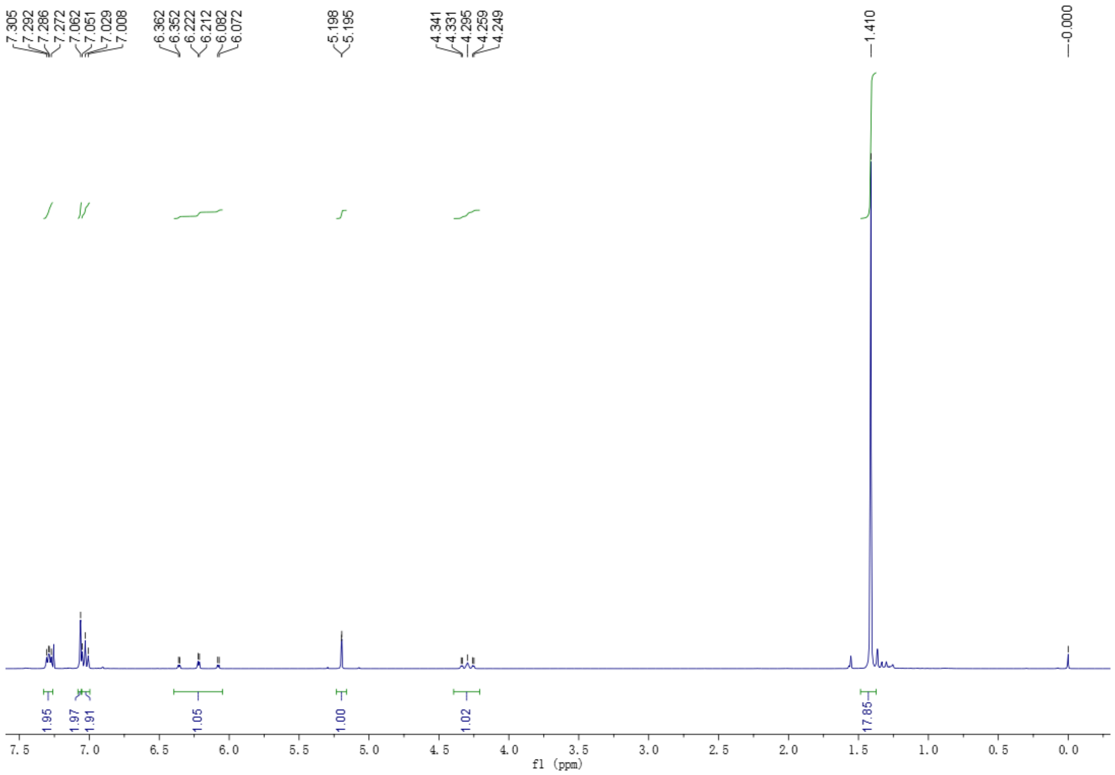

7.305
7.292
7.286
7.272
7.062
7.051
7.029
7.008
6.362
6.352
6.222
6.212
6.082
6.072
5.198
5.195
4.341
4.331
4.295
4.259
4.249
1.410
-0.000
1.95
1.97
1.91
1.05
1.00
1.02
17.85
7.5
7.0
6.5
6.0
5.5
5.0
4.5
4.0
3.5
3.0
2.5
2.0
1.5
1.0
0.5
0.0
f1 (ppm)

$^{13}C\{^{1}H\}$ **NMR**

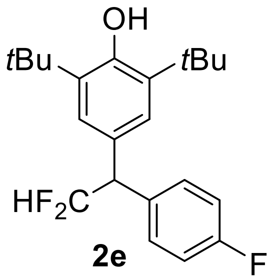


**2e**

(101 MHz, $CDCl_3$)

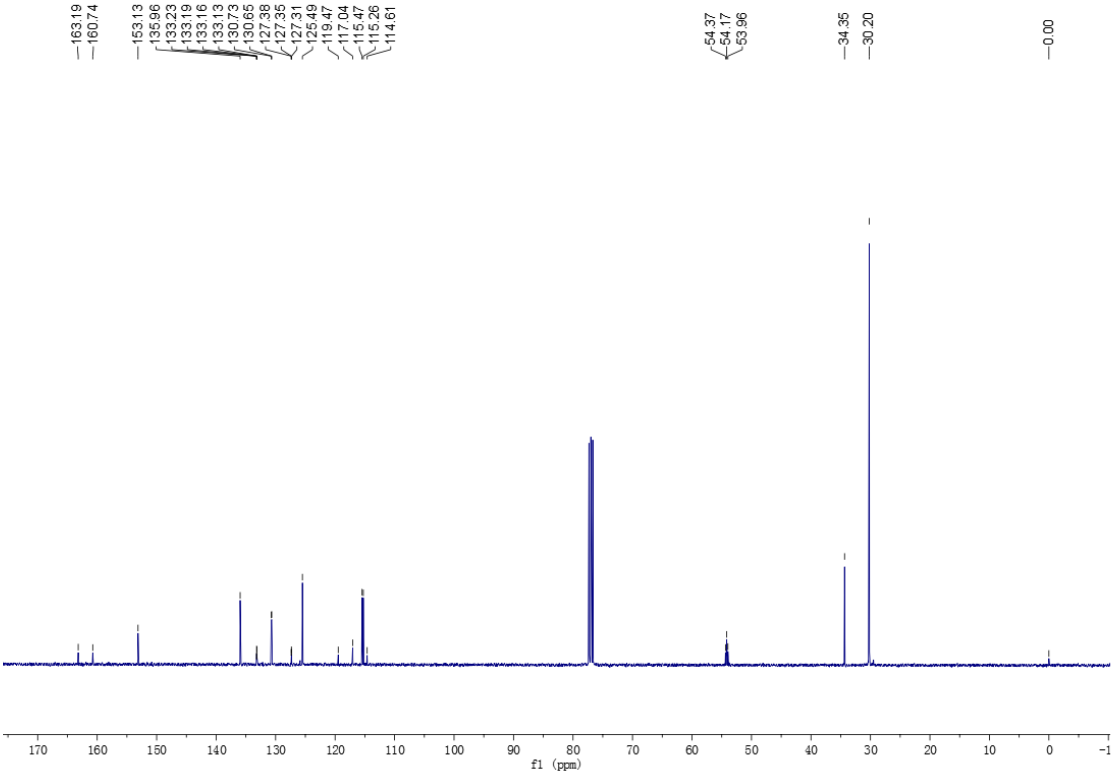

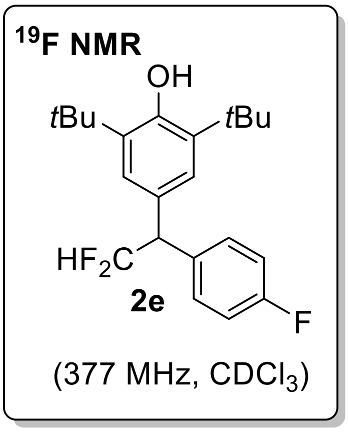
19F NMR
OH
tBu
tBu
HF2C
2e
F
(377 MHz, CDCl3)

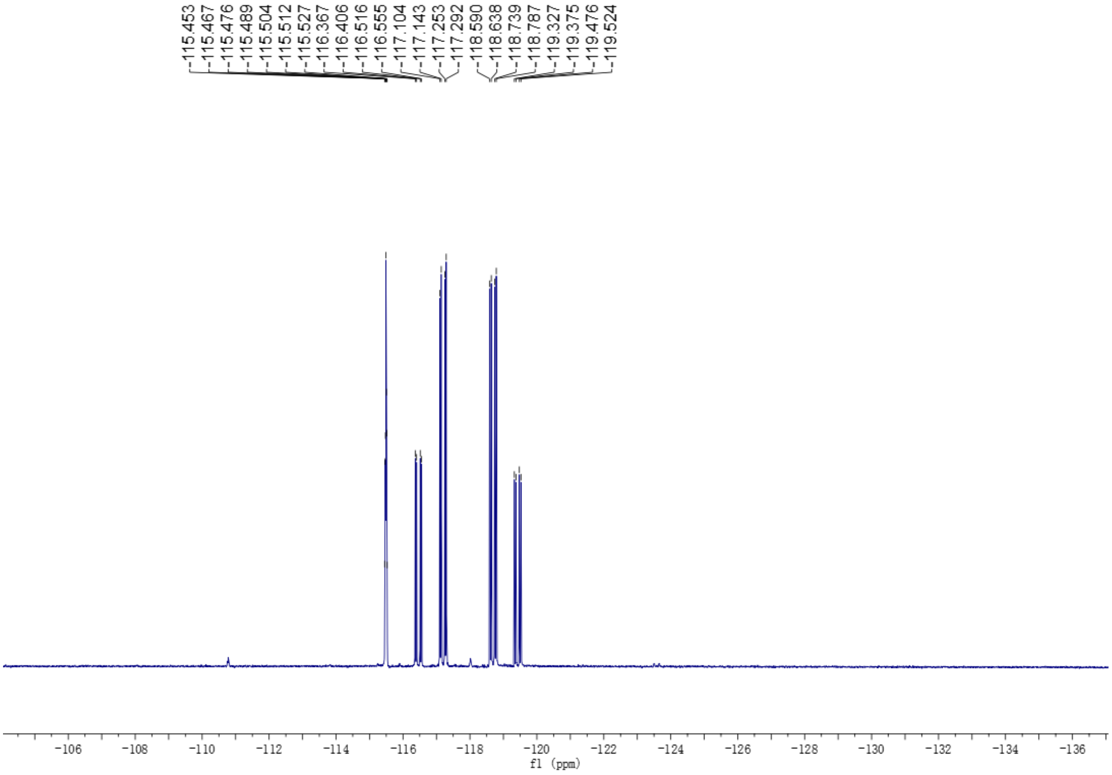
-115.453
-115.467
-115.476
-115.489
-115.504
-115.512
-115.527
-116.367
-116.406
-116.516
-116.555
-117.104
-117.143
-117.253
-117.292
-118.590
-118.638
-118.739
-118.787
-119.327
-119.375
-119.476
-119.524
-106
-108
-110
-112
-114
-116
-118
-120
-122
-124
-126
-128
-130
-132
-134
-136
f1 (ppm)

**$^{1}$H NMR**

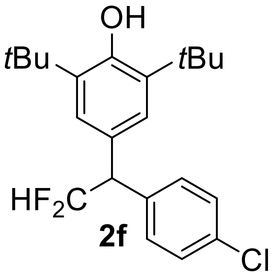


**2f**

(400 MHz, $CDCl_3$)

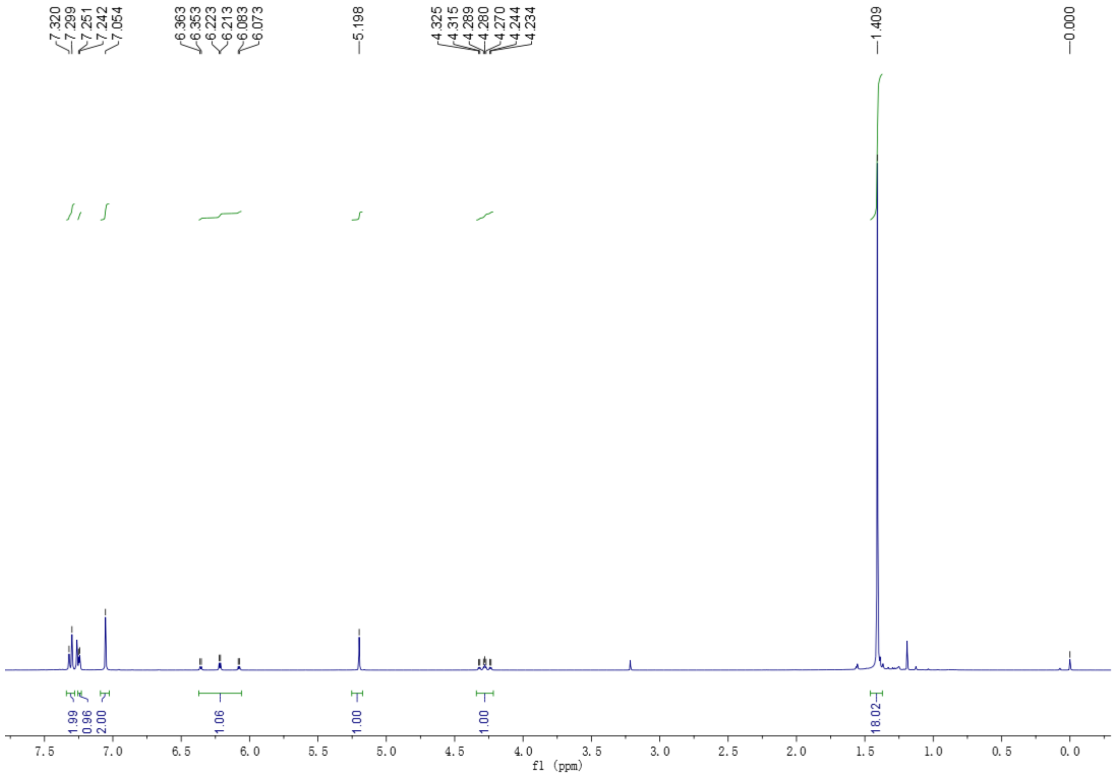


**$^{13}C\{^{1}H\}$ NMR**

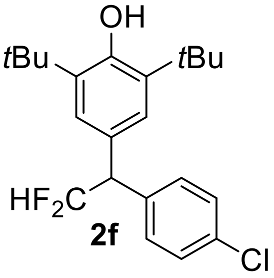


**2f**

(101 MHz, $CDCl_3$)

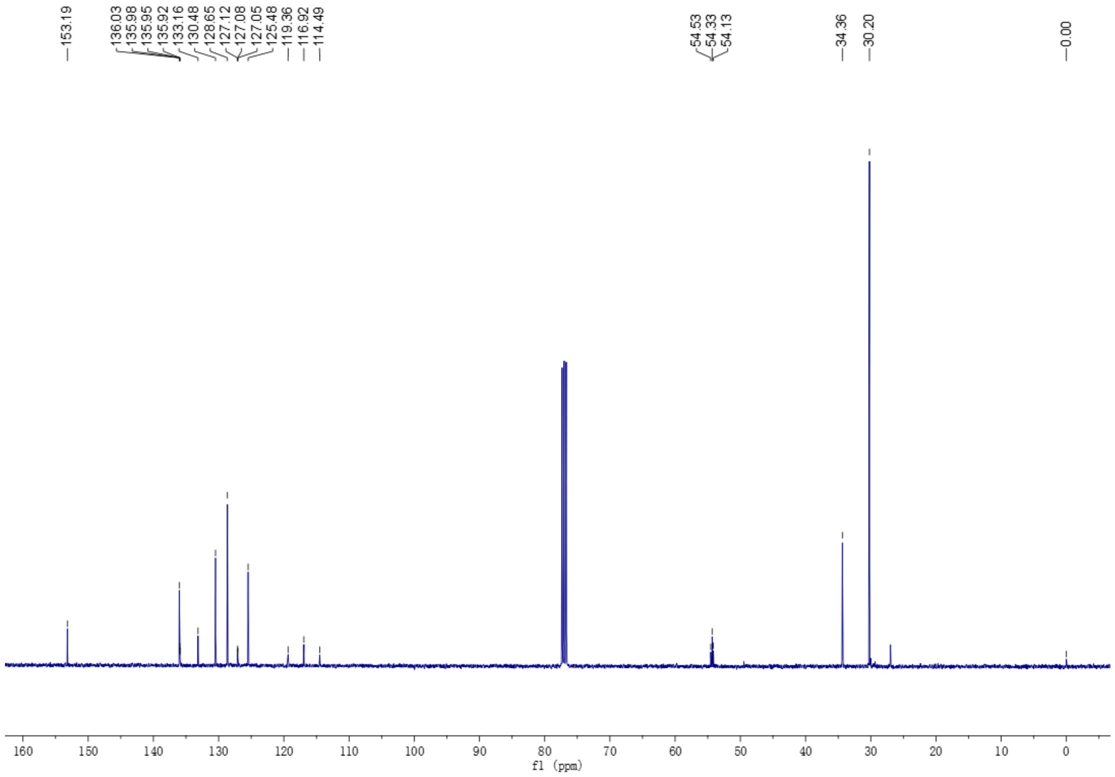

153.19
136.03
135.98
135.95
135.92
133.16
130.48
128.65
127.12
127.08
127.05
125.48
119.36
116.92
114.49
54.53
54.33
54.13
34.36
30.20
0.00
160
150
140
130
120
110
100
90
80
70
60
50
40
30
20
10
0
f1 (ppm)

**$^{19}$F NMR**

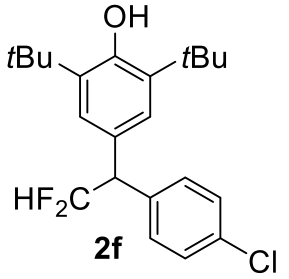


**2f**

(377 MHz, $CDCl_3$)

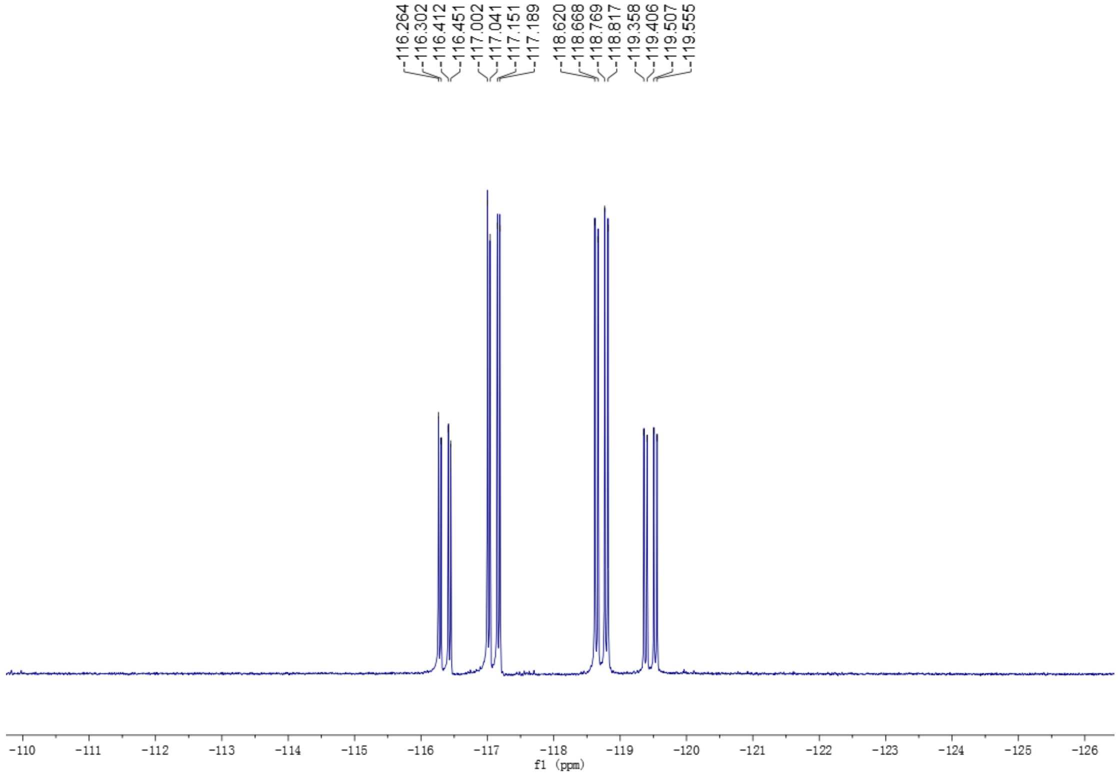

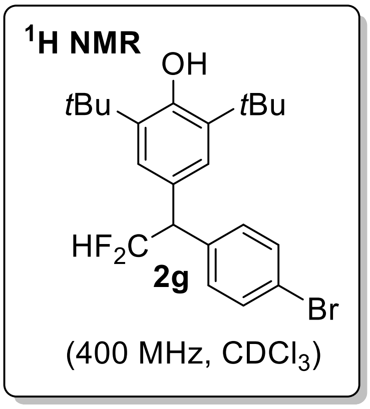

1H NMR
OH
tBu
tBu
HF2C
2g
Br
(400 MHz, CDCl3)


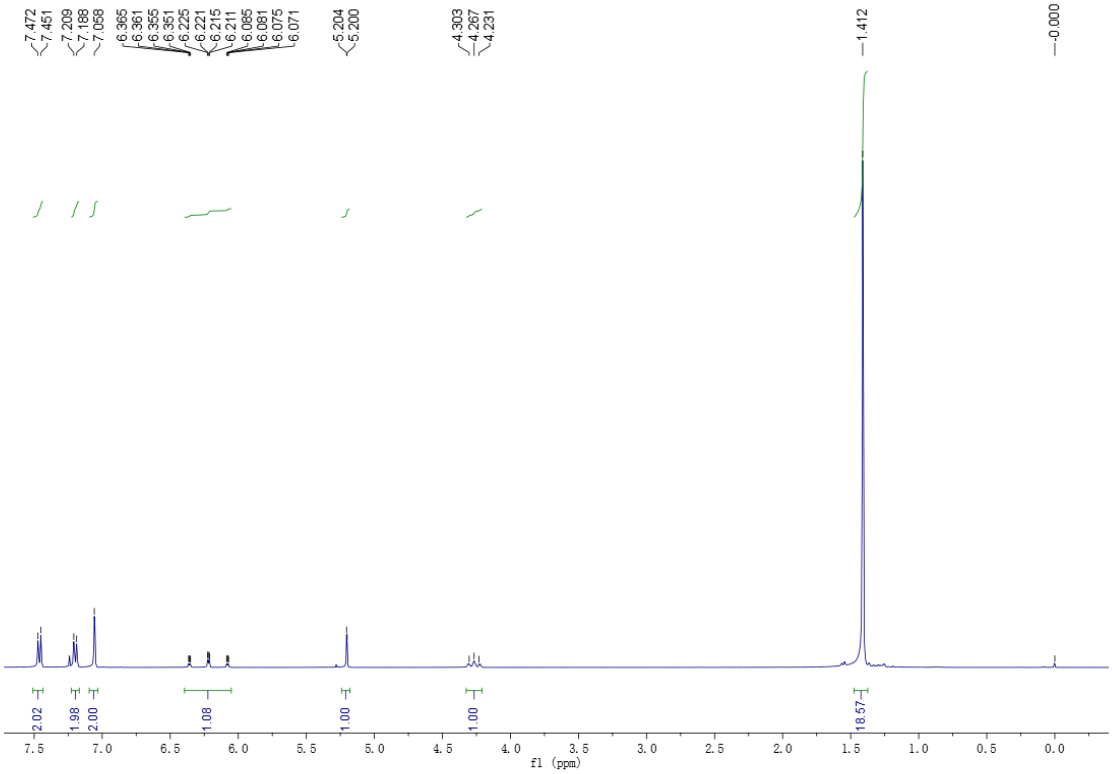

7.472
7.451
7.209
7.188
7.058
6.365
6.361
6.355
6.351
6.225
6.221
6.215
6.211
6.085
6.081
6.075
6.071
5.204
5.200
4.303
4.267
4.231
1.412
0.000
2.02
1.98
2.00
1.08
1.00
1.00
18.57
f1 (ppm)

**$^{13}C\{^{1}H\}$ NMR**

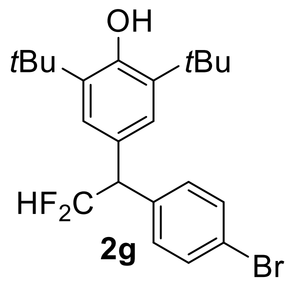


**2g**

(101 MHz, $CDCl_3$)

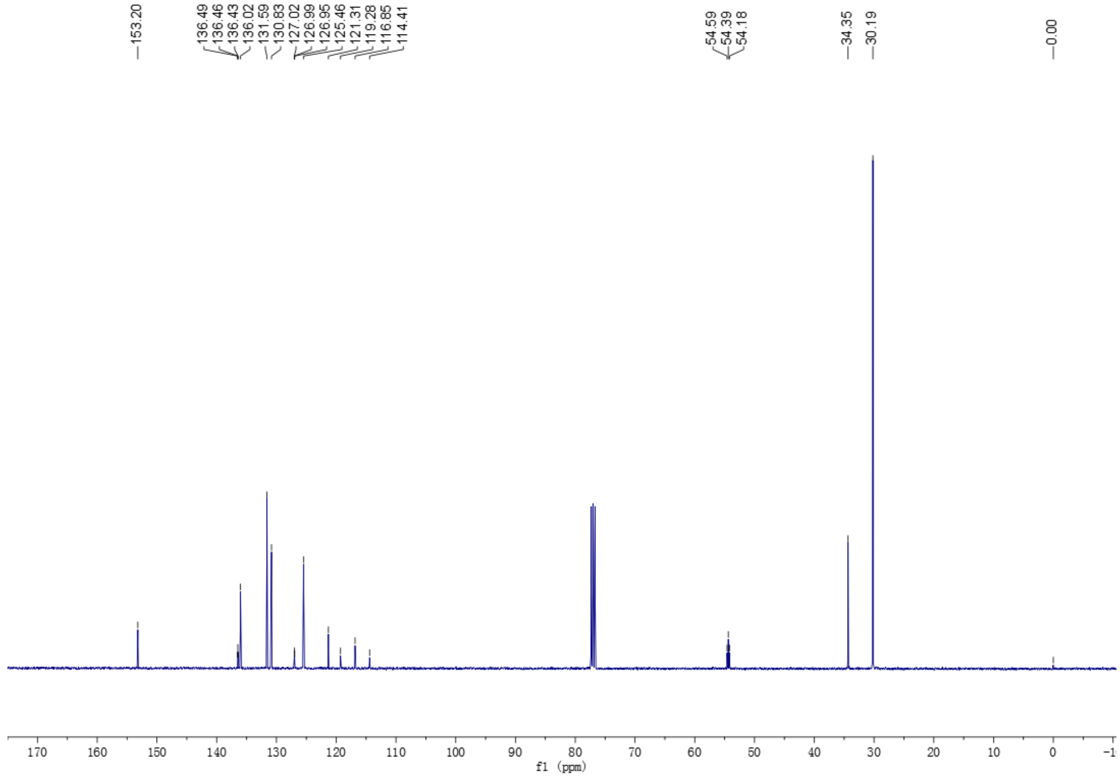

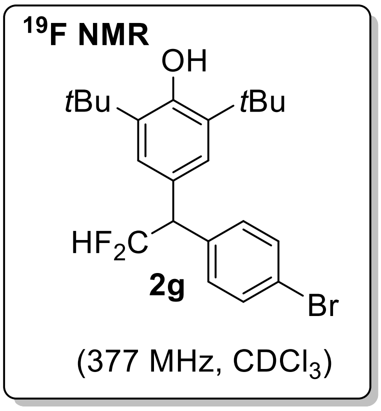

19F NMR
OH
tBu
tBu
HF2C
2g
Br
(377 MHz, CDCl3)


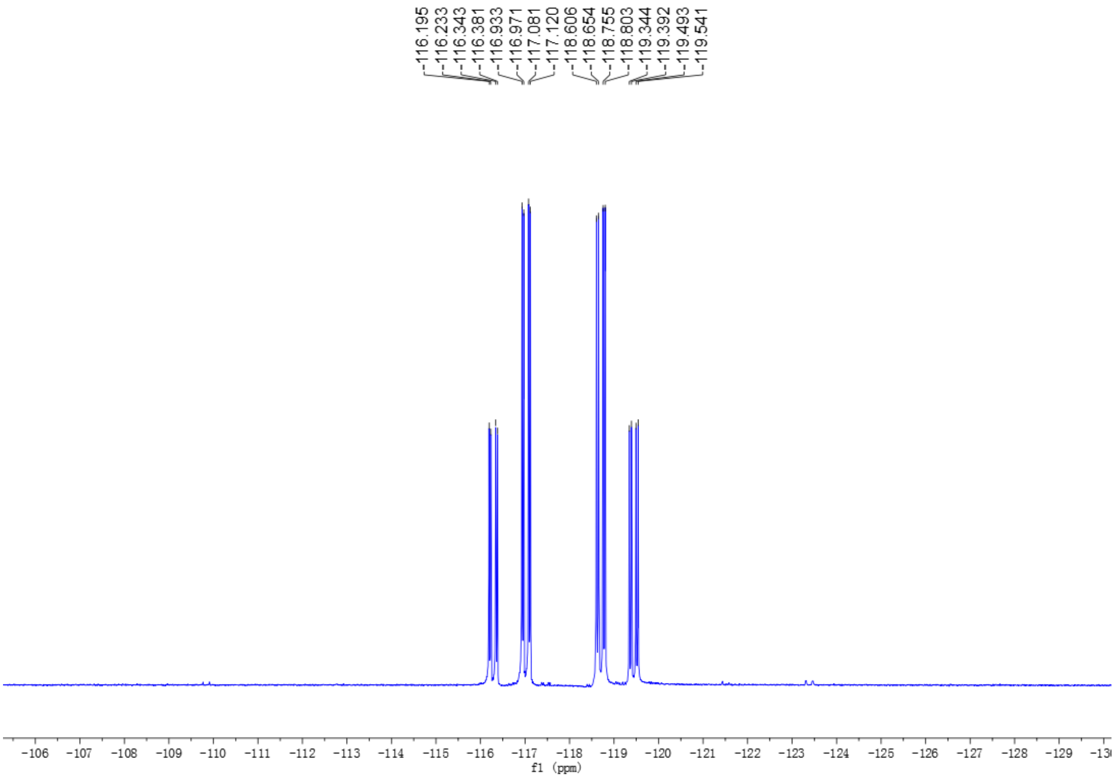

-116.195
-116.233
-116.343
-116.381
-116.933
-116.971
-117.081
-117.120
-118.606
-118.654
-118.755
-118.803
-119.344
-119.392
-119.493
-119.541
-106 -107 -108 -109 -110 -111 -112 -113 -114 -115 -116 -117 -118 -119 -120 -121 -122 -123 -124 -125 -126 -127 -128 -129
f1 (ppm)

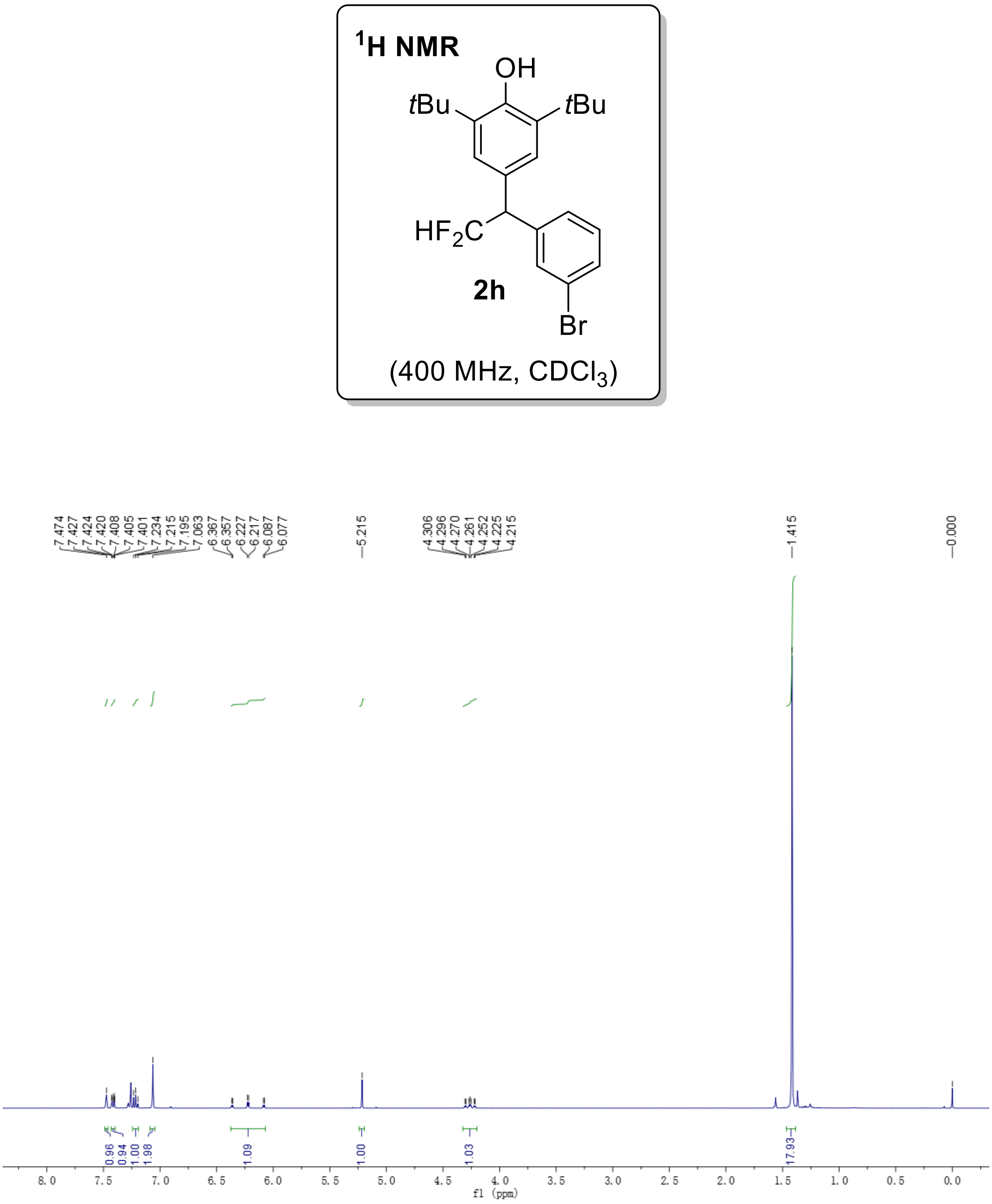

1H NMR
OH
tBu
tBu
HF2C
2h
Br
(400 MHz, CDCl3)
7.474 7.427 7.424 7.420 7.408 7.405 7.401 7.234 7.215 7.195 7.063 6.367 6.357 6.227 6.217 6.087 6.077
5.215
4.306 4.296 4.270 4.261 4.252 4.225 4.215
1.415
0.000
0.96 0.94 1.00 1.98
1.09
1.00
1.03
17.93
8.0 7.5 7.0 6.5 6.0 5.5 5.0 4.5 4.0 3.5 3.0 2.5 2.0 1.5 1.0 0.5 0.0
f1 (ppm)

**$^{13}C\{^1H\}$ NMR**

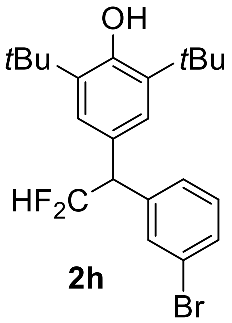


**2h**

(101 MHz, $CDCl_3$)

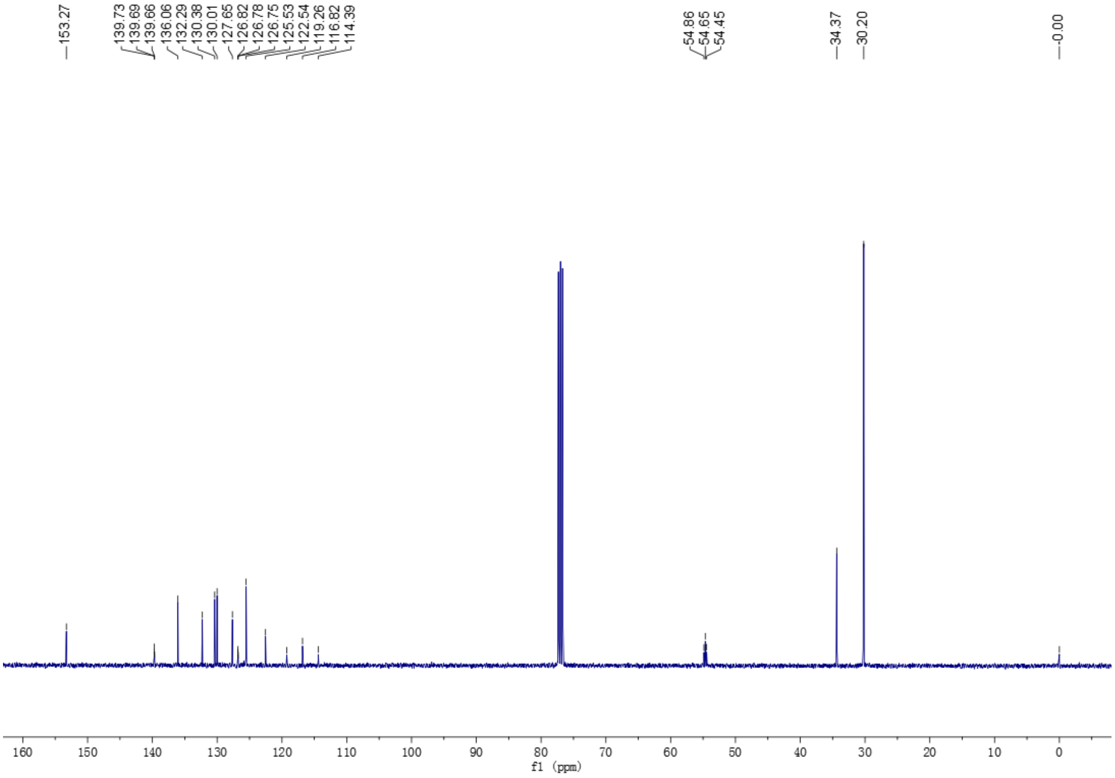

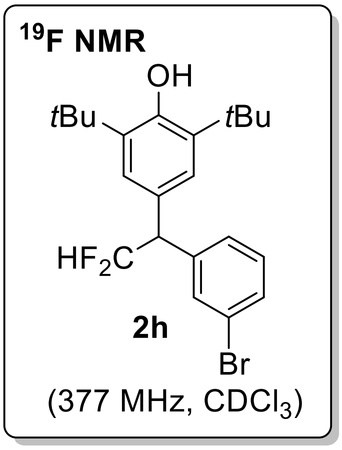

19F NMR
OH
tBu
tBu
HF2C
2h
Br
(377 MHz, CDCl3)

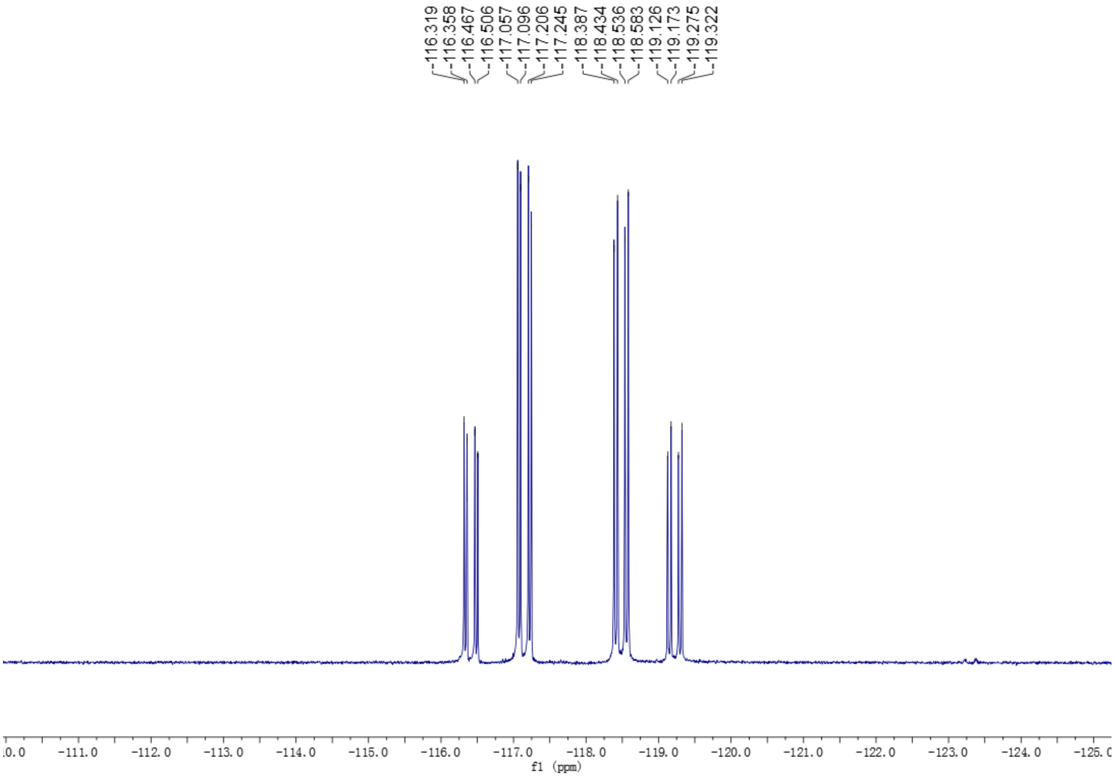


**$^{1}$H NMR**

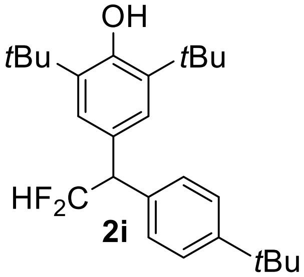


**2i**

(400 MHz, $CDCl_3$)

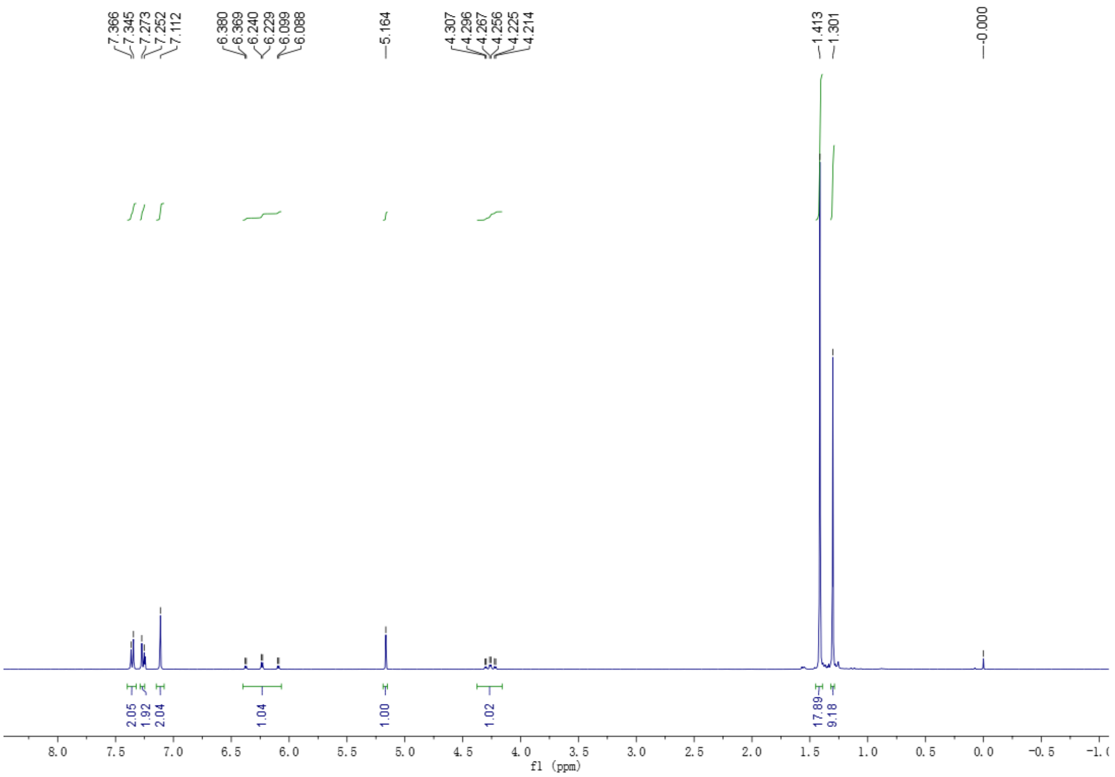


**$^{13}C\{^1H\}$ NMR**

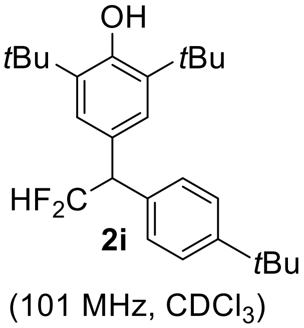


**2i**

(101 MHz, $CDCl_3$)

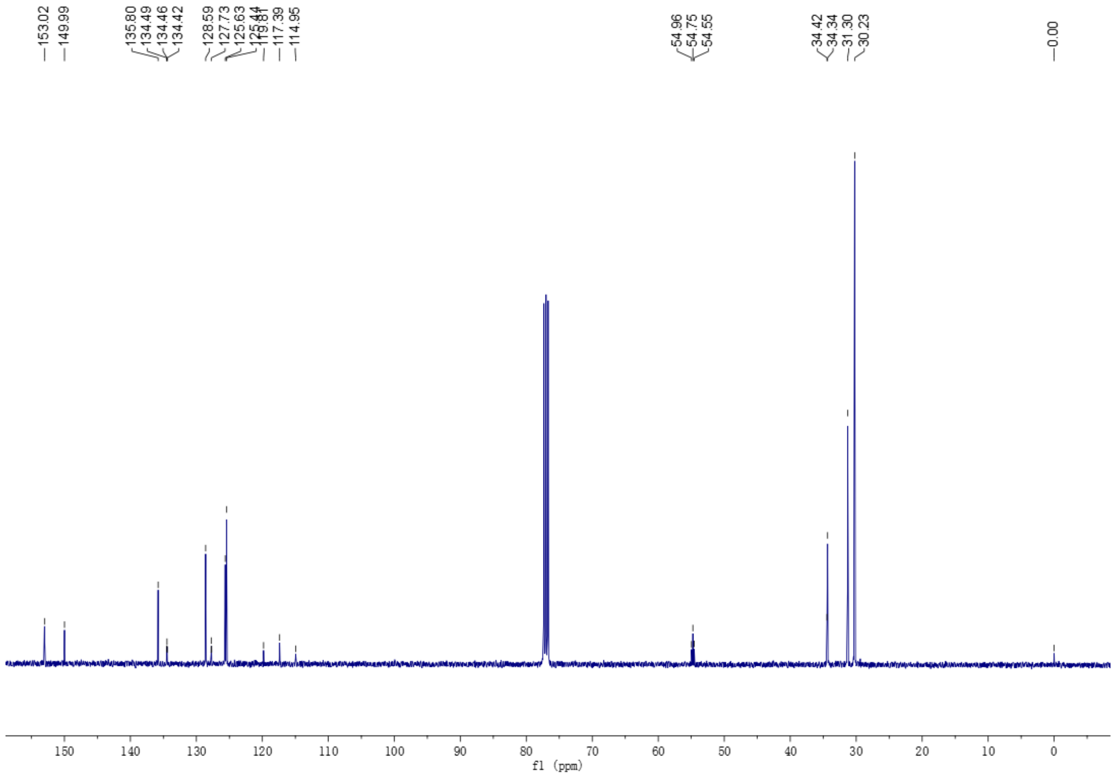

153.02
149.99
135.80
134.49
134.46
134.42
128.59
127.73
125.63
125.44
119.81
117.39
114.95
54.96
54.75
54.55
34.42
34.34
31.30
30.23
0.00
150
140
130
120
110
100
90
80
70
60
50
40
30
20
10
0
f1 (ppm)

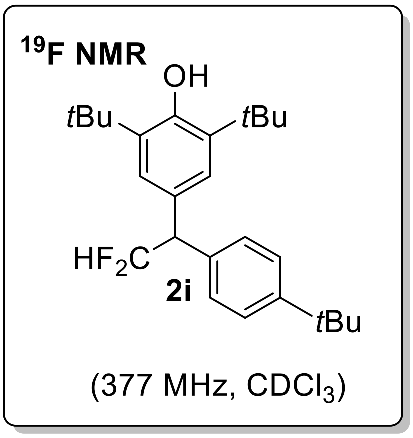

19F NMR
OH
tBu
tBu
HF2C
2i
tBu
(377 MHz, CDCl3)


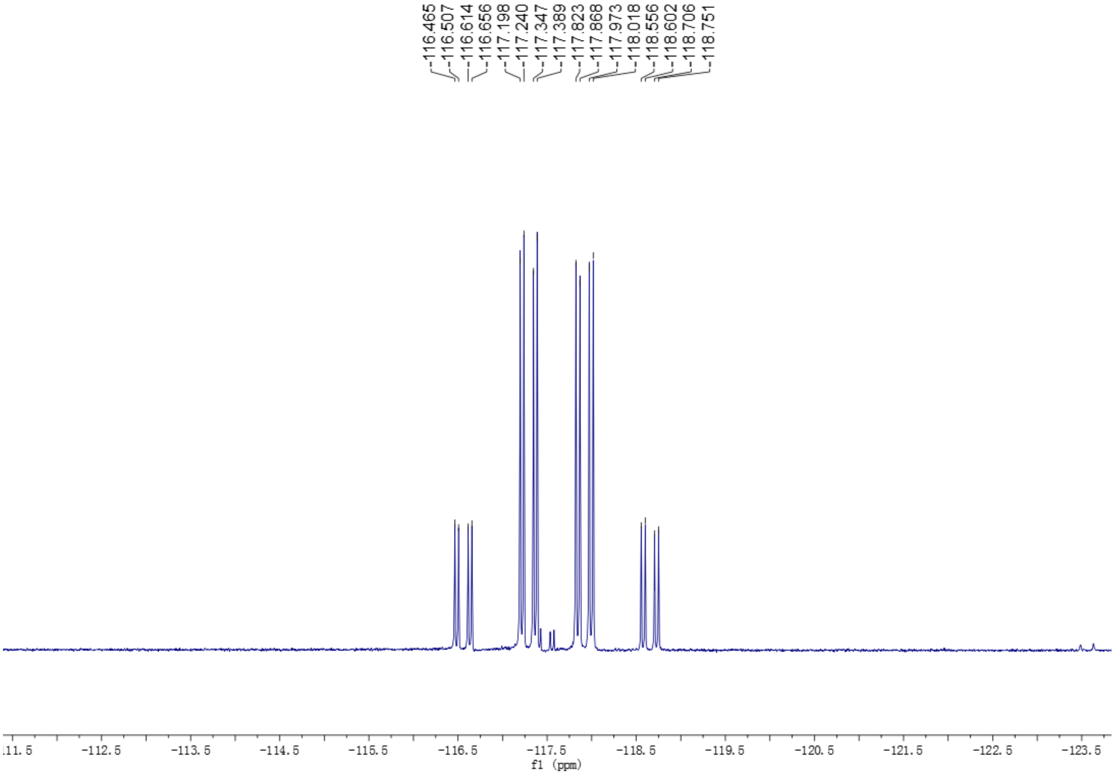

-116.465
-116.507
-116.614
-116.656
-117.198
-117.240
-117.347
-117.389
-117.823
-117.868
-117.973
-118.018
-118.556
-118.602
-118.706
-118.751
-112.5
-113.5
-114.5
-115.5
-116.5
-117.5
-118.5
-119.5
-120.5
-121.5
-122.5
-123.5
f1 (ppm)

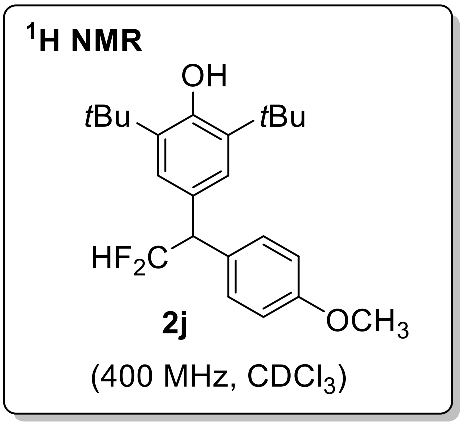

$^1$H NMR
OH
tBu
tBu
HF$_2$C
OCH$_3$
2j
(400 MHz, CDCl$_3$)


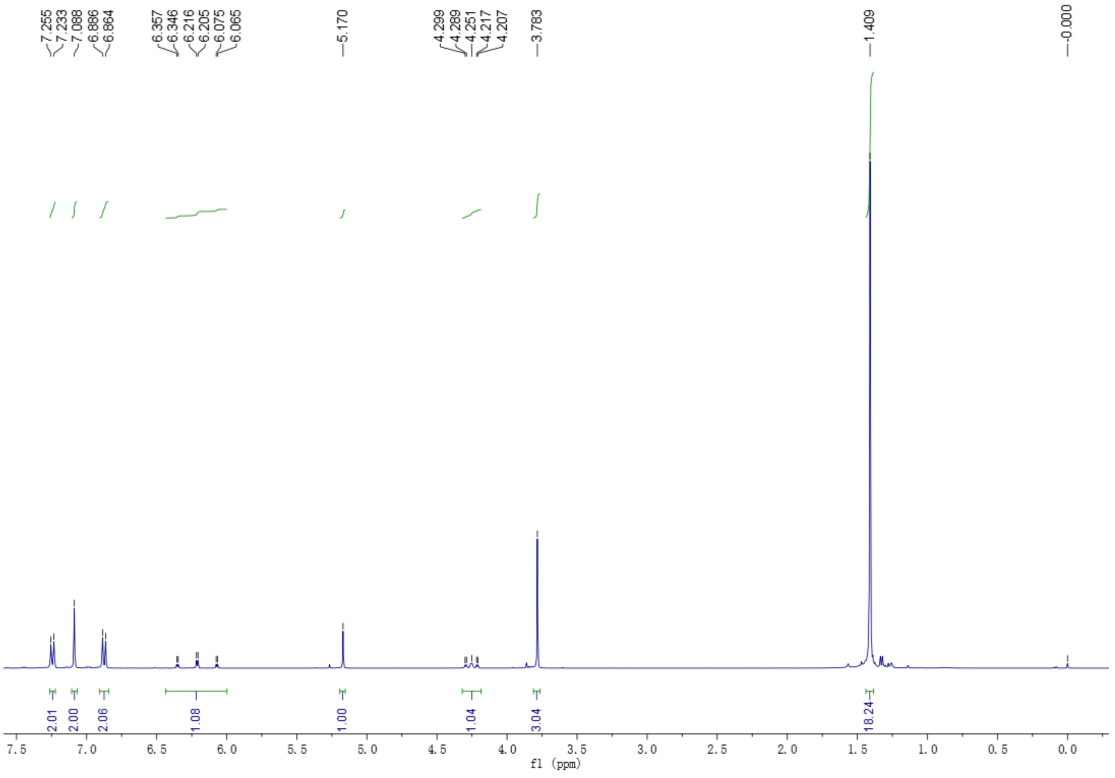

7.255
7.233
7.088
6.886
6.864
6.357
6.346
6.216
6.205
6.075
6.065
5.170
4.299
4.289
4.251
4.217
4.207
3.783
1.409
-0.000
2.01
2.00
2.06
1.08
1.00
1.04
3.04
18.24
7.5 7.0 6.5 6.0 5.5 5.0 4.5 4.0 3.5 3.0 2.5 2.0 1.5 1.0 0.5 0.0
f1 (ppm)

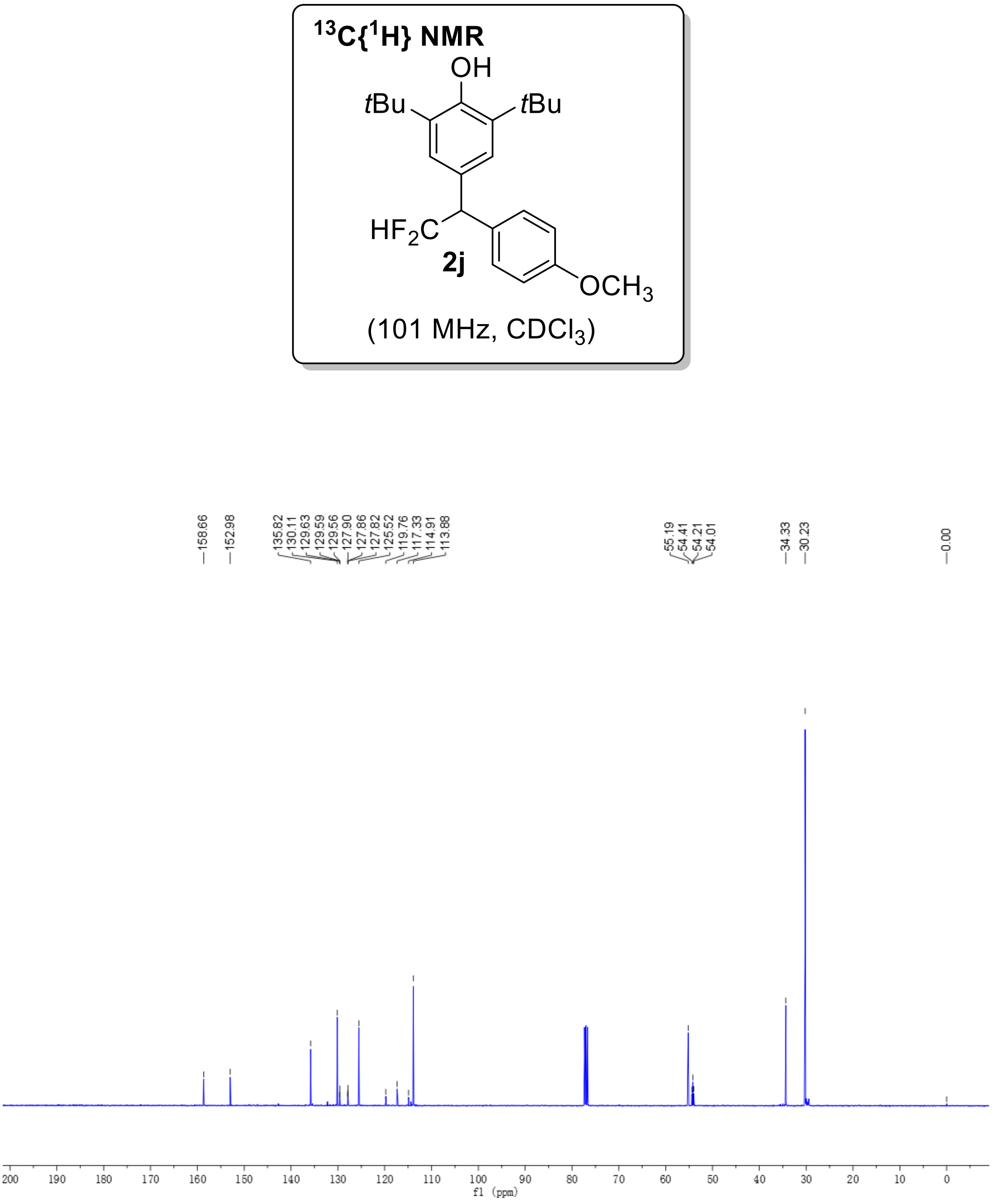
13C{1H} NMR
OH
tBu
tBu
HF2C
2j
OCH3
(101 MHz, CDCl3)
158.66
152.98
135.82
130.11
129.63
129.59
129.56
127.90
127.86
127.82
125.52
119.76
117.33
114.91
113.88
55.19
54.41
54.21
54.01
34.33
30.23
0.00
f1 (ppm)

**$^{19}$F NMR**

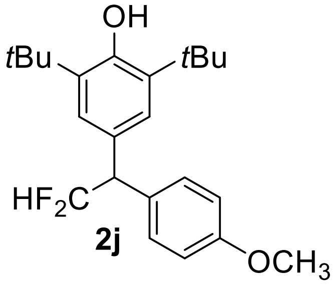


(377 MHz, $CDCl_3$)

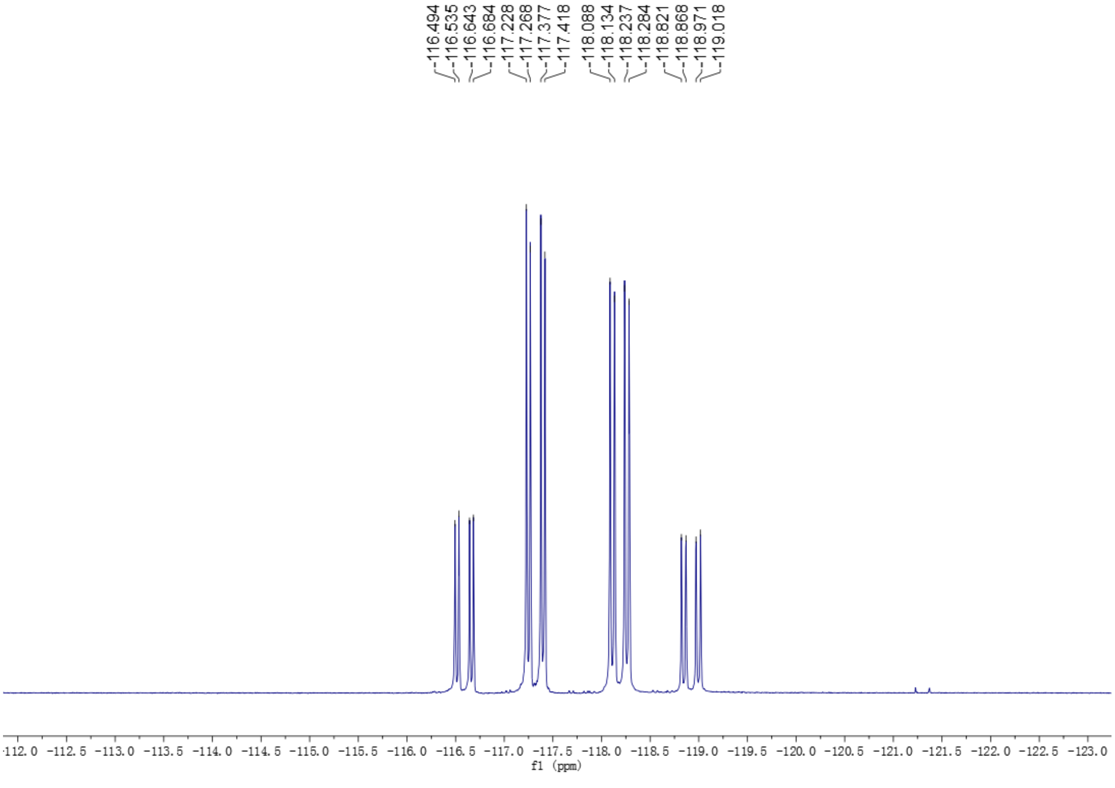

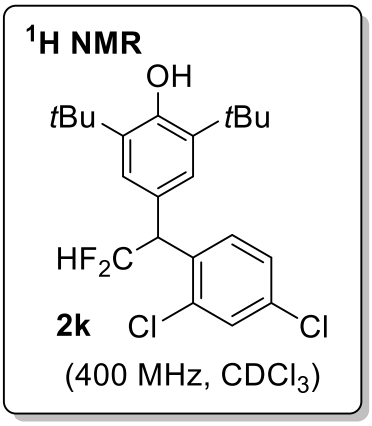

$^1$H NMR
OH
tBu
tBu
HF$_2$C
2k
Cl
Cl
(400 MHz, CDCl$_3$)


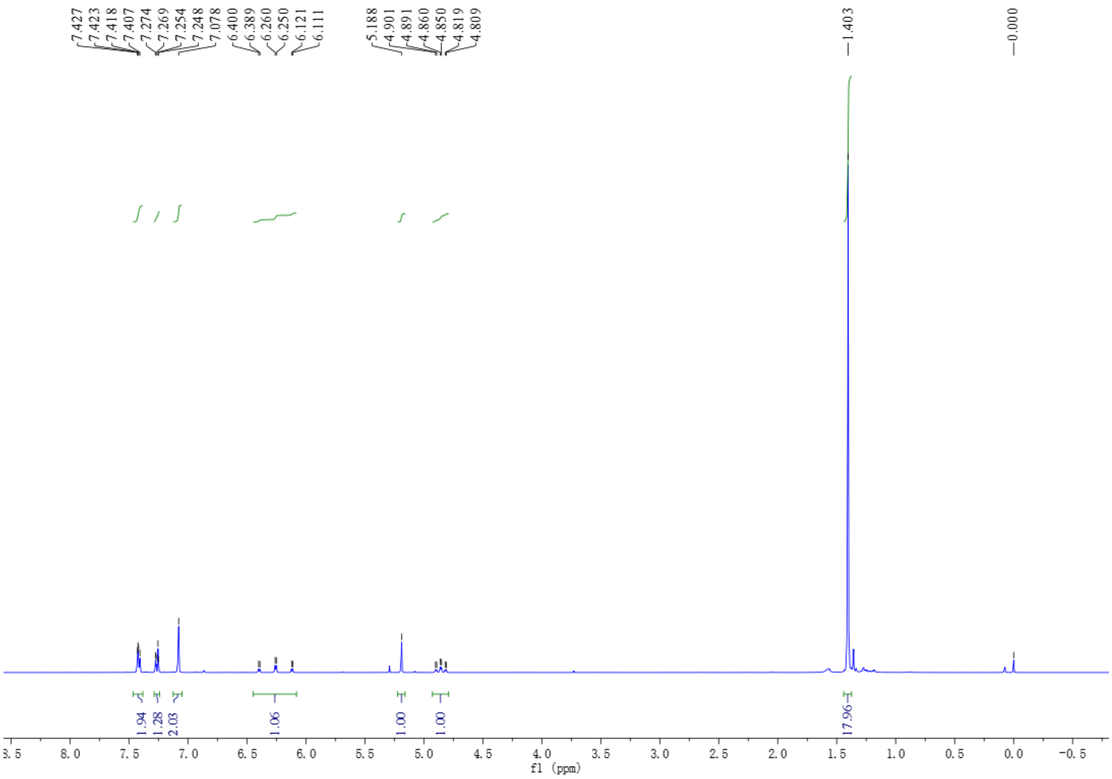

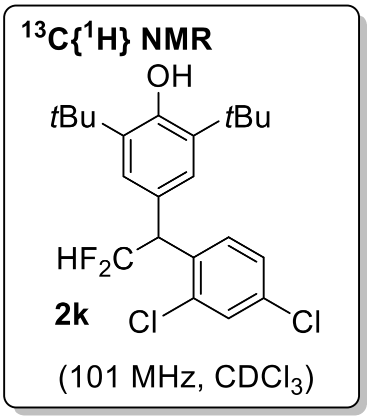

13C{1H} NMR
OH
tBu
tBu
HF2C
2k
Cl
Cl
(101 MHz, CDCl3)

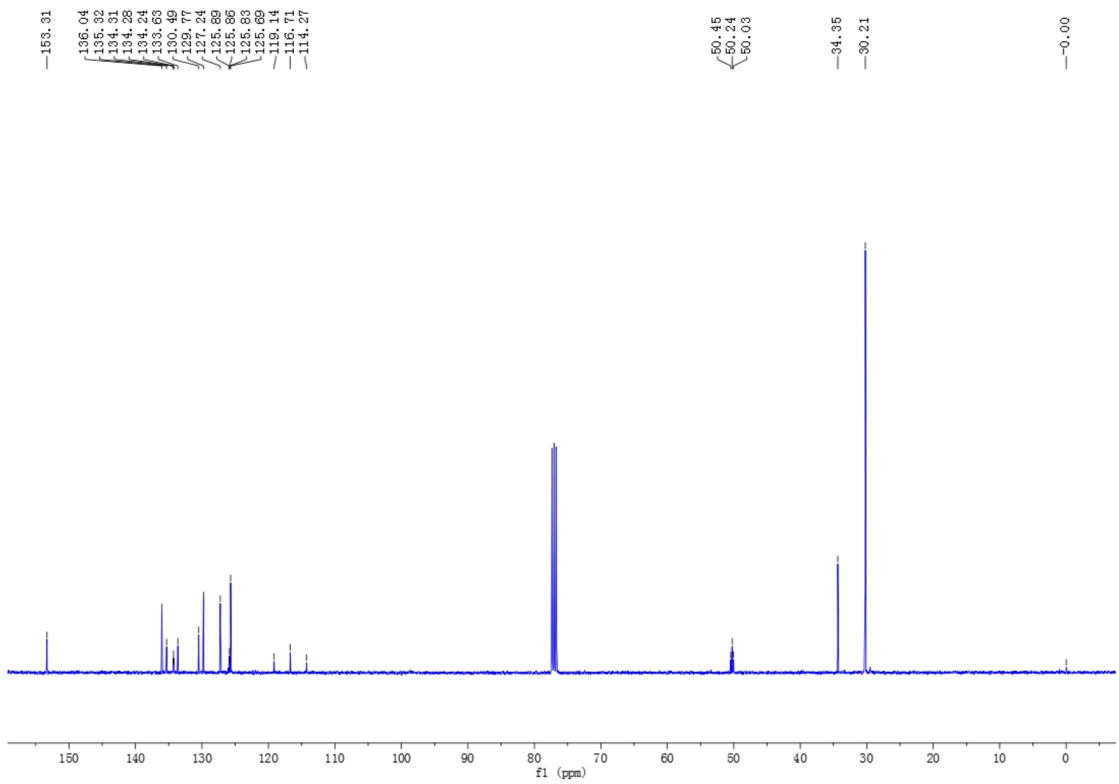


**$^{19}$F NMR**

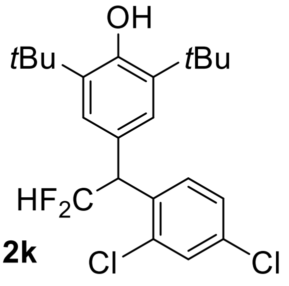


**2k**

(377 MHz, $CDCl_3$)

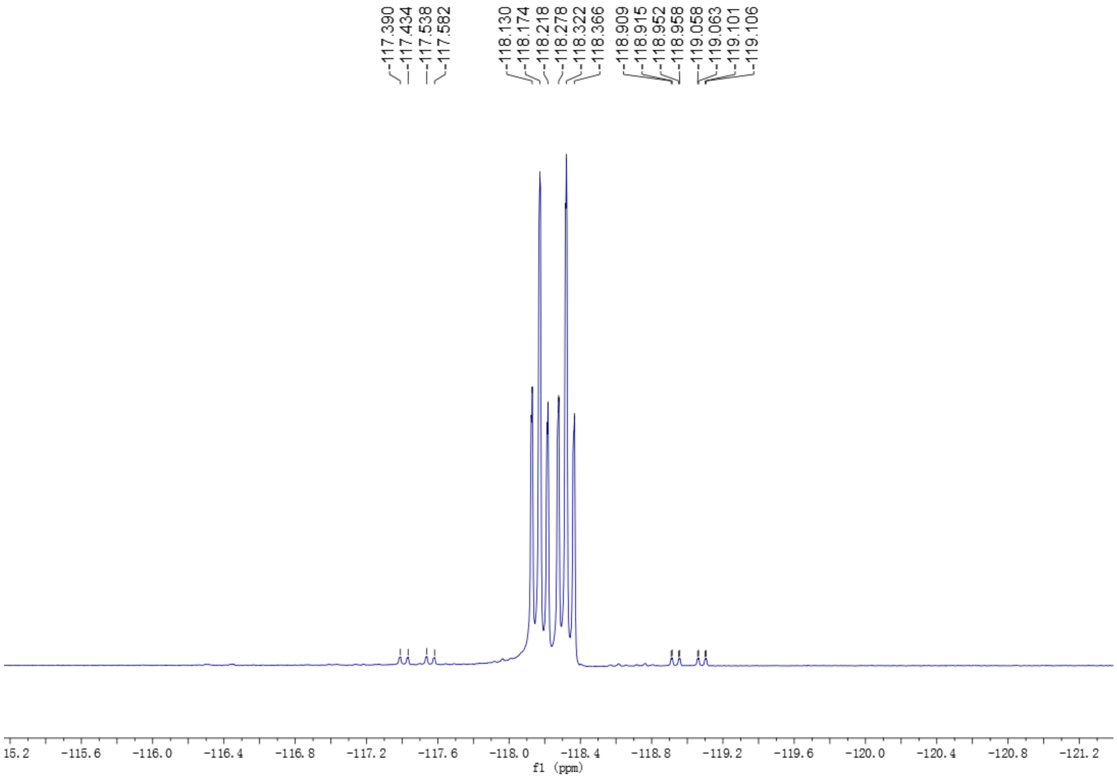


**$^1$H NMR**

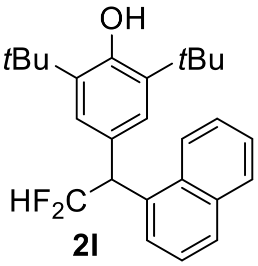


**2l**

(400 MHz, $CDCl_3$)

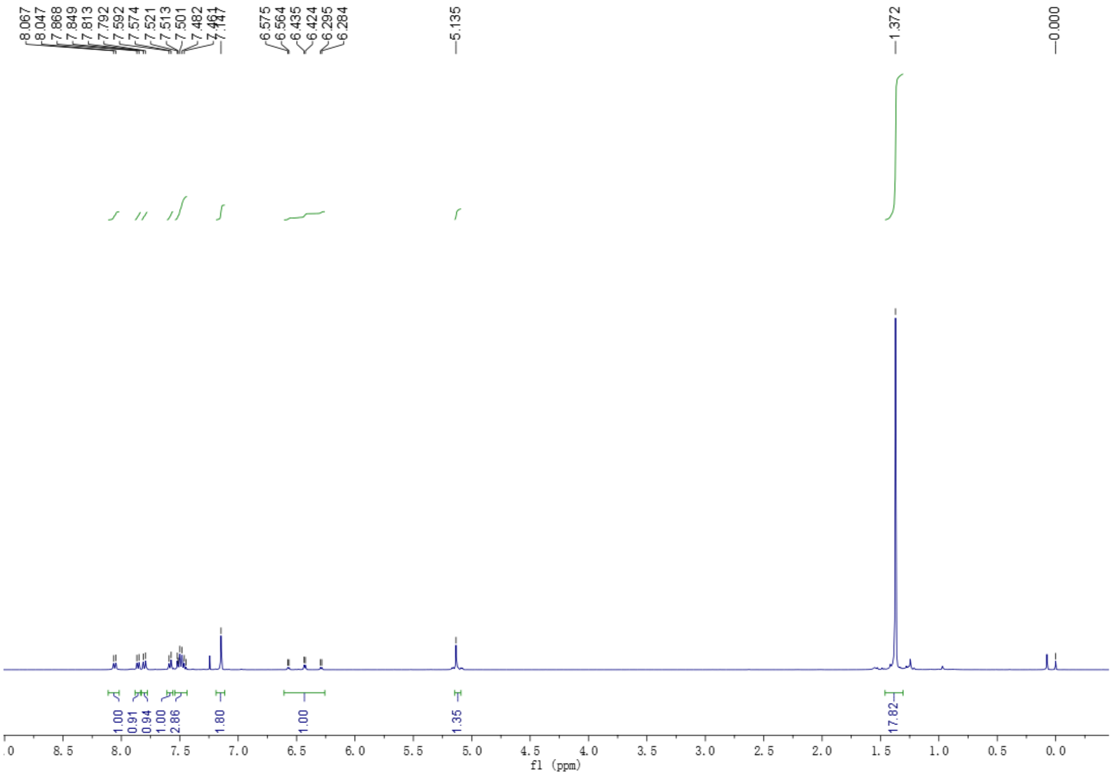


**$^{13}C\{^{1}H\}$ NMR**

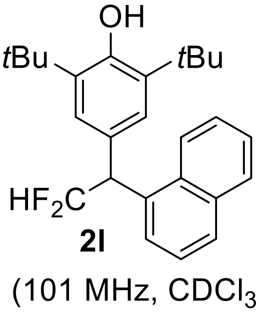


**2l**

(101 MHz, $CDCl_3$)

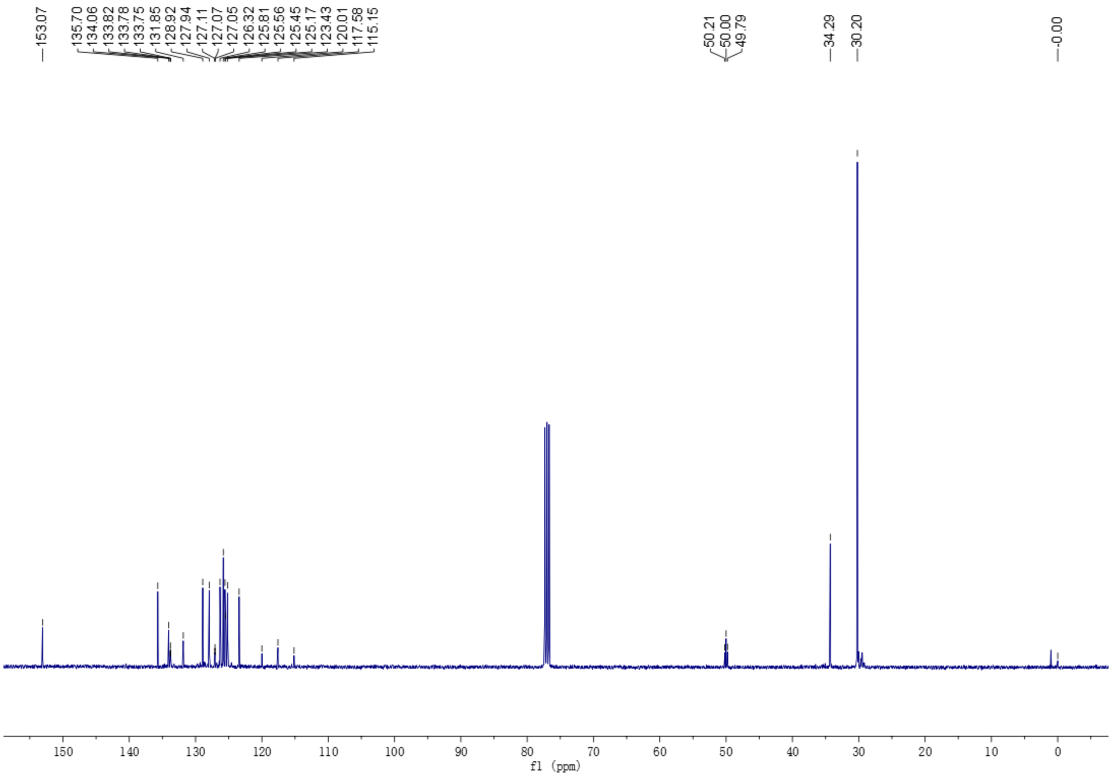
153.07
135.70
134.06
133.82
133.78
133.75
131.85
128.92
127.94
127.11
127.07
127.05
126.32
125.81
125.56
125.45
125.17
123.43
120.01
117.58
115.15
50.21
50.00
49.79
34.29
30.20
0.00
150
140
130
120
110
100
90
80
70
60
50
40
30
20
10
0
f1 (ppm)

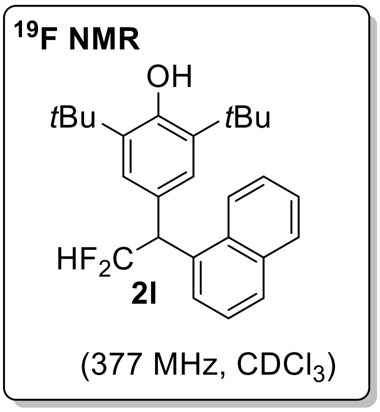

19F NMR
OH
tBu
tBu
HF2C
2l
(377 MHz, CDCl3)


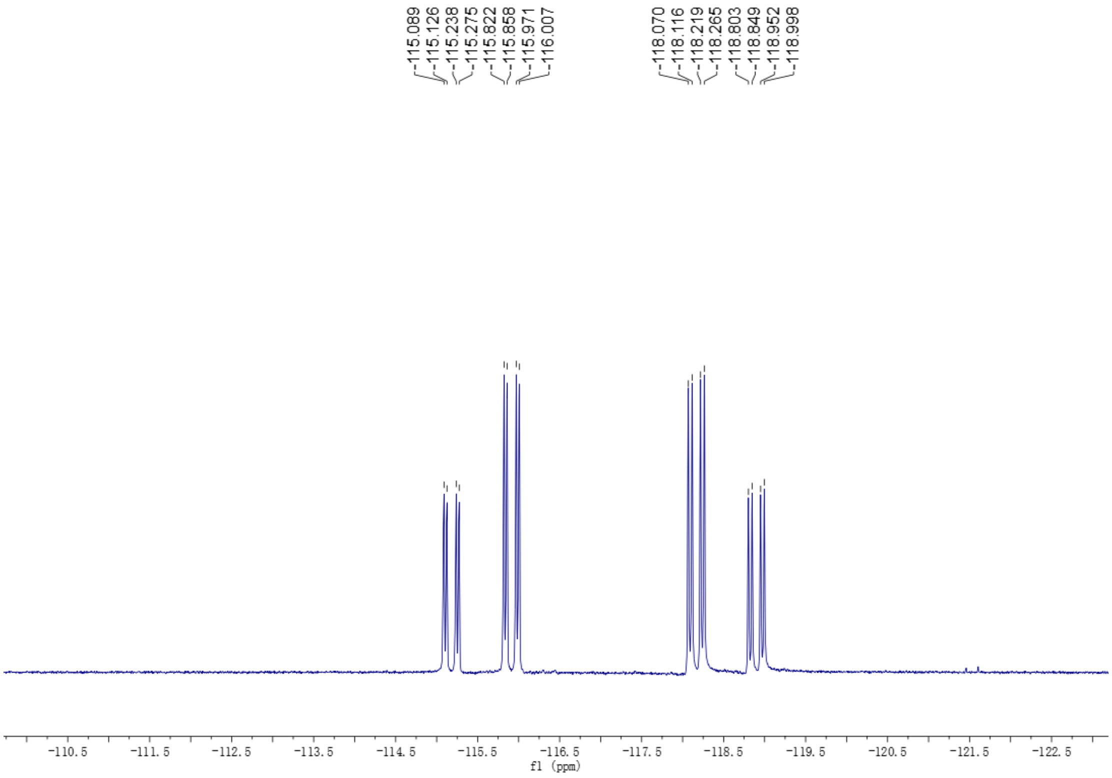

-115.089
-115.126
-115.238
-115.275
-115.822
-115.858
-115.971
-116.007
-118.070
-118.116
-118.219
-118.265
-118.803
-118.849
-118.952
-118.998
-110.5
-111.5
-112.5
-113.5
-114.5
-115.5
-116.5
-117.5
-118.5
-119.5
-120.5
-121.5
-122.5
f1 (ppm)

**$^1$H NMR**

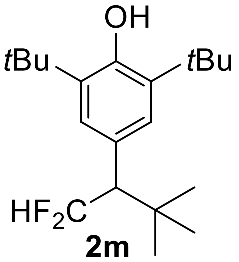


**2m**

(400 MHz, $CDCl_3$)

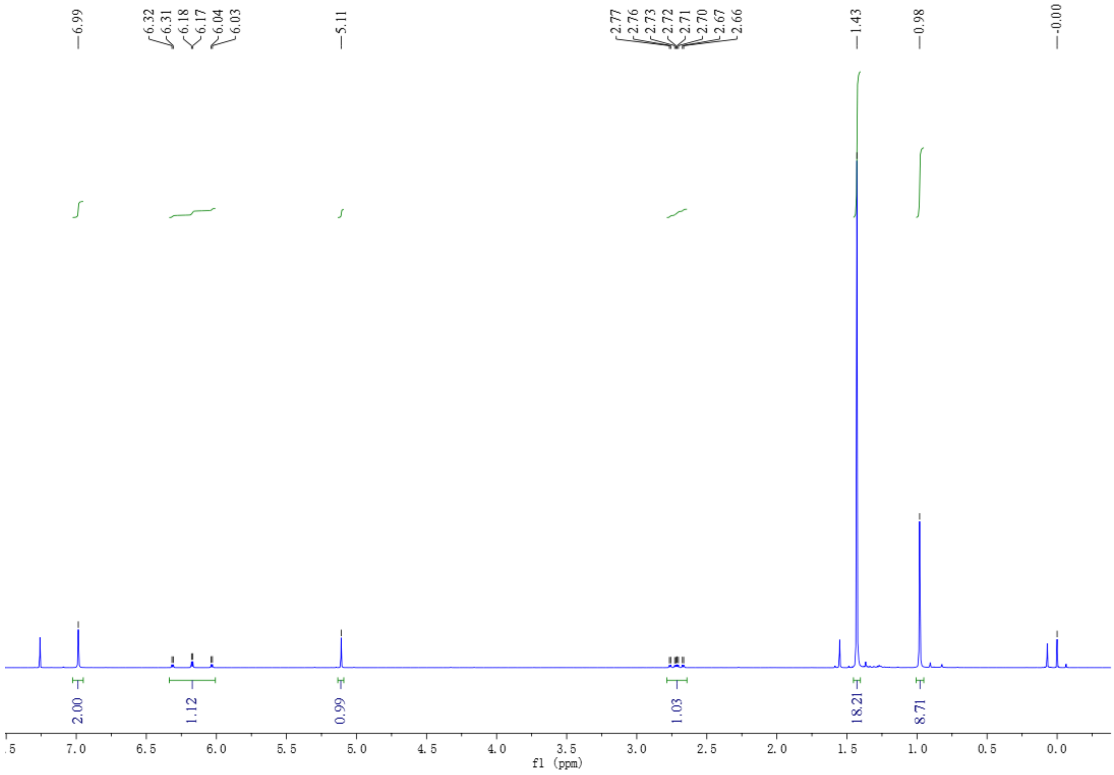

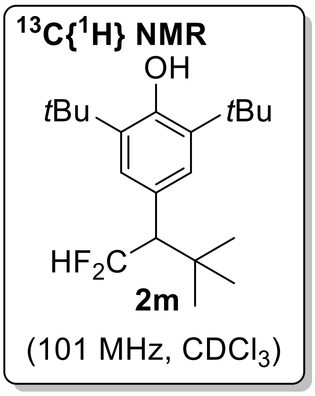

13C{1H} NMR
OH
tBu
tBu
HF2C
2m
(101 MHz, CDCl3)


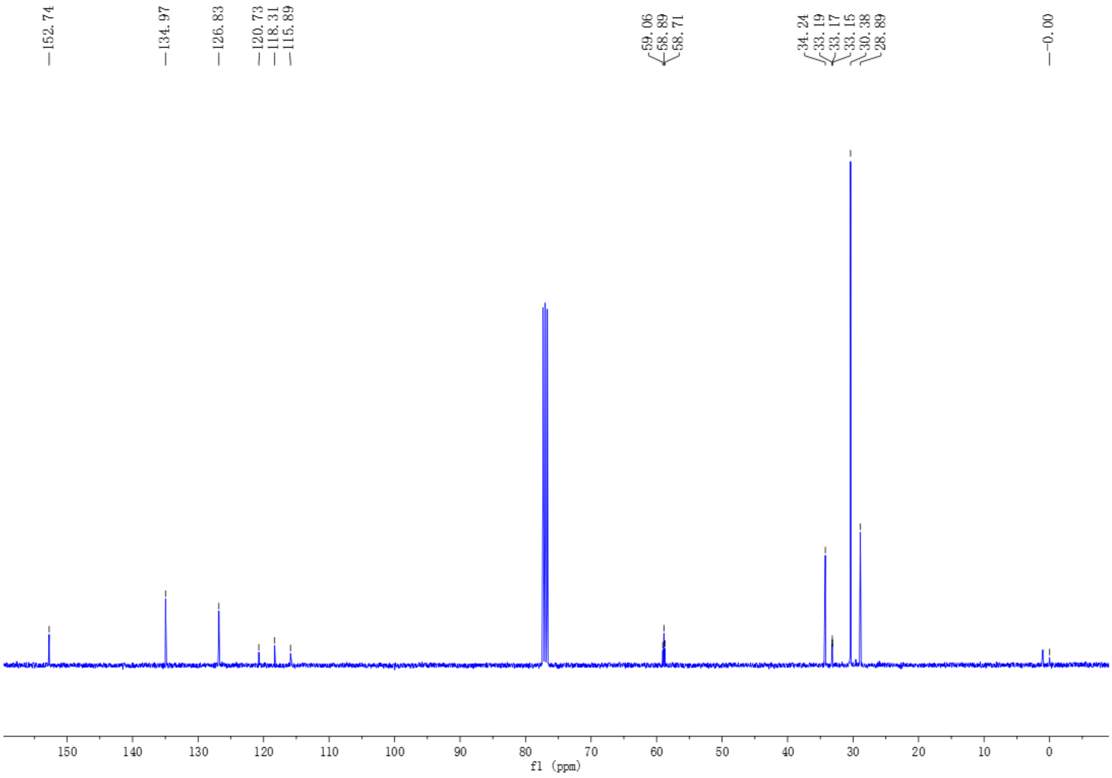

152.74
134.97
126.83
120.73
118.31
115.89
59.06
58.89
58.71
34.24
33.19
33.17
33.15
30.38
28.89
-0.00
150 140 130 120 110 100 90 80 70 60 50 40 30 20 10 0
f1 (ppm)

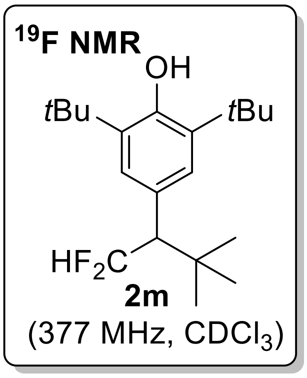
19F NMR
OH
tBu
tBu
HF2C
2m
(377 MHz, CDCl3)

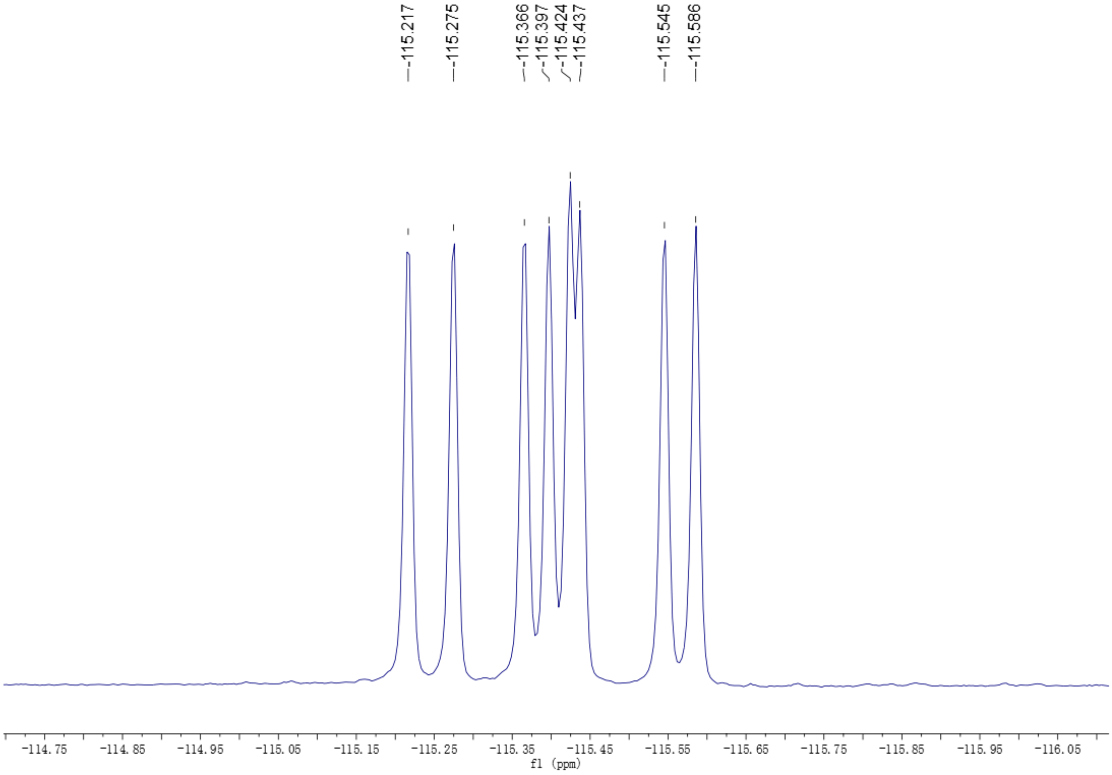
-115.217
-115.275
-115.366
-115.397
-115.424
-115.437
-115.545
-115.586
-114.75
-114.85
-114.95
-115.05
-115.15
-115.25
-115.35
-115.45
-115.55
-115.65
-115.75
-115.85
-115.95
-116.05
f1 (ppm)

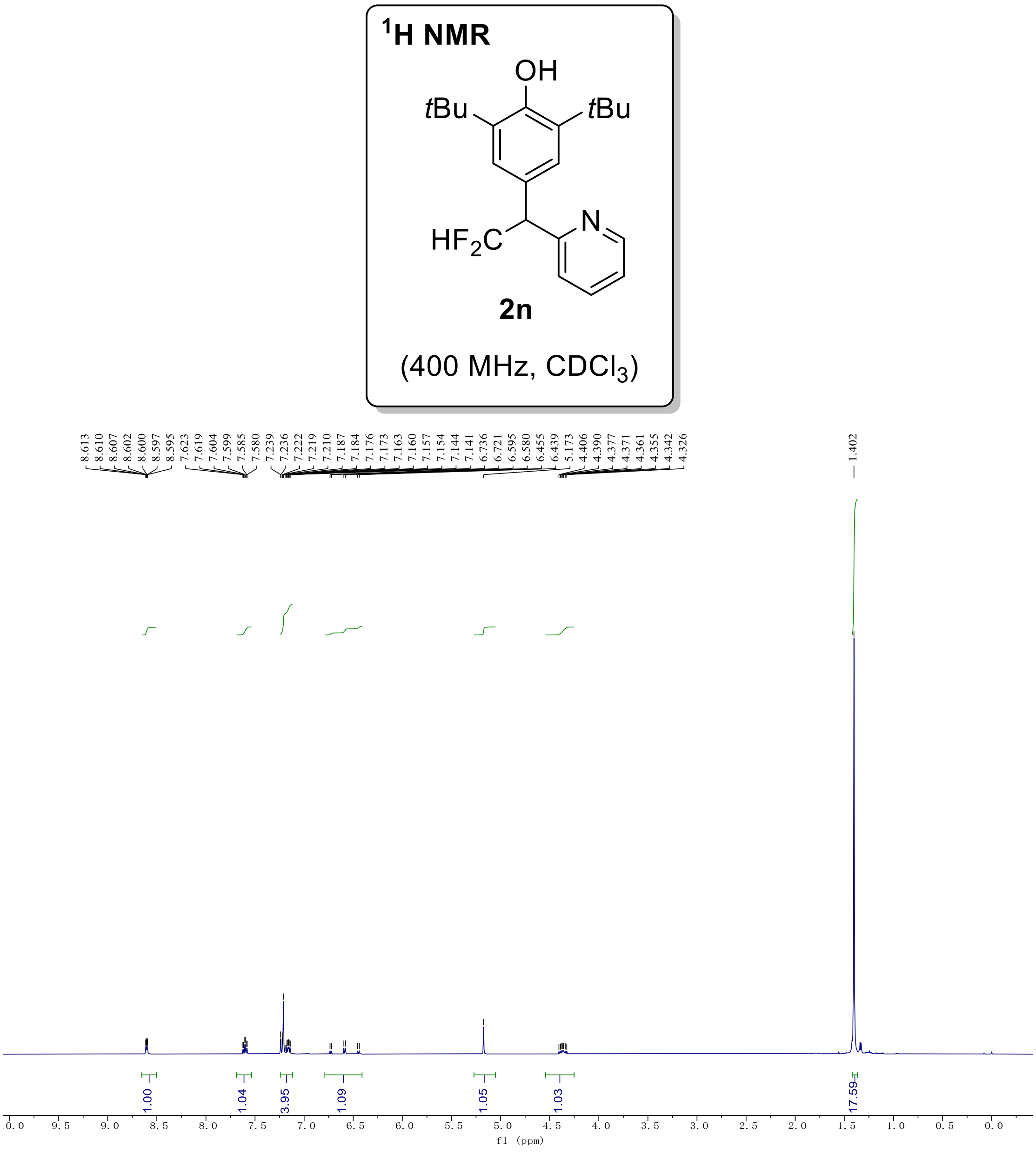
1H NMR
OH
tBu
tBu
N
HF2C
2n
(400 MHz, CDCl3)
8.613 8.610 8.607 8.602 8.600 8.597 8.595 7.623 7.619 7.604 7.599 7.585 7.580 7.239 7.236 7.222 7.219 7.210 7.187 7.184 7.176 7.173 7.163 7.160 7.157 7.154 7.144 7.141 6.736 6.721 6.595 6.580 6.455 6.439 5.173 4.406 4.390 4.377 4.371 4.361 4.355 4.342 4.326
1.402
1.00
1.04
3.95
1.09
1.05
1.03
17.59
f1 (ppm)

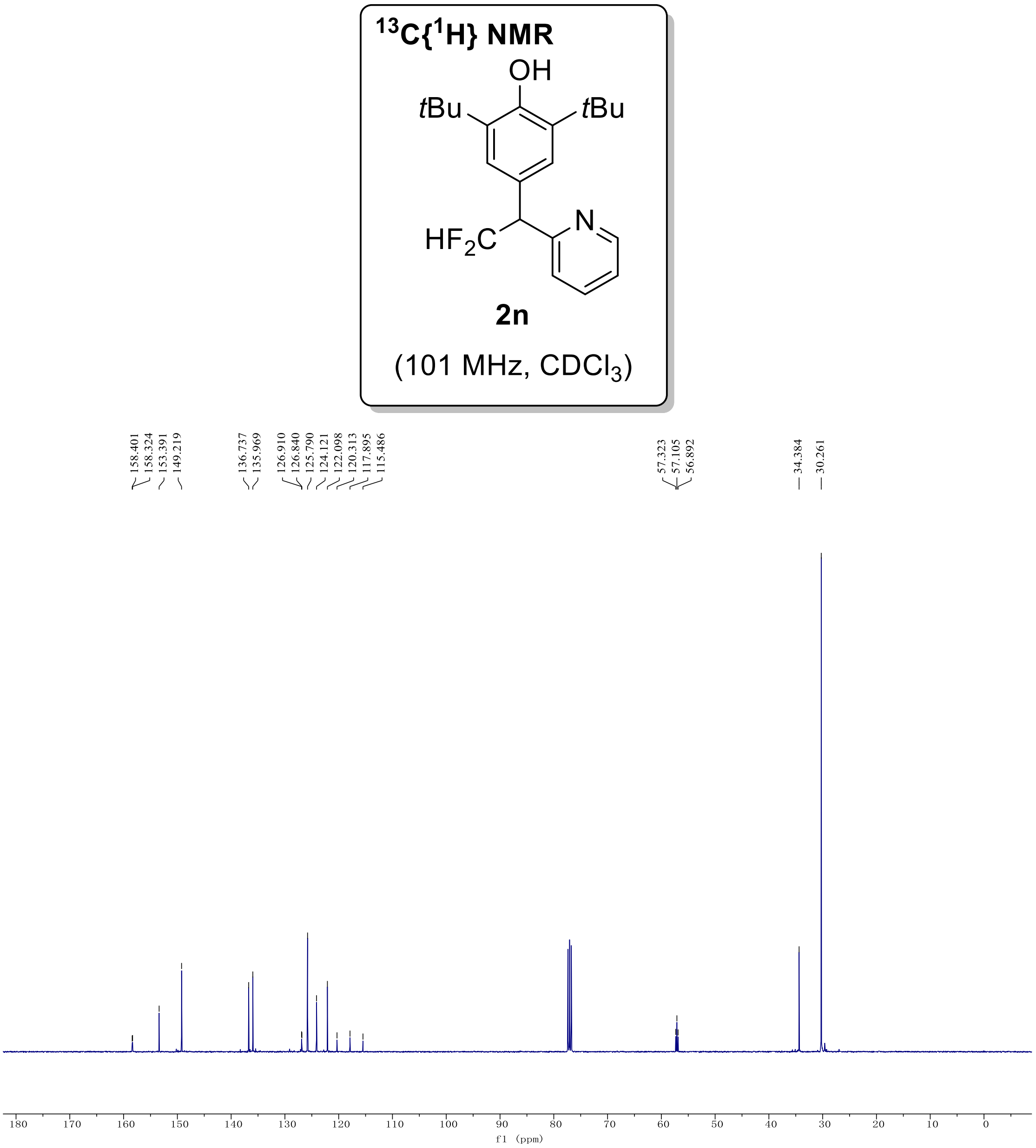

$^{13}C\{^1H\}$ NMR
OH
tBu
tBu
N
$HF_2C$
2n
(101 MHz, $CDCl_3$)
158.401
158.324
153.391
149.219
136.737
135.969
126.910
126.840
125.790
124.121
122.098
120.313
117.895
115.486
57.323
57.105
56.892
34.384
30.261
180
170
160
150
140
130
120
110
100
90
80
70
60
50
40
30
20
10
0
f1 (ppm)

$^{19}$F NMR

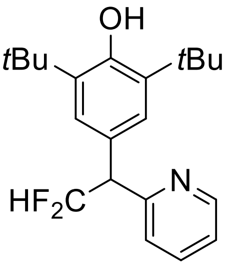


**2n**

(376 MHz, $CDCl_3$)

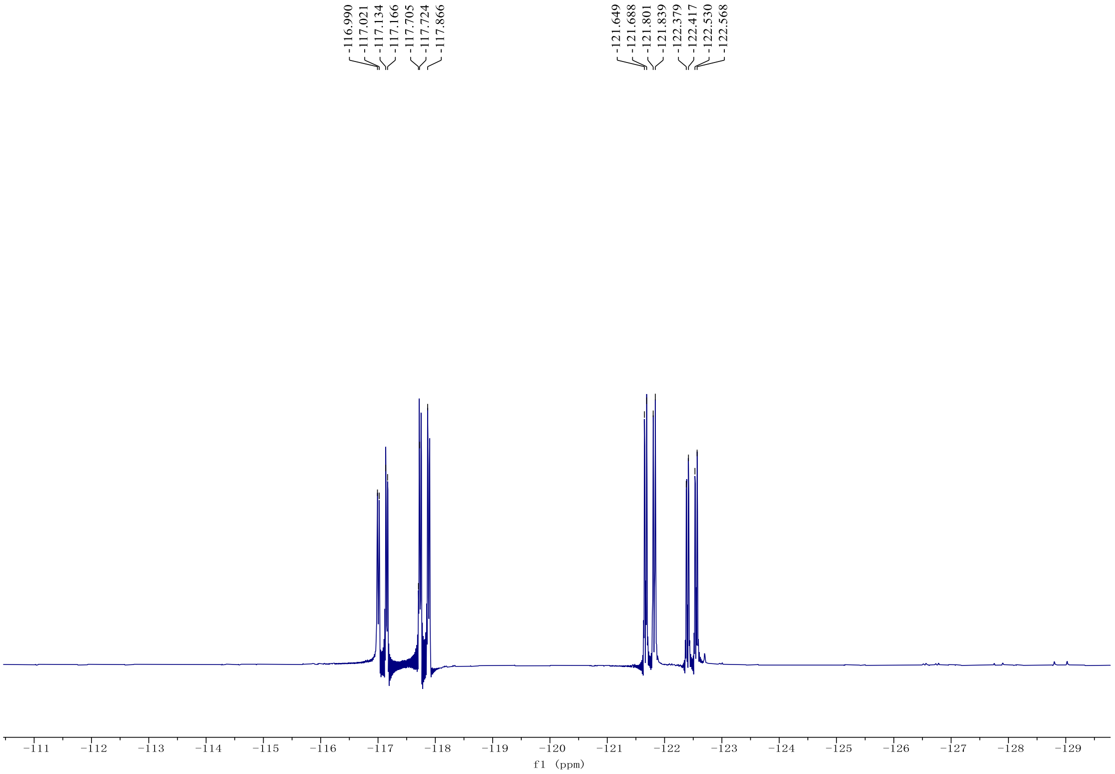

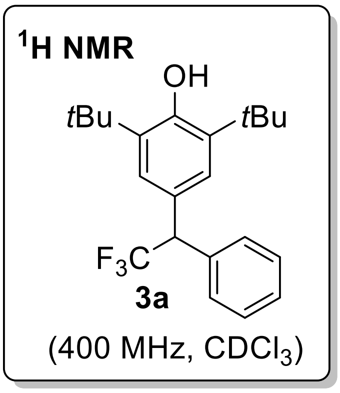

1H NMR
OH
tBu
tBu
F3C
3a
(400 MHz, CDCl3)


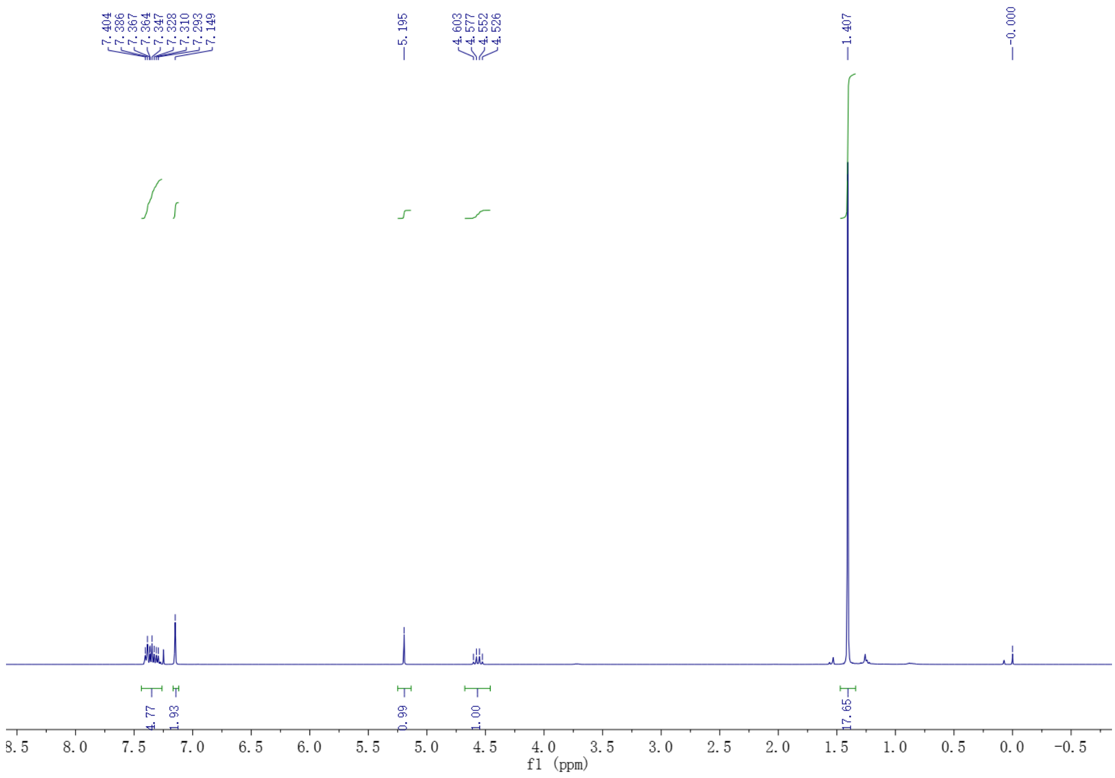

7.404
7.386
7.367
7.364
7.347
7.328
7.310
7.293
7.149
5.195
4.603
4.577
4.552
4.526
1.407
-0.000
4.77
1.93
0.99
1.00
17.65
8.5
8.0
7.5
7.0
6.5
6.0
5.5
5.0
4.5
4.0
3.5
3.0
2.5
2.0
1.5
1.0
0.5
0.0
-0.5
f1 (ppm)

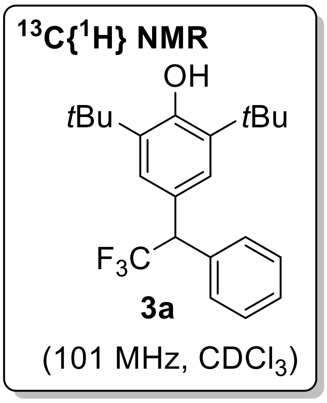

13C{1H} NMR
OH
tBu
tBu
F3C
3a
(101 MHz, CDCl3)


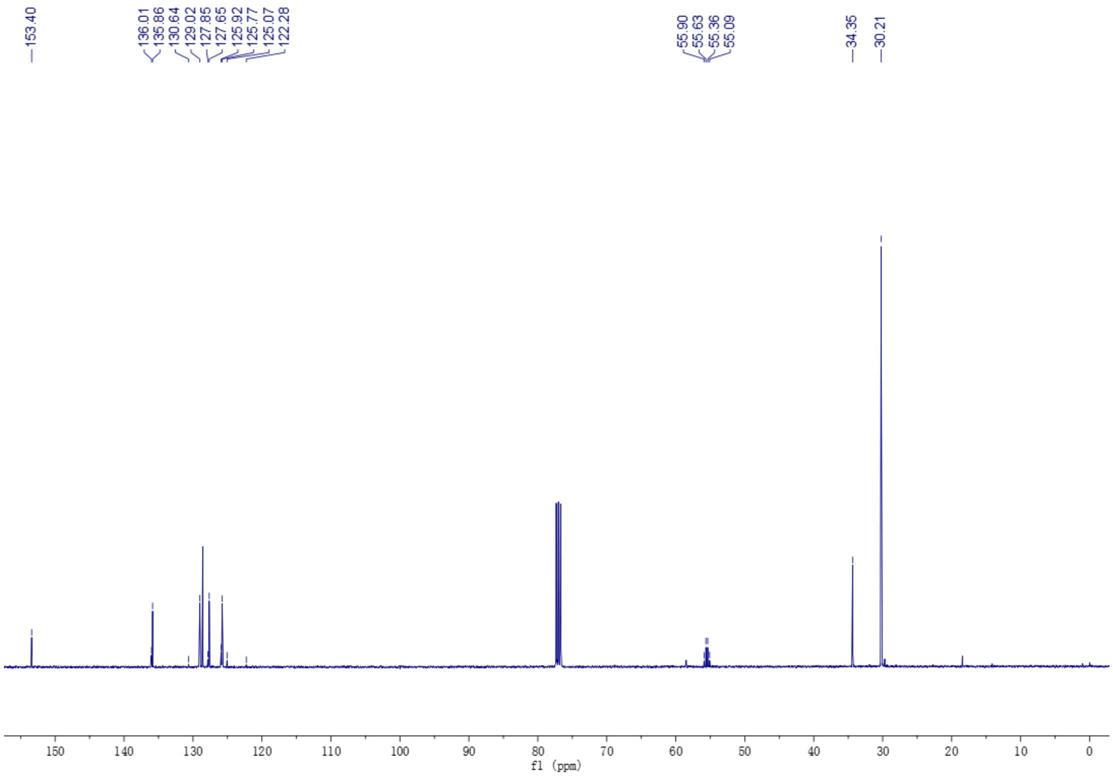

153.40
136.01
135.86
130.64
129.02
127.85
127.65
125.92
125.77
125.07
122.28
55.90
55.63
55.36
55.09
34.35
30.21
150
140
130
120
110
100
90
80
70
60
50
40
30
20
10
0
f1 (ppm)

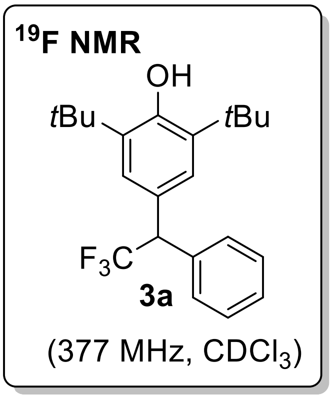

19F NMR
OH
tBu
tBu
F3C
3a
(377 MHz, CDCl3)


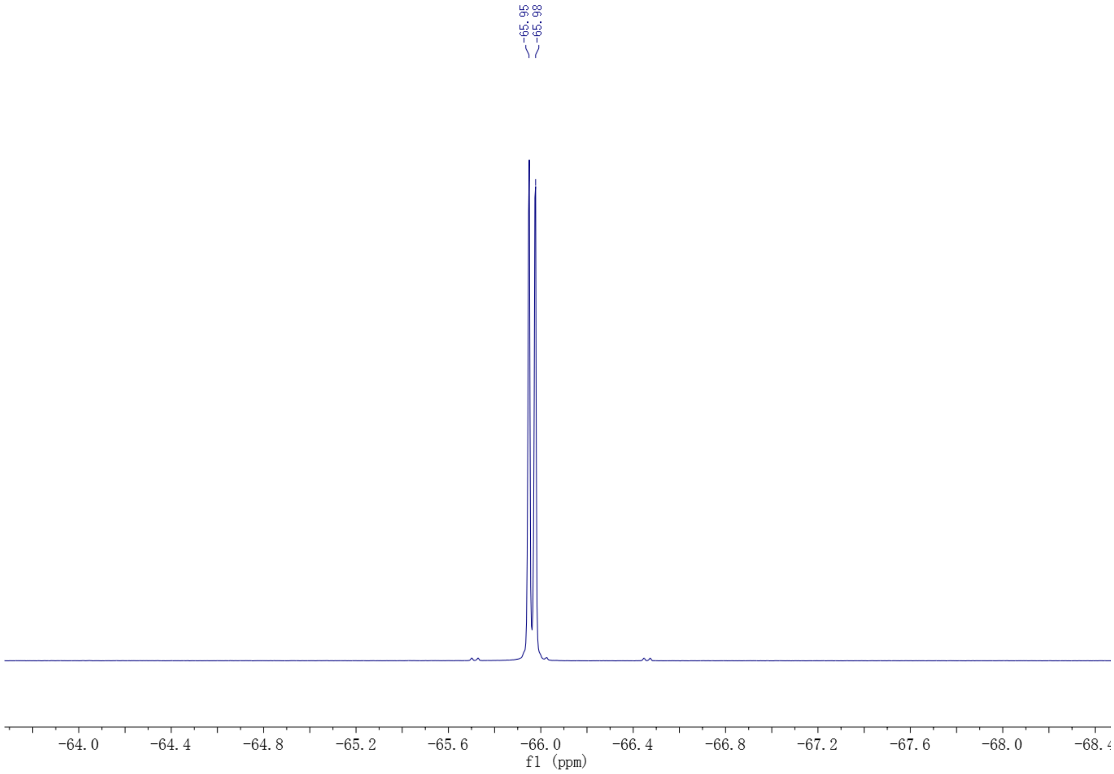

-65.95
-65.98
-64.0
-64.4
-64.8
-65.2
-65.6
-66.0
-66.4
-66.8
-67.2
-67.6
-68.0
f1 (ppm)

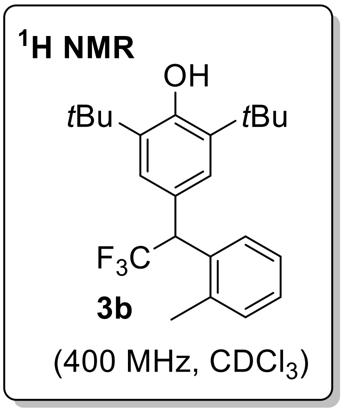

1H NMR
OH
tBu
tBu
F3C
3b
(400 MHz, CDCl3)


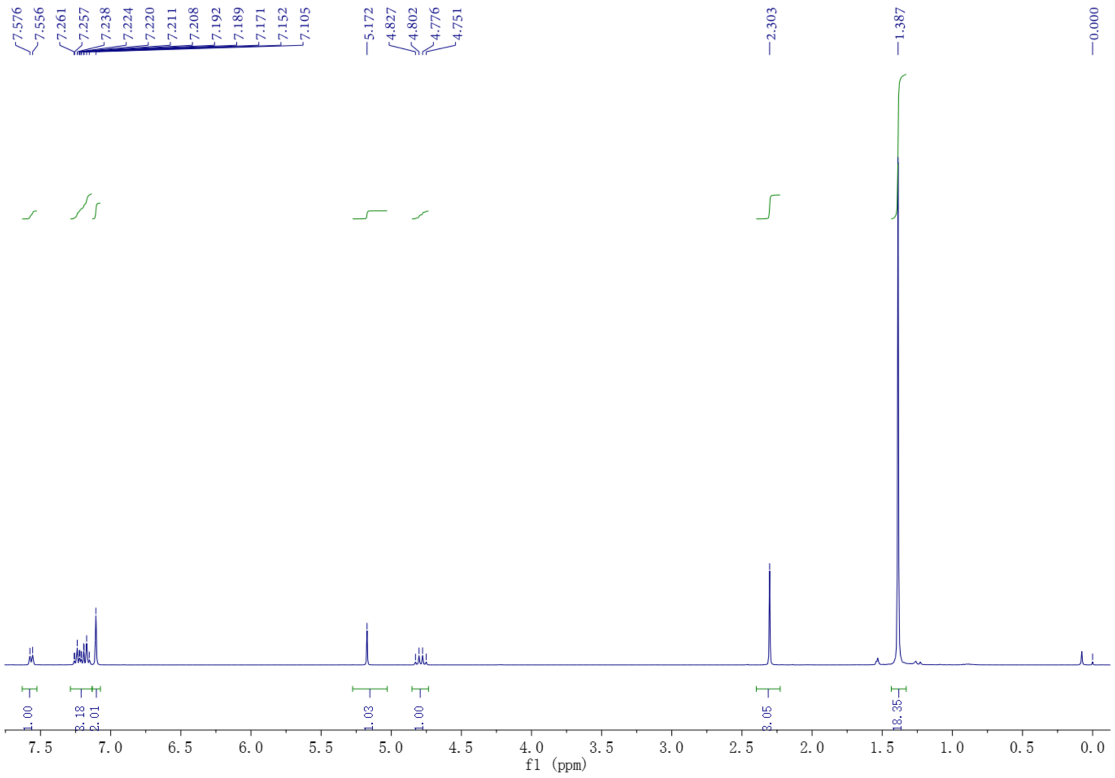

7.576
7.556
7.261
7.257
7.238
7.224
7.220
7.211
7.208
7.192
7.189
7.171
7.152
7.105
5.172
4.827
4.802
4.776
4.751
2.303
1.387
0.000
1.00
3.18
2.01
1.03
1.00
3.05
18.35
f1 (ppm)

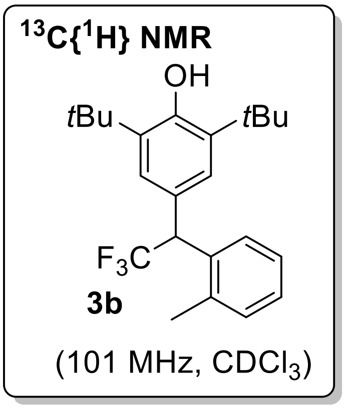
13C{1H} NMR
OH
tBu
tBu
F3C
3b
(101 MHz, CDCl3)

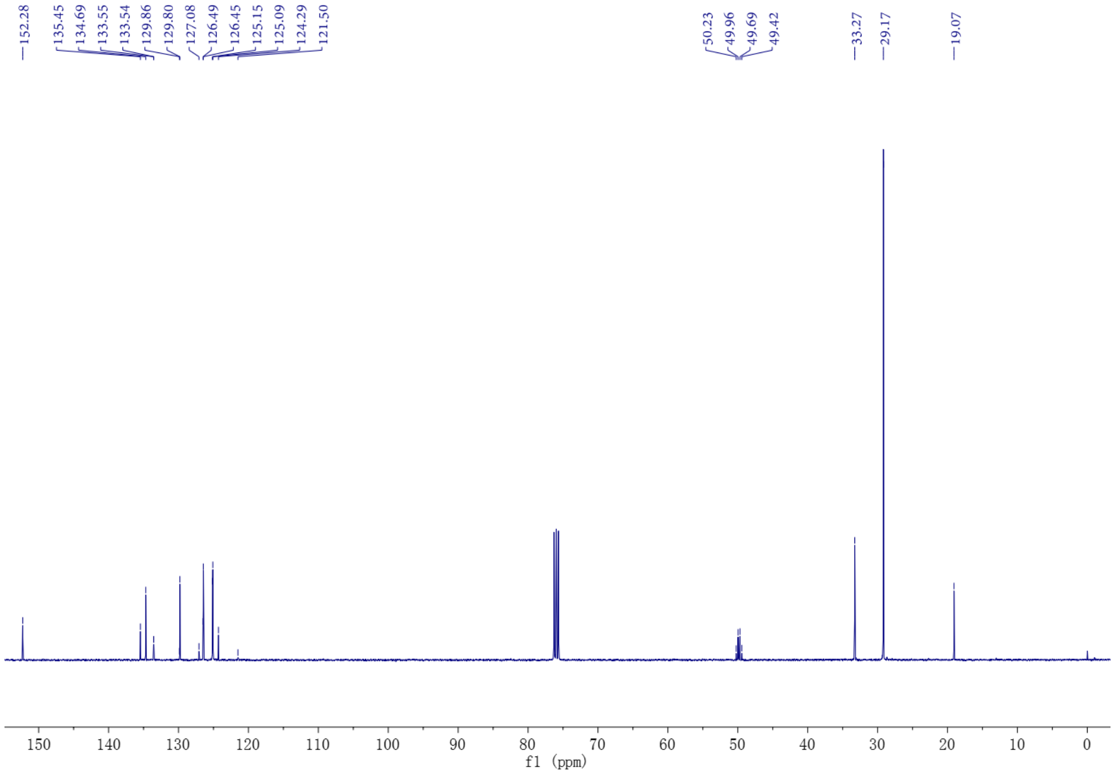
152.28
135.45
134.69
133.55
133.54
129.86
129.80
127.08
126.49
126.45
125.15
125.09
124.29
121.50
50.23
49.96
49.69
49.42
33.27
29.17
19.07
150
140
130
120
110
100
90
80
70
60
50
40
30
20
10
0
f1 (ppm)

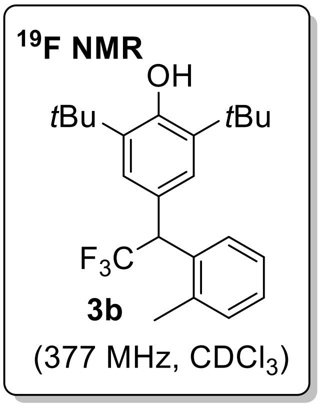

19F NMR
OH
tBu
tBu
F3C
3b
(377 MHz, CDCl3)


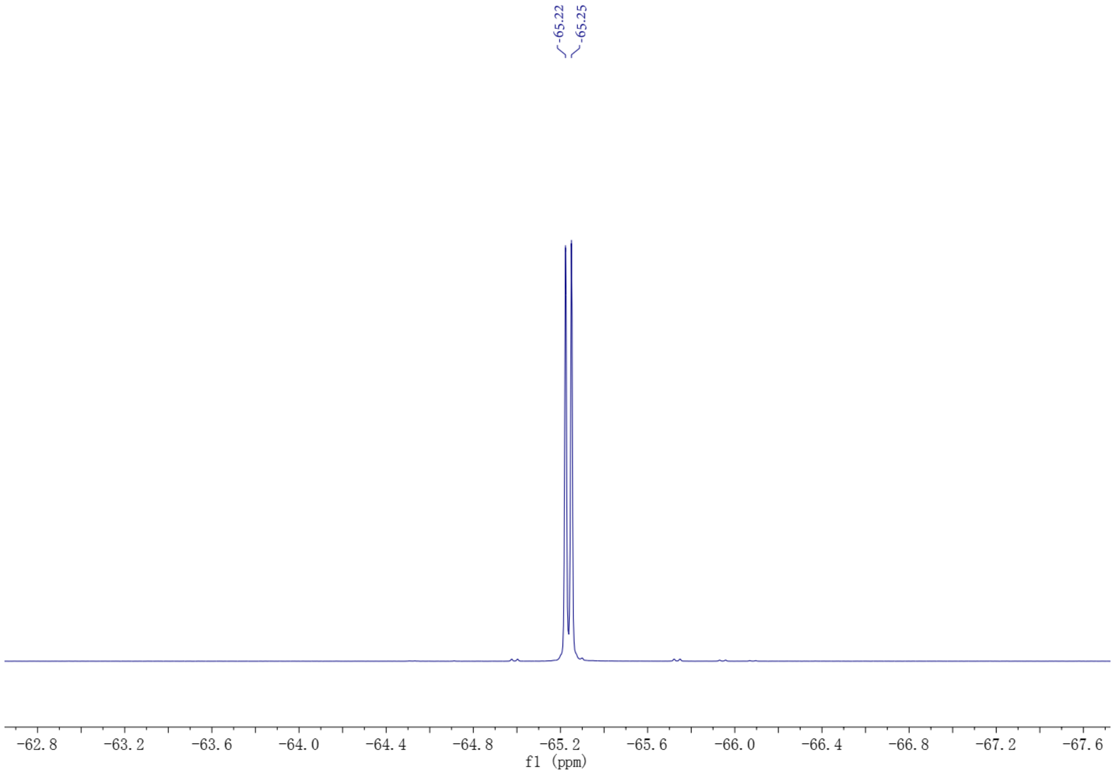

-65.22
-65.25
-62.8
-63.2
-63.6
-64.0
-64.4
-64.8
-65.2
-65.6
-66.0
-66.4
-66.8
-67.2
-67.6
f1 (ppm)

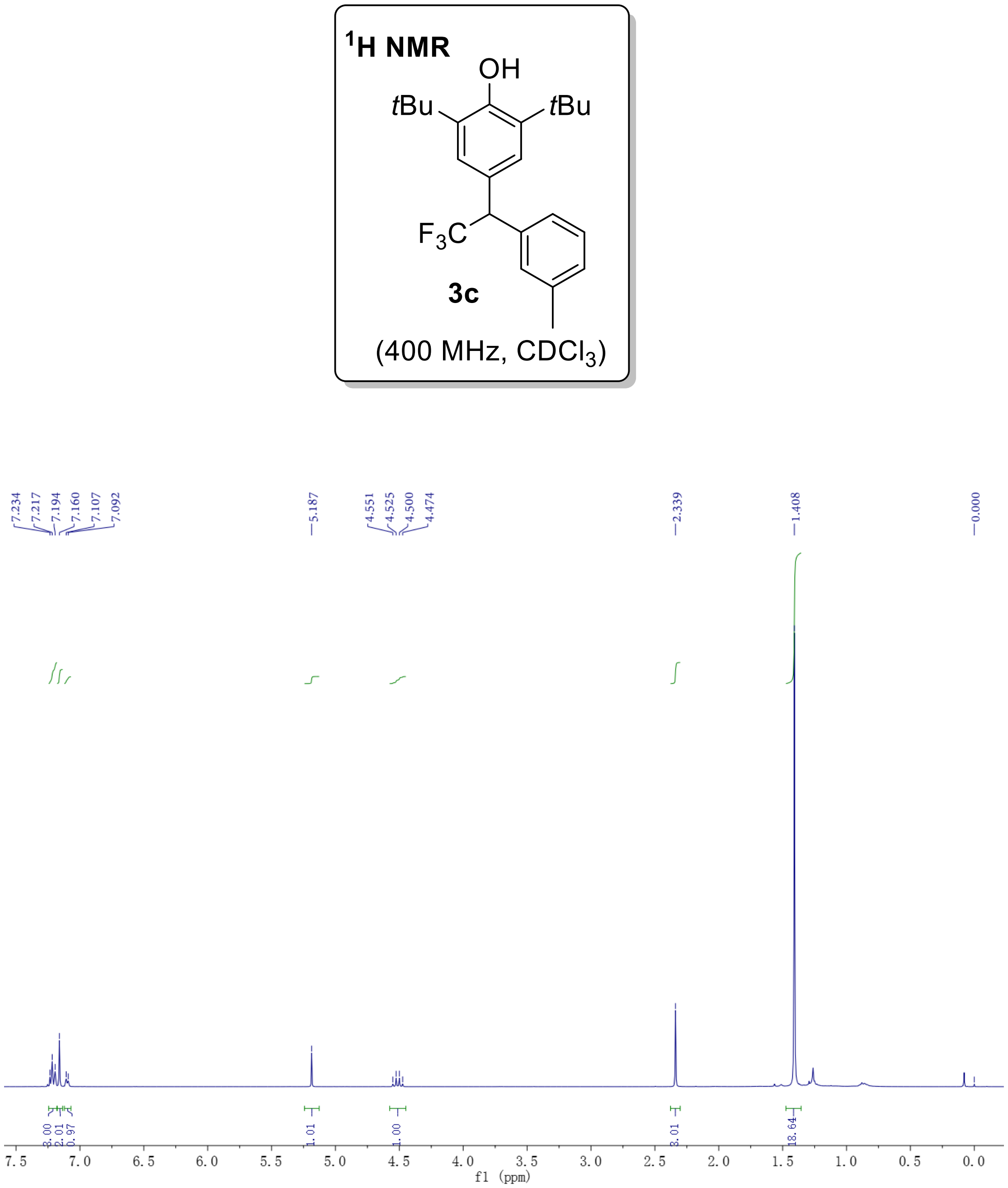

1H NMR
OH
tBu
tBu
F3C
3c
(400 MHz, CDCl3)
7.234
7.217
7.194
7.160
7.107
7.092
5.187
4.551
4.525
4.500
4.474
2.339
1.408
0.000
3.00
2.01
0.97
1.01
1.00
3.01
18.64
f1 (ppm)

**$^{13}C\{^1H\}$ NMR**

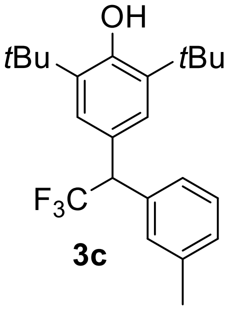


**3c**

(101 MHz, $CDCl_3$)

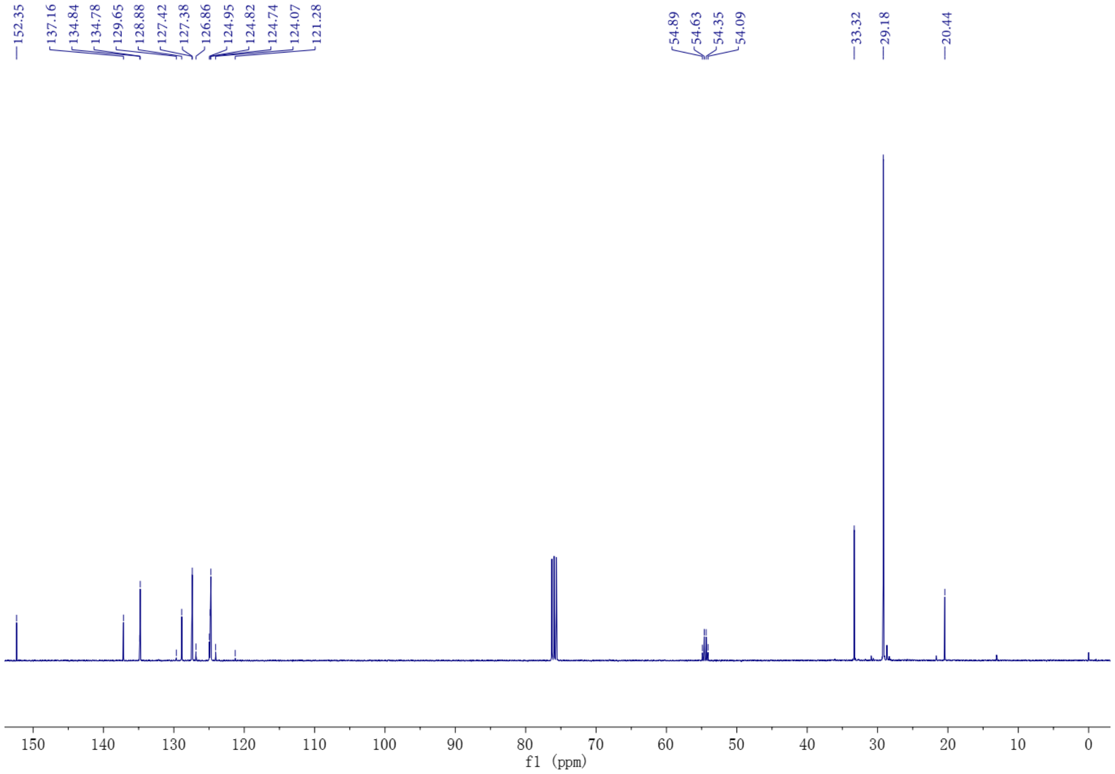

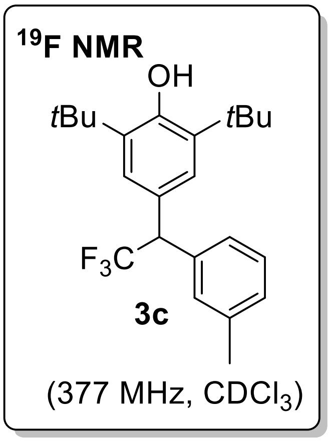

$^{19}$F NMR
OH
tBu
tBu
F3C
3c
(377 MHz, CDCl3)


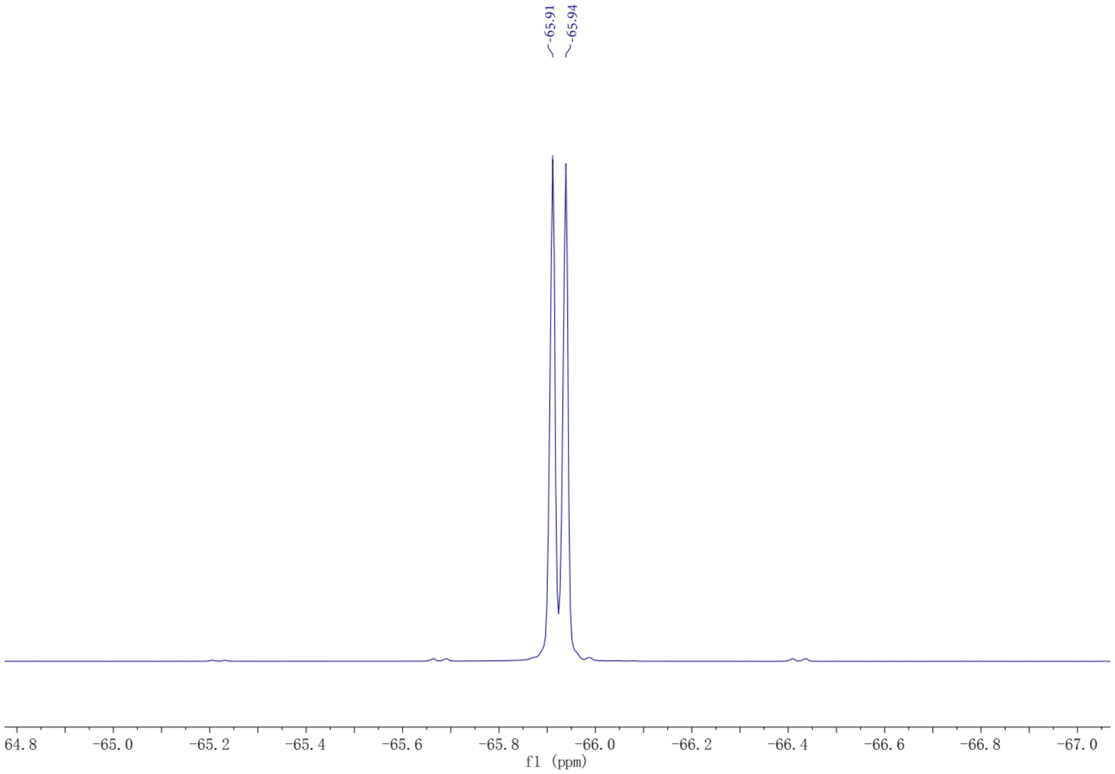

-65.91
-65.94
64.8
-65.0
-65.2
-65.4
-65.6
-65.8
-66.0
-66.2
-66.4
-66.6
-66.8
-67.0
f1 (ppm)

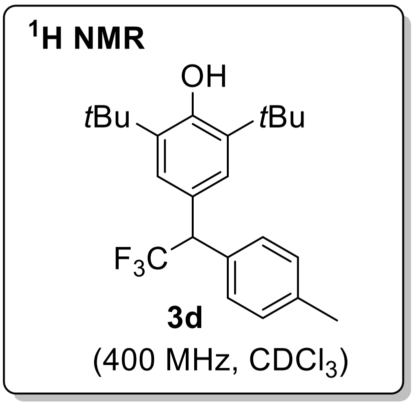

1H NMR
OH
tBu
tBu
F3C
3d
(400 MHz, CDCl3)


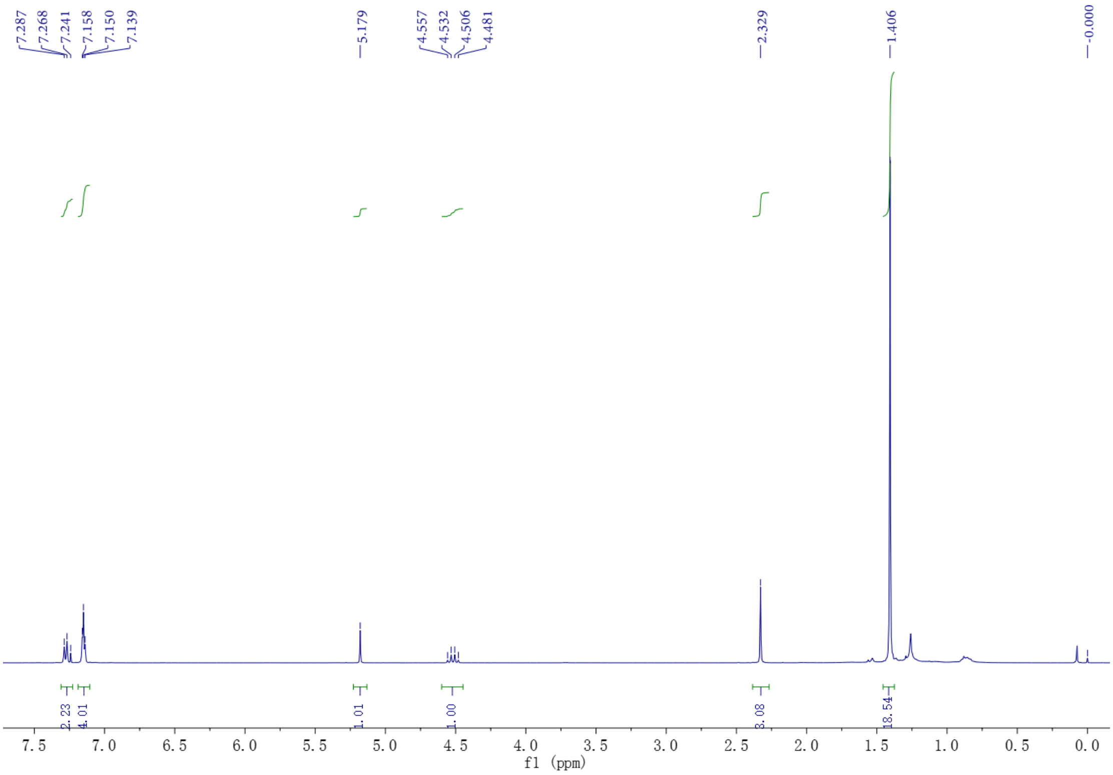

7.287
7.268
7.241
7.158
7.150
7.139
5.179
4.557
4.532
4.506
4.481
2.329
1.406
-0.000
2.23
4.01
1.01
1.00
3.08
18.54
7.5
7.0
6.5
6.0
5.5
5.0
4.5
4.0
3.5
3.0
2.5
2.0
1.5
1.0
0.5
0.0
f1 (ppm)

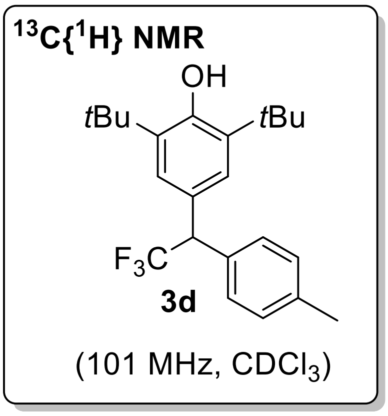

13C{1H} NMR
OH
tBu
tBu
F3C
3d
(101 MHz, CDCl3)


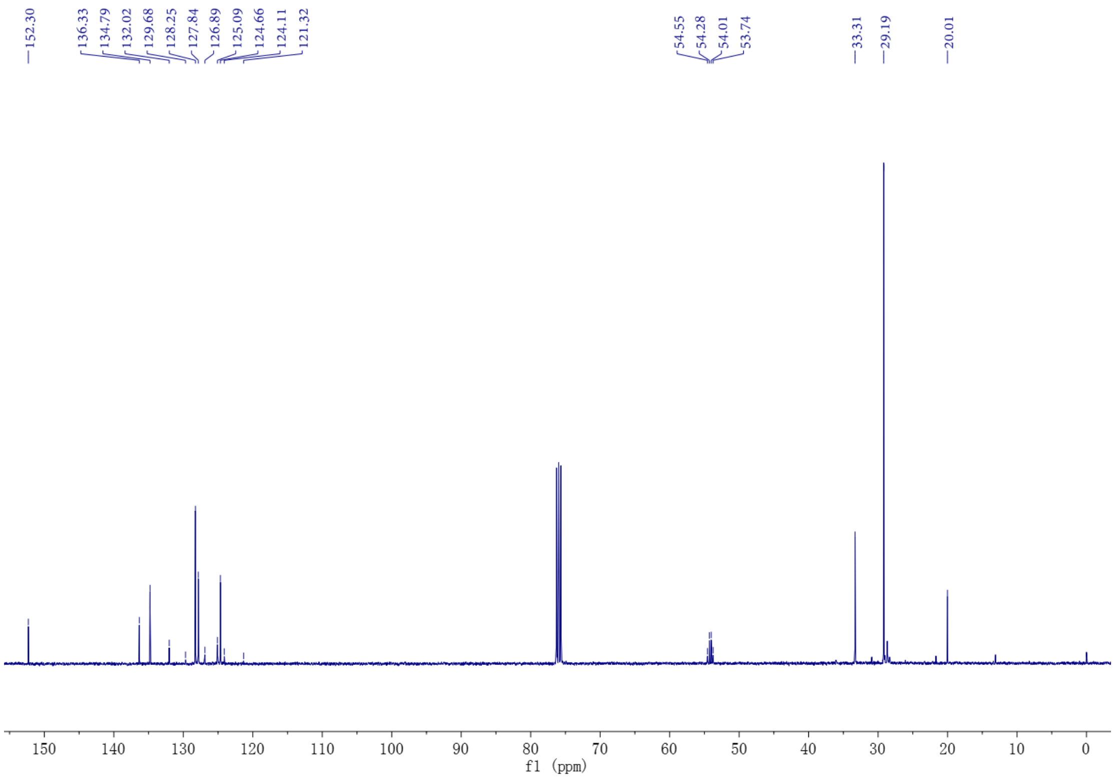

152.30
136.33
134.79
132.02
129.68
128.25
127.84
126.89
125.09
124.66
124.11
121.32
54.55
54.28
54.01
53.74
33.31
29.19
20.01
150
140
130
120
110
100
90
80
70
60
50
40
30
20
10
0
f1 (ppm)

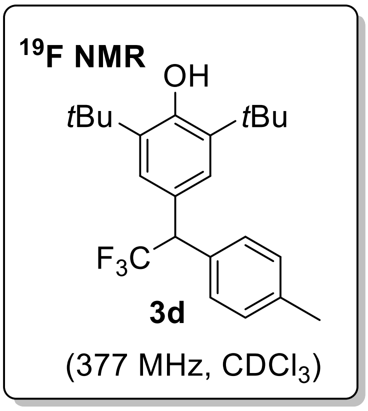

19F NMR
OH
tBu
tBu
F3C
3d
(377 MHz, CDCl3)


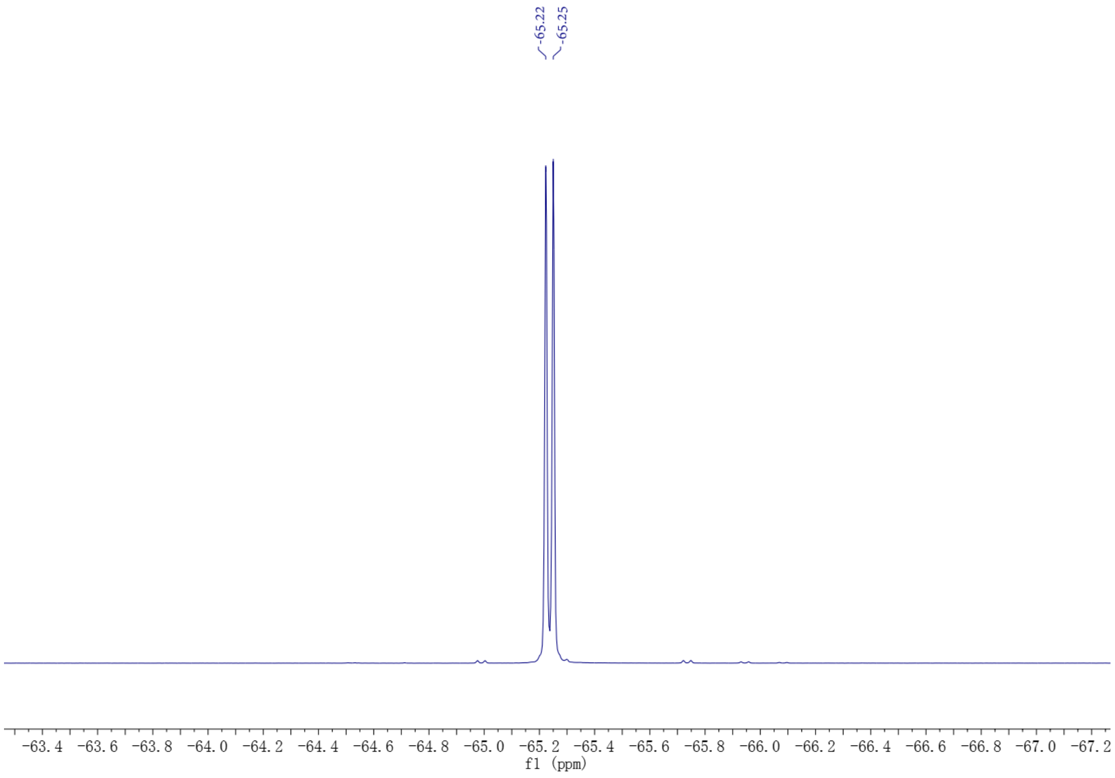

-65.22
-65.25
-63.4
-63.6
-63.8
-64.0
-64.2
-64.4
-64.6
-64.8
-65.0
-65.2
-65.4
-65.6
-65.8
-66.0
-66.2
-66.4
-66.6
-66.8
-67.0
-67.2
f1 (ppm)

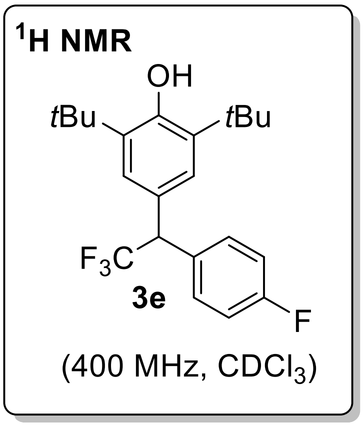

$^1$H NMR
OH
tBu
tBu
$F_3C$
3e
F
(400 MHz, $CDCl_3$)


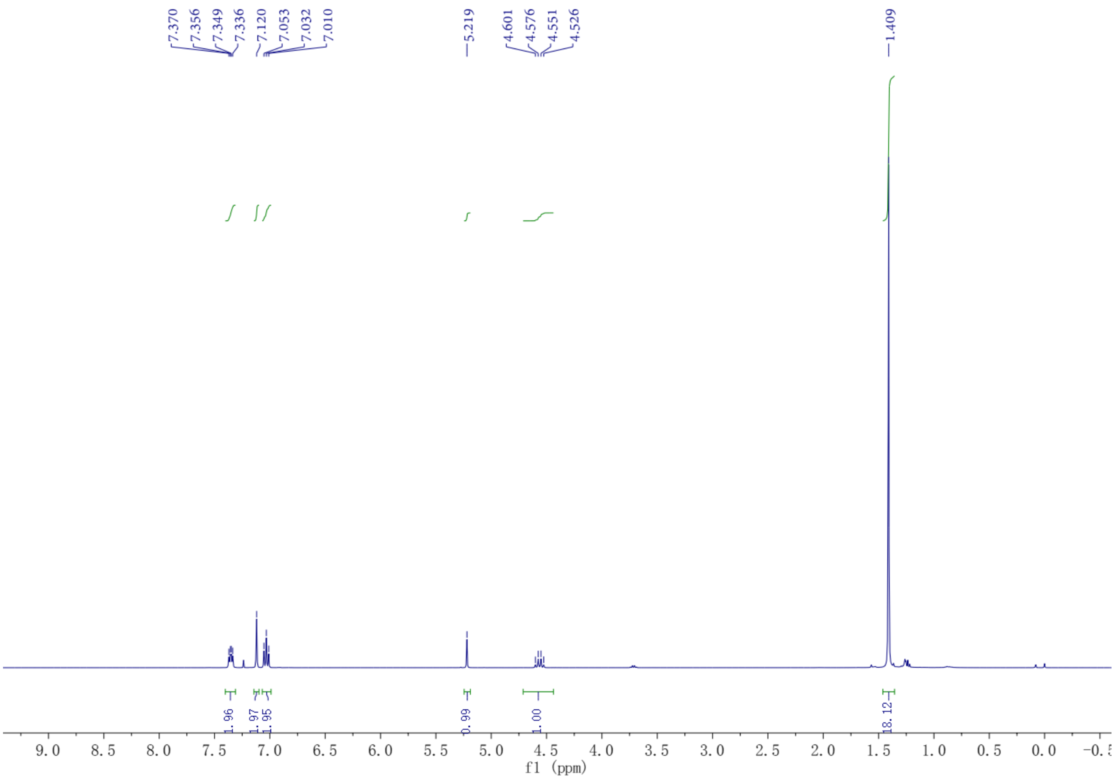

7.370
7.356
7.349
7.336
7.120
7.053
7.032
7.010
5.219
4.601
4.576
4.551
4.526
1.409
1.96
1.97
1.95
0.99
1.00
18.12
9.0 8.5 8.0 7.5 7.0 6.5 6.0 5.5 5.0 4.5 4.0 3.5 3.0 2.5 2.0 1.5 1.0 0.5 0.0 -0.5
f1 (ppm)

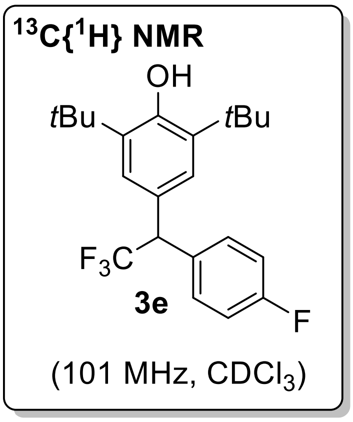

$^{13}C\{^1H\}$ NMR
OH
tBu
tBu
$F_3C$
F
3e
(101 MHz, $CDCl_3$)


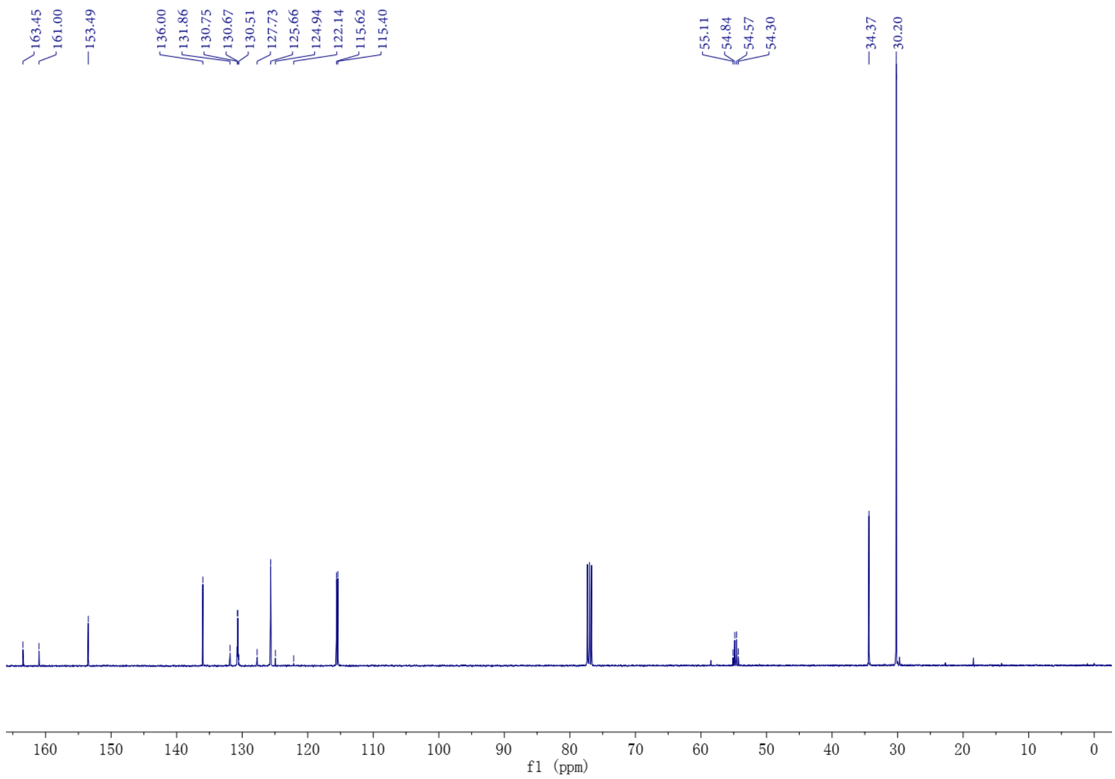

163.45
161.00
153.49
136.00
131.86
130.75
130.67
130.51
127.73
125.66
124.94
122.14
115.62
115.40
55.11
54.84
54.57
54.30
34.37
30.20
160
150
140
130
120
110
100
90
80
70
60
50
40
30
20
10
0
f1 (ppm)

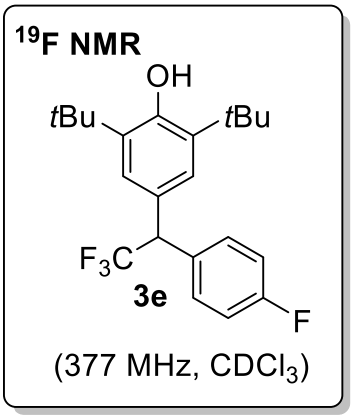

19F NMR
OH
tBu
tBu
F3C
3e
F
(377 MHz, CDCl3)


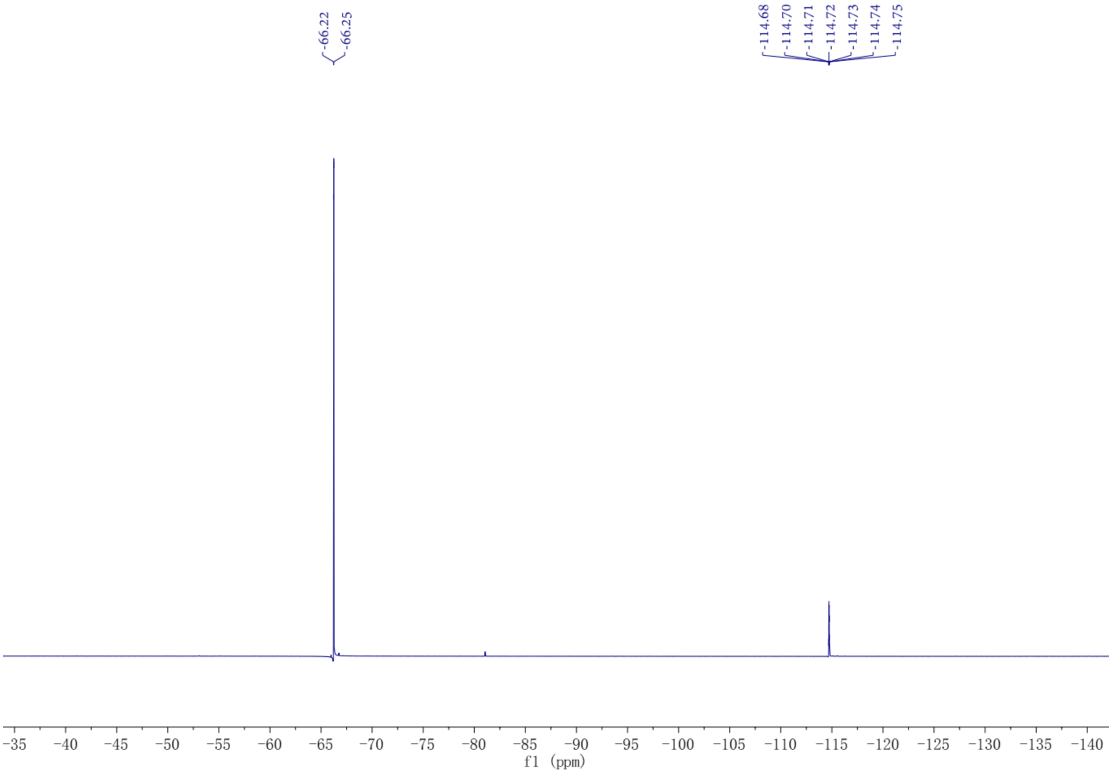

-66.22
-66.25
-114.68
-114.70
-114.71
-114.72
-114.73
-114.74
-114.75
−35 −40 −45 −50 −55 −60 −65 −70 −75 −80 −85 −90 −95 −100 −105 −110 −115 −120 −125 −130 −135 −140
f1 (ppm)

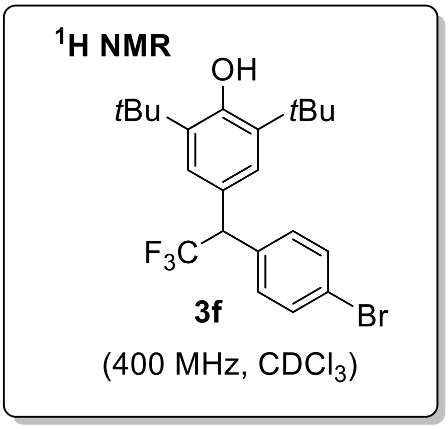

$^1$H NMR
OH
tBu
tBu
F3C
Br
3f
(400 MHz, CDCl3)


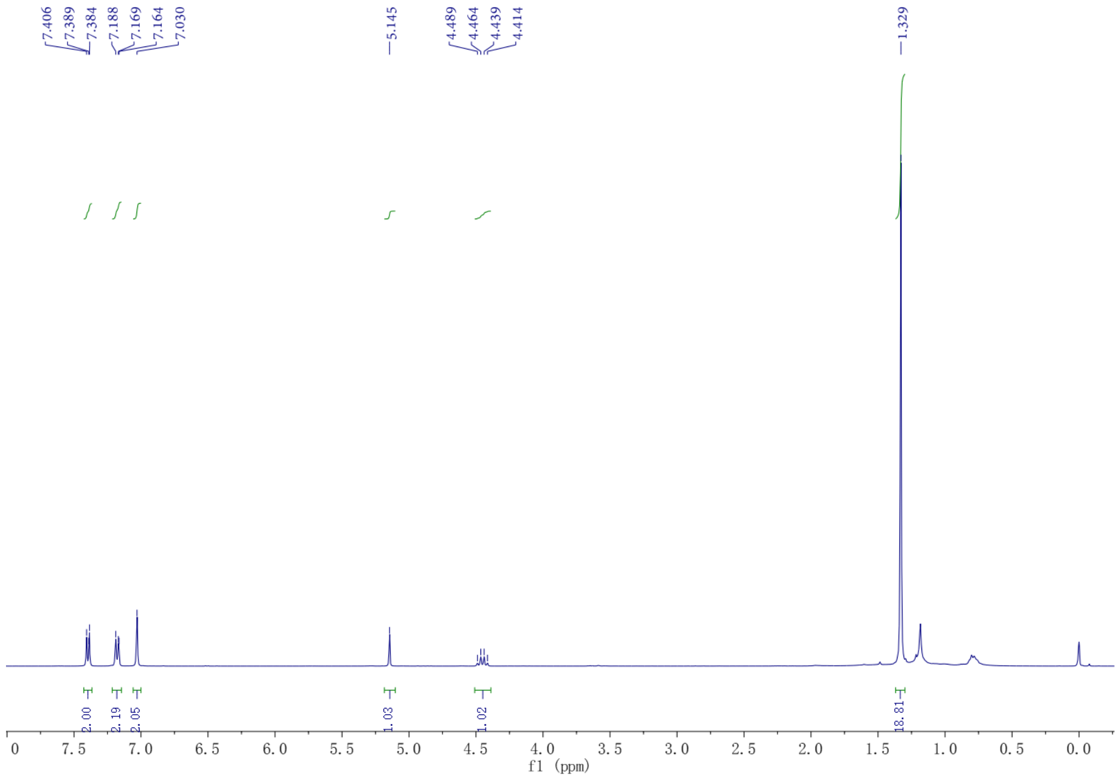

7.406
7.389
7.384
7.188
7.169
7.164
7.030
5.145
4.489
4.464
4.439
4.414
1.329
2.00
2.19
2.05
1.03
1.02
18.81
f1 (ppm)

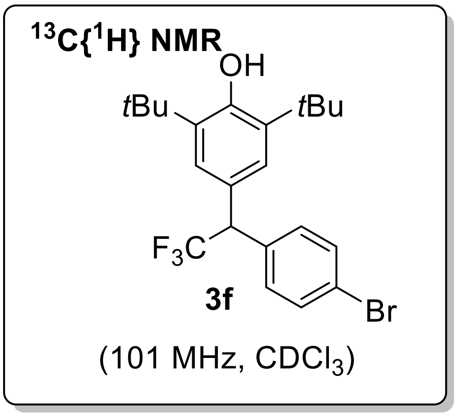

13C{1H} NMR
OH
tBu
tBu
F3C
3f
Br
(101 MHz, CDCl3)


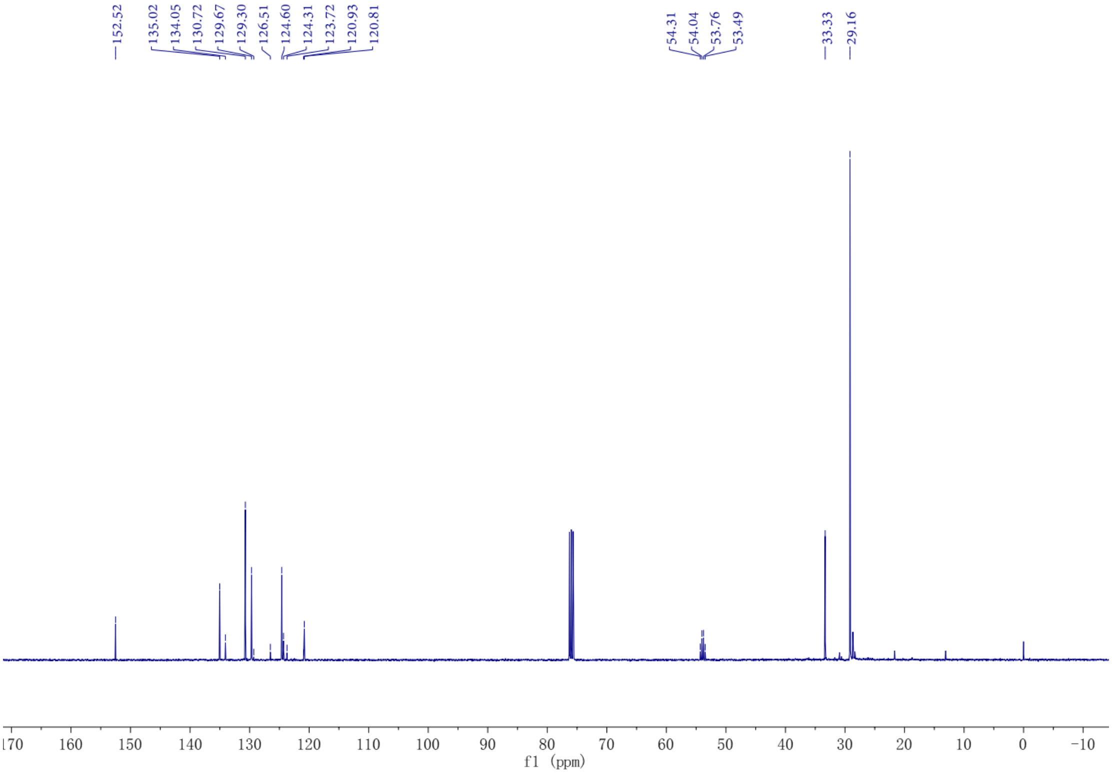

152.52
135.02
134.05
130.72
129.67
129.30
126.51
124.60
124.31
123.72
120.93
120.81
54.31
54.04
53.76
53.49
33.33
29.16
170
160
150
140
130
120
110
100
90
80
70
60
50
40
30
20
10
0
-10
f1 (ppm)

**$^{19}$F NMR**

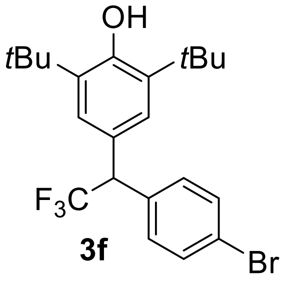


**3f**

(377 MHz, $CDCl_3$)

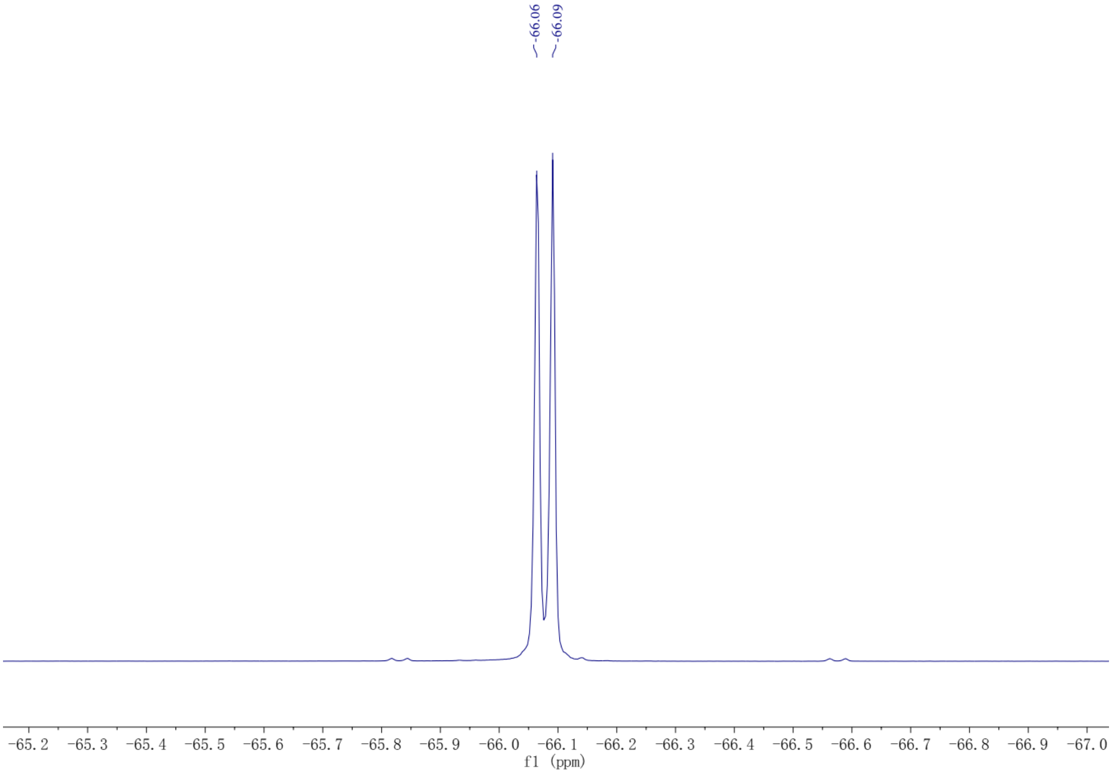

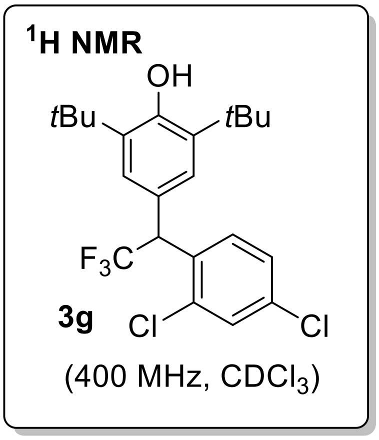

1H NMR
OH
tBu
tBu
F3C
3g
Cl
Cl
(400 MHz, CDCl3)


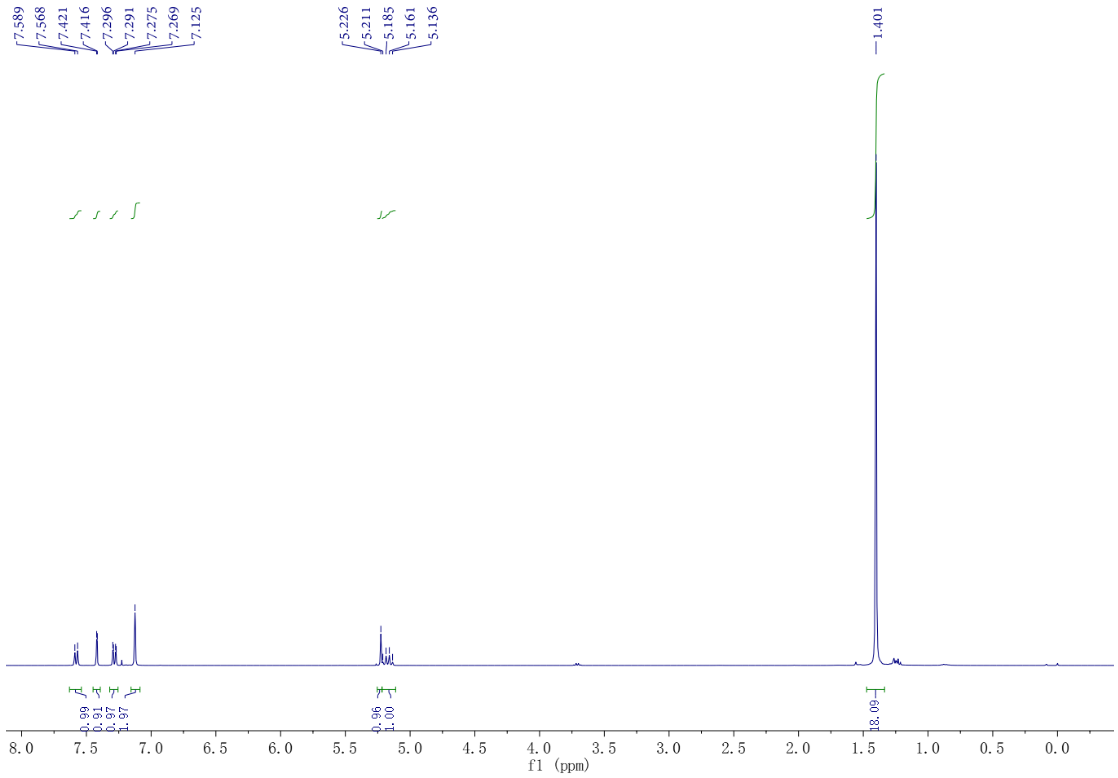

7.589
7.568
7.421
7.416
7.296
7.291
7.275
7.269
7.125
5.226
5.211
5.185
5.161
5.136
1.401
0.99
0.91
0.97
1.97
0.96
1.00
18.09
8.0
7.5
7.0
6.5
6.0
5.5
5.0
4.5
4.0
3.5
3.0
2.5
2.0
1.5
1.0
0.5
0.0
f1 (ppm)

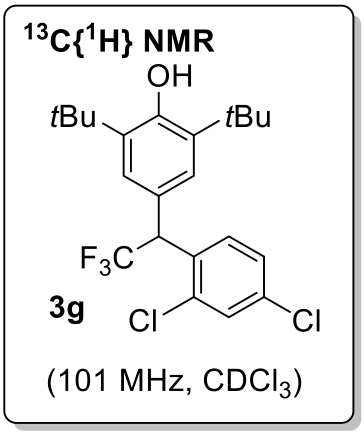

$^{13}C\{^1H\}$ NMR
OH
tBu
tBu
$F_3C$
3g
Cl
Cl
(101 MHz, $CDCl_3$)


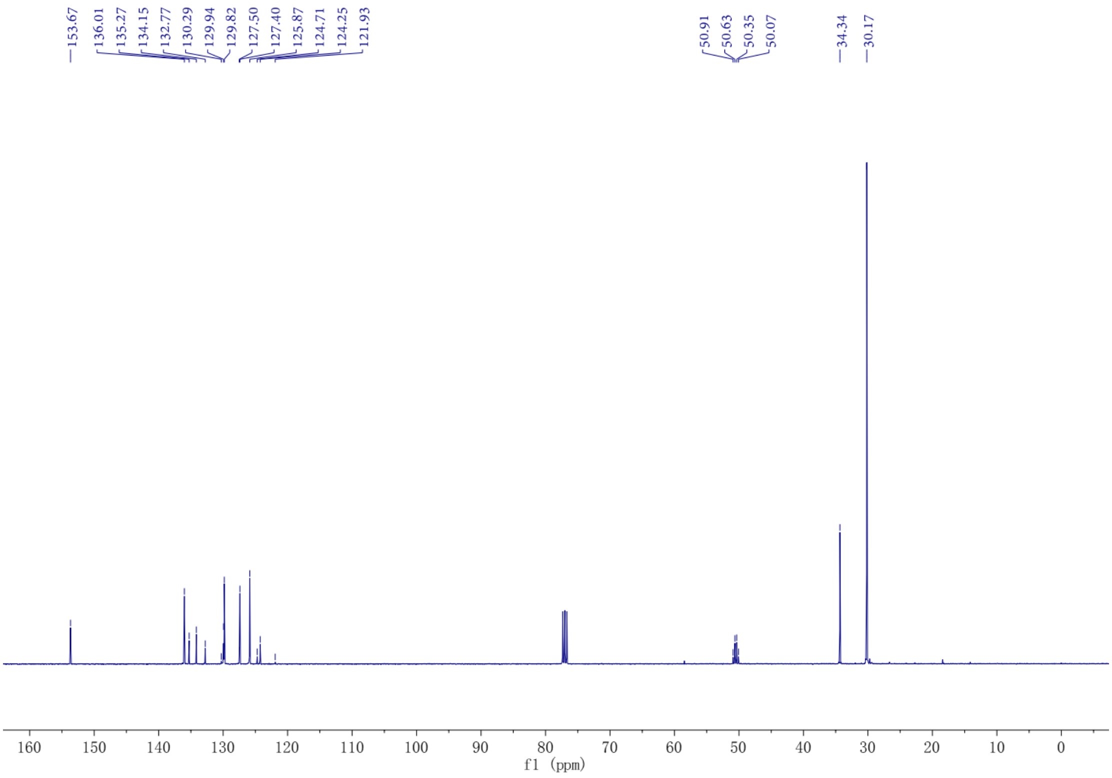

153.67
136.01
135.27
134.15
132.77
130.29
129.94
129.82
127.50
127.40
125.87
124.71
124.25
121.93
50.91
50.63
50.35
50.07
34.34
30.17
160
150
140
130
120
110
100
90
80
70
60
50
40
30
20
10
0
f1 (ppm)

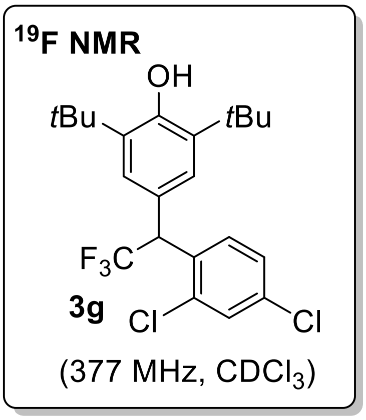

$^{19}$F NMR
OH
tBu
tBu
F3C
3g
Cl
Cl
(377 MHz, CDCl3)


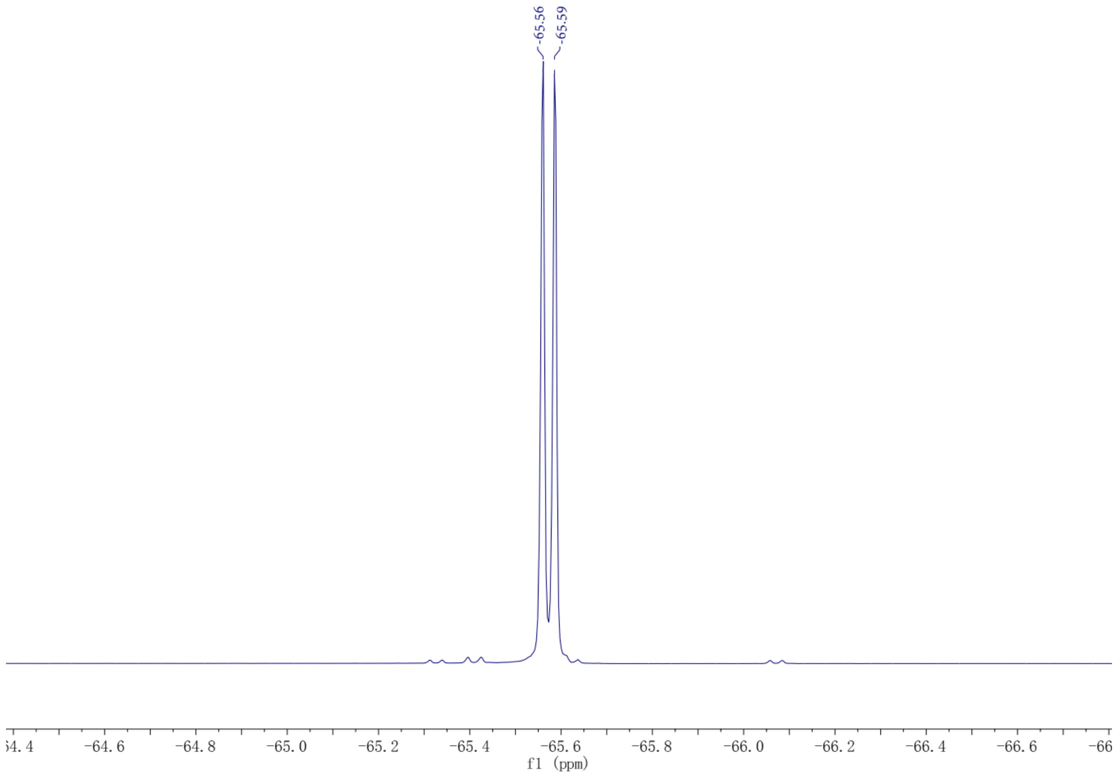

-65.56
-65.59
-64.6
-64.8
-65.0
-65.2
-65.4
-65.6
-65.8
-66.0
-66.2
-66.4
-66.6
f1 (ppm)

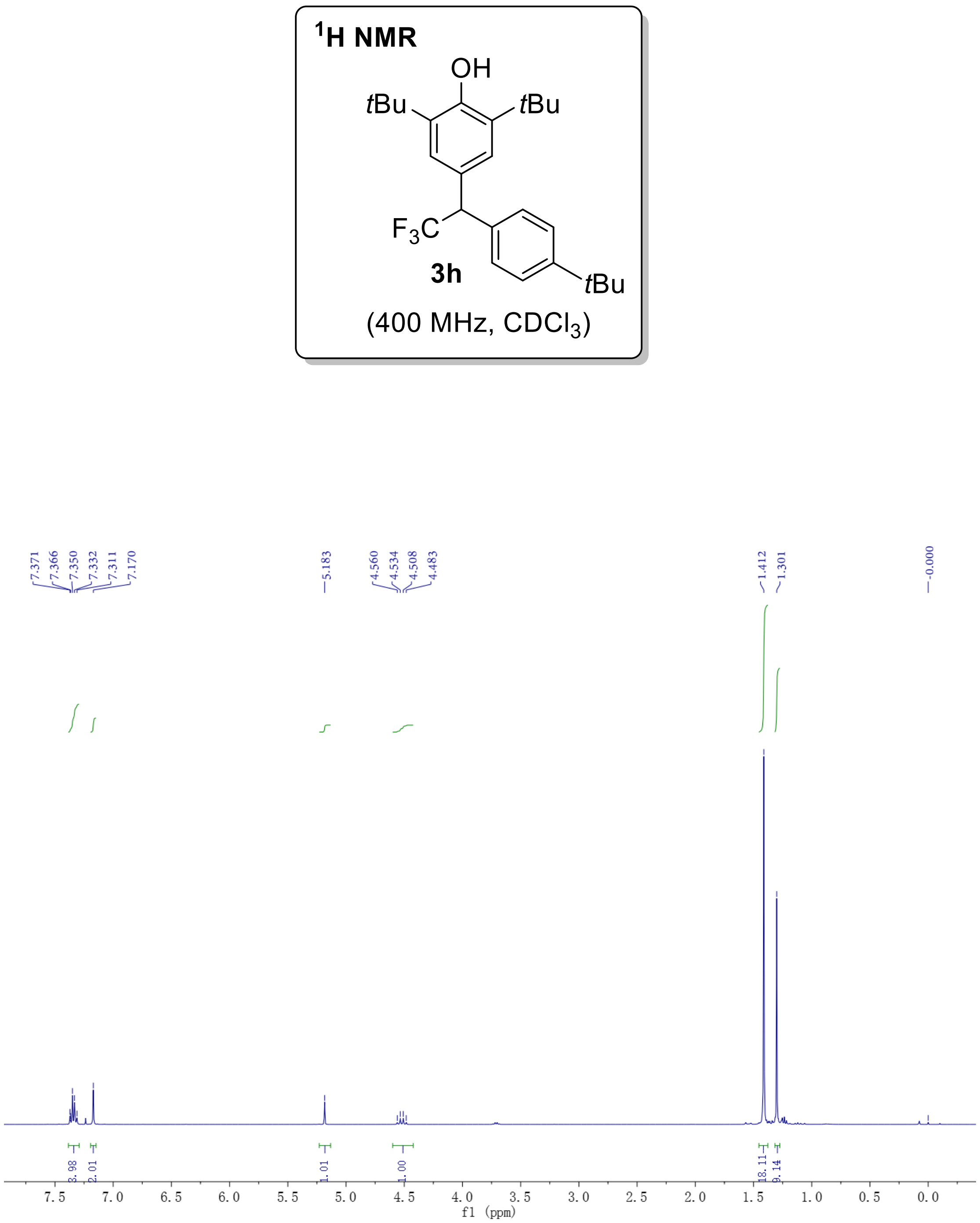

1H NMR
OH
tBu
tBu
F3C
3h
tBu
(400 MHz, CDCl3)
7.371
7.366
7.350
7.332
7.311
7.170
5.183
4.560
4.534
4.508
4.483
1.412
1.301
-0.000
3.98
2.01
1.01
1.00
18.11
9.14
7.5
7.0
6.5
6.0
5.5
5.0
4.5
4.0
3.5
3.0
2.5
2.0
1.5
1.0
0.5
0.0
f1 (ppm)

**$^{13}C\{^{1}H\}$ NMR**

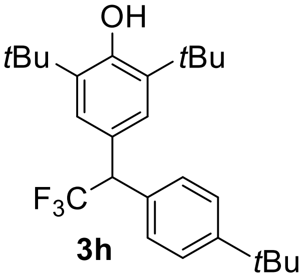


**3h**

(101 MHz, $CDCl_3$)

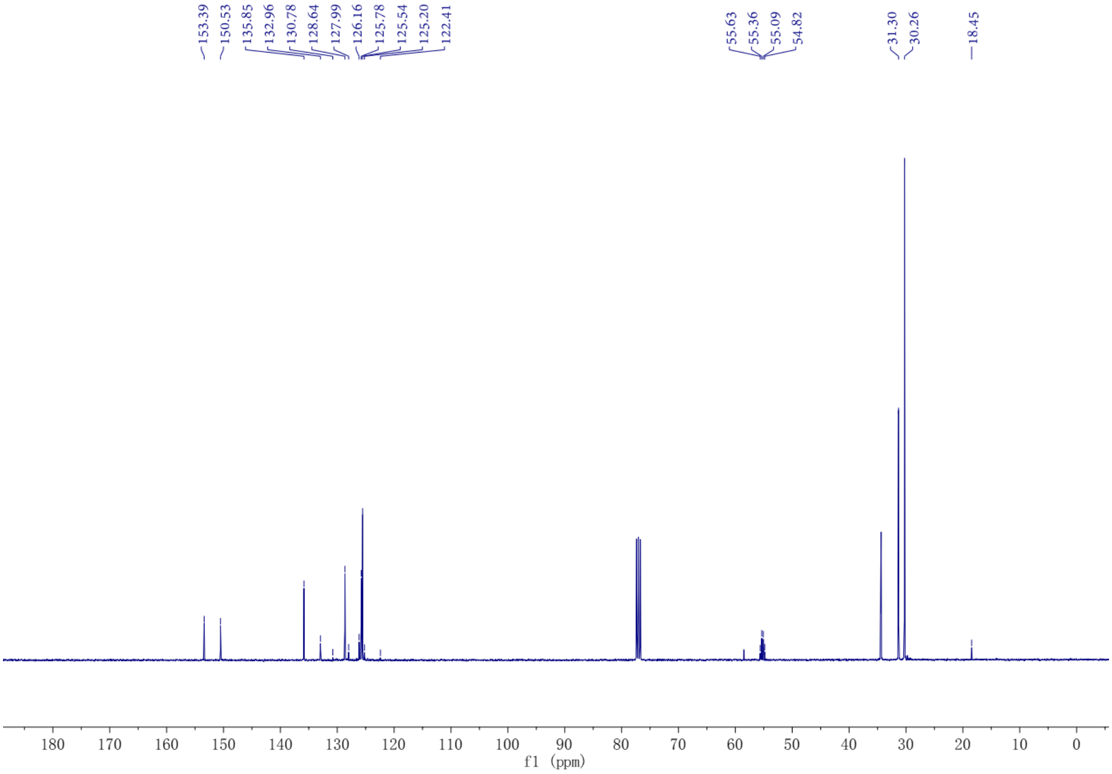

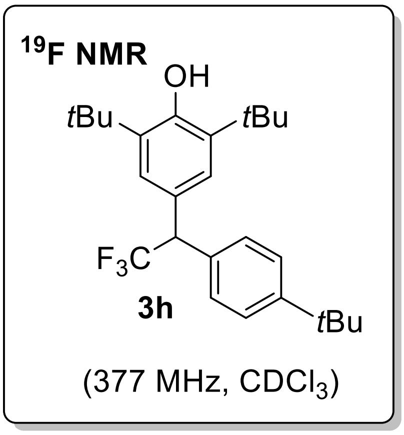

19F NMR
OH
tBu
tBu
F3C
3h
tBu
(377 MHz, CDCl3)


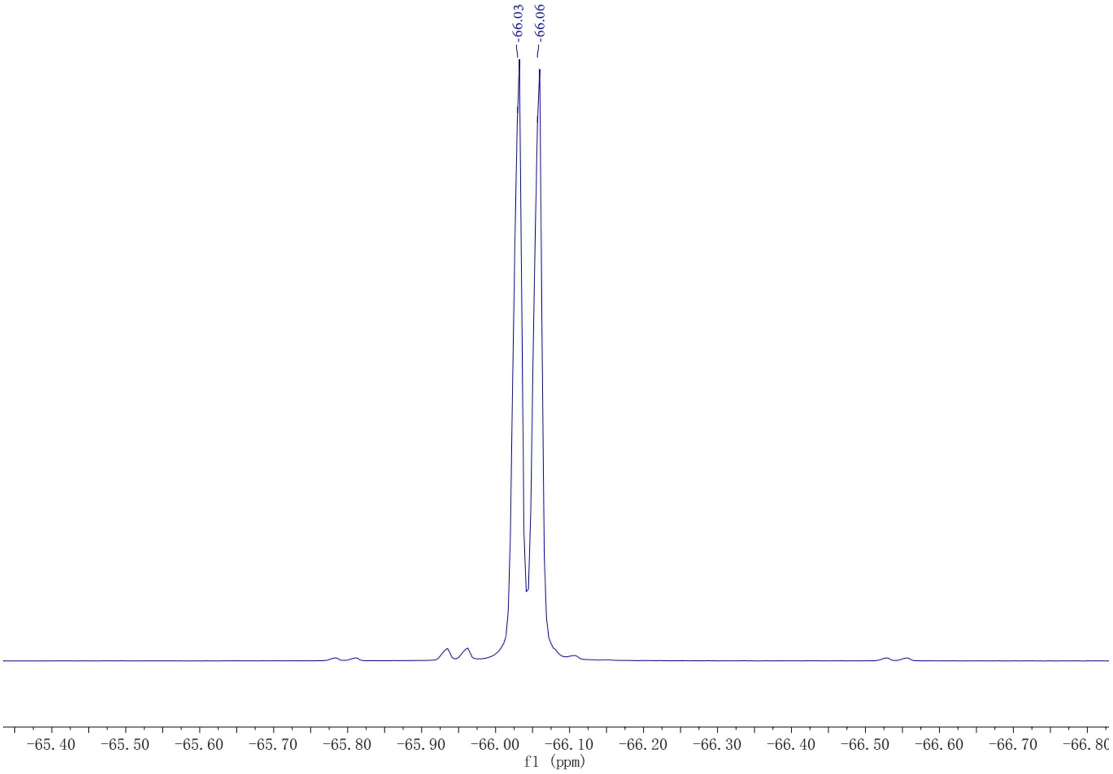

-66.03
-66.06
-65.40
-65.50
-65.60
-65.70
-65.80
-65.90
-66.00
-66.10
-66.20
-66.30
-66.40
-66.50
-66.60
-66.70
-66.80
f1 (ppm)

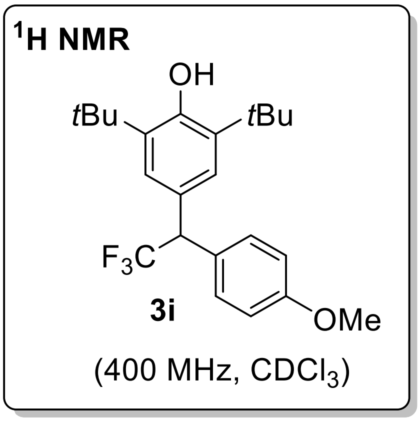

$^1$H NMR
OH
*t*Bu
*t*Bu
$F_3C$
3i
OMe
(400 MHz, $CDCl_3$)


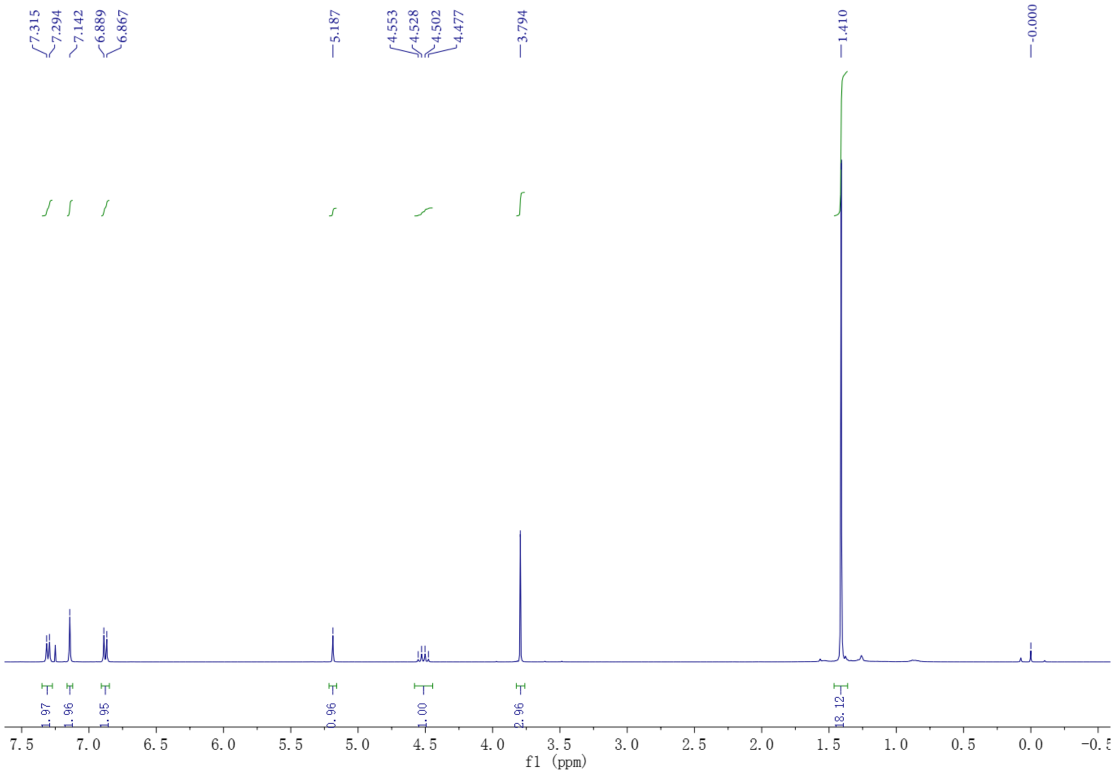

7.315
7.294
7.142
6.889
6.867
5.187
4.553
4.528
4.502
4.477
3.794
1.410
-0.000
1.97
1.96
1.95
0.96
1.00
2.96
18.12
f1 (ppm)

**$^{13}C\{^{1}H\}$ NMR**

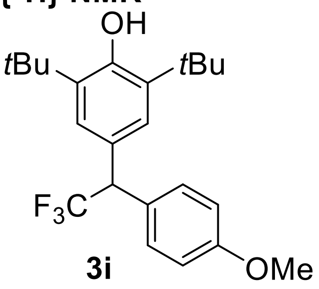


**3i**

(101 MHz, $CDCl_3$)

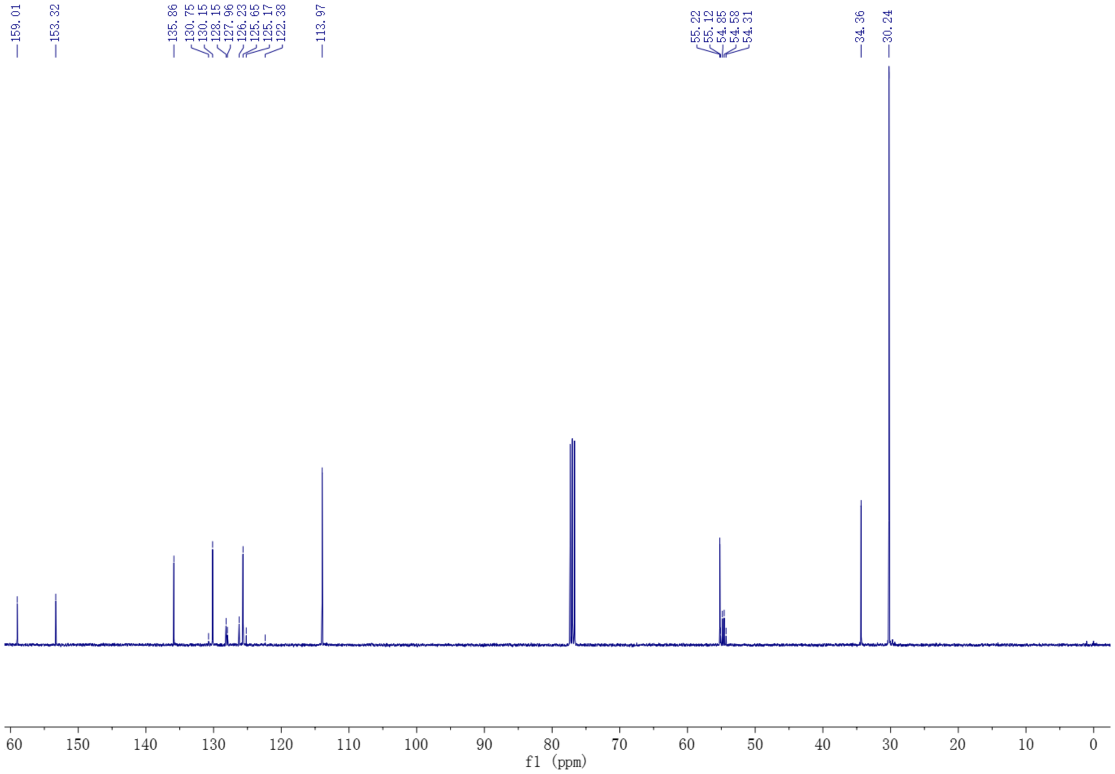

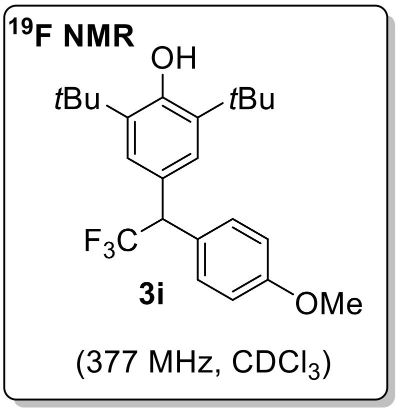

19F NMR
OH
tBu
tBu
F3C
3i
OMe
(377 MHz, CDCl3)


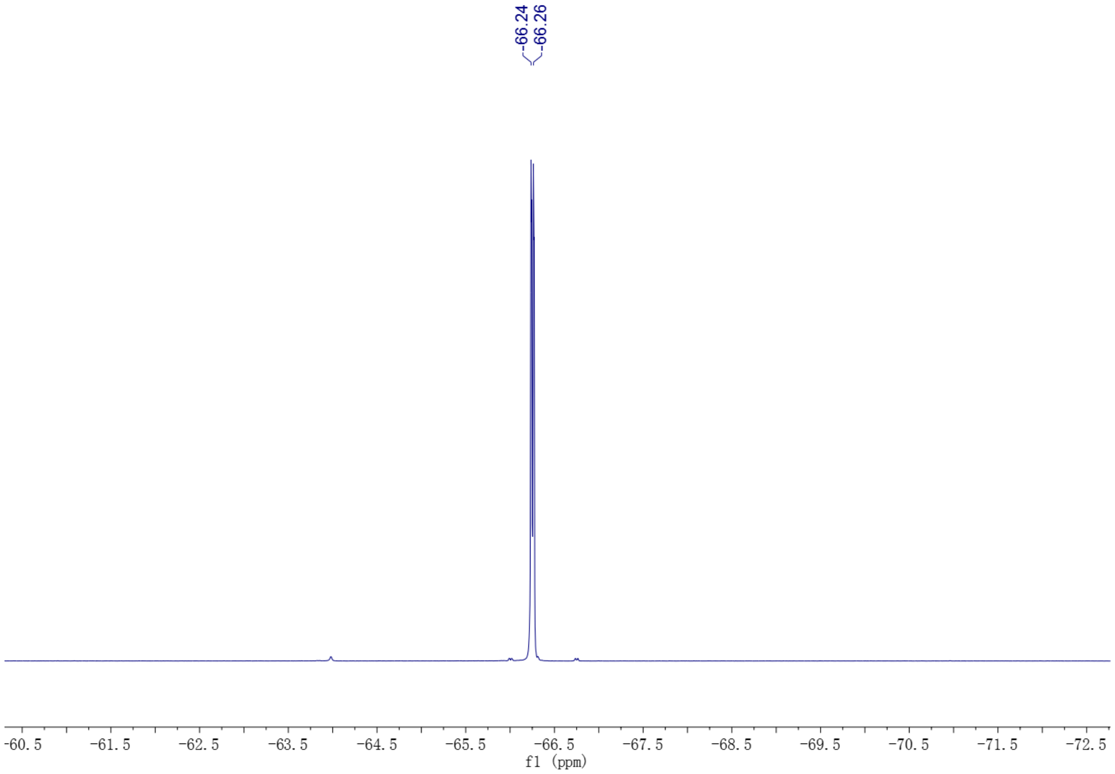

-66.24
-66.26
-60.5
-61.5
-62.5
-63.5
-64.5
-65.5
-66.5
-67.5
-68.5
-69.5
-70.5
-71.5
-72.5
f1 (ppm)

**$^1$H NMR**

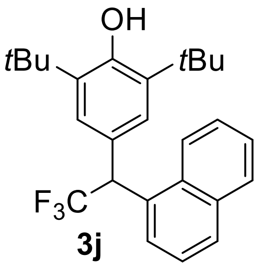


**3j**

(400 MHz, $CDCl_3$)

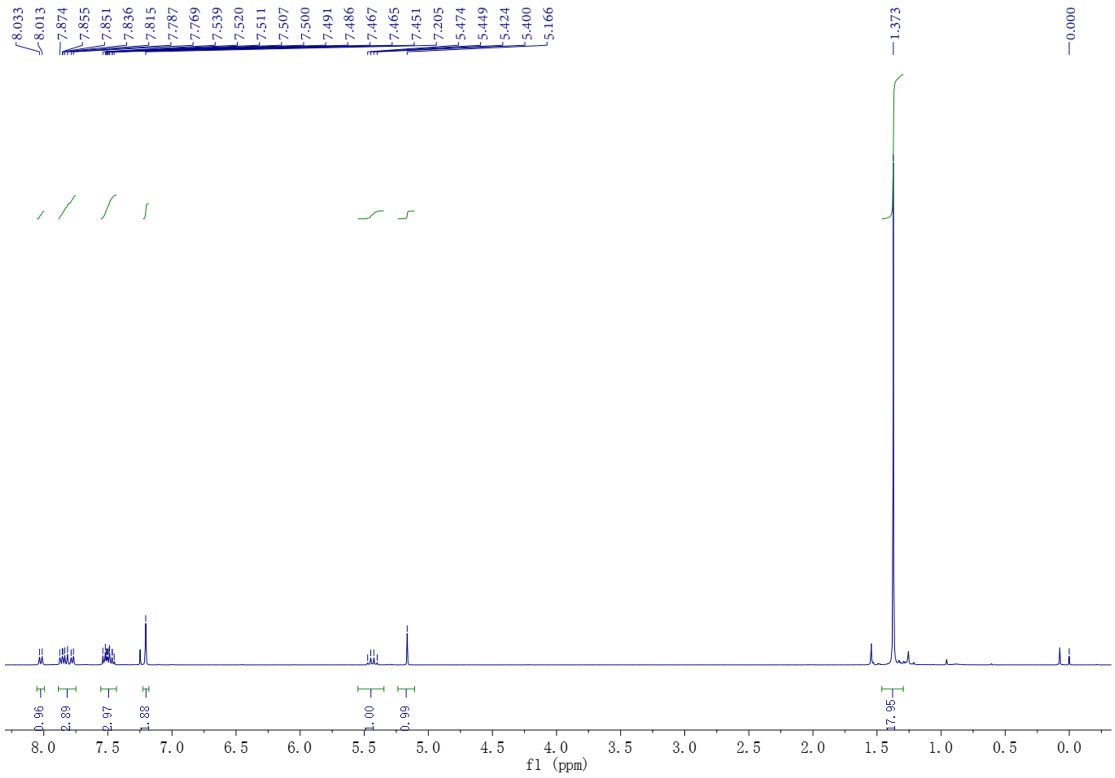

**$^{13}C\{^{1}H\}$ NMR**

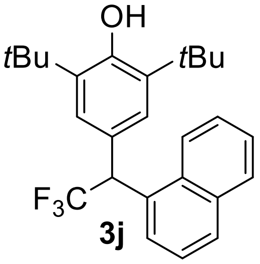


**3j**

(101 MHz, $CDCl_3$)

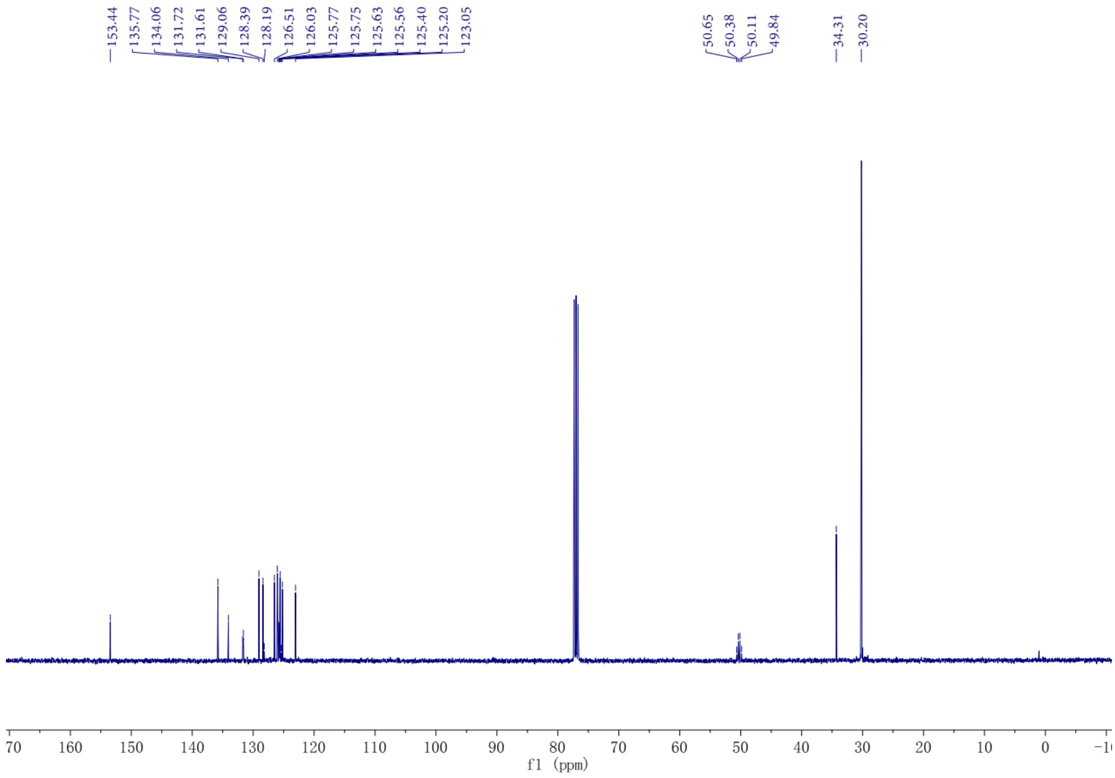

**$^{19}$F NMR**

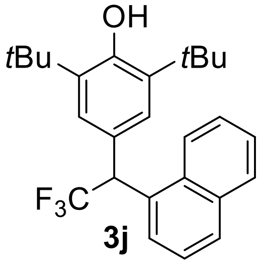


**3j**

(377 MHz, $CDCl_3$)

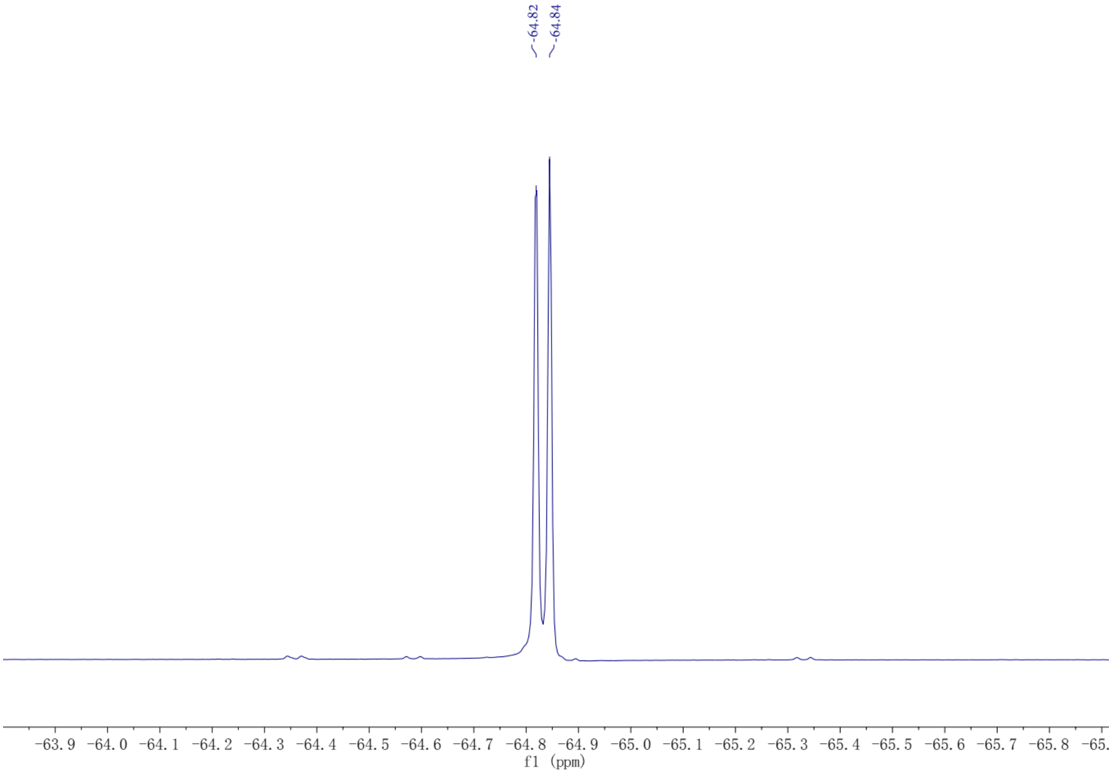

**$^{1}$H NMR**

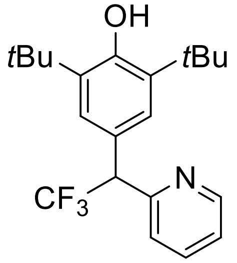


**3k**

(400 MHz, $CDCl_3$)

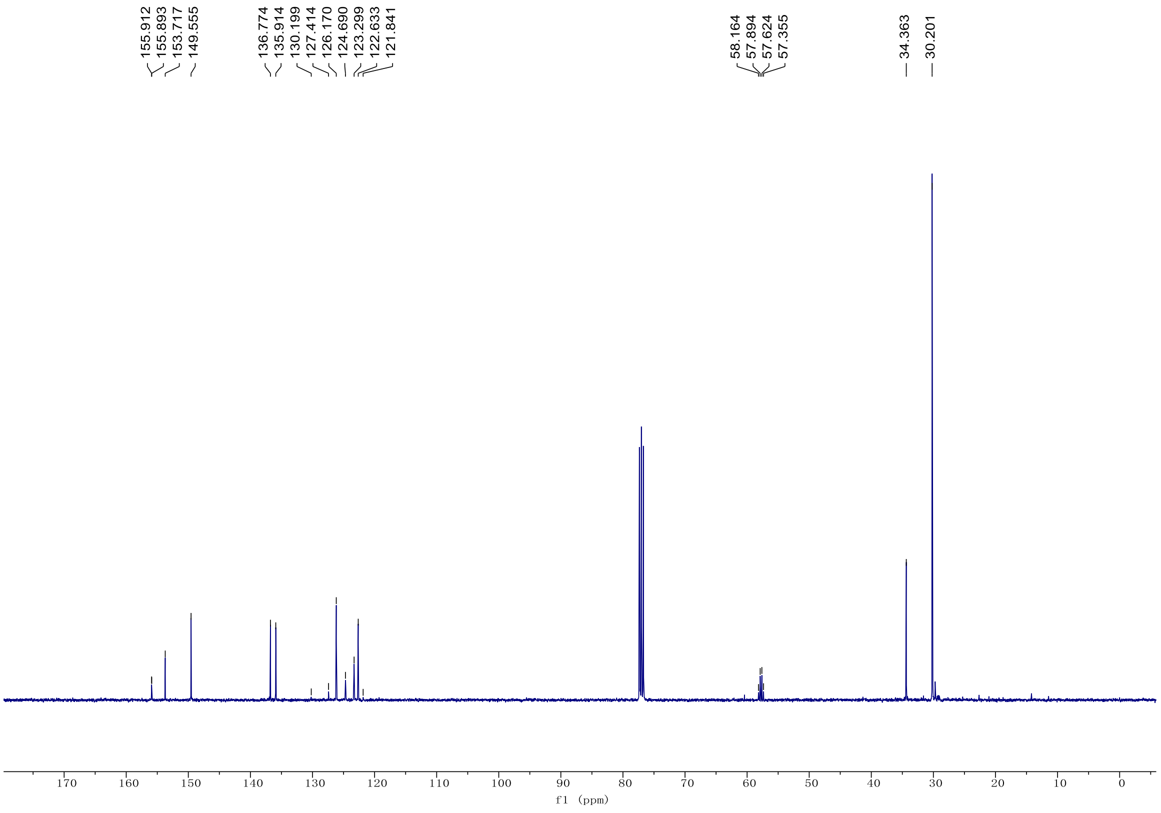

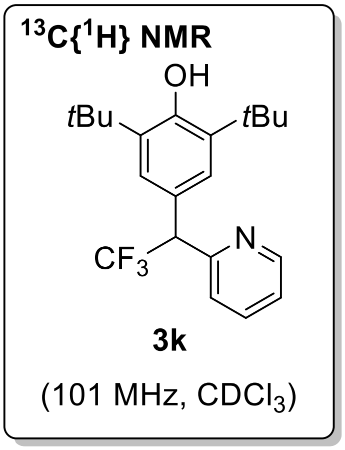

13C{1H} NMR
OH
tBu
tBu
N
CF3
3k
(101 MHz, CDCl3)


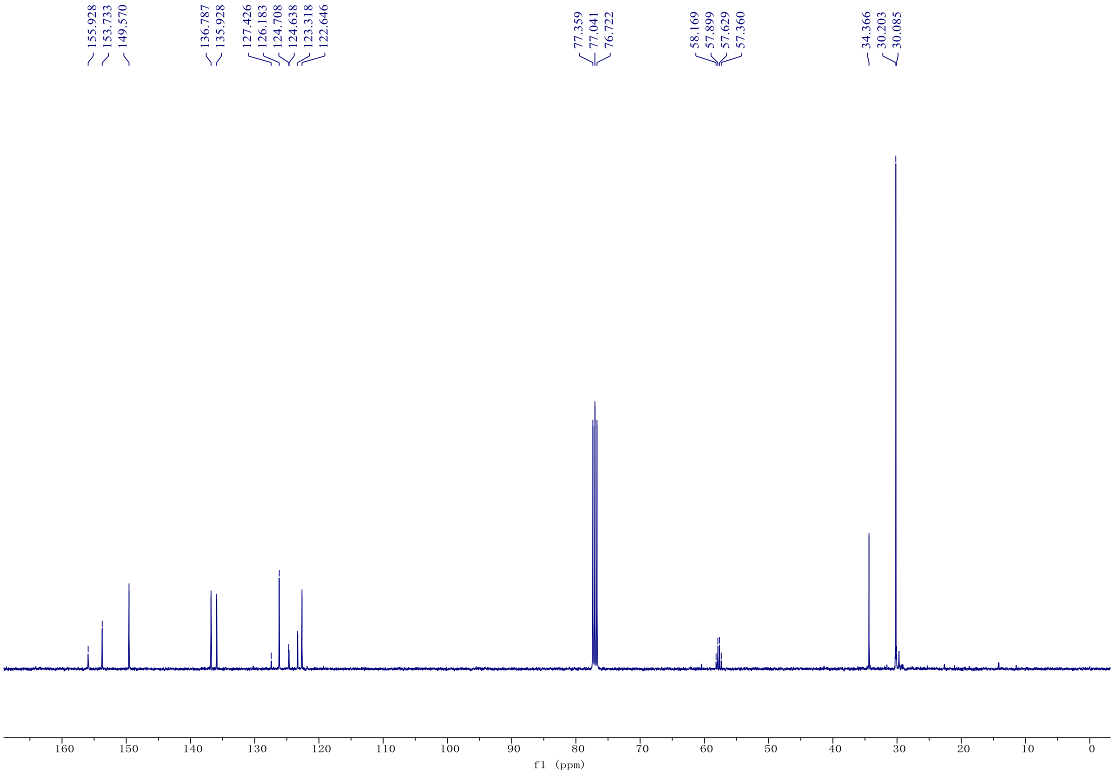

155.928
153.733
149.570
136.787
135.928
127.426
126.183
124.708
124.638
123.318
122.646
77.359
77.041
76.722
58.169
57.899
57.629
57.360
34.366
30.203
30.085
f1 (ppm)

$^{19}$F NMR

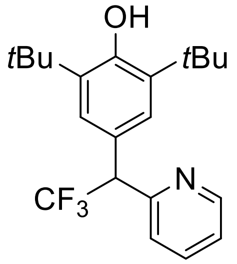


**3k**

(376 MHz, $CDCl_3$)

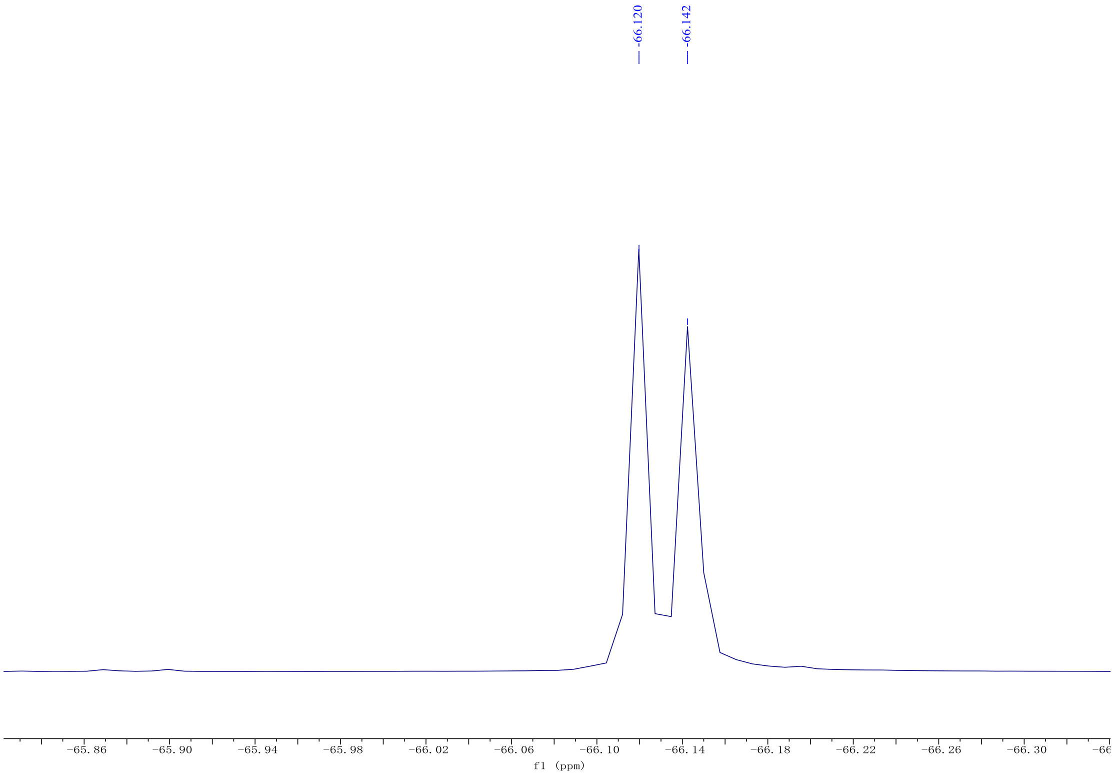

**$^{1}$H NMR**

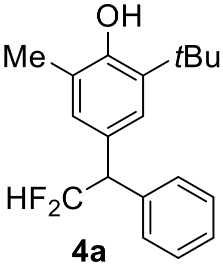


**4a**

(400 MHz, $CDCl_3$)

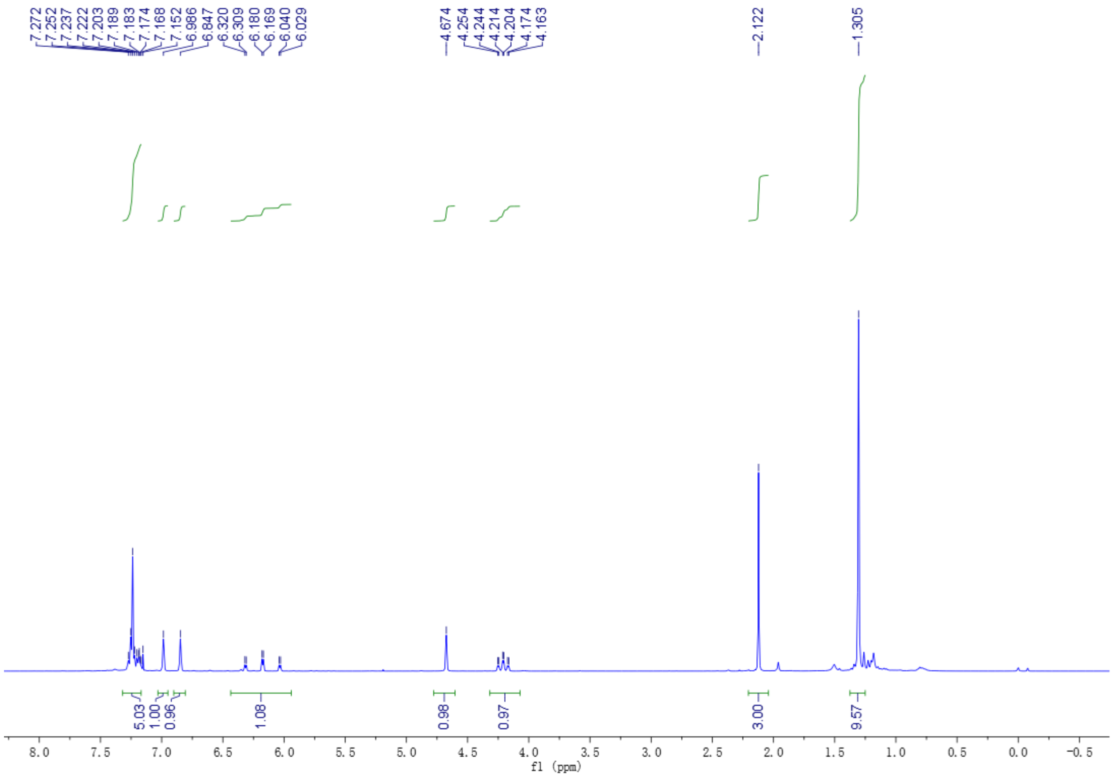

**$^{13}C\{^{1}H\}$ NMR**

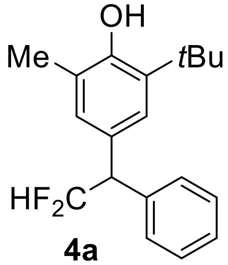


**4a**

(101 MHz, $CDCl_3$)

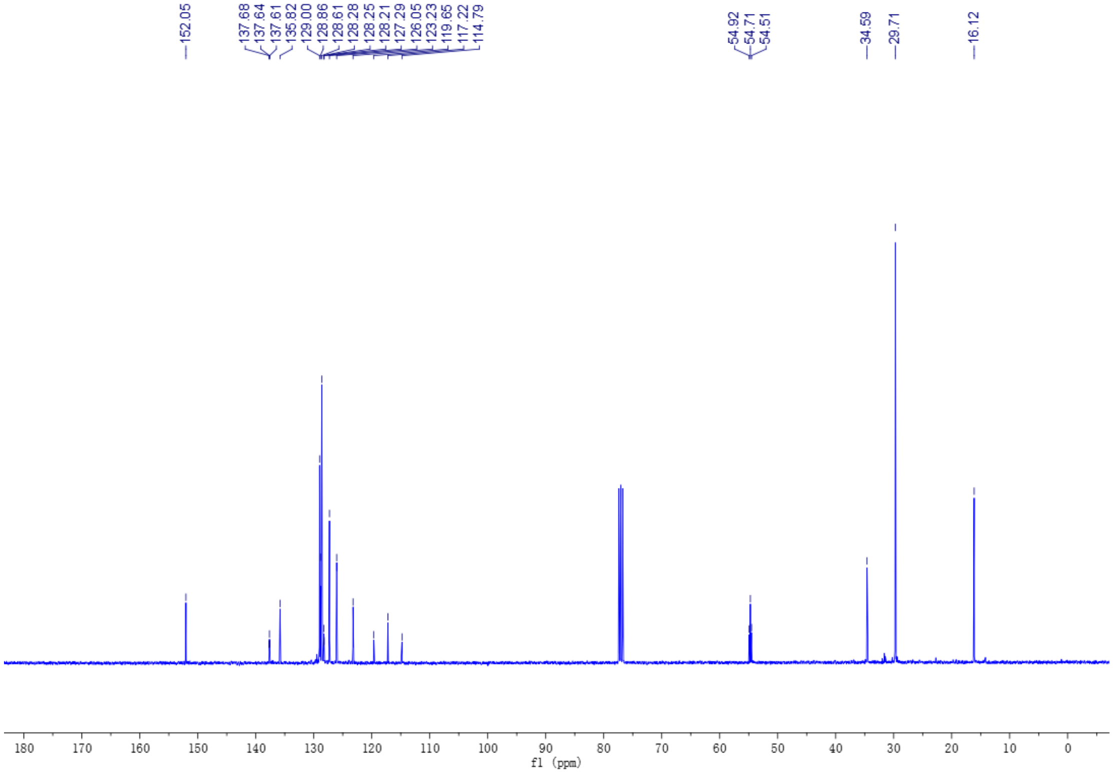

**$^{19}$F NMR**

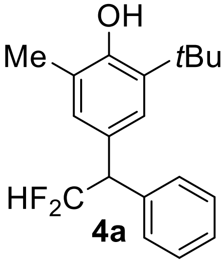


**4a**

(377 MHz, $CDCl_3$)

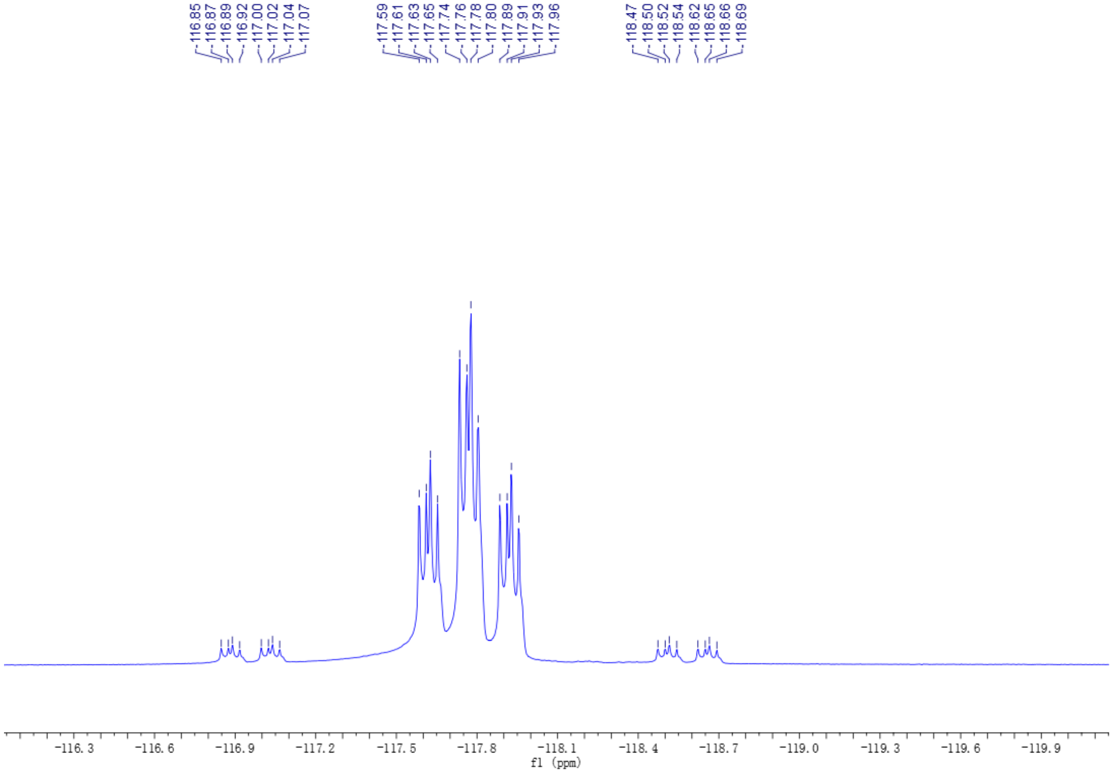

**$^1$H NMR**

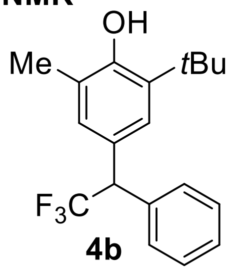


**4b**

(400 MHz, $CDCl_3$)

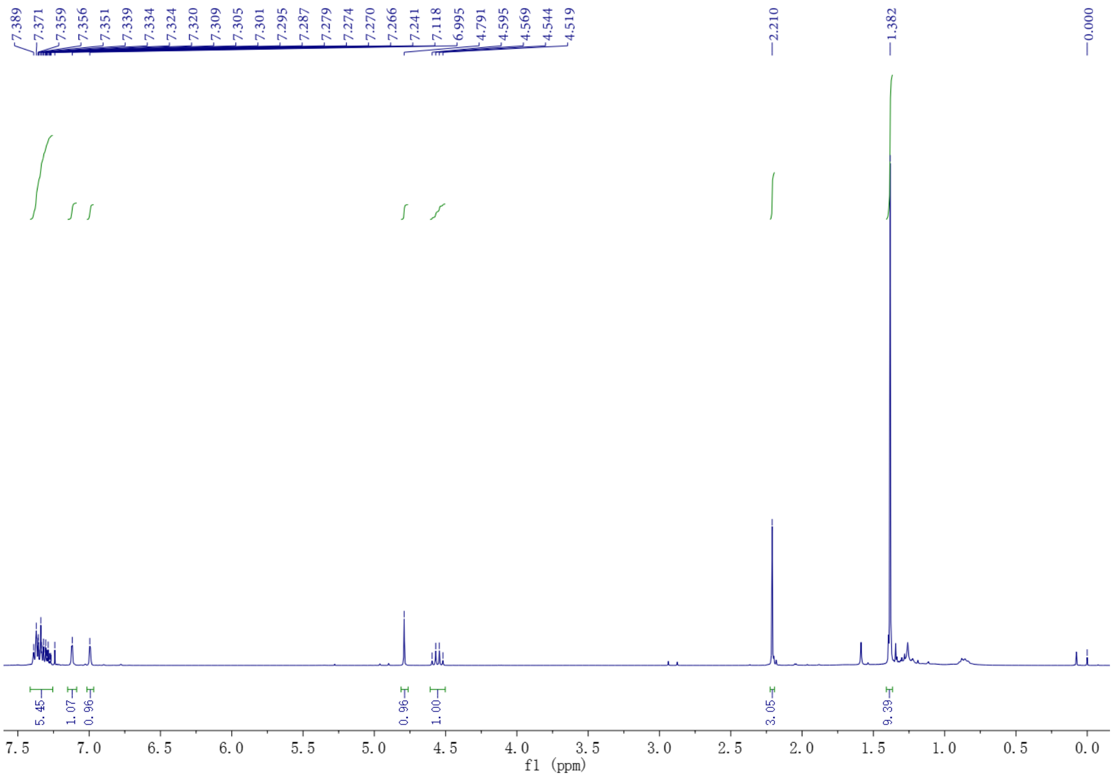

**$^{13}C\{^{1}H\}$ NMR**

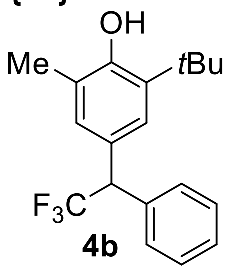


**4b**

(101 MHz, $CDCl_3$)

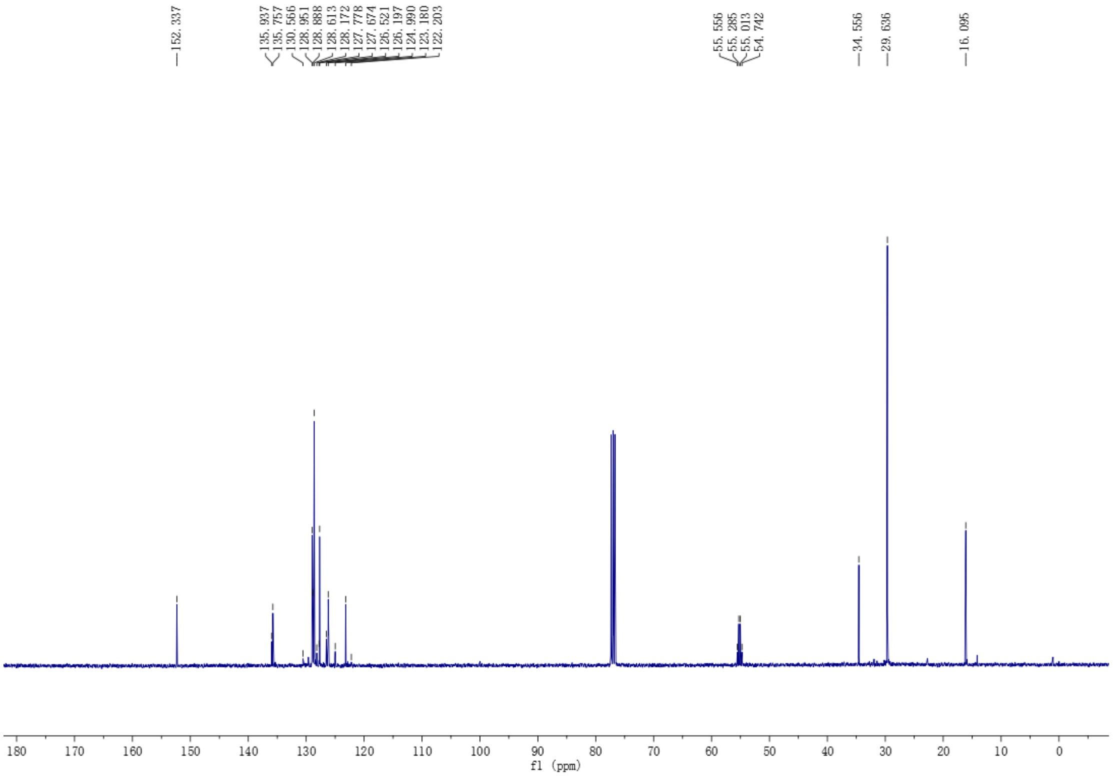

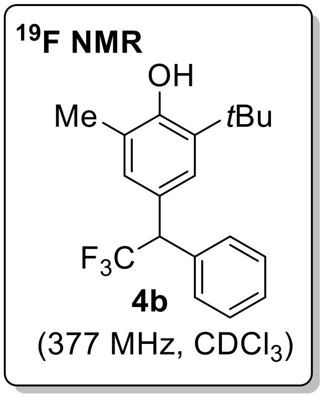
19F NMR
OH
Me
tBu
F3C
4b
(377 MHz, CDCl3)

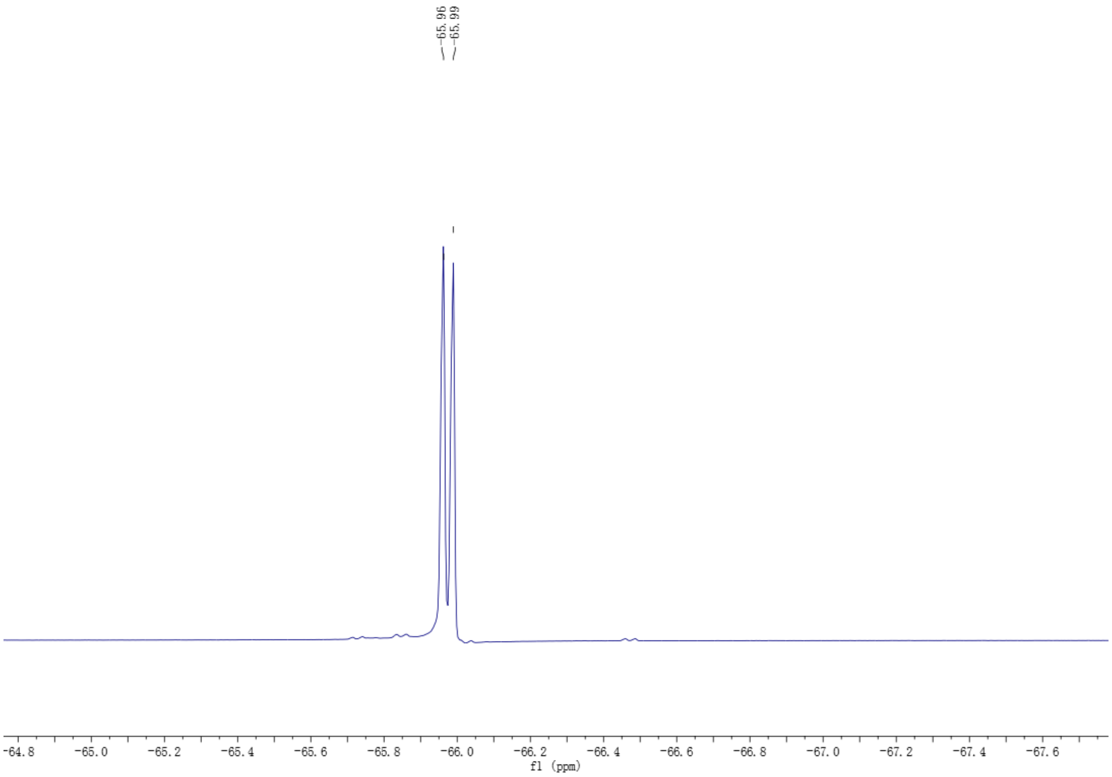
-65.96
-65.99
-64.8
-65.0
-65.2
-65.4
-65.6
-65.8
-66.0
-66.2
-66.4
-66.6
-66.8
-67.0
-67.2
-67.4
-67.6
f1 (ppm)

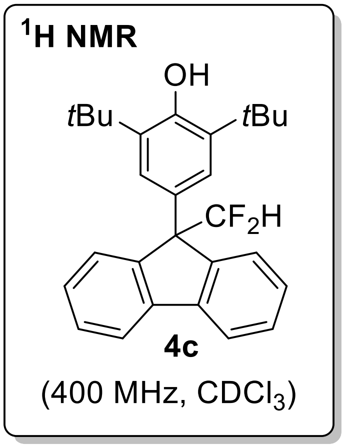

1H NMR
OH
tBu
tBu
CF2H
4c
(400 MHz, CDCl3)


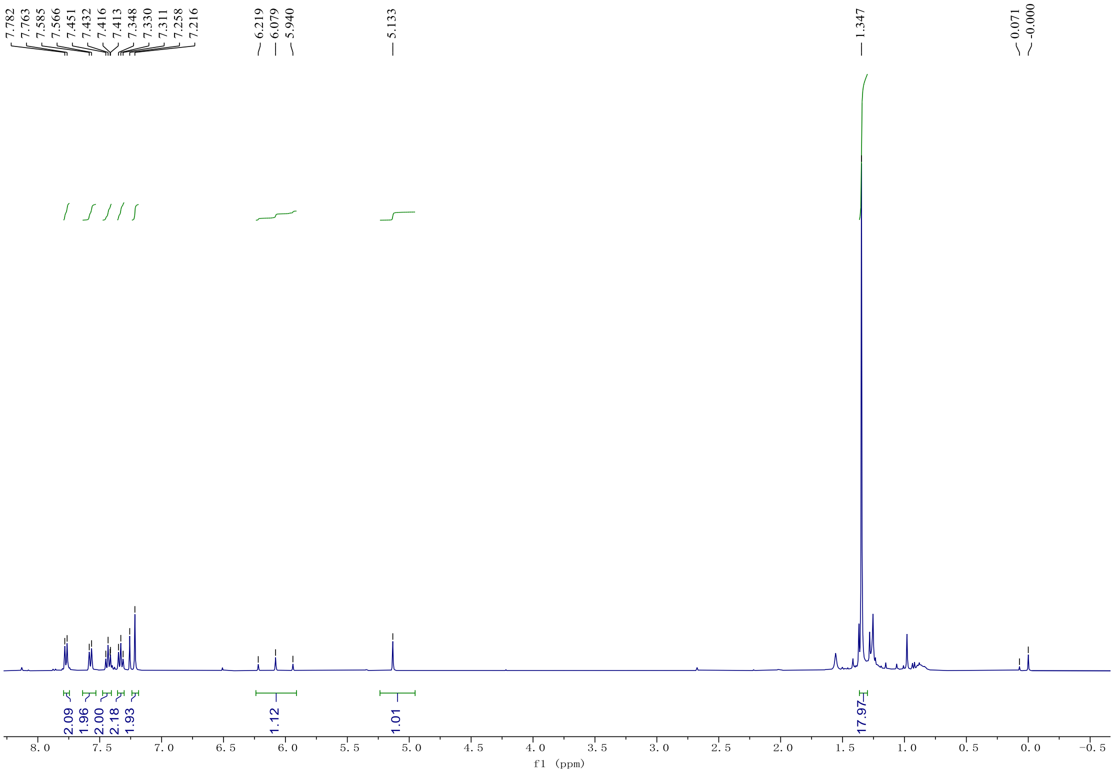

7.782
7.763
7.585
7.566
7.451
7.432
7.416
7.413
7.348
7.330
7.311
7.258
7.216
6.219
6.079
5.940
5.133
1.347
0.071
-0.000
2.09
1.96
2.00
2.18
1.93
1.12
1.01
17.97
8.0
7.5
7.0
6.5
6.0
5.5
5.0
4.5
4.0
3.5
3.0
2.5
2.0
1.5
1.0
0.5
0.0
-0.5
f1 (ppm)

**$^{13}C\{^{1}H\}$ NMR**

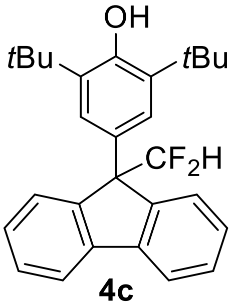


**4c**

(101 MHz, $CDCl_3$)

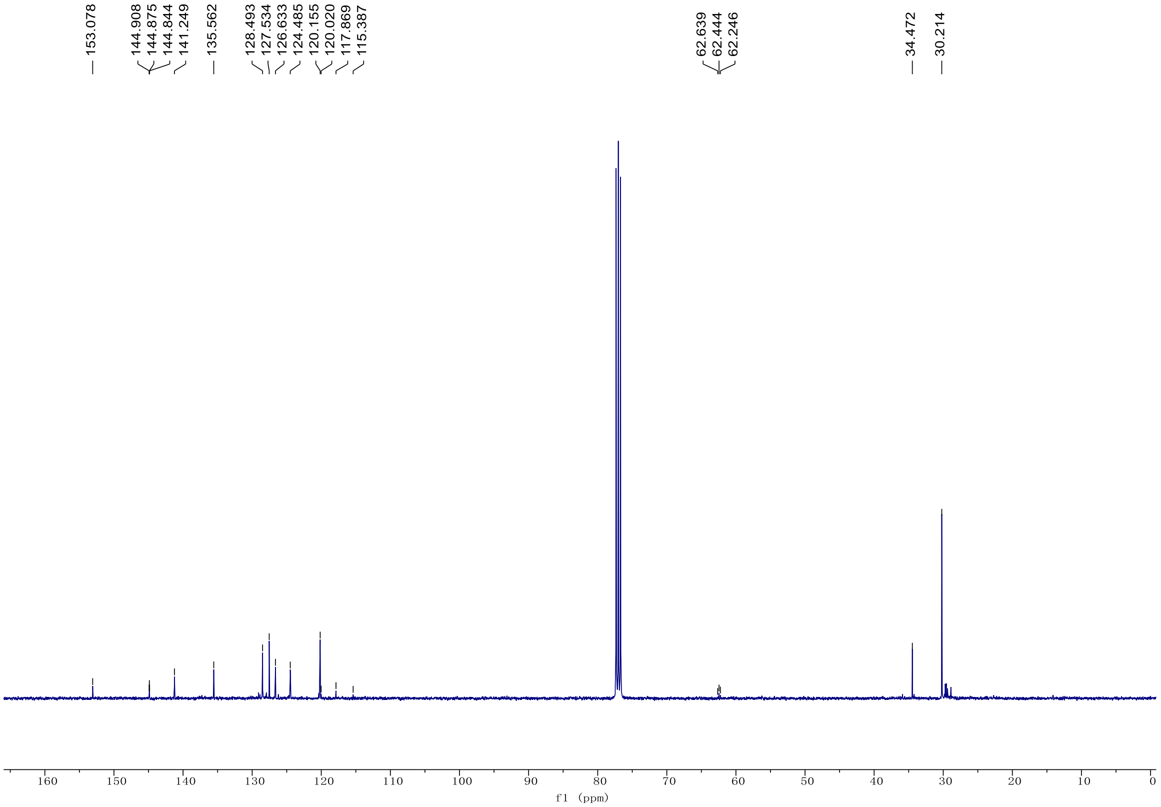

**$^{19}$F NMR**

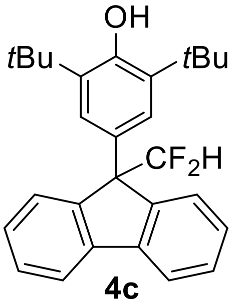


**4c**

(377 MHz, $CDCl_3$)

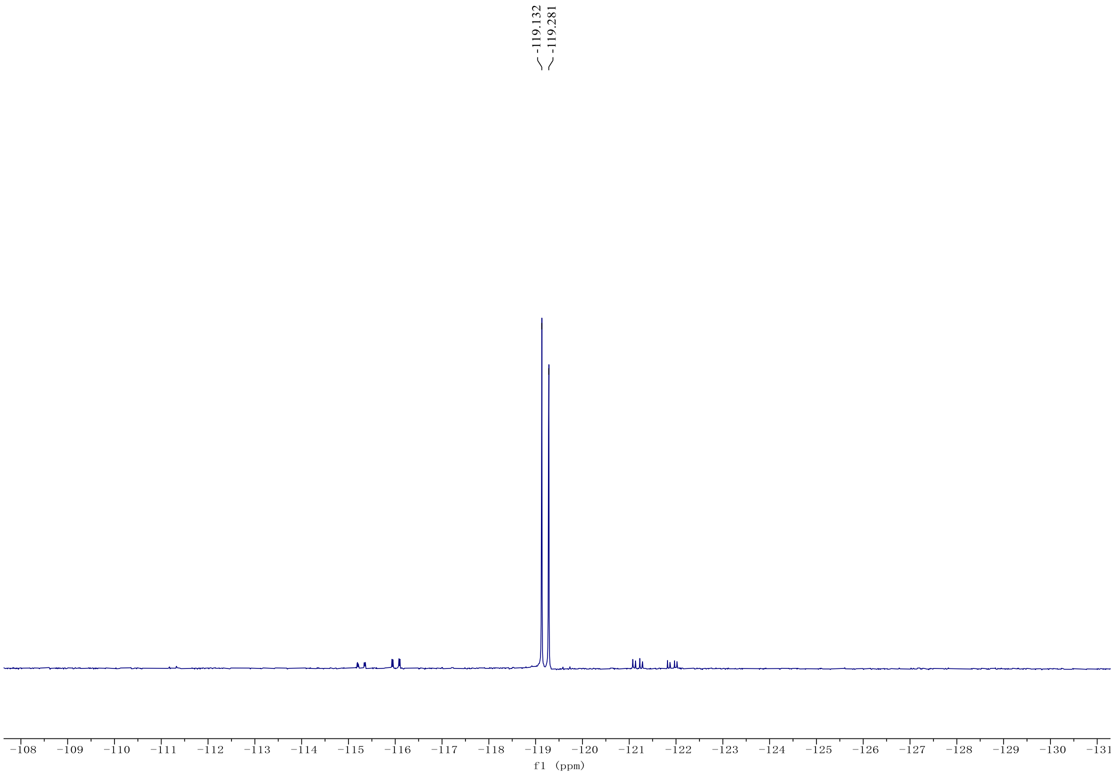

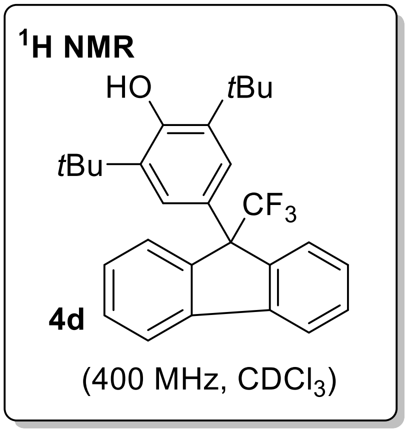

$^{1}$H NMR
HO
tBu
tBu
CF3
4d
(400 MHz, CDCl3)


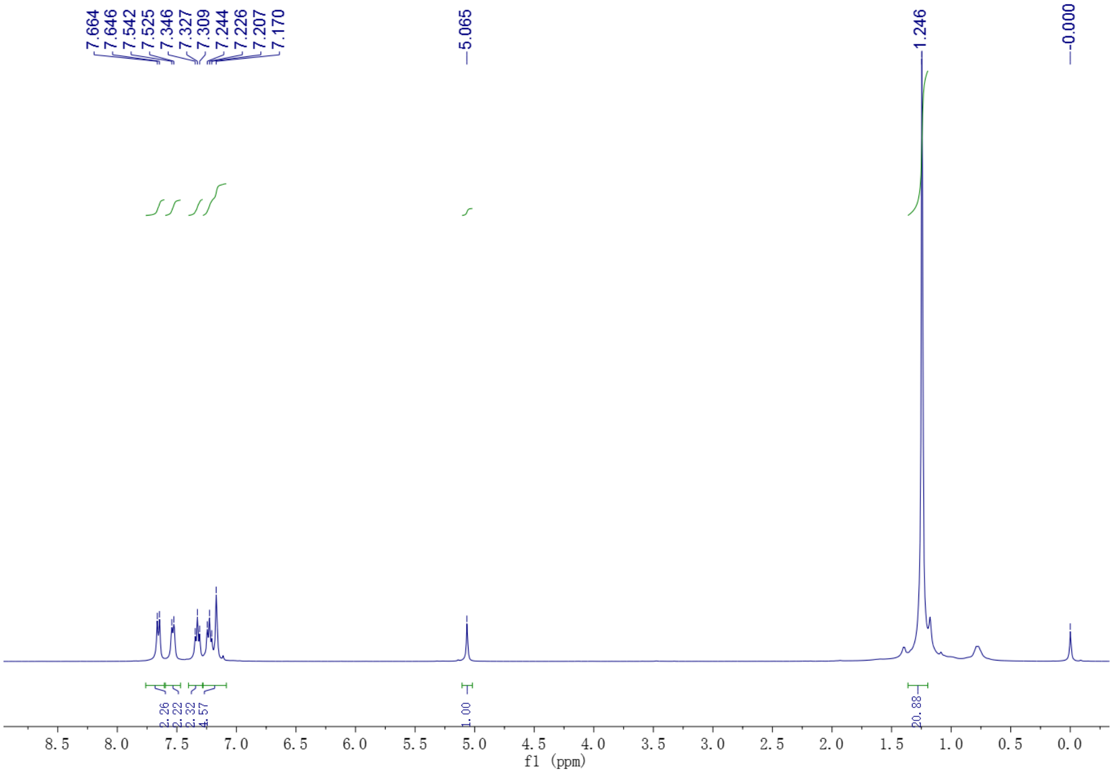

7.664
7.646
7.542
7.525
7.346
7.327
7.309
7.244
7.226
7.207
7.170
5.065
1.246
0.000
2.26
2.22
2.32
4.57
1.00
20.88
8.5
8.0
7.5
7.0
6.5
6.0
5.5
5.0
4.5
4.0
3.5
3.0
2.5
2.0
1.5
1.0
0.5
0.0
f1 (ppm)

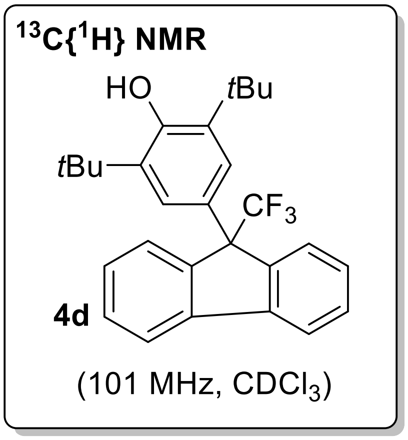

13C{1H} NMR
HO
tBu
tBu
CF3
4d
(101 MHz, CDCl3)


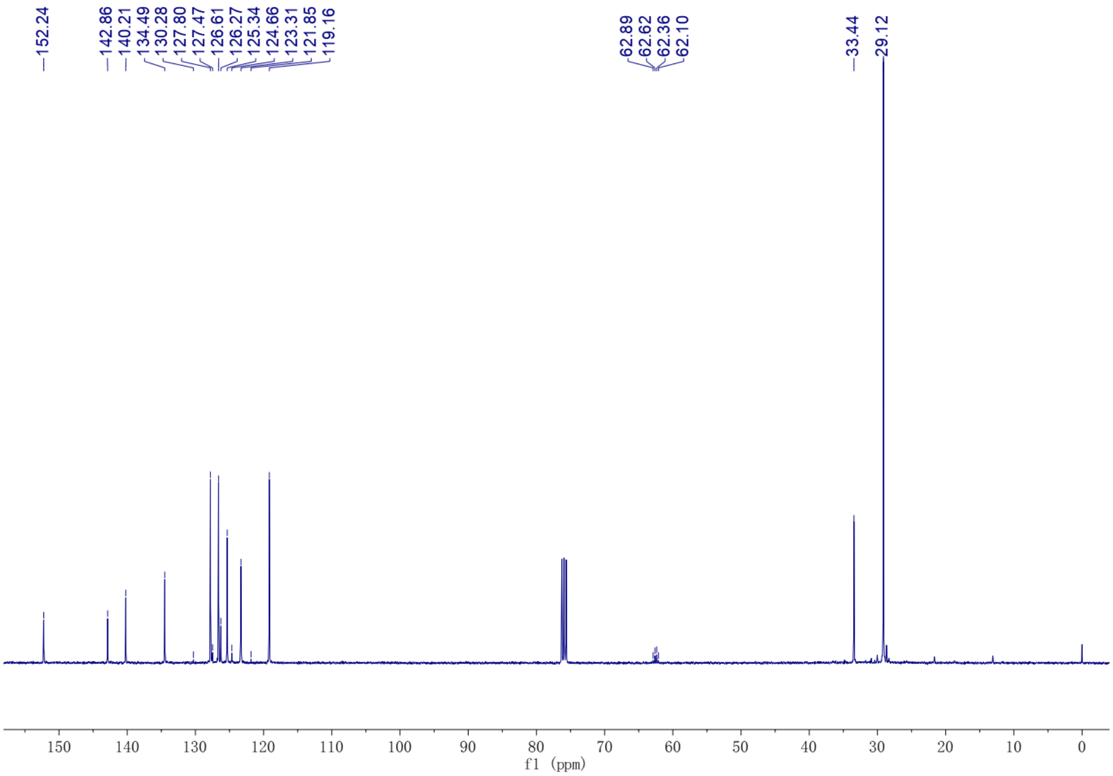

152.24
142.86
140.21
134.49
130.28
127.80
127.47
126.61
126.27
125.34
124.66
123.31
121.85
119.16
62.89
62.62
62.36
62.10
33.44
29.12
150
140
130
120
110
100
90
80
70
60
50
40
30
20
10
0
f1 (ppm)

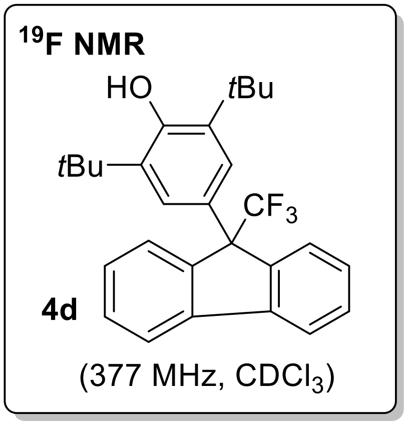

19F NMR
HO
tBu
tBu
CF3
4d
(377 MHz, CDCl3)


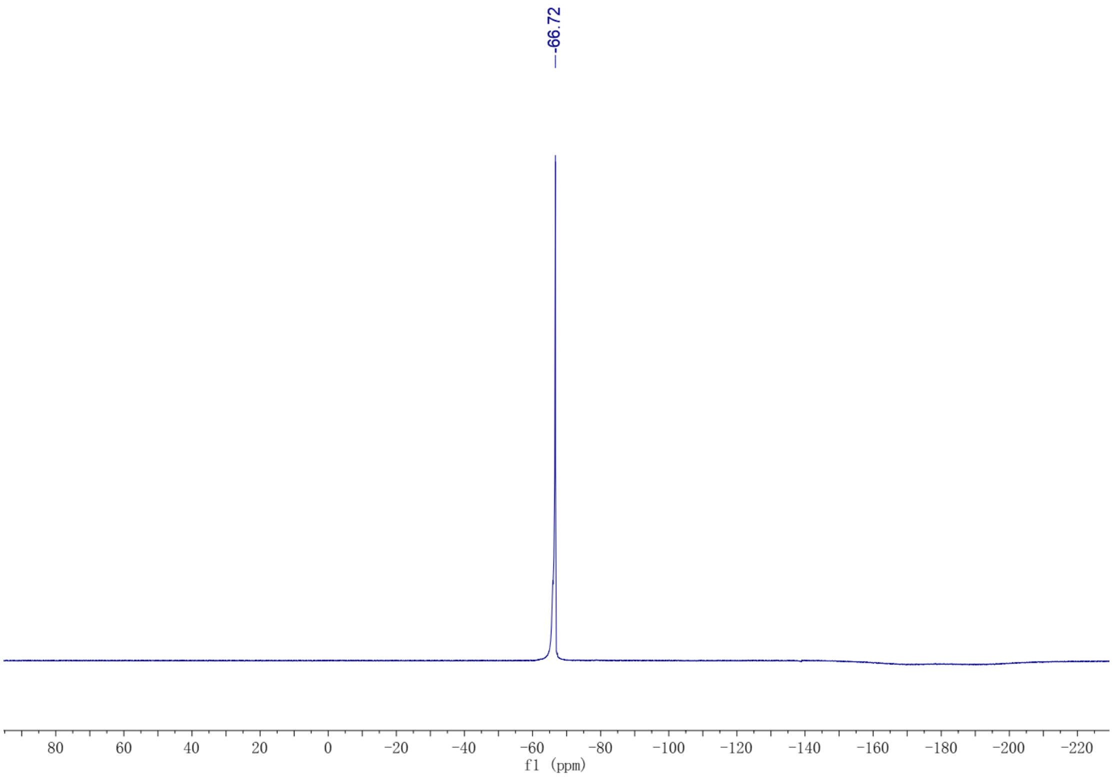

-66.72
80
60
40
20
0
−20
−40
−60
−80
−100
−120
−140
−160
−180
−200
−220
f1 (ppm)

**$^1$H NMR**

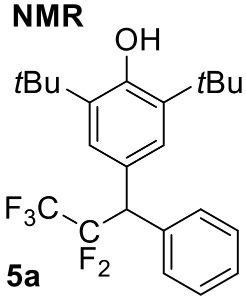


**5a**

(400 MHz, $CDCl_3$)

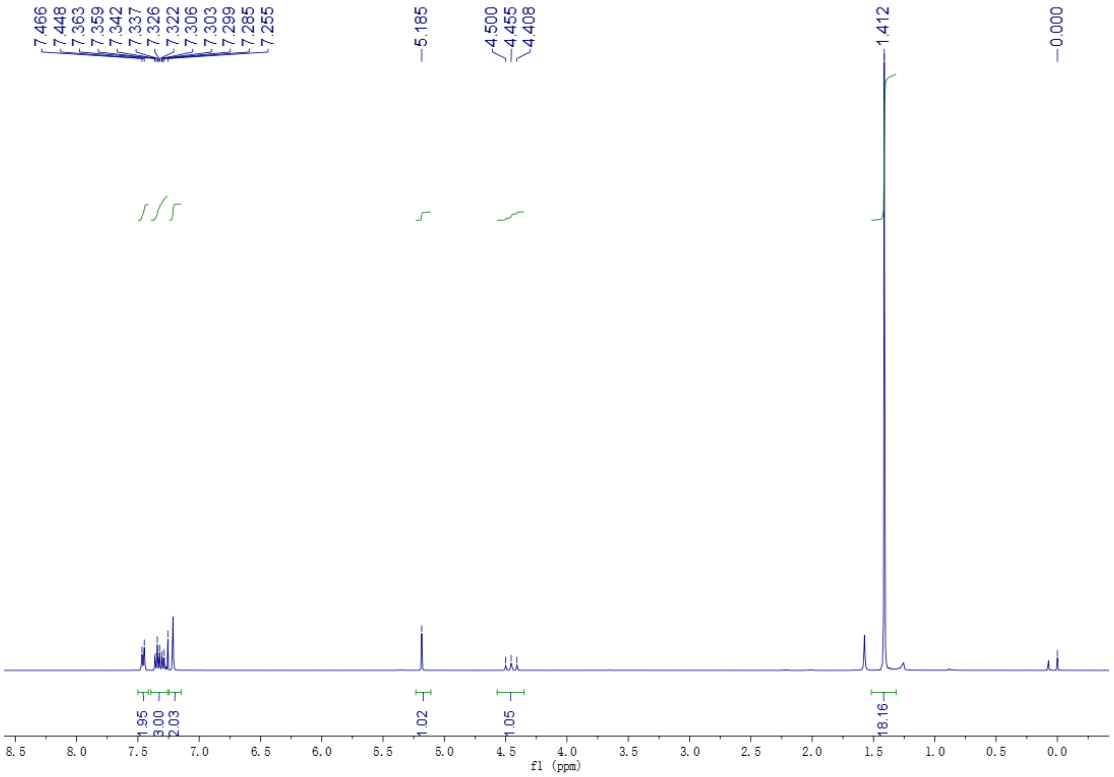

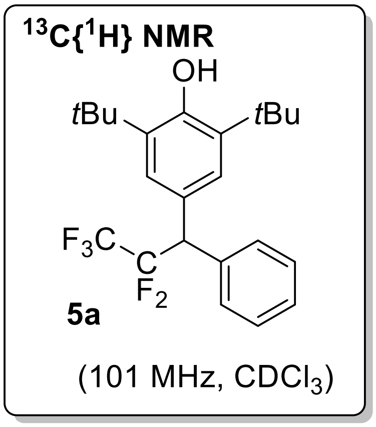

$^{13}C\{^1H\}$ NMR
OH
tBu
tBu
$F_3C$
C
$F_2$
5a
(101 MHz, $CDCl_3$)


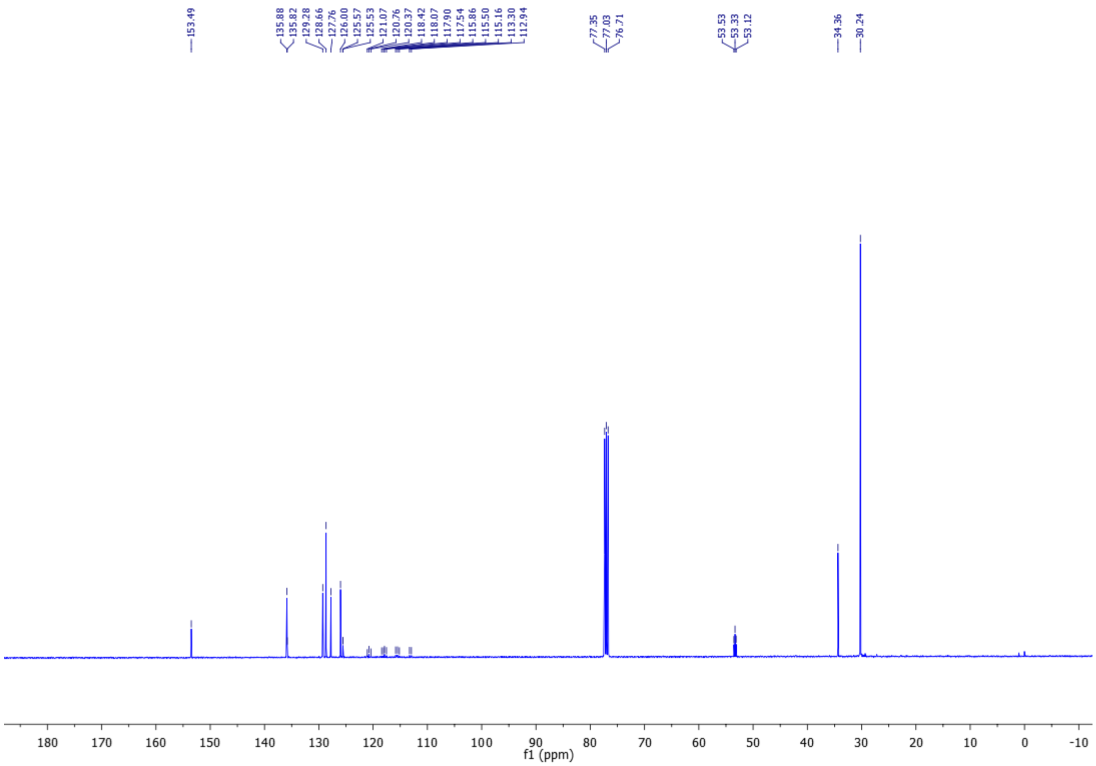

153.49
135.88
135.82
129.28
128.66
127.76
126.00
125.57
125.53
121.07
120.76
120.37
118.42
118.07
117.90
117.54
115.86
115.50
115.16
113.30
112.94
77.35
77.03
76.71
53.53
53.33
53.12
34.36
30.24
180
170
160
150
140
130
120
110
100
90
80
70
60
50
40
30
20
10
0
-10
f1 (ppm)

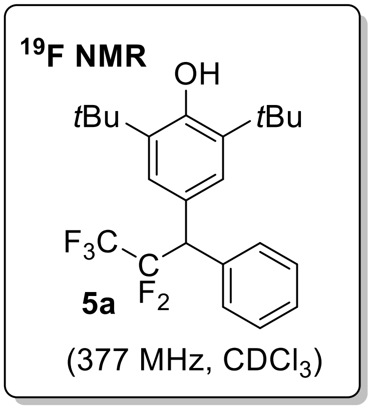

19F NMR
OH
tBu
tBu
F3C
C
F2
5a
(377 MHz, CDCl3)


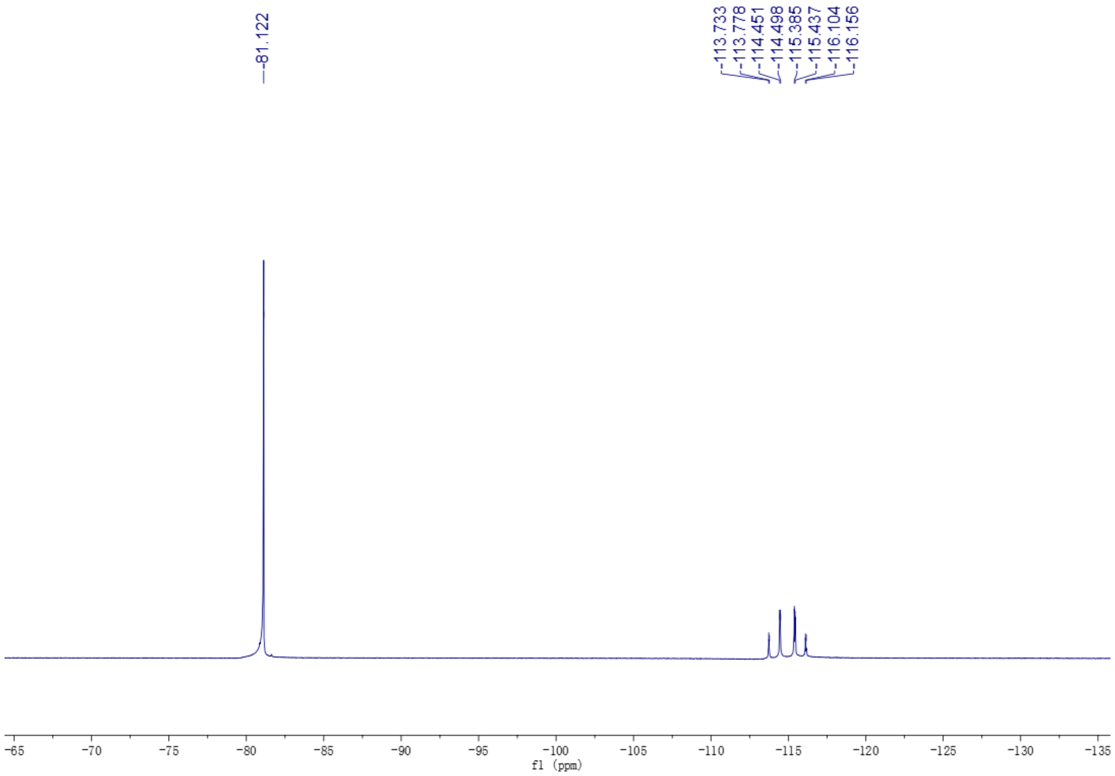

-81.122
-113.733
-113.778
-114.451
-114.498
-115.385
-115.437
-116.104
-116.156
-65
-70
-75
-80
-85
-90
-95
-100
-105
-110
-115
-120
-125
-130
-135
f1 (ppm)

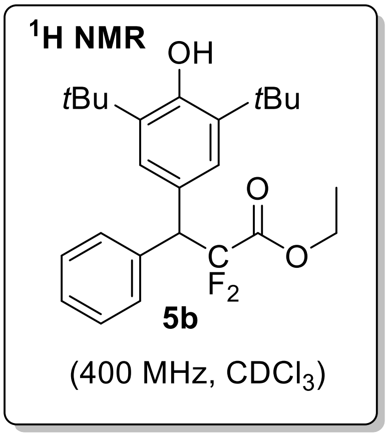

1H NMR
OH
tBu
tBu
O
C
O
F2
5b
(400 MHz, CDCl3)


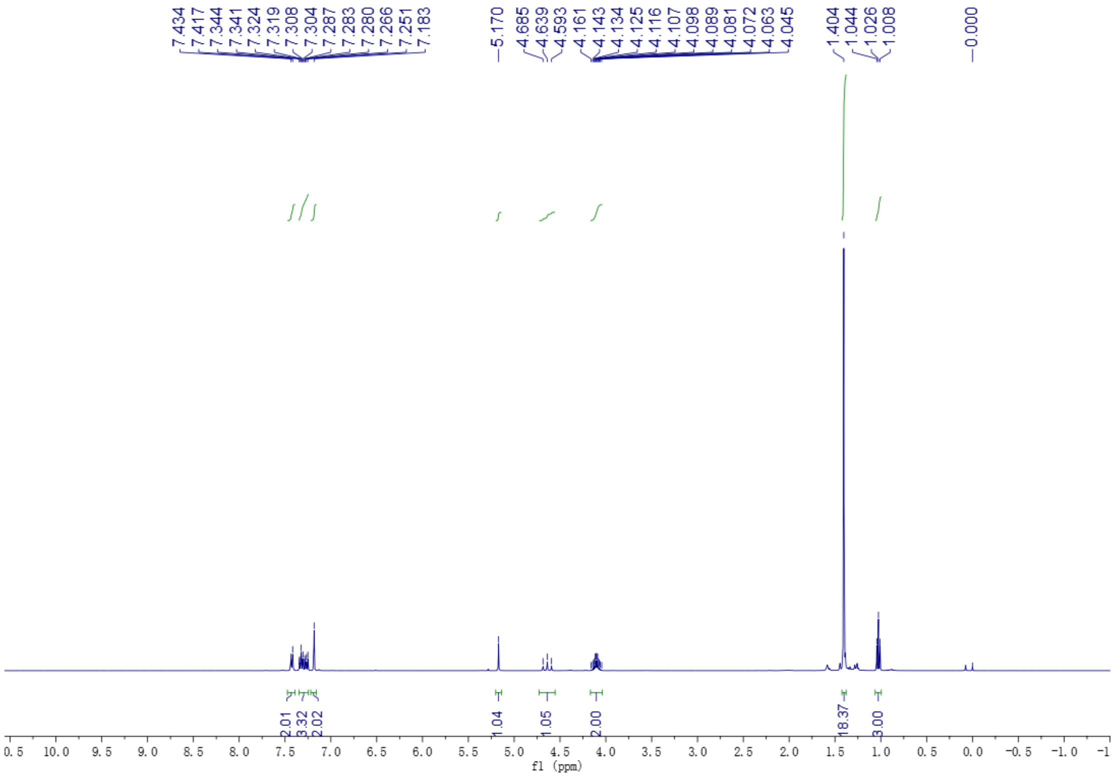

7.434
7.417
7.344
7.341
7.324
7.319
7.308
7.304
7.287
7.283
7.280
7.266
7.251
7.183
5.170
4.685
4.639
4.593
4.161
4.143
4.134
4.125
4.116
4.107
4.098
4.089
4.081
4.072
4.063
4.045
1.404
1.044
1.026
1.008
0.000
2.01
3.32
2.02
1.04
1.05
2.00
18.37
3.00
f1 (ppm)

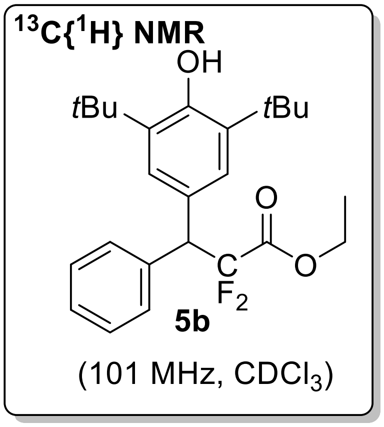
13C{1H} NMR
OH
tBu
tBu
O
O
C
F2
5b
(101 MHz, CDCl3)

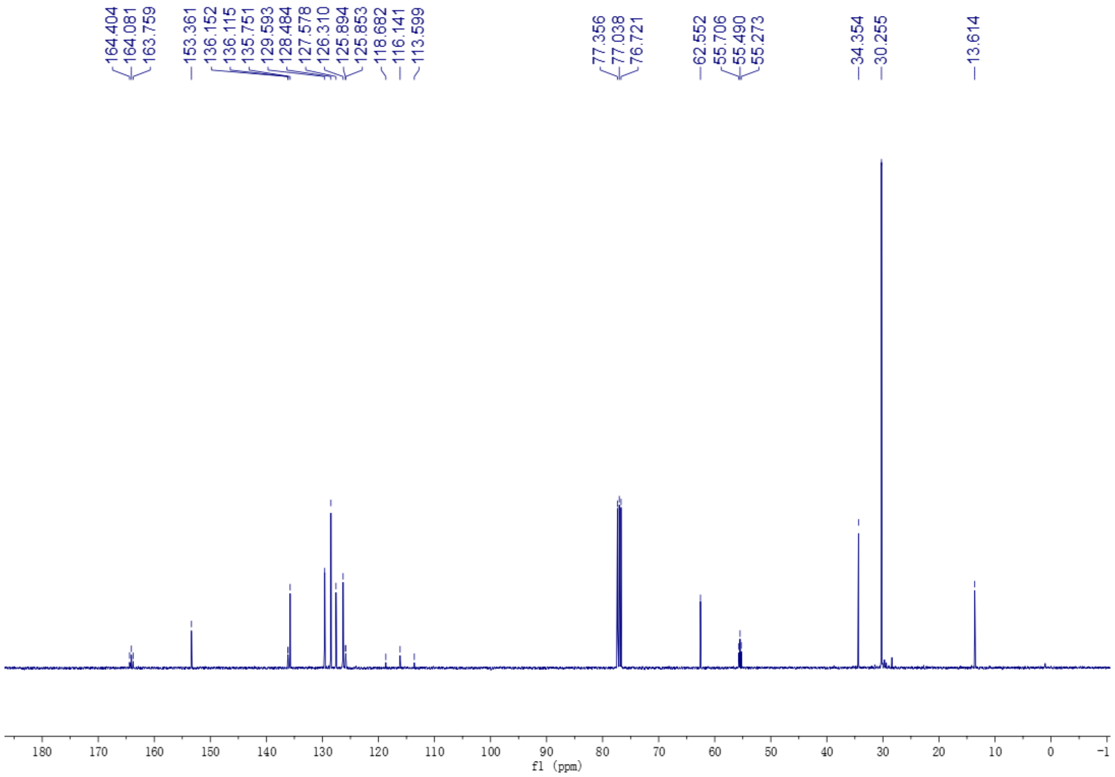
164.404
164.081
163.759
153.361
136.152
136.115
135.751
129.593
128.484
127.578
126.310
125.894
125.853
118.682
116.141
113.599
77.356
77.038
76.721
62.552
55.706
55.490
55.273
34.354
30.255
13.614
180 170 160 150 140 130 120 110 100 90 80 70 60 50 40 30 20 10 0 -1
f1 (ppm)

**$^{19}$F NMR**

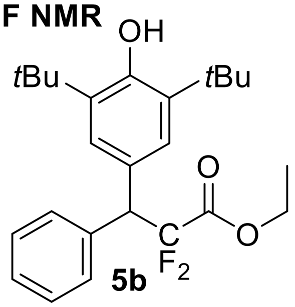


**5b**

(377 MHz, $CDCl_3$)

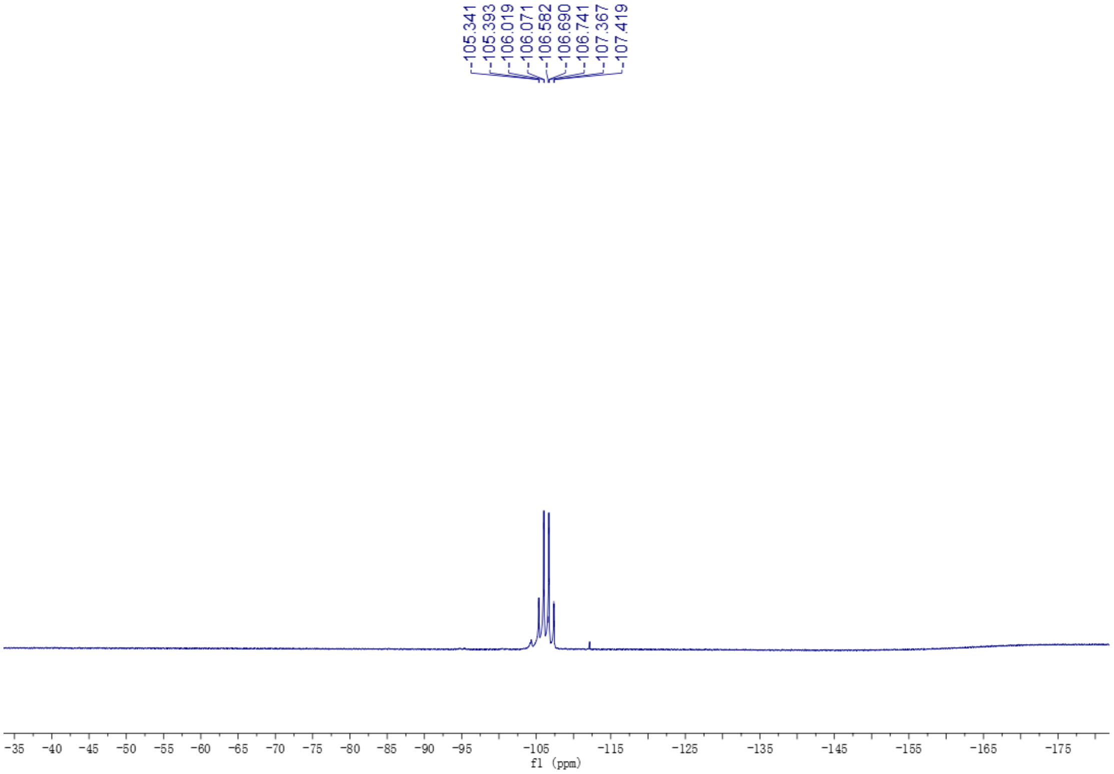

**$^1$H NMR**

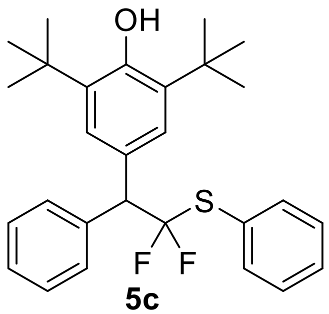

**5c**

(400 MHz, $CDCl_3$)

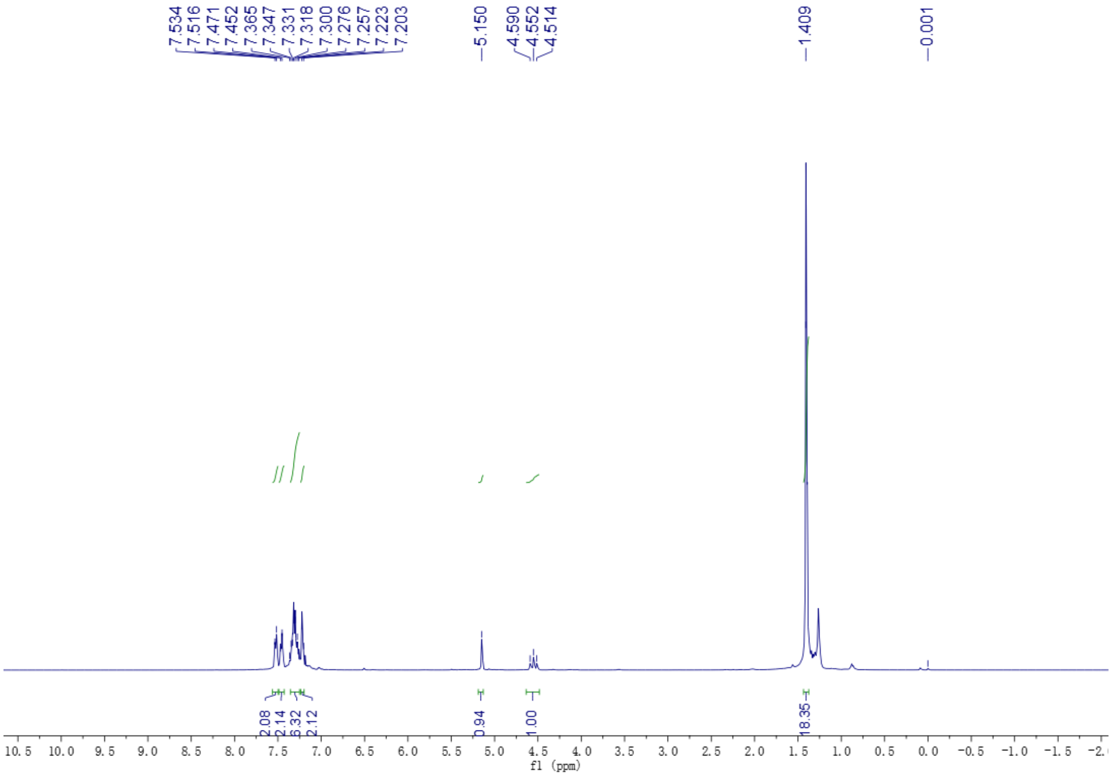

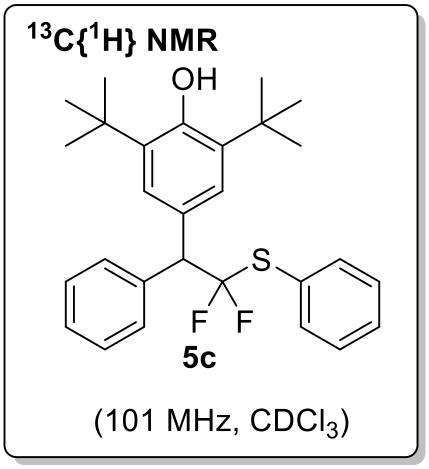

13C{1H} NMR
OH
S
F F
5c
(101 MHz, CDCl3)


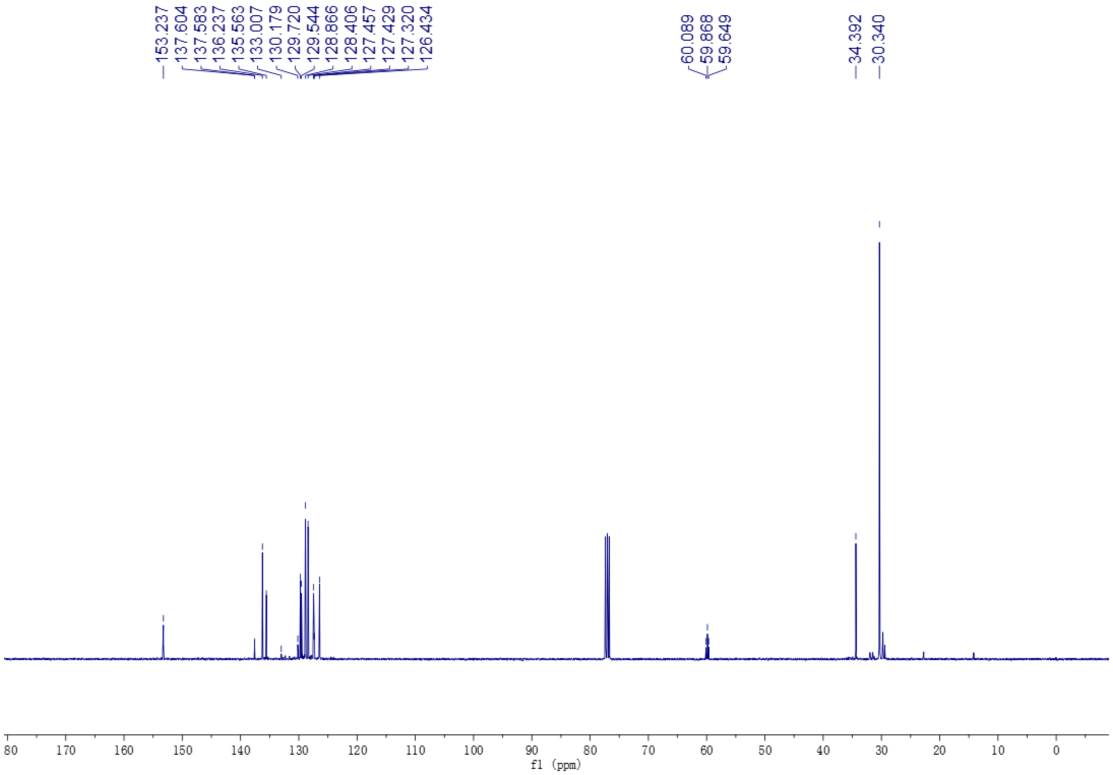

153.237
137.604
137.583
136.237
135.563
133.007
130.179
129.720
129.544
128.866
128.406
127.457
127.429
127.320
126.434
60.089
59.868
59.649
34.392
30.340
180
170
160
150
140
130
120
110
100
90
80
70
60
50
40
30
20
10
0
f1 (ppm)

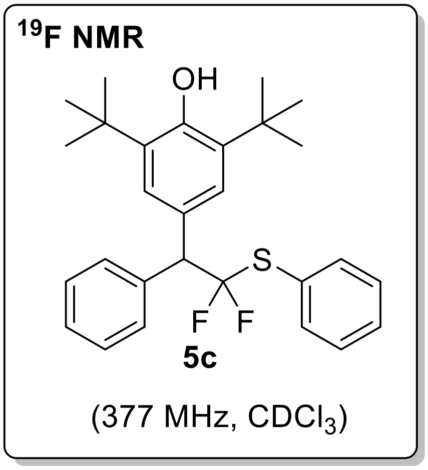

¹⁹F NMR
OH
S
F F
5c
(377 MHz, CDCl3)


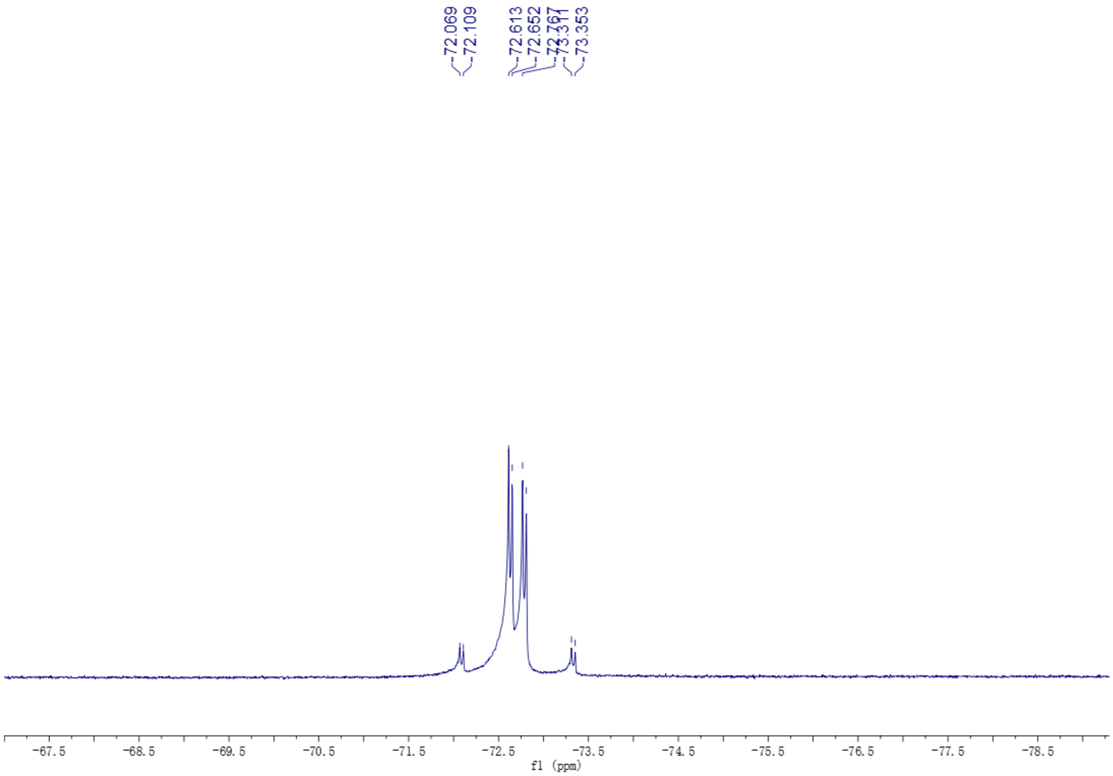

-72.069
-72.109
-72.613
-72.652
-73.767
-73.311
-73.353
-67.5
-68.5
-69.5
-70.5
-71.5
-72.5
-73.5
-74.5
-75.5
-76.5
-77.5
-78.5
f1 (ppm)

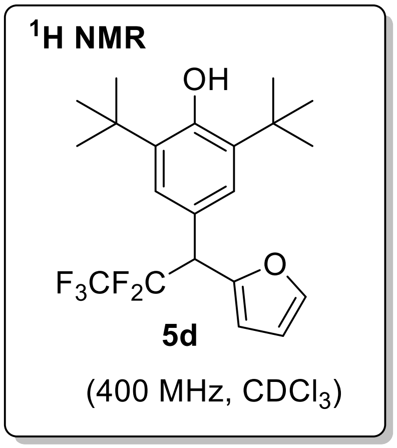


**5d**

(400 MHz, $CDCl_3$)

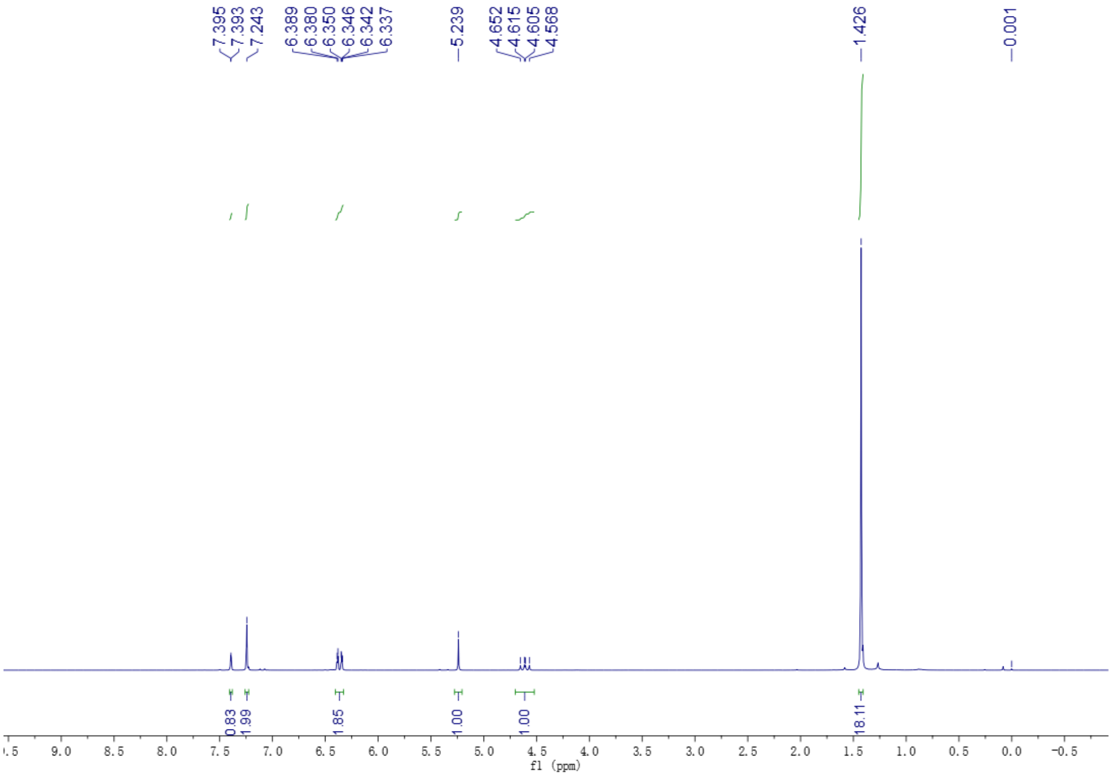

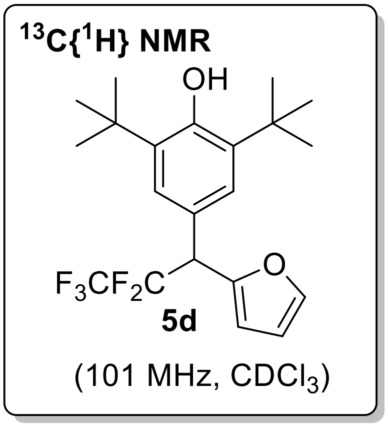
13C{1H} NMR
OH
F3CF2C
O
5d
(101 MHz, CDCl3)

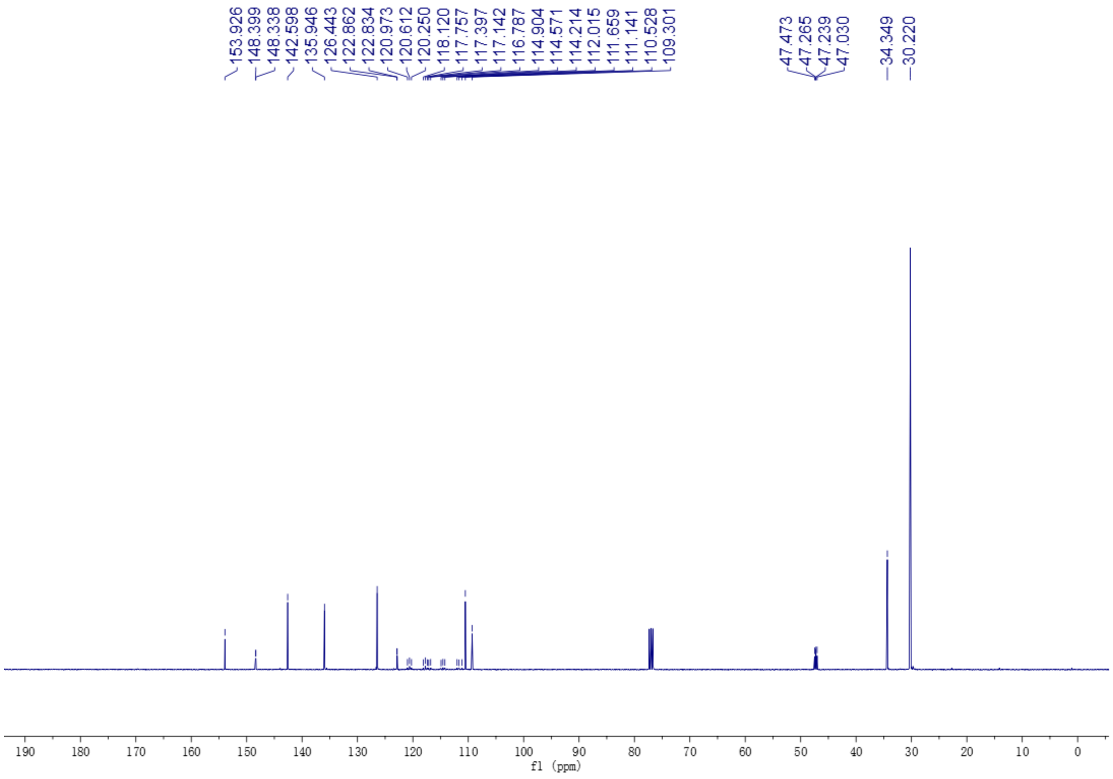
153.926
148.399
148.338
142.598
135.946
126.443
122.862
122.834
120.973
120.612
120.250
118.120
117.757
117.397
117.142
116.787
114.904
114.571
114.214
112.015
111.659
111.141
110.528
109.301
47.473
47.265
47.239
47.030
34.349
30.220
190
180
170
160
150
140
130
120
110
100
90
80
70
60
50
40
30
20
10
0
f1 (ppm)

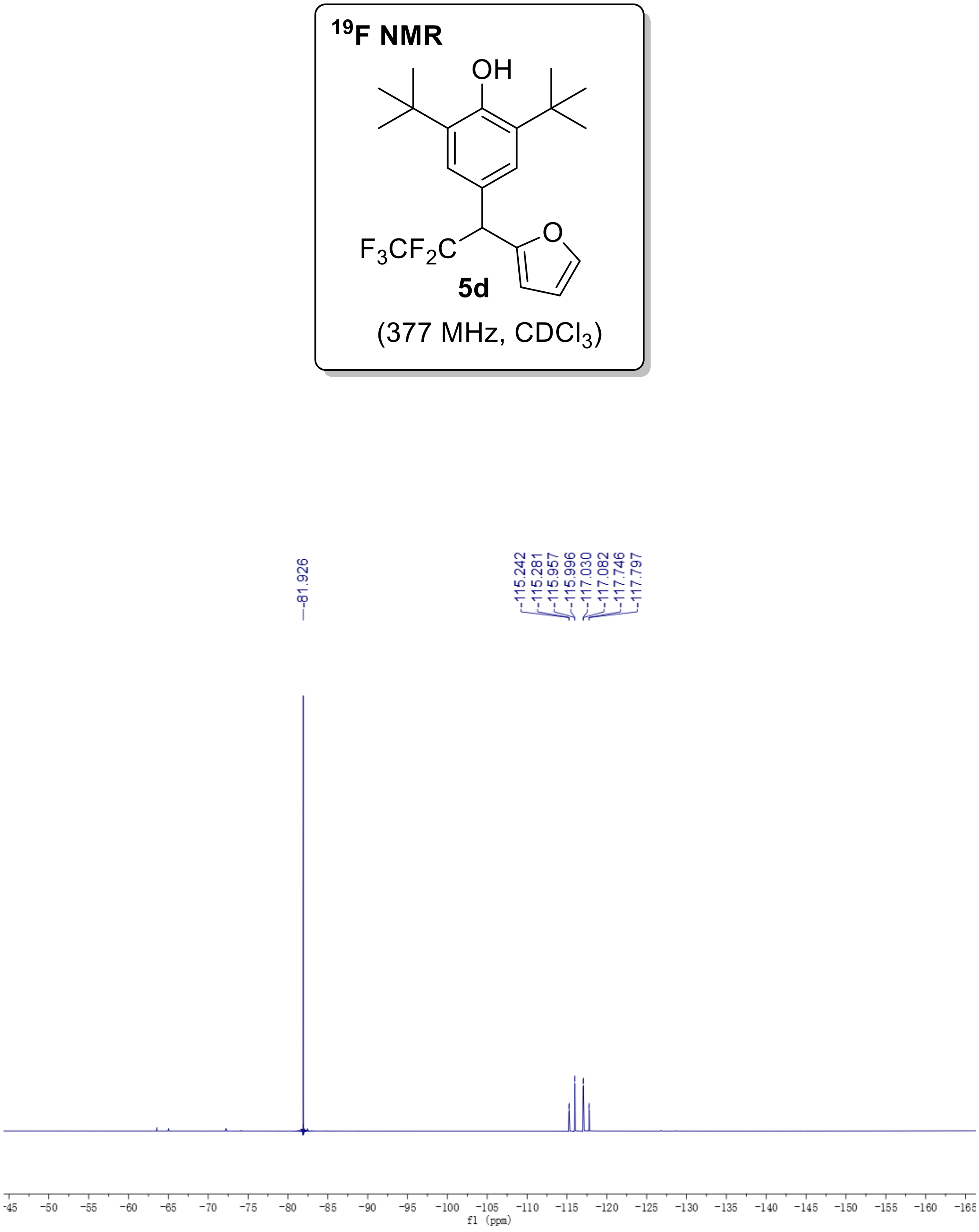

$^{19}$F NMR
OH
$F_3CF_2C$
O
5d
(377 MHz, $CDCl_3$)
-81.926
-115.242
-115.281
-115.957
-115.996
-117.030
-117.082
-117.746
-117.797
f1 (ppm)

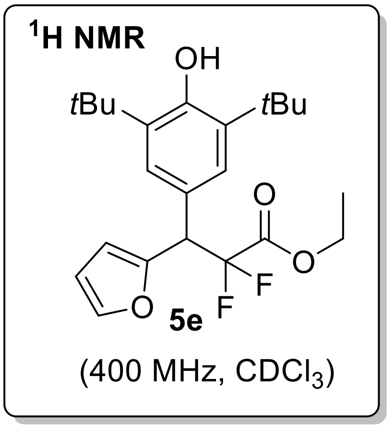
1H NMR
OH
tBu
tBu
O
O
F
F
O
5e
(400 MHz, CDCl3)

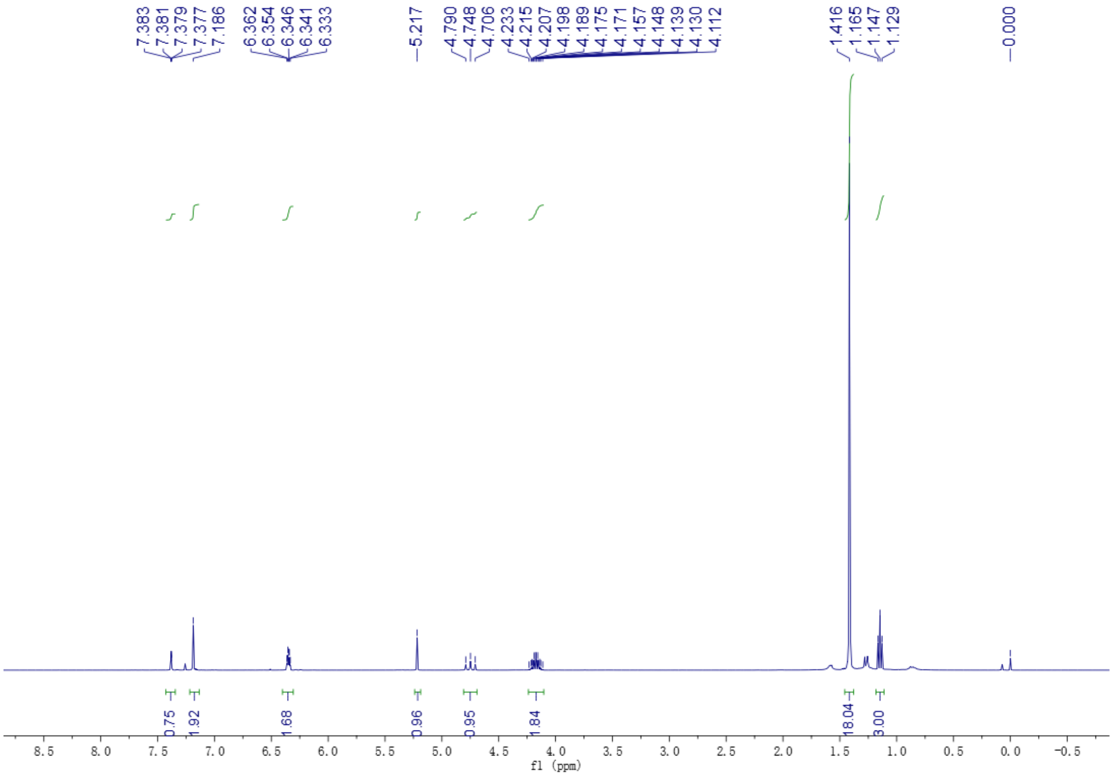
7.383
7.381
7.379
7.377
7.186
6.362
6.354
6.346
6.341
6.333
5.217
4.790
4.748
4.706
4.233
4.215
4.207
4.198
4.189
4.175
4.171
4.157
4.148
4.139
4.130
4.112
1.416
1.165
1.147
1.129
0.000
0.75
1.92
1.68
0.96
0.95
1.84
18.04
3.00
8.5
8.0
7.5
7.0
6.5
6.0
5.5
5.0
4.5
4.0
3.5
3.0
2.5
2.0
1.5
1.0
0.5
0.0
-0.5
f1 (ppm)

**$^{13}C\{^{1}H\}$ NMR**

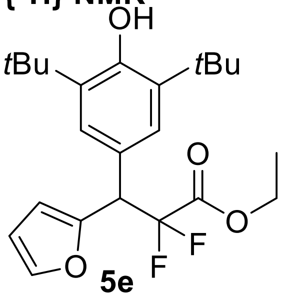

**5e**

(101 MHz, $CDCl_3$)

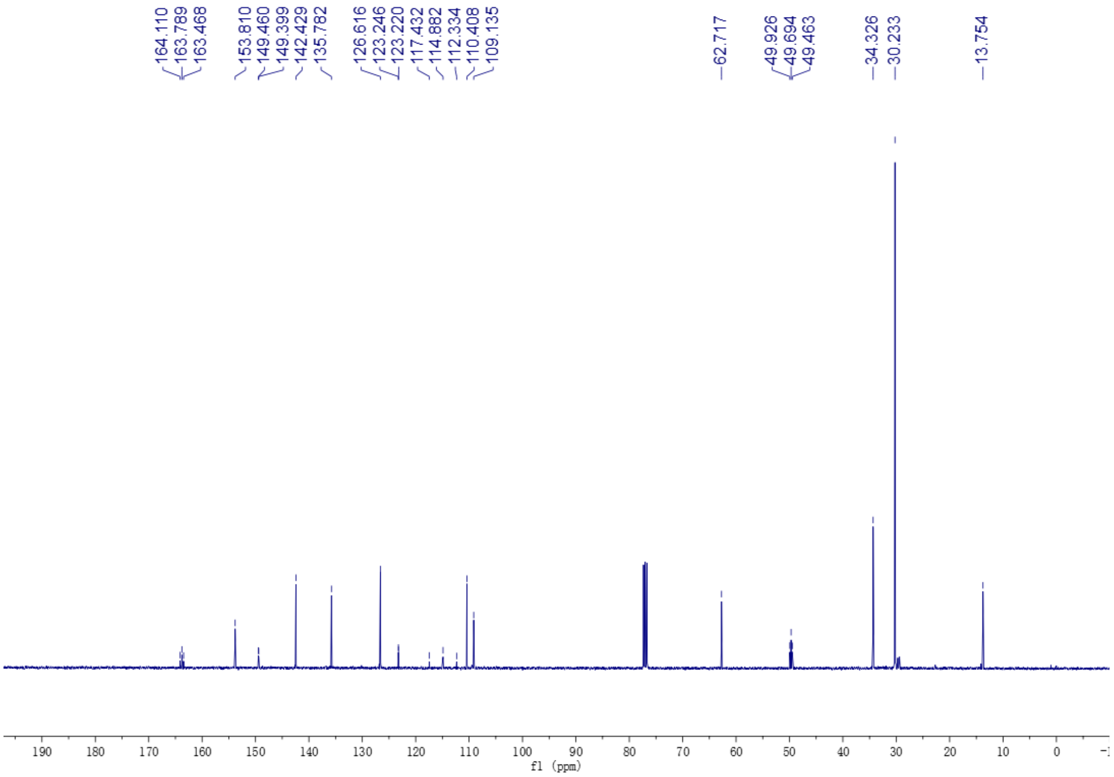

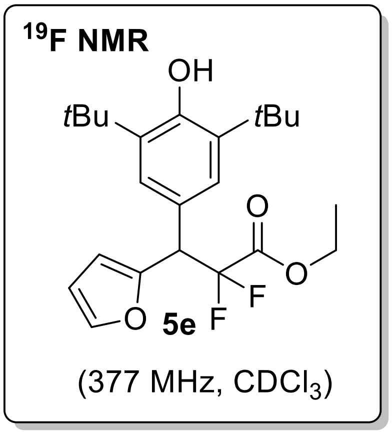
19F NMR
OH
tBu
tBu
O
O
F
F
O
5e
(377 MHz, CDCl3)

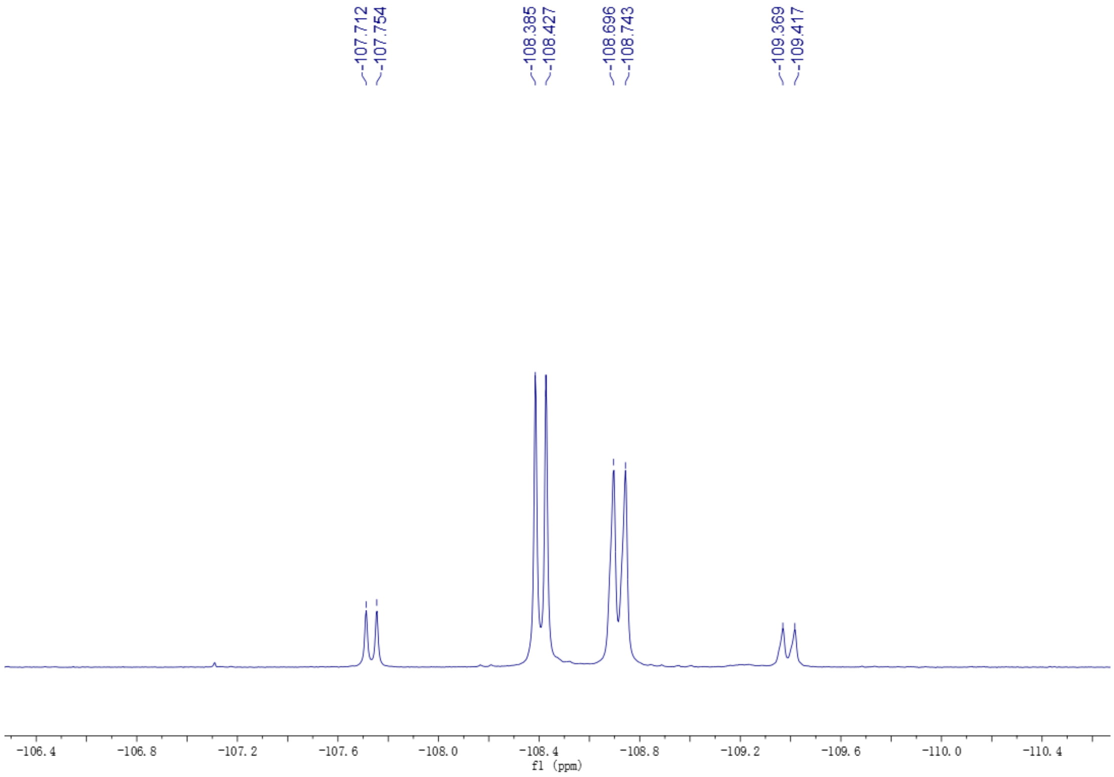
-107.712
-107.754
-108.385
-108.427
-108.696
-108.743
-109.369
-109.417
-106.4
-106.8
-107.2
-107.6
-108.0
-108.4
-108.8
-109.2
-109.6
-110.0
-110.4
f1 (ppm)

**$^1$H NMR**

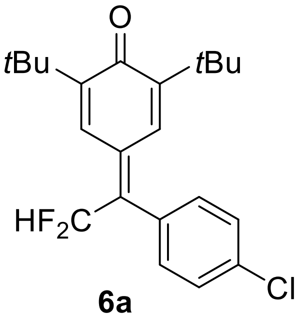


**6a**

(400 MHz, $CDCl_3$)

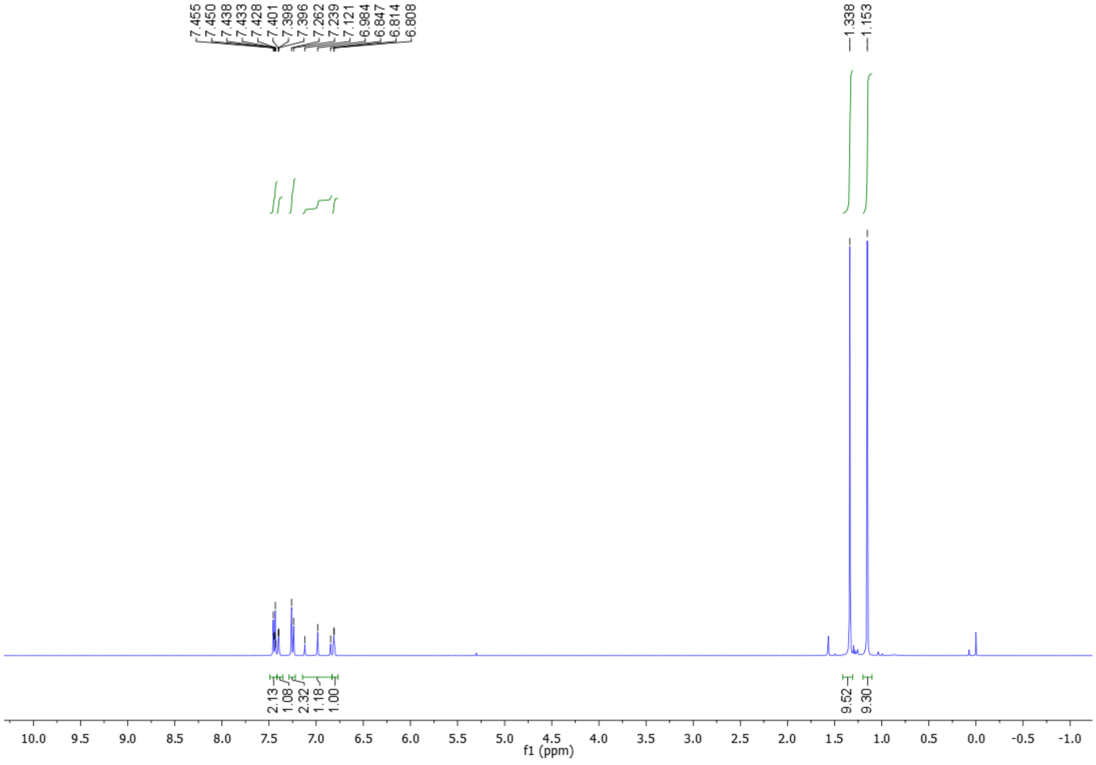

**$^{13}C\{^1H\}$ NMR**

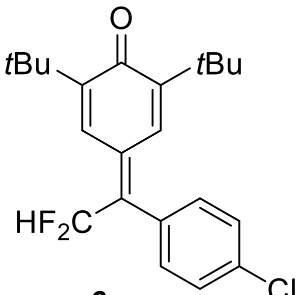


**6a**

(101 MHz, $CDCl_3$)

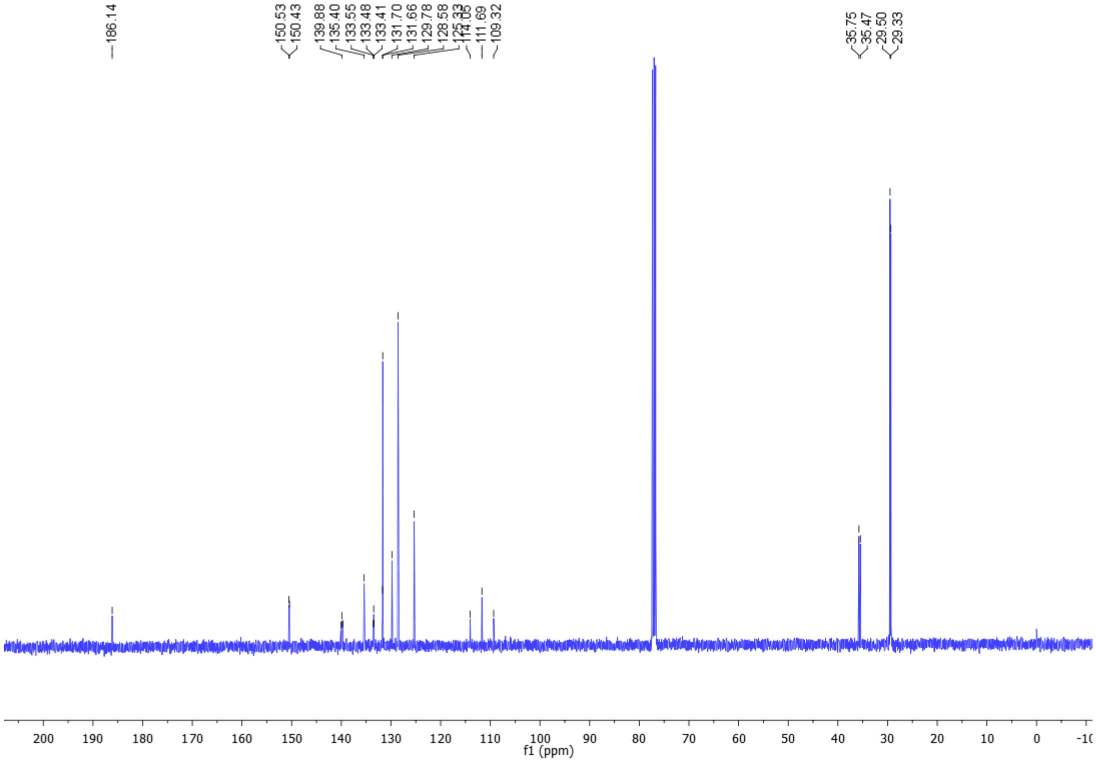

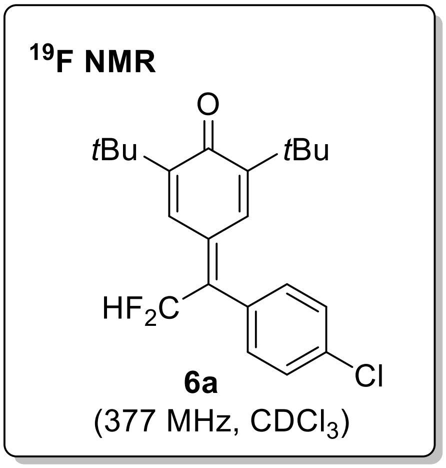

19F NMR
O
tBu
tBu
HF2C
Cl
6a
(377 MHz, CDCl3)


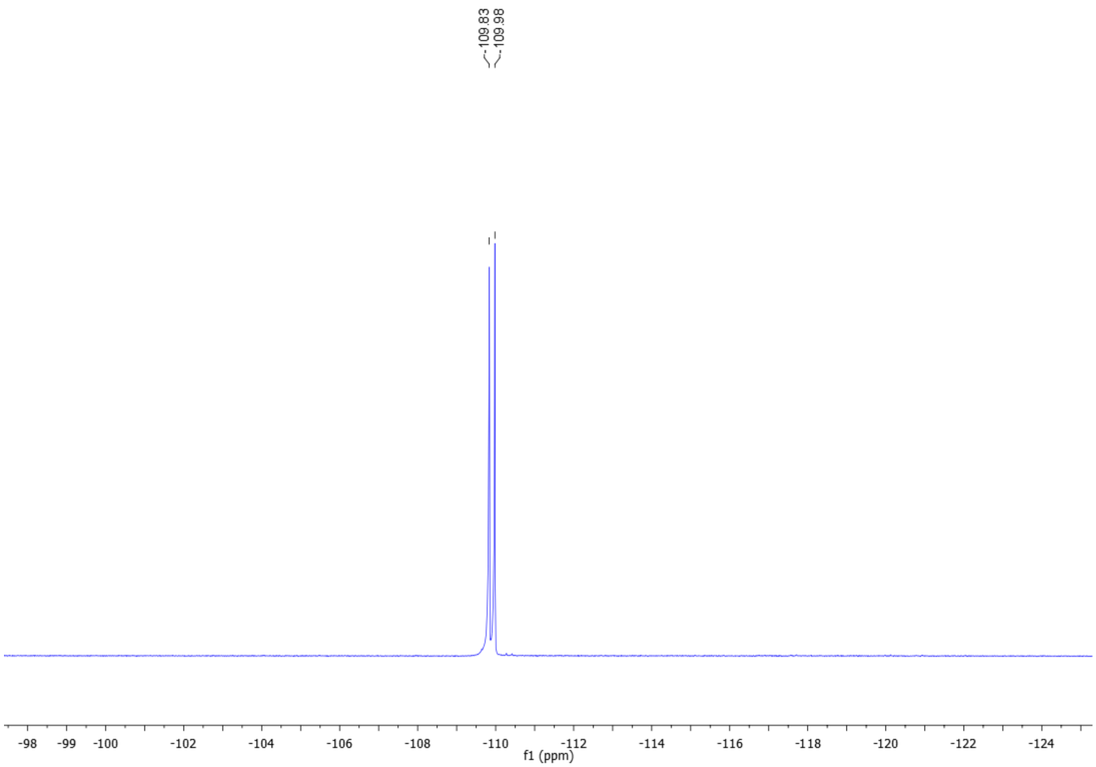

-109.83
-109.98
-98
-99
-100
-102
-104
-106
-108
-110
-112
-114
-116
-118
-120
-122
-124
f1 (ppm)

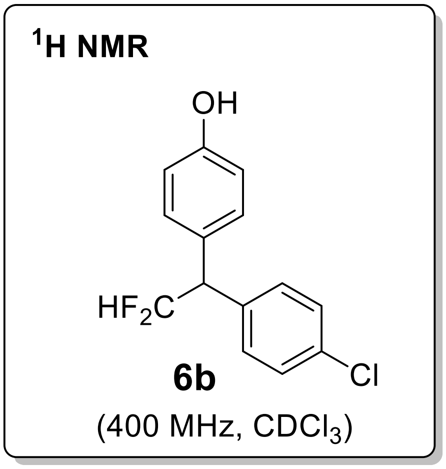
1H NMR
OH
HF2C
Cl
6b
(400 MHz, CDCl3)

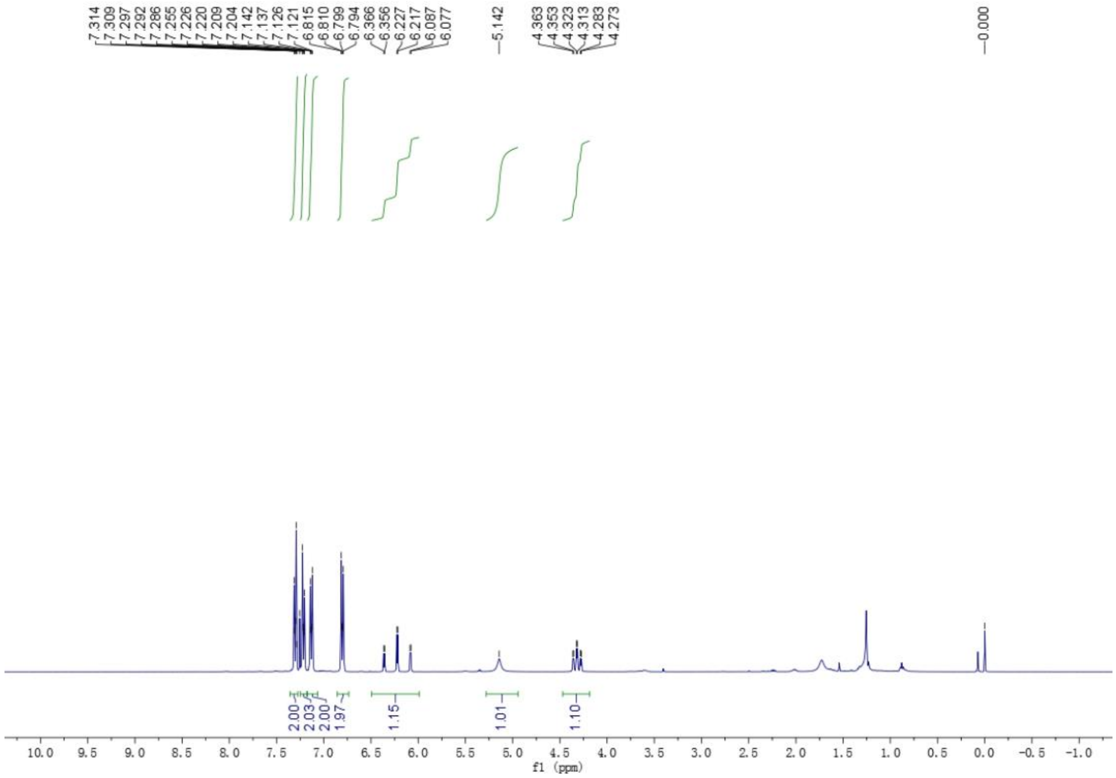
7.314
7.309
7.297
7.292
7.286
7.255
7.226
7.220
7.209
7.204
7.142
7.137
7.126
7.121
6.815
6.810
6.799
6.794
6.366
6.356
6.227
6.217
6.087
6.077
5.142
4.363
4.353
4.323
4.313
4.283
4.273
0.000
2.00
2.03
2.00
1.97
1.15
1.01
1.10
f1 (ppm)

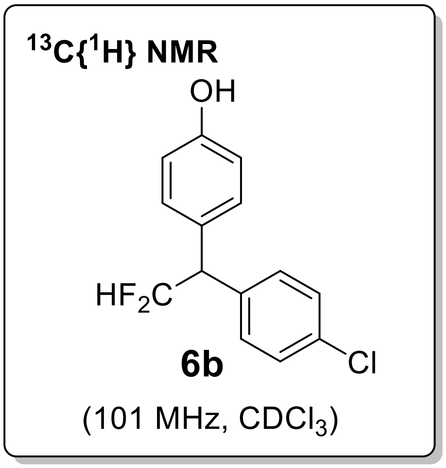
13C{1H} NMR
OH
HF2C
6b
Cl
(101 MHz, CDCl3)

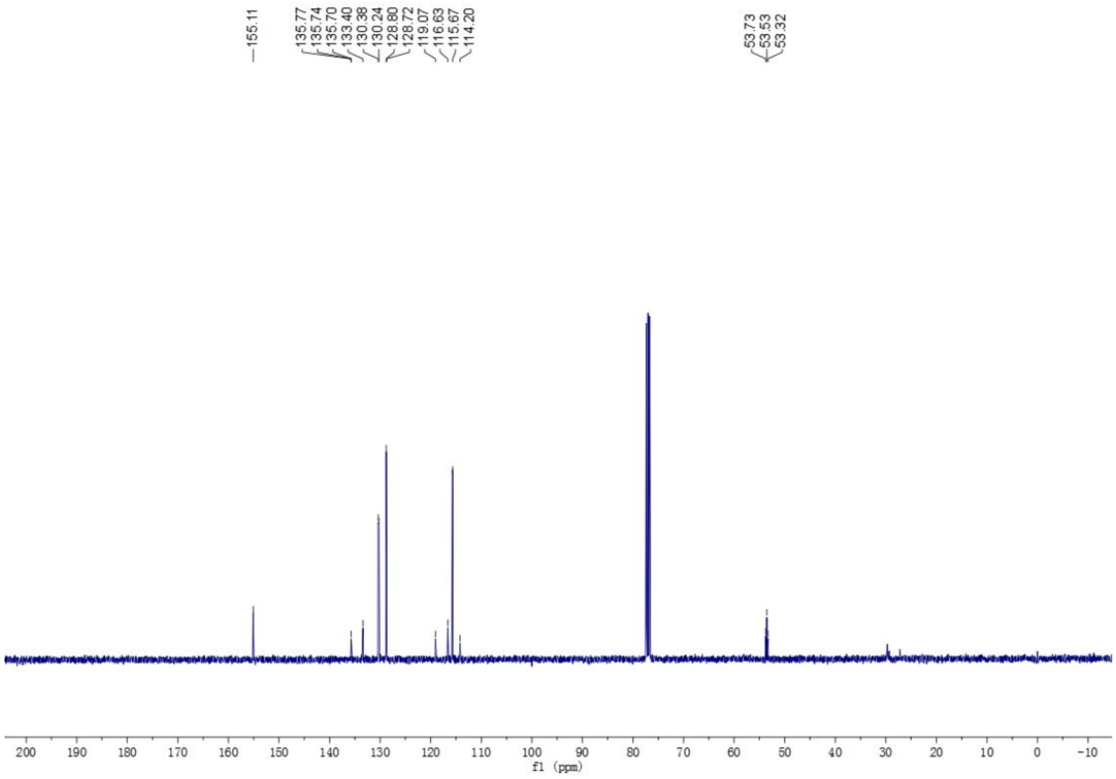
155.11
135.77
135.74
135.70
133.40
130.38
130.24
128.80
128.72
119.07
116.63
115.67
114.20
53.73
53.53
53.32
200
190
180
170
160
150
140
130
120
110
100
90
80
70
60
50
40
30
20
10
0
-10
f1 (ppm)

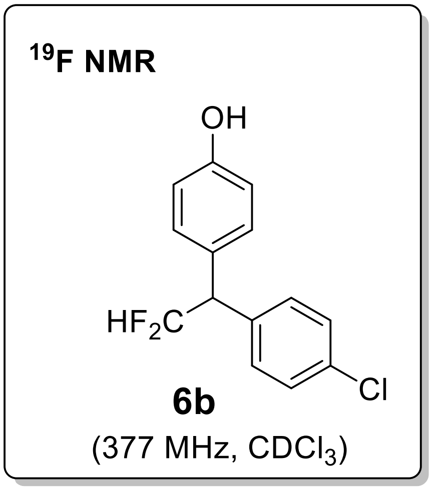

19F NMR
OH
HF2C
Cl
6b
(377 MHz, CDCl3)


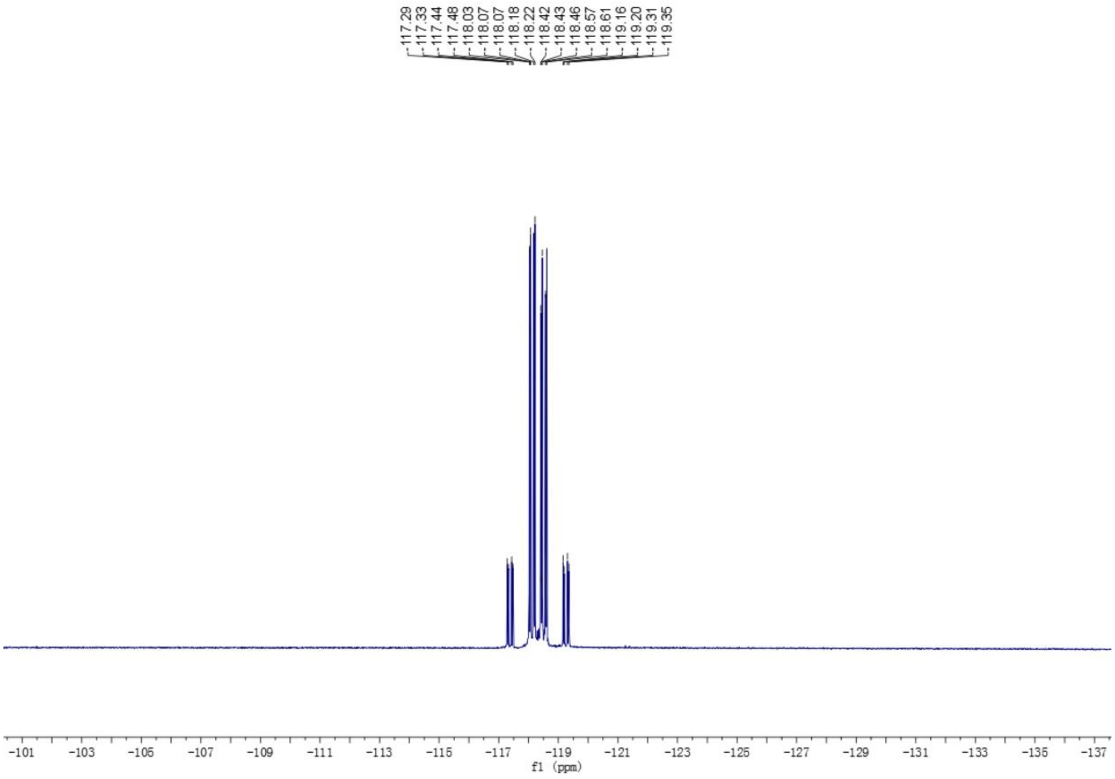

-117.29
-117.33
-117.44
-117.48
-118.03
-118.07
-118.07
-118.18
-118.22
-118.42
-118.43
-118.46
-118.57
-118.61
-119.16
-119.20
-119.31
-119.35
-101 -103 -105 -107 -109 -111 -113 -115 -117 -119 -121 -123 -125 -127 -129 -131 -133 -135 -137
f1 (ppm)

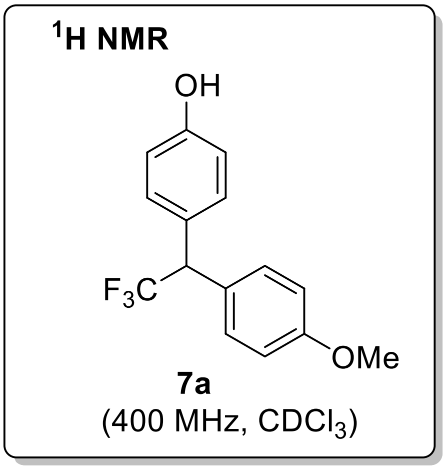
1H NMR
OH
F3C
OMe
7a
(400 MHz, CDCl3)

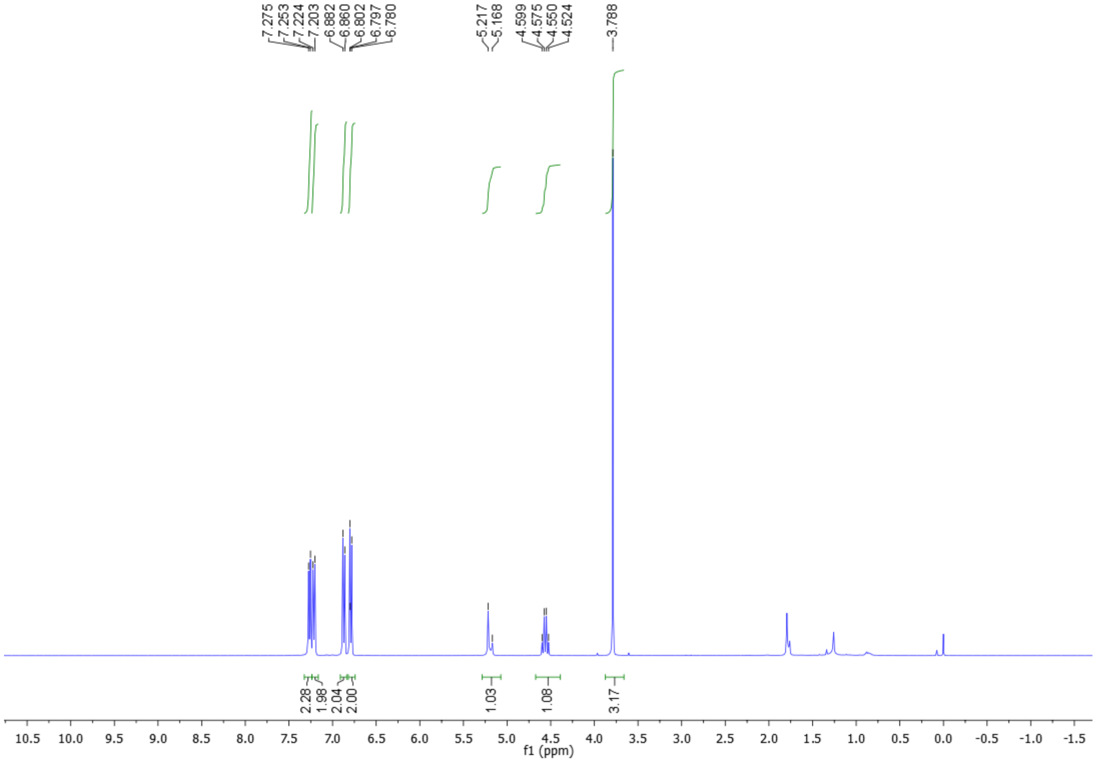
7.275
7.253
7.224
7.203
6.882
6.860
6.802
6.797
6.780
5.217
5.168
4.599
4.575
4.550
4.524
3.788
2.28
1.98
2.04
2.00
1.03
1.08
3.17
f1 (ppm)

**$^{13}C\{^1H\}$ NMR**

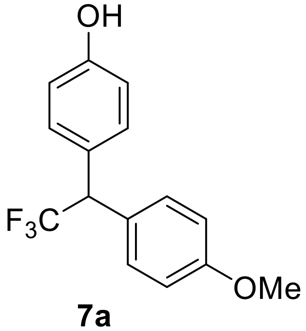


**7a**

(101 MHz, $CDCl_3$)

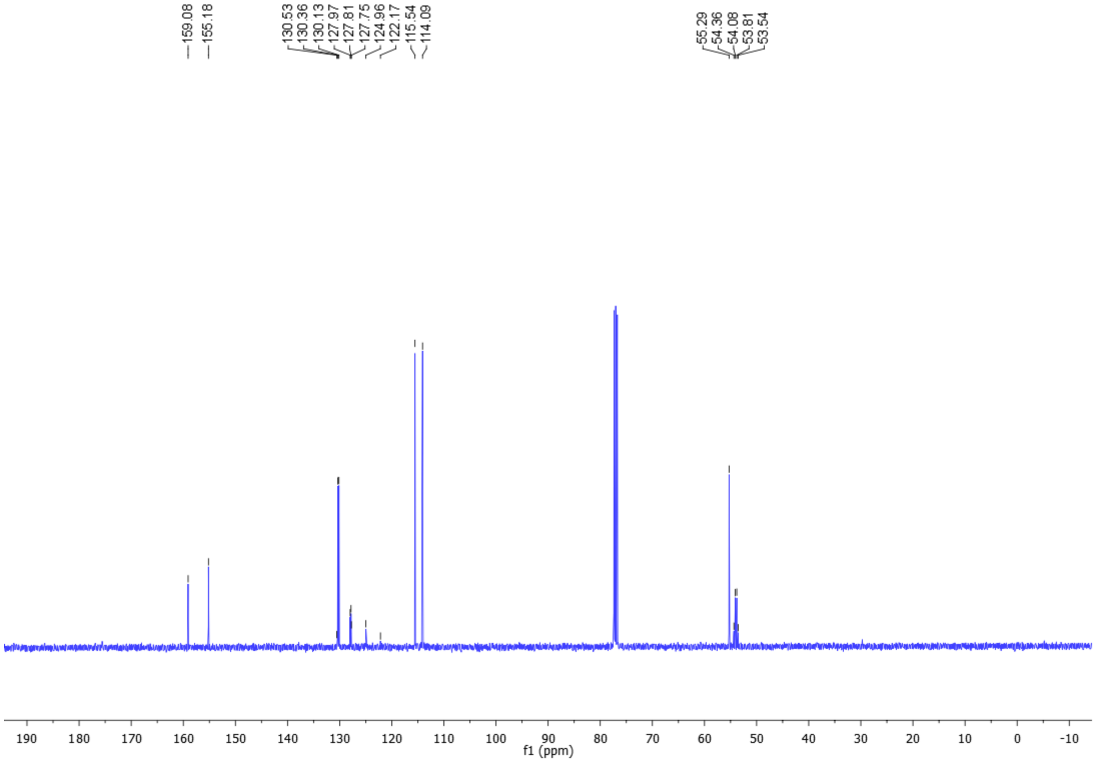

$^{19}F$ NMR

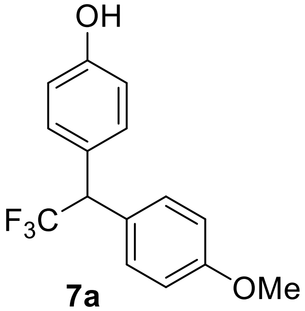


**7a**

(377 MHz, $CDCl_3$)

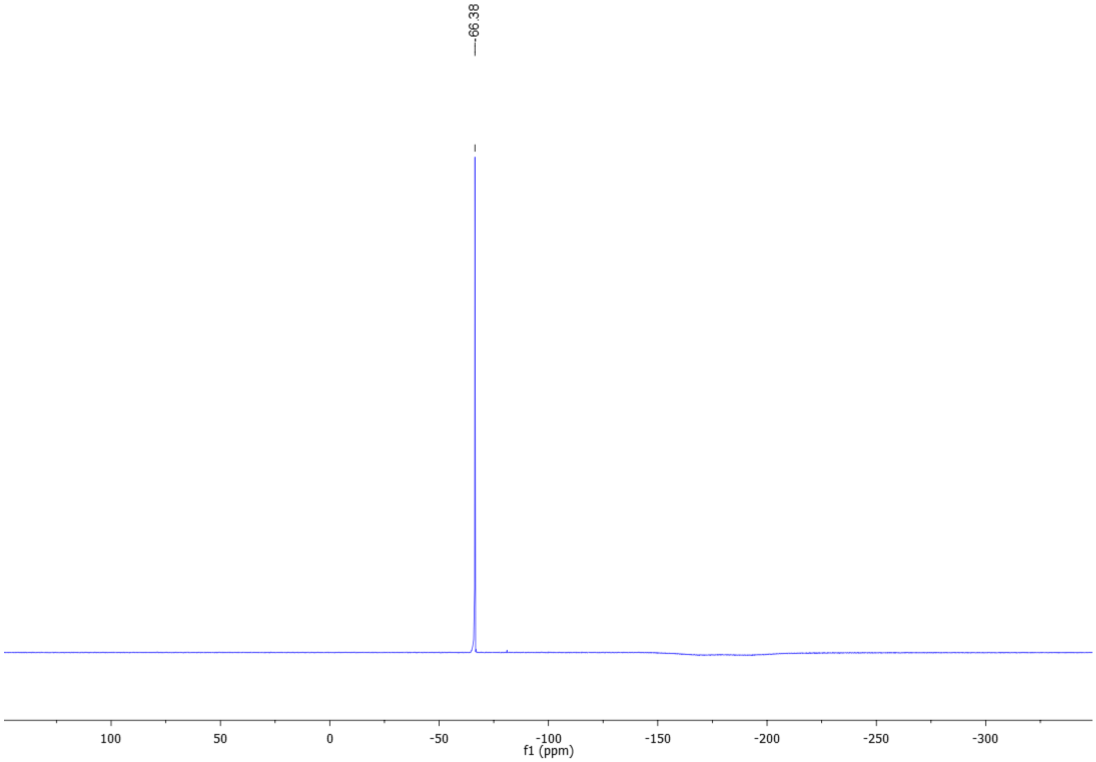

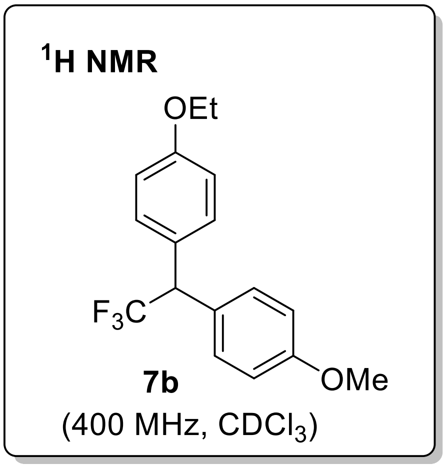

1H NMR
OEt
F3C
OMe
7b
(400 MHz, CDCl3)


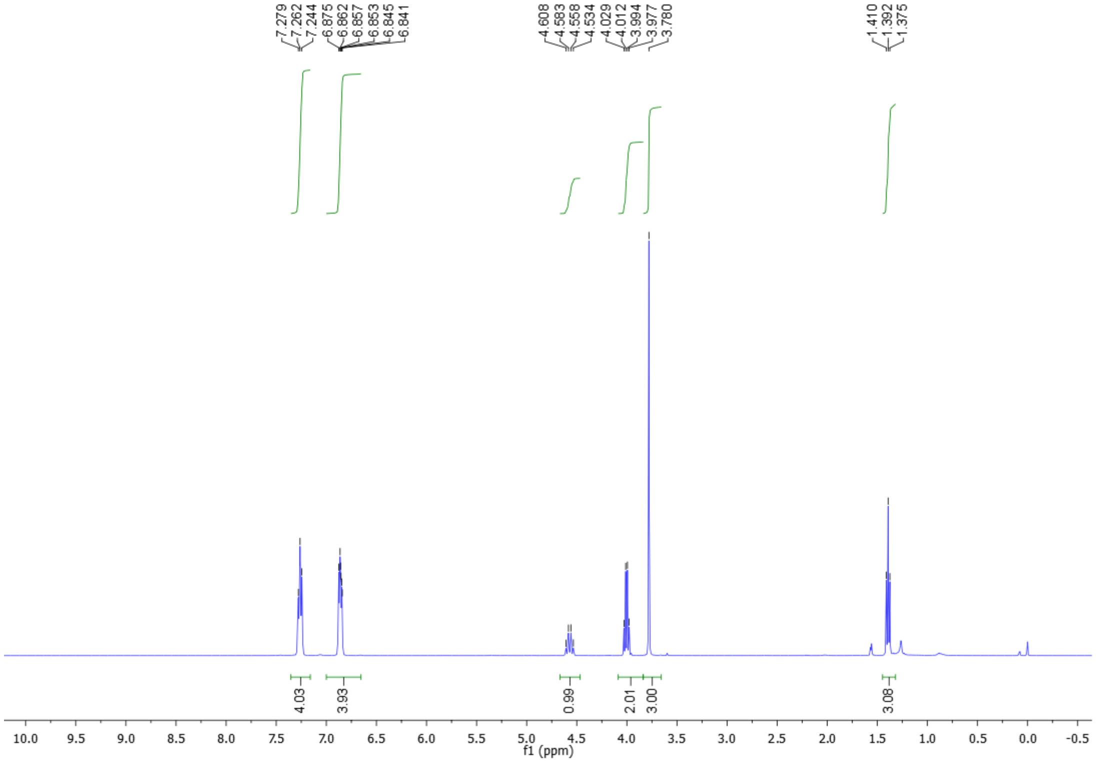

7.279
7.262
7.244
6.875
6.862
6.857
6.853
6.845
6.841
4.608
4.583
4.558
4.534
4.029
4.012
3.994
3.977
3.780
1.410
1.392
1.375
4.03
3.93
0.99
2.01
3.00
3.08
f1 (ppm)

**$^{13}C\{^1H\}$ NMR**

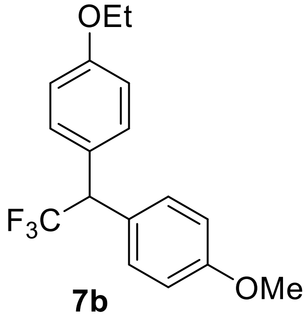


**7b**

(101 MHz, $CDCl_3$)

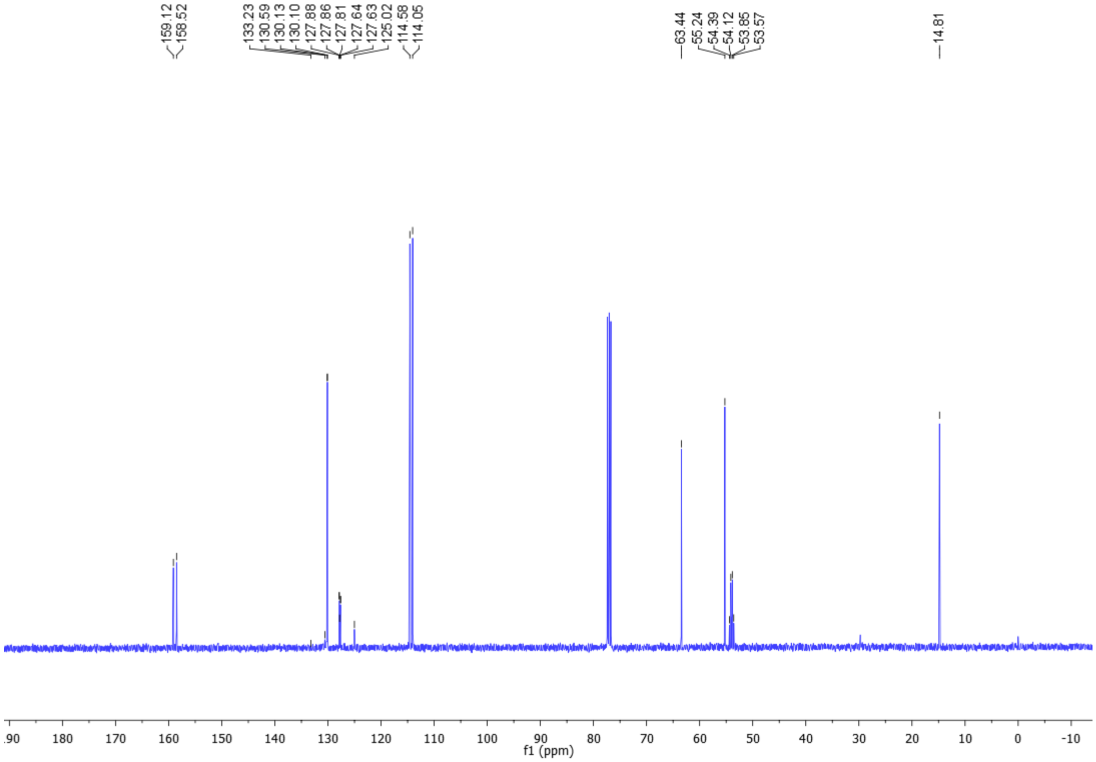

**$^{19}$F NMR**

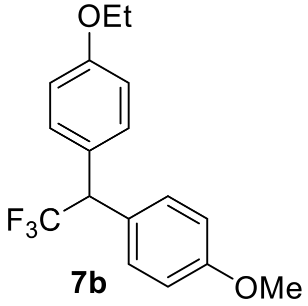


**7b**

(377 MHz, $CDCl_3$)

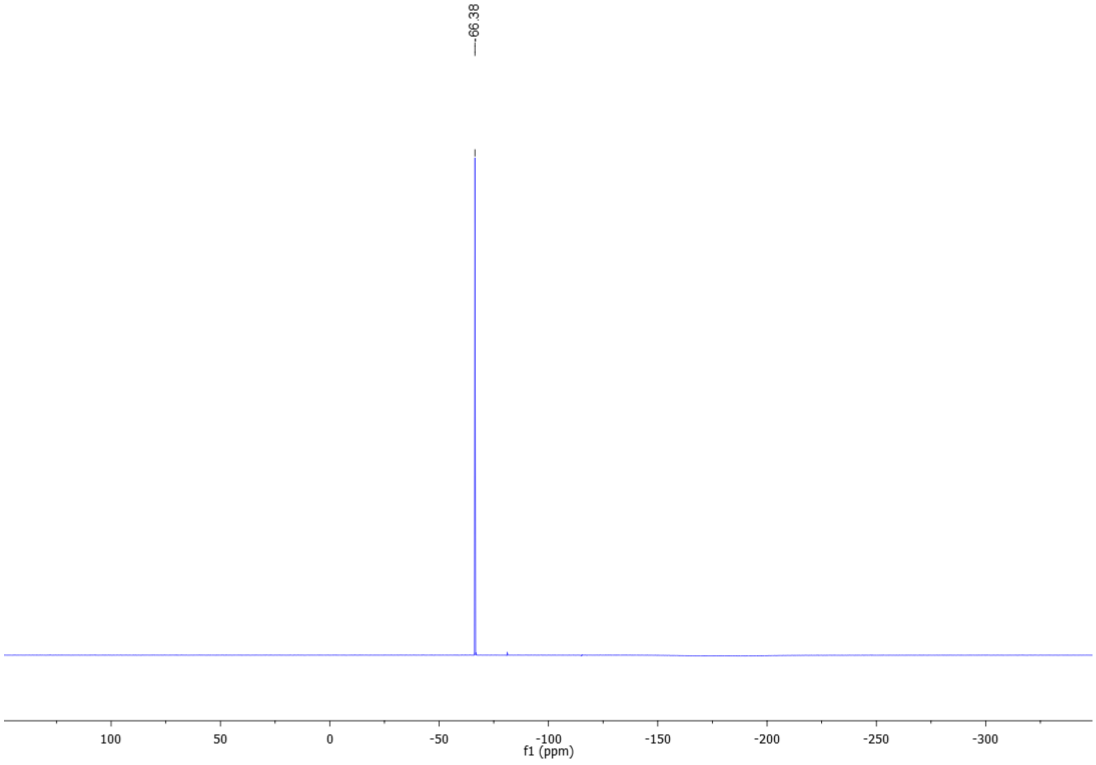

Supplement: Supplementary file 1 [file molecules-29-02905-s001.zip › molecules-2969871-supplementary.pdf]
